# Recommendations for the surveillance of cancer-related fatigue in childhood, adolescent, and young adult cancer survivors: a report from the International Late Effects of Childhood Cancer Guideline Harmonization Group

**DOI:** 10.1007/s11764-020-00904-9

**Published:** 2020-08-25

**Authors:** Salome Christen, Katharina Roser, Renée L. Mulder, Anica Ilic, Hanne C. Lie, Jacqueline J. Loonen, Anneli V. Mellblom, Leontien C. M. Kremer, Melissa M. Hudson, Louis S. Constine, Roderick Skinner, Katrin Scheinemann, Jordan Gilleland Marchak, Gisela Michel, on behalf of the IGHG psychological late effects group

**Affiliations:** 1 Department of Health Sciences and Medicine, University of Lucerne, Lucerne, Switzerland; 2 Princess Máxima Center for Pediatric Oncology, Utrecht, The Netherlands; 3 Department of Behavioural Sciences in Medicine, Institute of Basic Medical Sciences, Faculty of Medicine, University of Oslo, Oslo, Norway; 4 Department of Hematology, Radboud University Medical Center, Nijmegen, The Netherlands; 5 Departments of Epidemiology and Cancer Control, and Oncology, St. Jude Children’s Research Hospital, Memphis, USA; 6 Departments of Radiation Oncology and Pediatrics, University of Rochester Medical Center, Rochester, USA; 7 Department of Paediatric and Adolescent Haematology/Oncology, Great North Children’s Hospital and Newcastle University Centre for Cancer, Newcastle upon Tyne, UK; 8 Division of Hematology/Oncology, Department of Pediatrics, Kantonsspital Aarau, Aarau, Switzerland; 9 Division of Hematology/Oncology, University Children’s Hospital Basel and University of Basel, Basel, Switzerland; 10 Department of Pediatrics, McMaster Children’s Hospital and McMaster University, Hamilton, Canada; 11 Aflac Cancer and Blood Disorders Center, Children’s Healthcare of Atlanta, Atlanta, GA USA; 12 Department of Pediatrics, Emory University School of Medicine, Atlanta, GA USA

**Keywords:** Survivors, Childhood, adolescent, and young adult cancer, Surveillance, Late effects, Cancer-related fatigue, Evidence-based guidelines

## Abstract

**Purpose:**

Cancer-related fatigue (CRF) negatively affects the lives of childhood, adolescent, and young adult (CAYA) cancer survivors. We aimed to provide an evidence-based clinical practice guideline (CPG) with internationally harmonized CRF surveillance recommendations for CAYA cancer survivors diagnosed < 30 years.

**Methods:**

This CPG was developed by a multidisciplinary panel under the umbrella of the International Late Effects of Childhood Cancer Guideline Harmonization Group. After evaluating concordances and discordances of four existing CPGs, we performed systematic literature searches. We screened articles for eligibility, assessed quality, extracted, and summarized the data from included articles. We formulated recommendations based on the evidence and clinical judgment.

**Results:**

Of 3647 articles identified, 70 articles from 14 countries were included. The prevalence of CRF in CAYA cancer survivors ranged from 10–85%. We recommend that healthcare providers are aware of the risk of CRF, implement regular screening with validated measures, and recommend effective interventions to fatigued survivors.

**Conclusions:**

A considerable proportion of CAYA cancer survivors suffers from CRF even years after the end of treatment.

**Implications for Cancer Survivors:**

We recommend that healthcare providers adopt regular screening to detect and treat CRF early and positively influence survivors’ health and quality of life.

**Electronic supplementary material:**

The online version of this article (10.1007/s11764-020-00904-9) contains supplementary material, which is available to authorized users.

## Introduction

Thanks to advances in treatment, long-term survival of childhood, adolescent, and young adult (CAYA) cancers has improved remarkably over the past decades [1–3]. As a result, the population of CAYA cancer survivors is increasing [4]. However, most survivors experience late effects from cancer and its treatment, such as cardiovascular disease, renal dysfunction, endocrinopathies, impaired cognitive function, poor mental health, and musculoskeletal problems [4–7].

Cancer-related fatigue is a well-known problem during the active treatment phase of cancer but can also be a frequent problem for survivors many years after completion of therapy [8, 9]. The National Comprehensive Cancer Network of the USA defined cancer-related fatigue as “a distressing, persistent, subjective sense of physical, emotional and/or cognitive tiredness or exhaustion related to cancer or cancer treatment that is not proportional to recent activity and interferes with usual functioning” [10]. In survivors of adult cancers, about 30% experience cancer-related fatigue even years after completion of treatment [11, 12]. For CAYA cancer survivors, the literature on the prevalence of cancer-related fatigue (hereafter referred to as fatigue) is contradictory. A number of studies have reported a high prevalence of fatigue in CAYA cancer survivors [13–15], but other studies have observed prevalence rates or fatigue levels comparable to controls [16, 17]. Fatigue has a negative impact on many aspects of CAYA cancer survivors’ lives, such as personal relationships, school or work, and activities of daily life, and is associated with lower self-reported quality of life [9, 14, 18].

Clinical practice guidelines (CPG) could help improve consistency of care, evidence-based healthcare delivery, and thus health outcomes and quality of life in survivors [19, 20]. This is especially important as, to date, long-term follow-up (LTFU) for CAYA cancer survivors is not always well organized and few pediatric oncology institutions offer LTFU to adult survivors of CAYA cancers [21]. As many CAYA cancer survivors are followed by healthcare providers outside the pediatric oncology setting [22], CPGs can help to inform healthcare providers, as well as survivors, about cancer- and treatment-related risks such as fatigue.

In survivors of adult cancers, regular screening for fatigue is recommended [23, 24]. For CAYA cancer survivors, different groups in North America and Europe have developed LTFU CPGs to promote early detection of potential late effects [25–28]. However, these guidelines were developed independently and differ regarding their recommendations. This can cause uncertainty about which CPG to use in clinical practice and could impede the implementation of a CPG for LTFU of CAYA cancer survivors. Therefore, the International Late Effects of Childhood Cancer Guideline Harmonization Group (IGHG) was founded to harmonize CPGs for CAYA cancer survivors [29]. As the psychological late effects group of the IGHG, we aimed to harmonize the recommendations for fatigue surveillance in CAYA cancer survivors diagnosed before the age of 30.

## Methods

To develop this CPG, we utilized the international guideline harmonization methodology previously described in detail by Kremer et al. [29]. A multidisciplinary international group of 14 experts in pediatric oncology, radiation oncology, psychology, physiotherapy, epidemiology, and guideline methodology prepared the fatigue surveillance recommendations. The final recommendations were discussed with a wider group of 23 additional experts from 10 countries and reviewed by four patient stakeholders (Table S3).

### Comparison of existing guidelines

Our group of experts first evaluated concordances and discordances among the existing CPGs from the Children’s Oncology Group (COG) [25], the Dutch Childhood Oncology Group (DCOG) [26], the Scottish Intercollegiate Guidelines Network (SIGN) [27], and the United Kingdom Children’s Cancer Study Group Late Effects Group (UKCCLG) [28] regarding fatigue surveillance recommendations. In case of discordance between the CPGs, we formulated clinical questions to achieve consensus. The clinical questions addressed five key issues: (1) Who needs surveillance? (2) At what age or time from exposure should surveillance be performed? (3) At what frequency should surveillance be performed? (4) What surveillance modality should be used? and (5) What should be done when abnormalities are found?

### Search strategy and selection criteria

Systematic literature searches in MEDLINE (through PubMed), Web of Science, PsycInfo, and Scopus were performed in February 2016 and the search in MEDLINE was updated in March 2019 to identify all available evidence. The search terms “childhood cancer”, “survivors”, “late effects”, and “fatigue” with synonyms and variations were used to search the databases (detailed search strategies provided in Tables S4a-S4c). Additionally, all reference lists of included articles were hand searched (by SC). We included only papers on humans, published in English, and published between January 1990 and March 18, 2019. Studies published after March 18, 2019 were not included in this CPG. If there was no evidence available for CAYA cancer survivors, we carefully extrapolated evidence from survivors of adult cancers. This evidence was identified by using the same search strategy but by replacing the “childhood cancer” term with “cancer”.

Two authors first independently screened titles and abstracts and excluded irrelevant articles (SC, KR, HCL, JJL, AVM, KS, and GM participated in the title/abstract screening). In a second step, two authors independently assessed the eligibility of the full-text articles (SC, KR, HCL, JJL, AVM, KS, and GM participated in the full-text screening). Inclusion criteria were (1) typical childhood, adolescent, or young adult cancer diagnosis; (2) ≥ 75% of study participants were < 30 years at cancer diagnosis; (3) survivors (≥ 50% of study participants were ≥ 2 years from diagnosis); (4) sample size ≥ 20 participants (detailed inclusion and exclusion criteria in Table S5). Throughout this manuscript, “survivor” is, therefore, defined as being at least 2 years since diagnosis. In case of a disagreement between the two authors, a third author helped finding consensus regarding inclusion or exclusion of the article (KS or GM). Next, we extracted relevant information of the articles into evidence tables. One article could address more than one clinical question. Corresponding authors were contacted in the event of missing primary data. We assessed the quality of the included articles by evidence-based methods provided by the Cochrane Childhood Cancer (Table S6). For every clinical question, we formulated and graded a conclusion of evidence based on an adapted version of the *Grading of Recommendations Assessment Development and Evaluation criteria* (GRADE; Table S7) [29, 30].

### Translating evidence into recommendations

Recommendations were based on consideration of the evidence, costs, benefits versus harms of the surveillance intervention, the need to maintain the flexibility of application across different healthcare systems, and clinical judgment. Decisions were made through group discussion and consensus, and final recommendations were supported unanimously. The strength of the fatigue recommendations was graded according to published evidence-based methods (Table S8) [31]. The harmonized fatigue surveillance recommendations were discussed with a wider group of additional 23 experts (Table S3) from 10 countries via teleconference and critically appraised by four survivor representatives (Table S3) via electronic communications. We used the feedback from these discussions for the finalization of the recommendations. The recommendations will be updated within 5 years.

## Results

Comparing the four existing surveillance recommendations for fatigue, we found they were discordant in all areas (Table S9). Based on the discordances, nine clinical questions were formulated to investigate the evidence in more detail (Table S10). The evidence tables (Table S11) and detailed conclusions of evidence (Table S12) are presented as Supplementary Material.

Of 3647 studies identified, 530 full texts were screened and 70 articles were eligible for the fatigue surveillance recommendations (Fig. S1), with a total sample of *n* = 11,628 CAYA cancer survivors. One CPG and four systematic reviews were included. The 65 original studies were conducted in 14 different countries in Asia (12 studies), Europe (21 studies), North America (30 studies), and South America (two studies). The conclusions of evidence and the recommendations are presented in Tables 1 and 2.

**Table 1:**
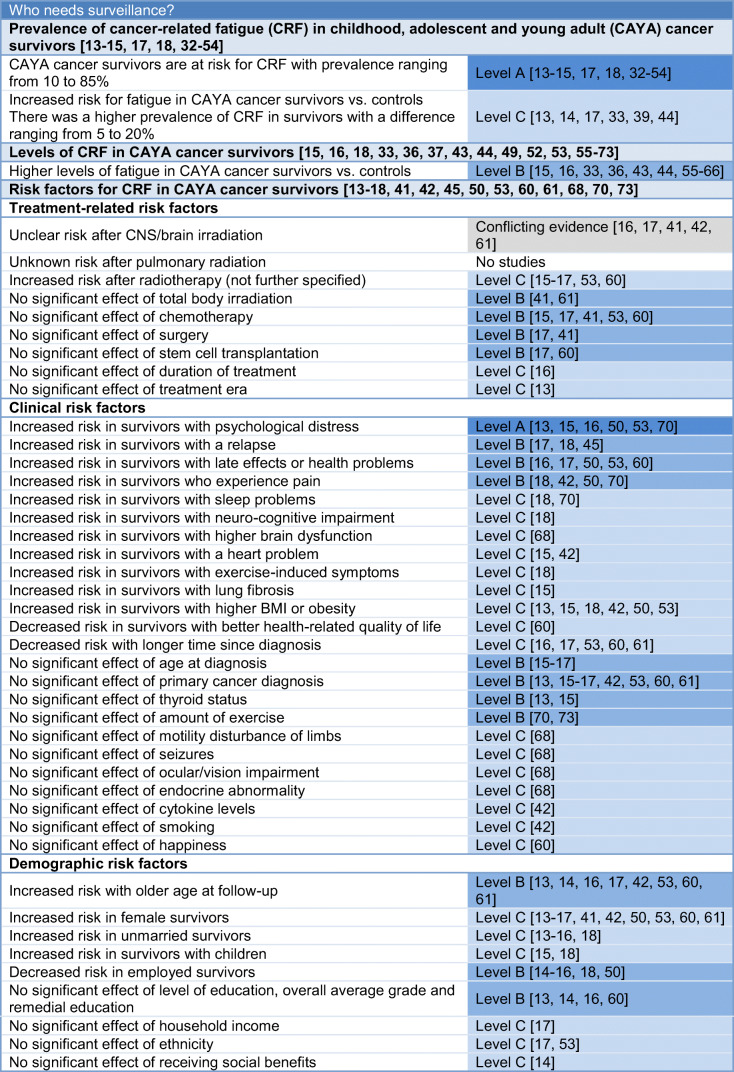

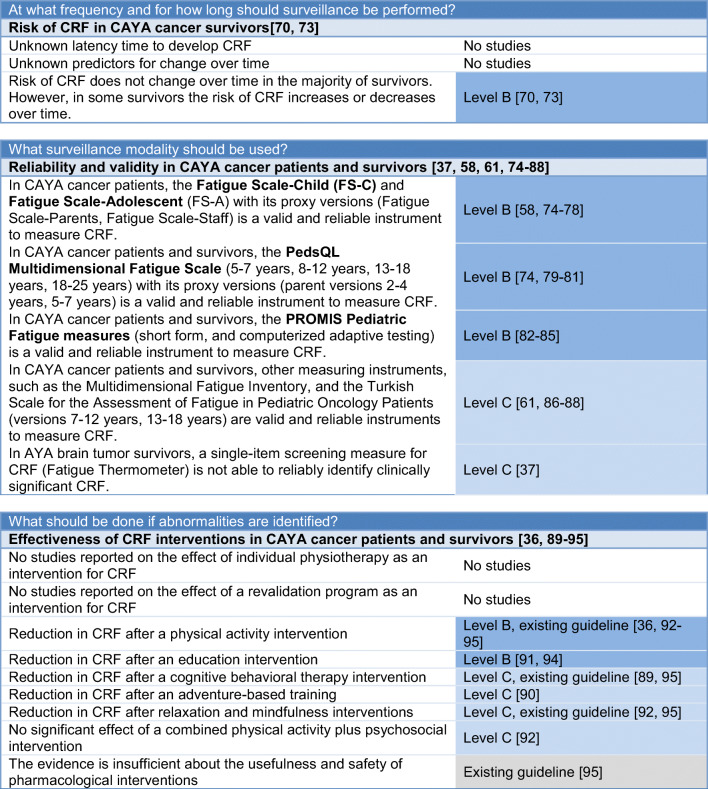
Overall conclusions of the evidence

**Table 2:**
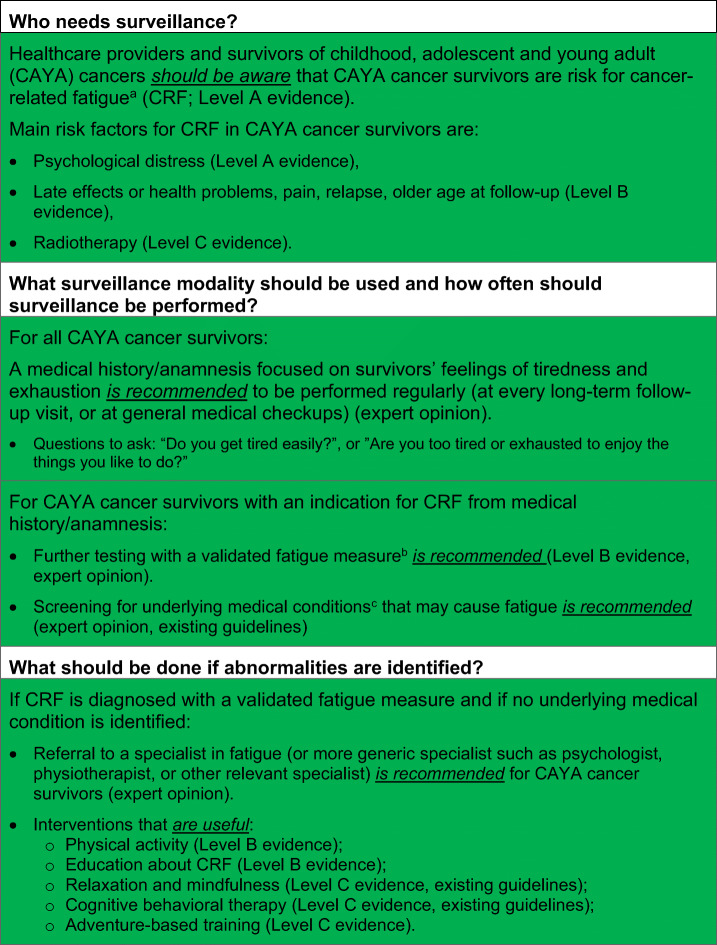
Surveillance recommendations for cancer-related fatigue in survivors of childhood, adolescent and young adult cancers (see Fig. 1 for process of CRF surveillance)

^a^CRF is defined as “a distressing, persistent, subjective sense of physical, emotional, and/or cognitive tiredness or exhaustion related to cancer or cancer treatment that is not proportional to recent activity and interferes with usual functioning” [10].

^b^Ideally the *PROMIS Pediatric Fatigue measure* (http://www.healthmeasures.net/index.php?Itemid=992 [accessed August 29th 2019]) or the *PedsQL Multidimensional Fatigue Scale* (https://eprovide.mapi-trust.org/instruments/pediatric-quality-of-life-inventory-multidimensional-fatigue-scale [accessed August 29th 2019]); see Table S13 for list of all measures validated in CAYA cancer patients and survivors

^c^e.g., other late effects like cardiac dysfunction, [96] endocrine dysfunction, pulmonary dysfunction, and renal dysfunction (IGHG guidelines under development); and/or other general causes like anemia, arthritis, neuromuscular complications, pain, fever and/or infection, and nutritional deficiencies [24, 97] (list not conclusive)

### Who needs surveillance?

#### Evidence on the risk of fatigue

The existing CPGs were discordant as only two specified the population at risk (“all survivors”), [25, 26] and only one surveillance recommendation identified risk factors for fatigue in CAYA cancer survivors (Table S9) [25].

There is evidence that CAYA cancer survivors are at risk for fatigue (level A; Table 1) [13–15, 17, 18, 32–54]. We found 28 articles (24 studies) that reported the prevalence of fatigue in CAYA cancer survivors (Table S2) [13–15, 17, 18, 32–54]. Prevalence of fatigue ranged from 10.2 to 85.0% over all 24 studies [13–15, 17, 18, 32–37, 39–41, 44–49, 51–54].

Some evidence suggests that there is an increased risk for fatigue in CAYA cancer survivors compared with controls (level C; Table 1) [13, 14, 17, 33, 39, 44]. Five studies found that the prevalence of fatigue is higher in survivors than controls with a difference ranging from 5 to 20% (two were statistically significant at *p* < 0.05; Table S2) [13, 14, 33, 39, 44]. One study reported a lower prevalence of fatigue in survivors that was not significantly different from that of community norms [17].

Evidence suggests higher levels of fatigue in survivors of CAYA cancer compared with controls (level B; Table 1) [15, 16, 33, 36, 43, 44, 55–66]. Of 30 articles (29 studies) describing a level of fatigue in CAYA cancer survivors [15, 16, 18, 33, 36, 37, 43, 44, 49, 52, 53, 55–73], ten articles reported the level of fatigue in CAYA cancer survivors only [18, 37, 49, 52, 53, 68, 69, 71–73], one was a case-control study with non-fatigued survivors as controls [70], and one used survivors of adult cancers as controls [67]. The other 18 articles compared levels of fatigue in survivors with healthy controls (Table S2) [15, 16, 33, 36, 43, 44, 55–66]. In twelve of these articles, survivors had statistically significant higher levels of fatigue compared with that of controls [15, 33, 36, 43, 44, 55, 56, 58, 62, 64–66], whereas two studies reported statistically significant lower levels of fatigue in survivors compared with controls [60, 61], and four studies reported no statistical difference between groups [57, 59, 63] or conflicting evidence [16].

#### Evidence on risk factors for fatigue

One existing CPGs identified pulmonary radiation as a major risk factor for developing fatigue [25]. However, we identified no studies investigating the risk of fatigue after pulmonary radiation in CAYA cancer survivors.

We identified 16 studies that evaluated risk factors for fatigue in survivors of CAYA cancers [13–18, 41, 42, 45, 50, 53, 60, 61, 68, 70, 73]. The only treatment-related risk factor associated with an increased risk for fatigue was any radiotherapy (level C; Table 1) [15–17, 53, 60]. No significant associations were found between fatigue and the following treatment-related risk factors: total body irradiation [41, 61], chemotherapy [15, 17, 41, 53, 60], surgery [17, 41], stem cell transplantation [17, 60] (all level B), duration of treatment [16], and treatment era [13] (both level C).

The following clinical risk factors were associated with an increased risk of fatigue: psychological distress (level A) [13, 15, 16, 50, 53, 70], a relapse [17, 18, 45], late effects or health problems [16, 17, 50, 53, 60], and pain [18, 42, 50, 70] (all level B). The following clinical risk factors were associated with a decreased risk of fatigue: longer time since diagnosis [16, 17, 53, 60, 61] and better health-related quality of life [60] (both level C). No significant associations were found between fatigue and the following clinical risk factors: age at diagnosis [15–17], primary cancer diagnosis [13, 15–17, 42, 53, 60, 61], thyroid status [13, 15], and amount of exercise [70, 73] (all level B).

The following demographic risk factors were associated with an increased risk for fatigue: older age at follow-up (level B) [13, 14, 16, 17, 42, 53, 60, 61], female sex [13–17, 41, 42, 50, 53, 60, 61], not being married/in a relationship [13–16, 18], and having children [15, 18] (all level C). Being employed was associated with a decreased risk for fatigue (level B) [14–16, 18, 50]. No significant associations were found between fatigue and the following demographic risk factors: level of education (level B) [13, 14, 16, 60], household income [17], ethnicity [17, 53], and receiving social benefits [14] (all level C).

### At what age or time from exposure should surveillance be performed?

#### Evidence on latency time

The existing CPGs were discordant; only two specified when surveillance for fatigue should start (2 years after the end of treatment [25] or at the late effects outpatient clinic [26]). We found no studies that reported on the latency time to develop fatigue in survivors of CAYA cancers.

### At what frequency should surveillance be performed?

#### Evidence on change of risk over time

The existing CPGs were discordant as only two specified the interval for fatigue surveillance (one recommends yearly surveillance [25], the other surveillance every 5 years [26]).

We identified two studies that reported unchanged risk for fatigue over time in the majority of CAYA cancer survivors but that fatigue status can change over time in some survivors (level B) [70, 73]. One study showed that long-term survivors can be persistently fatigued or persistently non-fatigued but also that fatigue status can change over time: a median of 2.7 years after the first assessment, 39.6% of former fatigue cases were no longer fatigued and 18.4% of former non-fatigue cases became fatigued [70]. The other study showed that mean levels of fatigue did not change significantly from end of treatment to 36 months post-therapy [73].

### What surveillance modality should be used?

#### Evidence on validity and reliability of fatigue measures

Only one existing CPG specified a measure that should be used for fatigue surveillance [26]. More general surveillance recommendations (screen for an underlying medical condition, psychosocial assessment) were made by two existing CPGs [25, 26].

In our systematic search, we found 17 studies and one systematic review that assessed the psychometric properties of seven different measures for fatigue in CAYA cancer survivors [37, 58, 61, 74–88]. Ten studies measured fatigue in CAYA cancer patients [74, 76–80, 82, 83, 86, 87], five in CAYA cancer survivors [37, 61, 81, 84, 88], and three in a mixed patient and survivor population [58, 75, 85]. Five studies and the systematic review investigated the Fatigue Scales (Fatigue Scale-Child, Fatigue Scale-Adolescent, and proxy versions) [58, 74–78], four studies the PROMIS Pediatric Fatigue Measures [82–85], three studies and the systematic review the PedsQL Multidimensional Fatigue Scale (Peds QL MFS) [74, 79–81], and five studies other measures (Fatigue Thermometer [37], Multidimensional Fatigue Inventory [88] Turkish Scale for the Assessment of Fatigue in Pediatric Oncology Patients [86, 87], and 12-item fatigue questionnaire [61]).

Evidence suggests that the Fatigue Scales, the PROMIS pediatric fatigue measures, and the PedsQL MFS are valid and reliable measures to diagnose fatigue in patients and survivors of CAYA cancers (level B; Table 1) [58, 74–85]. There is some evidence suggesting other measures, such as the Turkish Scale for the Assessment of Fatigue in Pediatric Oncology Patients and a 12-item fatigue questionnaire, are valid and reliable in measuring fatigue in patients and survivors of CAYA cancers (level C) [61, 86–88]. One study found that a single-item screening measure for fatigue is not able to reliably identify clinically significant fatigue in CAYA brain tumor survivors (level C) [37]. A list of the fatigue measures validated in patients or survivors of CAYA cancers is provided in Table S13.

### What should be done when abnormalities are found?

Only one existing surveillance recommendation specified possible interventions for fatigue in CAYA cancer survivors, namely individual cognitive therapy, a revalidation program, or individual physiotherapy [26].

We identified no studies reporting on a revalidation program or individual physiotherapy in the treatment of fatigue in CAYA cancer survivors. We found one study that investigated a cognitive-behavioral intervention in CAYA cancer survivors [89]. Two studies in CAYA cancer survivors [36, 90], one study in CAYA cancer patients [91], and three systematic reviews in CAYA cancer patients and survivors [92–94] investigated effects of other fatigue interventions. In addition, we identified one CPG on the management of fatigue among CAYA cancer patients [95].

Evidence suggests that physical activity interventions (e.g., aerobic, yoga, or combined) [36, 92–95] and education interventions lead to a reduction in fatigue in CAYA cancer survivors (both level B; Table 1). Some evidence suggests that adventure-based training (group activities including rock climbing and team building games) can improve fatigue in CAYA cancer survivors (level C) [90]. In addition, some evidence suggests that relaxation and mindfulness interventions (e.g., acupressure, massage, mindfulness) resulted in a reduction in fatigue (level C, existing guideline) [92, 95]. To date, there is insufficient evidence about the usefulness and safety of pharmacological fatigue interventions in CAYA cancer patients and survivors (existing guidelines) [95].

### Translating evidence into recommendations

Based on the evidence and group consensus, the panel recommends that healthcare providers and survivors should be aware of CAYA cancer survivors’ risk for fatigue (strong recommendation based on level A evidence; Table 2). The main risk factors for fatigue in CAYA cancer survivors are psychological distress, late effects or health problems, pain, relapse, older age at follow-up, and radiotherapy (strong recommendation based on levels A–C evidence; Table 2).

Many CAYA cancer survivors are not in regular LTFU [98–100] but should be screened for fatigue nevertheless. If survivors are not in LTFU care, they should be screened for fatigue at general medical checkups. In the case of fatigue, screening is not expected to be overly burdensome for the survivors nor are false-positive screening results and subsequent examinations. However, screening for fatigue in the regular LTFU appointment, including potential false-positive screening results with subsequent examinations and referrals, could result in slightly higher costs of LTFU care. Based on the identification of a broad range of risk factors, the knowledge of the high prevalence and high levels of fatigue among CAYA cancer survivors, and the low burden for survivors, the expert panel decided to strongly recommend surveillance of fatigue for all survivors. Based on the uncertainty regarding the risk of fatigue over time, the panel recommends lifelong screening for fatigue.

**Fig. 1:**
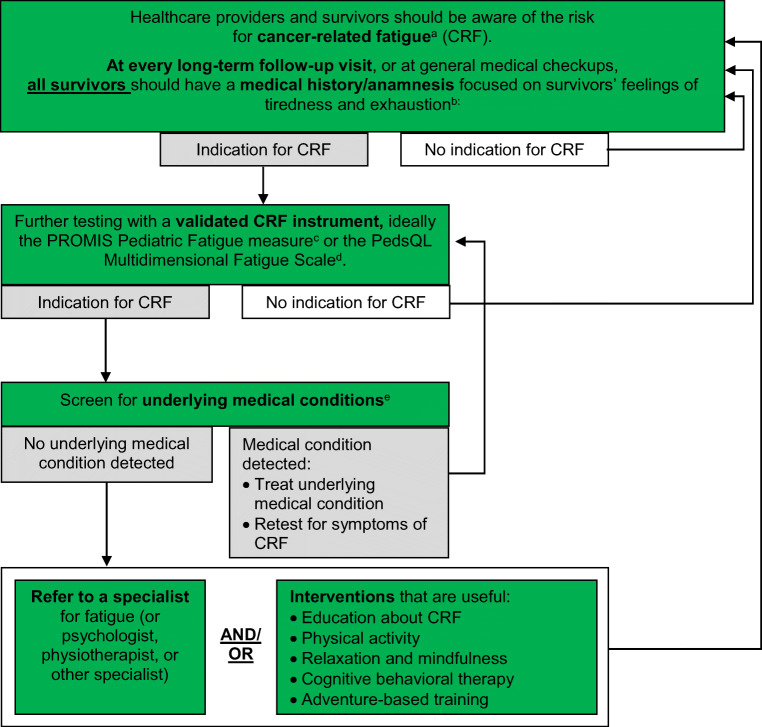
Process of screening and interventions for cancer-related fatigue in survivors of childhood, adolescent, and young adult cancers. The green color indicates a strong recommendation to do. Superscript letter “a”: cancer-related fatigue is defined as “a distressing, persistent, subjective sense of physical, emotional and/or cognitive tiredness or exhaustion related to cancer or cancer treatment that is not proportional to recent activity and interferes with usual functioning” [10]. Superscript letter “b”: questions to ask: “Do you get tired easily?” or “Are you too tired or exhausted to enjoy the things you like to do?” Superscript letter “c”: http://www.healthmeasures.net/index.php?Itemid=992 [accessed July 1, 2019]. Superscript letter “d”: https://eprovide.mapi-trust.org/instruments/pediatric-quality-of-life-inventory-multidimensional-fatigue-scale [accessed July 1st 2019]. Superscript letter “e”: e.g., other late effects like cardiac dysfunction, [96] endocrine dysfunction, pulmonary dysfunction, and renal dysfunction (IGHG guidelines under development); and/or other general causes like anemia, arthritis, neuromuscular complications, pain, fever and/or infection, and nutritional deficiencies [24, 97] (list not conclusive)

Existing CPGs for fatigue surveillance in survivors of adult cancers recommend a two-step assessment: first, screen for fatigue with a numerical rating scale (NRS; 0–10 scale) and perform further assessment if NRS ≥ 4 [24, 97]. The panel decided to use a similar approach, but not to use a one-item screening measure for fatigue, because it may not reliably identify clinically significant fatigue in survivors of CAYA cancers [37]. Therefore, we recommend that, for all CAYA cancer survivors, healthcare providers should perform a medical history/anamnesis focused on the survivor’s feelings of tiredness and exhaustion at every regular long-term follow-up visit or at general medical checkups (strong recommendation based on expert opinion; Table 2). If there is an indication for the presence of fatigue, the panel recommends further testing with a validated fatigue measure, ideally with the PROMIS Pediatric Fatigue Measures or the PedsQL MFS (strong recommendation based on level B evidence and expert opinion; Table 2). A description of the surveillance process is presented in Fig. 1.

We based our screening recommendations on several considerations. First, the measure has to demonstrate validity and reliability in CAYA cancer patients or survivors. Second, extensive assessments may not be feasible to implement in clinical practice. Third, to be useful along the cancer survivorship trajectory of CAYA cancer survivors, it is important that the measure has versions for different age groups, including a version for adult survivors. Fourth, the measure needs to be readily available for healthcare providers. Fifth, the measure needs to be available in different languages because it will only be used when available in the country’s language. The PROMIS Pediatric Fatigue Measure and the PedsQL MFS represent two assessment measures with these qualities (Table S13). Both measures are validated and demonstrate good psychometric properties, both have short versions (10–18 items), versions for different age groups including adult survivors (PedsQL MFS-Young Adult and Adult; PROMIS Fatigue measure), are available online and free of cost for clinicians, and have been translated into various languages.

There is a spectrum of health problems that can cause fatigue symptoms (e.g., late effects such as cardiac dysfunction [96], endocrine dysfunction, pulmonary dysfunction, and renal dysfunction (IGHG guidelines under development); and/or other health problems such as anemia, arthritis, neuromuscular complications, pain, fever and/or infection, and nutritional deficiencies) [24, 97]. Therefore, if there is an indication for the presence of fatigue in a CAYA cancer survivor, the panel recommends to screen for underlying medical conditions (strong recommendation based expert opinion; Table 2).

If no underlying medical condition is identified, the referral of fatigued CAYA cancer survivors to a specialist in fatigue (or a more generic specialist such as psychologist, physiotherapist, or another relevant specialist) is recommended (strong recommendation based on expert opinion; Table 2). Additionally, the panel recommends that all fatigued survivors should be provided with information about fatigue and strategies for the management of fatigue symptoms. Healthcare providers should also encourage survivors to engage in interventions that have been shown to be effective in reducing fatigue, such as physical activity and adventure-based training, relaxation and mindfulness interventions, and cognitive behavioral therapy (strong recommendation based on levels B and C evidence and existing guidelines; Table 2, Fig. 1).

## Discussion

We summarize the harmonized recommendations for fatigue surveillance in CAYA cancer survivors diagnosed before the age of 30 years. The recommendations were developed using scientifically rigorous methods, are based on evidence from published literature, and are supplemented by expert opinion for topics with little or no evidence. The recommendations are intended to standardize and improve the quality of LTFU care for CAYA cancer survivors and to positively influence fatigue outcomes in survivors. The panel also aimed to raise awareness about CAYA cancer survivors’ risk of fatigue among healthcare providers and CAYA cancer survivors and empower survivors to make informed health choices.

We found that the prevalence of fatigue in survivors of CAYA cancers varied widely from 10 to 85%. Several factors explain this variability. First, we included 28 studies from 14 different countries on three continents (America, Europe, and Asia) with different cultural backgrounds. Then, twelve of the 28 included studies used a tool for assessment of fatigue that was neither standardized nor validated. The other 16 studies used seven different measures to determine the prevalence of fatigue. Additionally, the populations of the included studies varied considerably regarding sample size, follow-up time, included primary diagnoses, and age at diagnosis. The use of so many different assessments for fatigue and heterogeneity in study populations likely contributed to the large differences in the prevalence of fatigue in CAYA cancer survivors.

Despite a sizable number of studies that reported risk factors for fatigue in CAYA cancer survivors, the level of evidence for the recommendations is mainly moderate to low. We found no studies that investigated the latency time to develop fatigue and only two studies that investigated the clinical course of fatigue in CAYA cancer survivors. Future studies should focus on high-quality research to investigate the risk of and risk factors for fatigue in CAYA cancer survivors using scientifically validated fatigue measures (preferably PROMIS Pediatric Fatigue Measure or PedsQL MFS) in CAYA cancer survivors and especially in older adult survivors of CAYA cancers. In addition, longitudinal assessment of fatigue in CAYA cancer patients and survivors is needed to identify the change of fatigue patterns over time (Table 3).

**Table 3:** Gaps in knowledge and future directions for research

• High-quality research on risk of fatigue and risk factors for fatigue in CAYA cancer survivors using scientifically validated fatigue measurements (PROMIS Pediatric Fatigue, PedsQL Multidimensional Fatigue Scale) • Longitudinal studies characterizing the course of fatigue in CAYA cancer patients and survivors and indicators for change • Investigations of the impact of aging and elapsed time from diagnosis on risk for fatigue • Investigations evaluating the risk for fatigue after CNS/brain irradiation • Evaluation of the reliability and validity of a 1-item screening tool for fatigue in CAYA cancer survivors (mixed diagnoses) and parents of very young survivors • Psychometric validation of fatigue measures in adult CAYA cancer survivors • Determination of clinically significant thresholds for fatigue measures • High-quality randomized controlled trials with larger samples to assess the effectiveness of fatigue interventions in CAYA cancer survivors. • Identify the most effective interventions for different age groups (pediatric survivors, adolescent survivors, young adult survivors, adult survivors of CAYA cancers) • Test safety and effectiveness of pharmacological interventions to reduce fatigue in CAYA cancer survivors	

*CAYA*, childhood, adolescent, and young adult; *CNS*, central nervous system; *PROMIS*, Patient-Reported Outcome Measure Information System

Thirteen different measures to assess fatigue were used in the included studies, and twelve studies used a non-standardized measure. The use of 25 different measures makes a comparison of study results difficult. To increase comparability across studies, as well as to measure the quality of care across countries, we highly recommend that researchers and clinicians use the recommended fatigue measures unless they need a more specific measure to answer their research questions. This recommendation is in line with other research that has proposed that the PROMIS fatigue measures (child and adult versions) should be adopted as standard measures of fatigue impact and severity [101].

It would be useful to have a psychometrically sound but very brief fatigue assessment to assess fatigue as a secondary outcome and increase the standardization of fatigue surveillance in survivors (Fig. 1). This would reduce the burden associated with fatigue surveillance and potentially improve clinician adherence to fatigue surveillance recommendations. In this regard, the psychometric properties of the Fatigue Thermometer (a 1-item screening tool) [37] should be tested in diagnostic groups other than brain tumor survivors (Table 3). Other barriers for surveillance of fatigue include not all institutions have a LTFU program or not all survivors have access to a LTFU program, time constraints during the follow-up appointments, providers’ lack of awareness that many CAYA cancer survivors suffer from fatigue, absence of highly effective treatments for fatigue, and previous contradictory evidence about the prevalence of fatigue in CAYA cancer survivors. Additionally, defining clinically meaningful thresholds for fatigue measures would be useful for clinical practice. Health problems such as fatigue, with unspecific and subjective symptoms, are difficult to measure objectively; validated clinically, significant thresholds are important to support health insurance coverage for interventions that remediate fatigue (Table 3).

When no underlying medical condition is identified, the panel recommends referring survivors who endorse fatigue to a specialist in fatigue (Fig. 1). However, not many countries have specialists for fatigue. Healthcare professionals might need to refer survivors to more generic specialists, such as psychologists or physiotherapists. If fatigue specialists are not available, healthcare professionals should counsel survivors about fatigue and interventions available to manage fatigue symptoms. Referral to a more generic specialist should be considered if the interventions are not successful in remediating fatigue, survivors find it difficult to adhere to the interventions, or need more support and guidance. Physical activity and adventure-based interventions should be appropriate for the survivor’s age and physical abilities. Survivors with a higher risk of injury due to chronic health problems or deconditioning should be referred to a physiotherapist for supervised training to assure physical activity are safely implemented. Depending on the maturity and cognitive abilities, relaxation and mindfulness interventions can be useful. Cognitive-behavioral interventions need more resources than the more generic interventions but should be considered for severely fatigued survivors or if other interventions are not successful. Pharmacological approaches (erythropoietin, methylphenidate) should not be routinely used to manage fatigue in CAYA cancer patients and survivors [95] and use of supplements such as *Paullinia cupana*, ginseng products, or CoQ10 is not recommended in survivors of adult cancers because of limited effectiveness and evidence [24]. Supplements should therefore not be routinely used in CAYA cancer survivors.

To date, only a few studies have assessed the effectiveness of interventions for fatigue in CAYA cancer patients or survivors in a reasonably large sample [36, 89–94]. A reason for this might be the lack of information about the mechanisms of fatigue and that, currently, there exists no gold standard intervention for the treatment of fatigue [102]. Other reasons might be that AYA cancer survivors can be difficult to reach due to transitions in care and changes of contact information and usually, studies including AYA cancer survivors have lower response rates than other groups [103]. Furthermore, barriers for screening for CRF might also contribute to the lack of intervention studies. More high-quality randomized controlled trials (RCT) are needed to study the effectiveness of the physical activity, adventure-based, educational, psychosocial, relaxation and mindfulness, cognitive behavioral therapy, and pharmacological interventions in survivors of CAYA cancers (Table 3). These studies’ focus should lie on identifying the most effective interventions for different age groups (pediatric survivors, adolescent survivors, young adult survivors, and adult survivors of CAYA cancers). Evidence from intervention studies in survivors of adult cancers is expected to be transferable to young adult cancer survivors and can be used to guide the design of intervention studies for pediatric and adolescent cancer survivors. Validated fatigue measures, such as the PROMIS Pediatric Fatigue Measure or the PedsQL MFS, should be used to assess CRF in RCTs to reduce bias and increase the comparability of results.

To facilitate dissemination and implementation of this CPG, the evidence and recommendations will be presented directly to clinicians through professional societies and conferences.

Strengths of this CPG are the multidisciplinary and international working group involved in the harmonization process, the evidence-based approach, and the transparency in formulating and grading the recommendations. The international collaboration means a reduction of duplication of effort to develop CPGs and brings together knowledge from different research fields and medical disciplines. A limitation of the recommendations is the gap in the literature regarding the risk of fatigue in survivors over time, and interventions to reduce fatigue in CAYA cancer survivors, as well as the great variability of measures used in the included studies. Research to address these gaps in knowledge should be approached in a systematic, comprehensive manner by sufficiently large single-institution studies, or national and international multi-center collaborative projects.

This surveillance guideline, and the international harmonization initiative that underpins it, aims to improve health outcomes by facilitating more consistent LTFU care for CAYA cancer survivors by improving surveillance, detection, and treatment of fatigue in survivors, as well as promoting strategically planned ongoing research that will inform future guideline updates.

## Electronic supplementary material

ESM 1 (PDF 1853 kb)
