# Supplementary material for: Recommendations for the surveillance of cancer-related fatigue in childhood, adolescent, and young adult cancer survivors: a report from the International Late Effects of Childhood Cancer Guideline Harmonization Group
Source: J Cancer Surviv. 2020 Aug 25;14(6):923–38. doi: 10.1007/s11764-020-00904-9 (PMC7572340; doi:10.1007/s11764-020-00904-9)
Supplement: Supplementary file 1 — (PDF 1853 kb) [file 11764_2020_904_MOESM1_ESM.pdf]

Recommendations for the Surveillance of Cancer-Related Fatigue in Childhood, Adolescent and Young Adult Cancer Survivors: A Report from the International Late Effects of Childhood Cancer Guideline Harmonization Group

Salome Christen+, Katharina Roser+, Renée L. Mulder, Anica Ilic, Hanne C. Lie, Jacqueline J. Loonen, Anneli V. Mellblom, Leontien C.M. Kremer, Melissa M. Hudson, Louis S. Constine, Roderick Skinner, Katrin Scheinemann*, Jordan Gilleland Marchak*, and Gisela Michel*, on behalf of the IGHG psychological late effects group
+ shared first authors; * shared last authors

Corresponding author:
Gisela Michel; gisela.michel@unilu.ch; University of Lucerne, Frohburgstrasse 3, P.O. Box 4466, 6002 Lucerne; +41 41 229 59 55

Journal of Cancer Survivorship

Online Resource 1

Figure S1. Prisma Flow Chart.

Table S2. Summary of the prevalence of cancer-related fatigue, and levels of fatigue reported by the included studies.

Table S3. Individuals involved in the development of the recommendations for surveillance of cancer-related fatigue in childhood, adolescent, and young adult cancer survivors.

Table S4a. Search strategy from February 18th 2016 (Pubmed (Medline), Web of Science, PsycInfo, Scopus).

Table S4b. Search strategy from the Cochrane Childhood Cancer Group searched on March 18th 2019 (Pubmed (Medline)).

Table S4c. Search strategy "Fatigue screening tool" from the Cochrane Childhood Cancer Group searched on March 18th 2019 (Pubmed (Medline)).

Table S5. Inclusion and exclusion criteria.

Table S6. Risk of bias assessment criteria for observational studies developed by Cochrane Childhood Cancer.

Table S7. Criteria for grading and formulating overall conclusions (adapted version of the *Grading of Recommendations Assessment Development and Evaluation* criteria).

Table S8. Criteria for grading the levels of evidence and strength of recommendations (adapted from the *Applying classification of recommendations and level of evidence criteria* of the American Heart Association).

Table S9. Concordances and discordances between existing surveillance recommendations for cancer-related fatigue.

Table S10. Five key issues and corresponding clinical questions (CQ).

Table S11. Evidence tables used for extracting the data from included studies of the surveillance recommendations of cancer-related fatigue in childhood, adolescent and young adult cancer survivors.

Table S12. Evidence summaries and overall conclusions for all clinical questions.

Table S13. List of measures for cancer-related fatigue that have been validated in survivors of childhood, adolescent, and young adult cancers.

Figure S1. Prisma Flow Chart.

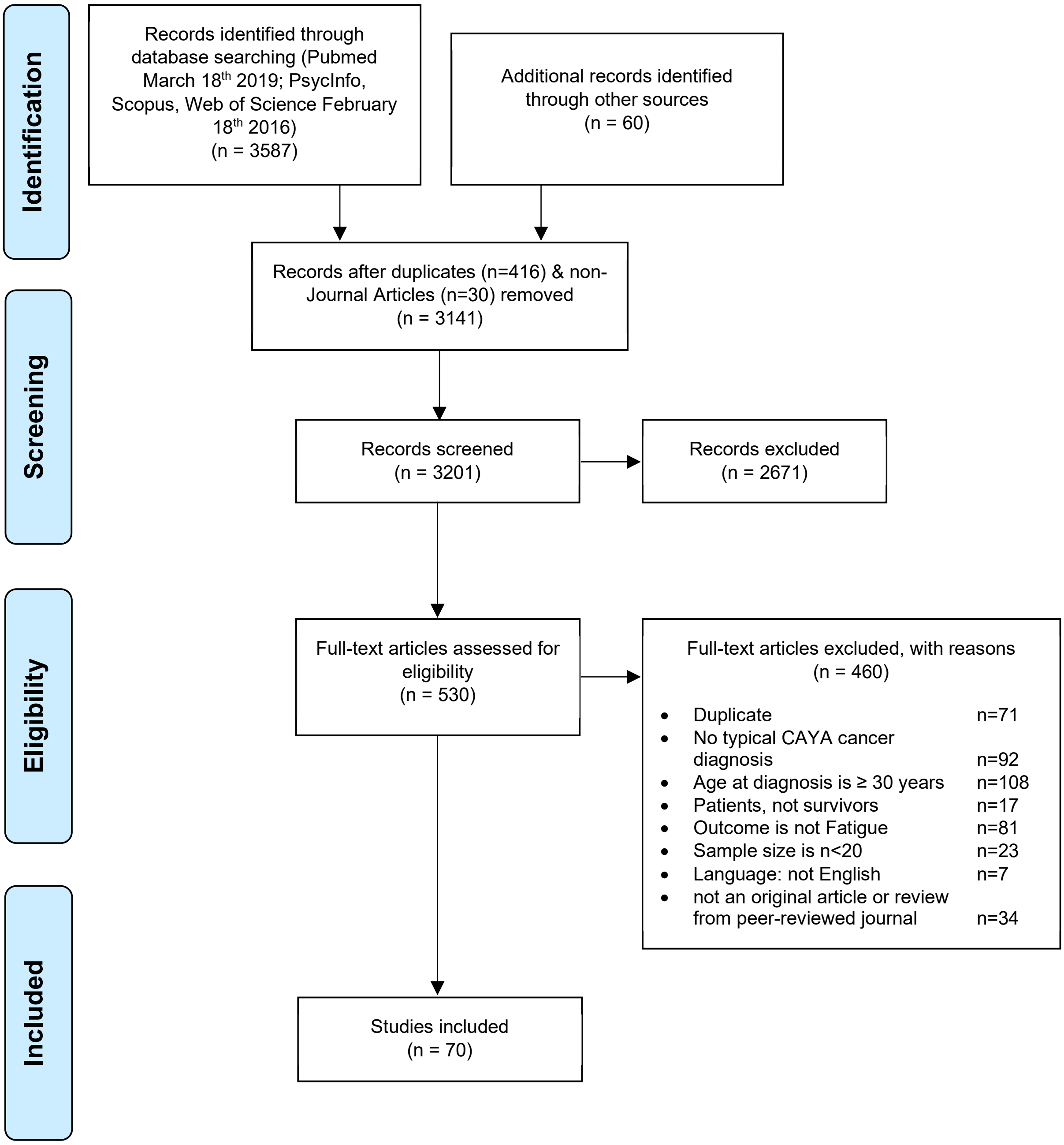

Table S2. Summary of the prevalence of cancer-related fatigue, and levels of fatigue reported by the included studies.

Study and country	Sample size	Prevalence	Prevalence in controls	p-value	Level of fatigue	Level of fatigue in controls	p-value	Assessment tool	Remarks
De Ruiter et al. (2016) [1], The Netherlands	82	··	··	··	63·23 (SD 21·8)	51·76 (SD 21·88)	p=0·01*	CIS↑	Brain tumor survivors; siblings as controls
Blaauwbroek et al. (2009) [2], The Netherlands	46	26·4%	··	··	81·4 (SD 20·1)	47·4 (SD 19·1)	p<0·001*	Prevalence: VAS fatigue↑<br>Levels: CIS↑	Healthy siblings or peers as controls
Barrera et al. 2012 [3][a], Canada	28	··	··	··	18·7 (SD 20·3)	33·9 (SD 26·1)	p<0·001[$]	EORTC-QLQ-30↑	Lower extremity bone tumor survivors; controls are cancer survivors <50 years
Calaminus et al. (2014) [4], Germany	333/725	··	··	··	19·0 (SD 21·7)	7·9 (SD 14·6)	p<0·001*	EORTC-QLQ-30↑	Hodgkin's disease survivors; results for male survivors/ controls from general population
	392/725	··	··	··	26·6 (SD 24·8)	14·0 (SD 20·1)	p<0·001*		Hodgkin's disease survivors; results for female survivors/ controls from general population
Korinthenberg et al. (2011) [5], Germany	28	··	··	··	28·0	28·8	n.s.	EORTC-QLQ-30↑	Deep-seated low-grade glioma survivors; controls from normal population
Sato et al. (2014) [6], Japan	104	··	··	··	26·6 (SD 20·1)	··	··	EORTC-QLQ-30↑	Brain tumor survivors
Sterkenburg et al. (2015) [7], Germany	108	··	··	··	21·0	··	··	EORTC-QLQ-30↑	Craniopharyngioma survivors without HI involvement; median scores
		··	··	··	37·0	··	··		Craniopharyngioma survivors with HI involvement; median scores
Clanton et al. (2011) [8][b], USA	1426	13·8%	··	··	··	··	··	FACIT-Fatigue↓	
Fortmann et al. (2018) [9], United Kingdom	202	26·7%	··	··	15·6 (SD 11·0)	··	··	FACIT-Fatigue↓	This study did not reverse code the FACIT-Fatigue score, so higher scores indicate more fatigue
Kenney et al. (2010) [10], USA	55	16·0%	3·1%	0·067	40·6 (SD 10·4)	45·2 (SD 6·9)	p=0·02*	FACIT-Fatigue↓	Siblings as controls

Table S2 continued

Study and country	Sample size	Prevalence	Prevalence in controls	p-value	Level of fatigue	Level of fatigue in controls	p-value	Assessment tool	Remarks
Mulrooney et al. (2008) [11][b], USA	1897	19·2%	··	··	40·8	42·0	p<0·05*	FACIT-Fatigue↓	Siblings as controls
Rach et al. (2017) [12][b], USA	751	17·0%	··	··	··	··	··	FACIT-Fatigue↓	Hodgkin’s lymphoma survivors
Aksnes et al. (2007) [13], Norway	57/208	14·0%	10·0%	p=0·30	13·2 (SD 3·8)	11·8 (SD 3·9)	p=0·003*	FQ↑	Malignant extremity bone tumor survivors; controls from normal population
	89/208	21·0%	10·0%	n.a.	13·4 (SD 4·8)	11·8 (SD 3·9)	n.a.		Hodgkin’s disease survivors; controls from normal population
	62/208	16·0%	10·0%	n.a.	13·4 (SD 4·7)	11·8 (SD 3·9)	n.a.		Testicular cancer survivors; controls from normal population
Hamre et al. (2013a) [14][c], Norway	290	28·0%	8·0%	OR 4·5 (p<0·001) for having fatigue*	··	··	··	FQ↑	ALL and lymphoma survivors; controls from norm population
Hamre et al. (2013b) [15][c], Norway	232	28·0%	··	··	··	··	··	FQ↑	ALL and lymphoma survivors; controls from norm population
Johannsdottir et al. (2012) [16], Norway	398	11·0%	5·9%	n.a.	··	··	··	FQ↑	Controls: nationally representative sample
Johannsdottir et al. (2017) [17], Norway	124	30·6%	··	··	··	··	··	FQ↑	Lymphoma survivors
Zeller et al. (2014) [18][a,c], Norway	62	··	··	··	20·0	10·5	p<0·001*	FQ↑	ALL and lymphoma survivors; case-control study: Controls were non-fatigued survivors
Ho et al. (2015) [19], Hong Kong, China	200	··	··	··	28·6 (SD 3·7)	22·1 (SD 4·8)	p<0·001*	FS-A↑	Healthy controls

Table S2 continued

Study and country	Sample size	Prevalence	Prevalence in controls	p-value	Level of fatigue	Level of fatigue in controls	p-value	Assessment tool	Remarks
Daniel et al. (2016) [20], USA	154	40·0%	22·0%	p=0·002*	–	–	–	Health Knowledge Inventory	Healthy controls
Langeveld et al. (2003) [21], The Netherlands	416	··	··	··	7·5 (SD 4·3)	8·8 (SD 3·8)	p<0·05$	MFI-20↑: general fatigue	Sex- and age-matched controls
		··	··	··	n.a.	n.a.	p<0·05*	MFI-20↑: mental fatigue	
		··	··	··	n.a.	n.a.	p<0·05$	MFI-20↑: reduced motivation	
Nies et al. (2017) [22], The Netherlands	67	··	··	··	10·0	9·0	p=0·075	MFI-20↑: general fatigue	Differentiated thyroid carcinoma survivors; healthy peers as controls
		··	··	··	9·0	7·0	p=0·012*	MFI-20↑: mental fatigue	
		··	··	··	8·0	6·0	p=0·083	MFI-20↑: physical fatigue	
		··	··	··	8·0	8·0	p=0·613	MFI-20↑: reduced activity	
		··	··	··	6·0	6·0	p=0·879	MFI-20↑: reduced motivation	
		··	··	··	41.0	36.0	p=0.129	MFI-20↑: total fatigue	
Brand et al. (2016) [23], USA	142	29·6%	··	··	70·7 (SD 18.7)	··	··	PedsQL MFS↓	Brain tumor survivors; level of CRF: Mean total fatigue score
Cheung et al. (2017) [24], USA	70	··	··	··	-0·61 (SD 1.20)	0·00 (SD 1·00)	p<0·001*	PedsQL MFS↓	ALL survivors; Fatigue scores were transformed into age-adjusted Z-scores (mean=0, SD=1.0); values in the table are the means for general fatigue
Frederick et al. (2016) [25], USA	268	13·8%	16·0%	0·467	··	··	··	PedsQL MFS↓	Controls: community sample data

Table S2 continued

Study and country	Sample size	Prevalence	Prevalence in controls	p-value	Level of fatigue	Level of fatigue in controls	p-value	Assessment tool	Remarks
Gordijn et al. 2013 [26], The Netherlands	62	··	··	··	78·7	76·8	n.s.	PedsQL MFS↓	ALL survivors; controls: national norm references; values in the table are the self-reported levels of total fatigue
Graef et al. (2016) [27], USA	76	··	··	··	69·2 (SD 20·1)	see remarks	p<0·001*	PedsQL MFS↓	Hematopoietic stem cell transplant survivors; compared to ratings described in another study[28], ratings of total fatigue in this study indicated more fatigue in survivors than in healthy peers (p<0.001).
Mört et al. (2011) [29], Finland	199	··	··	··	83·3	80·6	p<0·01$	PedsQL MFS↓	Matched controls from population registry; values in the table are the self-reported levels of total fatigue
Spathis et al. (2017) [30], United Kingdom	80	85·0%	··	··	44·3 (SD 20·5)	··	··	PedsQL MFS↓	Fatigue level was given for fatigued survivors only, it is not a mean value for all participants
Lowe et al. (2016) [31], USA	104	··	··	··	8·1 (SD 6·0)	··	··	POMS fatigue-inertia↑	
Meeske et al. (2005) [32][d], USA	161	30·0%	··	··	7·2 (SD 6·3)	··	··	Prevalence: R-PFS↑ Level: POMS fatigue-inertia↑	Leukemia survivors
Zeltzer et al. (1997) [33], USA	580	··	··	··	7·9 (SD 5·6)	8·4 (SD 5·8)	p=0·19	POMS↑	ALL survivors; sibling controls
Karimi et al. (2019) [34], USA	144	15·3%	··	··	4·1 (SD 4·0)	··	··	PROMIS V1.0 Pediatric Profile 25↑	
Zebrack et al. (2002) [35], USA	176	··	··	··	7·3	··	··	Quality of Life-Cancer survivors questionnaire↓	0 (severe problem) - 10 (no problem) scale; value in the table is the mean for fatigue
Vannatta et al. (1998) [36], USA	28	··	··	··	0·9	-0·2	p<0·001*	RCP↑	Brain tumor survivors; peer control group

Table S2 continued

Study and country	Sample size	Prevalence	Prevalence in controls	p-value	Level of fatigue	Level of fatigue in controls	p-value	Assessment tool	Remarks
Meeske et al. (2005) [32][a,d], USA	161	··	··	··	63·4	61·3	n.a.	SF-36: Vitality domain↓	Leukemia survivors; general population norms; value in the table is the mean score
Kanellopoulos et al. (2013) [37][c], Norway	285	27·0%	··	··	51·1 (SD 21·6)	60·1 (SD 19·3)	p<0·001*	Prevalence: FQ↑ Levels: SF-36 Vitality domain↓	ALL and lymphoma survivors; controls from norm population
Adams et al. (2004) [38], USA	48	67·0%	··	··	··	··	··	No standardized tool	Hodgkin's disease survivors
Arpaci & Kilicarslan Toruner (2016) [39], Turkey	61	29·7%	··	··	··	··	··	No standardized tool	ALL survivors
Berg et al (2009) [40], USA	25	24·0%	··	··	··	··	··	No standardized tool	
Berg et al. (2013) [41], USA	42	52·0%	··	··	··	··	··	No standardized tool	
Enskär et al. (2007) [42], Sweden	39	67·0%	··	··	··	··	··	No standardized tool	
Geenen et al. (2007) [43], The Netherlands	1284	10·2%	··	··	··	··	··	No standardized tool	
Khan et al. (2014) [44], USA	162	21·6%	··	··	··	··	··	No standardized tool	ALL survivors
Macpherson et al. (2015) [45], USA	103	··	··	··	2·73	··	··	No standardized tool↓	Hodgkin lymphoma survivors; 0 (very much so) – 4 (not at all) scale; value in the table is the mean for "felt tired"
Manley et al. (2012) [46], USA	28	50·0%	··	··	··	··	··	No standardized tool	Craniopharyngioma survivors

Table S2 continued

Study and country	Sample size	Prevalence	Prevalence in controls	p-value	Level of fatigue	Level of fatigue in controls	p-value	Assessment tool	Remarks
McClellan et al. (2013) [47], USA	271	30·0%	··	··	··	··	··	No standardized tool	
Nagai et al. (2012) [48], Japan	81	··	··	··	9·8	11·4	p<0·05$	No standardized tool↑	ALL and AML survivors; healthy controls; 0-3 Likert scale (0=not at all; 3=every day; Total score 0-36)
Yi et al. (2014) [49], Korea	225	25·8%	··	··	··	··	··	No standardized tool	

Abbreviations: ALL=acute lymphoblastic leukemia; AML=acute myeloid leukemia; AYA=adolescent and young adult; CIS=Checklist Individual Strength; CRF=cancer-related fatigue; EORTC-QLQ-30=European Organization for Research and Treatment of Cancer-Quality of Life Questionnaire-30; FACIT-Fatigue= Functional Assessment of Chronic Illness Therapy-Fatigue; FS-A=Fatigue Scale-Adolescent; FQ=Fatigue Questionnaire; GP=general practitioner; HI=hypothalamic involvement; MFI-20=Multidimensional Fatigue Inventory-20; n.a.=not available; n.s.=not statistically significant; SD=standard deviation; OR=odds ratio; PedsQL MFS= Pediatric Quality of Life Inventory Multidimensional Fatigue Scale; POMS=Profile of Mood States; SF-36=Short Form-36; RCP=Revised Class Play; R-PFS=Revised Piper Fatigue Scale; VAS=Visual Analogue Scale

[a] This study was not included for the comparison of CRF levels in survivors and controls.

[b] This study used data from the Childhood Cancer Survivor Study (CCSS)

[c] These articles are from the same study

[d] This study is listed twice because of two different CRF measurements (POMS fatigue-inertia, SF-36 Vitality domain)

↑ higher scores indicate more fatigue

↓ lower scores indicate more fatigue

* Survivors more fatigued than controls

$ Controls more fatigued than survivors

Table S3. Individuals involved in the development of the recommendations for surveillance of cancer-related fatigue in childhood, adolescent, and young adult cancer survivors.

	Name	Discipline / Content expertise	Institution	Geographical location	Role in the guideline development group
**Preparation of surveillance recommendations by group of 14 experts (authors)**					
1	Gisela Michel	Researcher, Psychologist	University of Lucerne	Lucerne, Switzerland	Project chair of the IGHG psychological late effects guidelines[a], CRF WG co-leader[b,c,d,e,f]
2	Jordan Gilleland Marchak	Researcher, Pediatric psychologist	Emory University and the Aflac Cancer & Blood Disorders Center of Children's Healthcare of Atlanta	Atlanta, Georgia, United States	Project chair of the IGHG psychological late effects guidelines[a,b,c,d,e,f]
3	Salome Christen	Researcher, Health Scientist, Physiotherapist	University of Lucerne	Lucerne, Switzerland	Project coordinator of the IGHG psychological late effects guidelines[a], CRF WG member[b,c,d,e,f]
4	Katrin Scheinemann	Pediatric oncologist	Kantonsspital Aarau	Aarau, Switzerland	CRF WG co-leader[b,c,d,e,f]
5	Katharina Roser	Researcher, Epidemiologist	University of Lucerne	Lucerne, Switzerland	CRF WG member[b,c,d,e,f]
6	Anica Ilic	Researcher, Communication Scientist	University of Lucerne	Lucerne, Switzerland	CRF WG member [c,d,f]
7	Hanne C. Lie	Researcher, Psychologist	University of Oslo	Oslo, Norway	CRF WG member [c,d,f]
8	Jacqueline J. Loonen	Pediatric oncologist	Radboud University Medical Center	Nijmegen, The Netherlands	CRF WG member [c,d,f]
9	Anneli V. Mellblom	Researcher, Psychologist	University of Oslo	Oslo, Norway	CRF WG member [c,d,f]
10	Renée L. Mulder	Researcher, Health Scientist, Guidelines methodology	Princess Máxima Center for Pediatric Oncology	Utrecht, The Netherlands	Advisor[b,c,d,e,f]
11	Leontien C. M. Kremer	Pediatrician, Researcher, Epidemiologist, Guidelines methodology	Princess Máxima Center for Pediatric Oncology	Utrecht, The Netherlands	Advisor[b,c,d,e,f]
12	Melissa M. Hudson	Pediatric oncology, Survivorship	St. Jude Children's Research Hospital	Memphis, United States	Advisor[b,d,e,f]
13	Louis S. Constine	Radiation oncologist	University of Rochester Medical Center	Rochester, United States	Advisor[b,d,e,f]
14	Roderick Skinner	Pediatric oncologist	Great North Children's Hospital and Newcastle University Centre for Cancer	Newcastle upon Tyne, UK	Advisor[b,d,e,f]

Table S3 continued

**Discussion of surveillance recommendations in wider group of 23 additional experts**					
1	Adrienne Viola	Public health researcher	The State University of New Jersey	New Brunswick, United States	IGHG psychological late effects guidelines group member[a,d]
2	Charlotte Sleurs	Psychologist, Researcher	KU Leuven	Leuven, Belgium	IGHG psychological late effects guidelines group member[a,d]
3	Christopher Recklitis	Psychologist	Dana-Farber Cancer Institute	Boston, United States	IGHG psychological late effects guidelines group member[a,d]
4	Claire Wakefield	Research psychologist	University of New South Wales and Sydney Children's Hospital	Sydney, Australia	IGHG psychological late effects guidelines group member[a,d]
5	Emma Potter	Clinical Nurse Specialist	The Royal Marsden Hospital	London, UK	IGHG psychological late effects guidelines group member[a,d]
6	Erika Harju	Researcher, Health Scientist, Registered Nurse	University of Lucerne	Lucerne, Switzerland	IGHG psychological late effects guidelines group member[a,d]
7	Fiona Schulte	Researcher, Pediatric psychologist	University of Calgary,	Calgary, Canada	IGHG psychological late effects guidelines group member[a,d]
8	Iris Elens	Child & adolescent psychiatrist	KU Leuven	Leuven, Belgium	IGHG psychological late effects guidelines group member[a,d]
9	Janine Vetsch	Researcher	School of Women's and Children's Health, UNSW Sydney, Australia; University of Applied Sciences, FHS St. Gallen	St. Gallen, Switzerland	IGHG psychological late effects guidelines group member[a,d]
10	Jennifer Lee	Researcher, Psychologist	Emory University	Atlanta, Georgia, United States	IGHG psychological late effects guidelines group member[a,d]
11	Johanna M. C. Blom	Pediatric Neuroscientist, Researcher	University of Modena and Reggio Emilia	Modena, Italy	IGHG psychological late effects guidelines group member[a,d]
12	Joel Khor	Child & adolescent psychiatrist	The Royal Marsden NHS Foundation Trust	London, UK	IGHG psychological late effects guidelines group member[a,d]
13	Jurgen Lemiere	Clinical Psychologist	University Hospital Leuven, KU Leuven	Leuven, Belgium	IGHG psychological late effects guidelines group member[a,d]
14	Katie Recuay (Devine)	Psychologist, Researcher	The State University of New Jersey	New Brunswick, United States	IGHG psychological late effects guidelines group member[a,d]
15	Katja Baust	Researcher, Psychologist	University Hospital Bonn	Bonn, Germany	IGHG psychological late effects guidelines group member[a,d]
16	Lisa M. Ingerski	Pediatric psychologist	Emory University	Atlanta, Georgia, United States	IGHG psychological late effects guidelines group member[a,d]
17	Lori Wiener	Researcher	National Cancer Institute	Bethesda, United States	IGHG psychological late effects guidelines group member[a,d]
18	Luzius Mader	Researcher	Danish Cancer Society Research Center	Copenhagen, Denmark	IGHG psychological late effects guidelines group member[a,d]
19	Morven Brown	Researcher, Health Psychologist	Newcastle University	Newcastle upon Tyne, UK	IGHG psychological late effects guidelines group member[a,d]
20	Nina Kadan-Lottick	Pediatric Oncologist	Yale University	New Haven, United States	IGHG psychological late effects guidelines group member[a,d]
21	Satomi Sato Funaki	Clinical psychologist	National Center for Child Health and Development	Tokyo, Japan	IGHG psychological late effects guidelines group member[a,d]
22	Susanna Waern	Consultant clinical psychologist	The Royal Marsden Hospital	London, UK	IGHG psychological late effects guidelines group member[a,d]
23	Tara M. Brinkman	Researcher, Psychologist	St. Jude Children's Research Hospital	Memphis, TN, United States	IGHG psychological late effects guidelines group member[a,d]

Table S3 continued

**Review of surveillance recommendations by four patient stakeholders**					
1	Clarissa Schilstra	Survivor, Researcher	University of New South Wales	Sydney, Australia	Patient stakeholder,[d]
2	Jaap den Hartogh	Survivor representative/ patient advocate	Dutch Childhood Cancer Parent Organization; CCI Europe	Nieuwegein, The Netherlands	Patient stakeholder,[d]
3	Zuzana Tomášiková	Survivor representative/ patient advocate	Childhood Cancer Switzerland, CCI Europe	Basel, Switzerland	Patient stakeholder,[d]
4	Carina Schneider	Psychologist, survivor representative/ patient advocate	Austrian Childhood Cancer Organization, CCI Europe	Vienna, Austria	Patient stakeholder,[d]

Abbreviations: CCI=Childhood Cancer International; CRF=cancer-related fatigue; WG=working group

[a]The IGHG psychological late effects guidelines group develops surveillance recommendations for the three outcomes “mental health problems”, “psychosocial issues”, and “cancer-related fatigue”. The group consists of two project chairs (Gisela Michel, Jordan Gilleland Marchak), one project coordinator (Salome Christen), five advisors (Renée L. Mulder, Leontien C. M. Kremer, Melissa M. Hudson, Louis S. Constine, Roderick Skinner), and is organized in three working groups (one for each outcome) with each two co-leaders and 6-13 members.

[b]SC, KR, RLM, LCMK, MMH, RS, LSC, KS, JGM and GM contributed to the conception and design of the study.

[c]SC, KR, RLM, AI, HCL, JJL, AVM, LCMK, KS, JGM and GM contributed to the search strategy, data extraction, interpretation of the data, and formulation of the recommendations.

[d]All authors, members of the IGHG psychological late effects guidelines group, and patient stakeholders critically revised the recommendations.

[e]SC drafted, and KR, RLM, LCMK, MMH, RS, LSC, KS, JGM and GM critically revised the report.

[f]All authors approved the final version.

Table S4a. Search strategy from February 18, 2016 (Pubmed (Medline), Web of Science, PsycInfo, Scopus).

**1. Childhood**	infant OR infan* OR newborn OR newborn* OR new-born* OR baby OR baby* OR babies OR neonat* OR perinat* OR postnat* OR child OR child* OR schoolchild* OR schoolchild OR school child OR school child* OR kid OR kids OR toddler* OR adolescent OR adoles* OR teen* OR boy* OR girl* OR minors OR minors* OR underag* OR under ag* OR juvenil* OR youth* OR kindergar* OR puberty OR puber* OR pubescen* OR prepubescen* OR prepuberty* OR pediatrics OR pediatric* OR paediatric* OR peadiatric* OR schools OR nursery school* OR preschool* OR pre school* OR primary school* OR secondary school* OR elementary school* OR elementary school OR high school* OR highschool* OR school age OR schoolage OR school age* OR schoolage* OR infancy OR schools, nursery OR infant, newborn OR young adult	
**2. Cancer**	(((leukemia OR leukemi* OR leukaemi* OR (childhood ALL) OR AML OR lymphoma OR lymphom* OR hodgkin OR hodgkin* OR T-cell OR B-cell OR non-hodgkin OR sarcoma OR sarcom* OR sarcoma, Ewing's OR Ewing* OR osteosarcoma OR osteosarcom* OR wilms tumor OR wilms* OR nephroblastom* OR neuroblastoma OR neuroblastom* OR rhabdomyosarcoma OR rhabdomyosarcom* OR teratoma OR teratom* OR hepatoma OR hepatom* OR hepatoblastoma OR hepatoblastom* OR PNET OR medulloblastoma OR medulloblastom* OR PNET* OR neuroectodermal tumors, primitive OR retinoblastoma OR retinoblastom* OR meningioma OR meningiom* OR glioma OR gliom*) OR (pediatric oncology OR paediatric oncology)) OR (childhood cancer OR childhood tumor OR childhood tumors)) OR (brain tumor* OR brain tumour* OR brain neoplasms OR central nervous system neoplasm OR central nervous system neoplasms OR central nervous system tumor* OR central nervous system tumour* OR brain cancer* OR brain neoplasm* OR intracranial neoplasm*) OR (leukemia lymphocytic acute) OR (leukemia, lymphocytic, acute[mh]) OR cancer OR cancers OR cancer* OR oncology OR oncolog* OR neoplasm OR neoplasms OR neoplasm* OR carcinoma OR carcinom* OR tumor OR tumour OR tumor* OR tumour* OR tumors OR tumours OR malignan* OR malignant OR hematooncological OR hemato oncological OR hemato-oncological OR hematologic neoplasms OR hematolo*	
**3. Survivor**	Survivor OR survivors OR Long-Term Survivors OR Long Term Survivors OR Long-Term Survivor OR Survivor, Long-Term OR Survivors, Long-Term OR survivo* OR survivi*	
**4. Fatigue**	Fatigue OR tiredness	
**Limits:**	English language<br>Humans<br>Published 1990-2016	
**Combined**	**1 AND 2 AND 3 AND 4**	**= 1078 hits**

Table S4b. Search strategy from the Cochrane Childhood Cancer Group searched on March 18, 2019 (Pubmed (Medline)).

**1. Childhood cancer**	((leukemia OR leukemi* OR leukaemi* OR (childhood ALL) OR AML OR lymphoma OR lymphom* OR hodgkin OR hodgkin* OR T-cell OR B-cell OR non-hodgkin OR sarcoma OR sarcom* OR sarcoma, Ewing's OR Ewing* OR osteosarcoma OR osteosarcom* OR wilms tumor OR wilms* OR nephroblastom* OR neuroblastoma OR neuroblastom* OR rhabdomyosarcoma OR rhabdomyosarcom* OR teratoma OR teratom* OR hepatoma OR hepatom* OR hepatoblastoma OR hepatoblastom* OR PNET OR medulloblastoma OR medulloblastom* OR PNET* OR neuroectodermal tumors, primitive OR retinoblastoma OR retinoblastom* OR meningioma OR meningiom* OR glioma OR gliom*) OR (pediatric oncology OR paediatric oncology) OR (childhood cancer OR childhood tumor OR childhood tumors)) OR (brain tumor* OR brain tumour* OR brain neoplasms OR central nervous system neoplasm OR central nervous system neoplasms OR central nervous system tumor* OR central nervous system tumour* OR brain cancer* OR brain neoplasm* OR intracranial neoplasm*) OR testis neoplasm OR neoplasm, testicular OR testicular neoplasm OR testicular neoplasms OR testis cancer OR testicular cancer OR testis tumor OR testicular cancer OR cancer of testis OR testis tumour OR testis neoplasm* OR testis tumour* OR testis tumor* OR (leukemia, lymphocytic, acute[mh])
**2. Survivors**	Survivor OR survivors OR survivor* OR long term survivor OR long term survivors OR long term survivor* OR survivo* OR surviving OR long term survival[tiab] OR survival[mh]
**3. Late effects**	"late effect" OR "late effects" OR "late effect*" OR "late side effect" OR "late side effects" OR "late side effect*" OR "late adverse effect" OR "late adverse effects" OR "late adverse effect*" OR long term effect[tiab] OR long term effect* OR long term adverse effects[mh] OR aftercare OR follow up studie* OR follow up study
**4. Fatigue**	fatigue[mh] OR fatigue OR fatigu* OR tired[tiab] OR tiredness[tiab] OR tired* OR asthenia[mh] OR asthenia OR astheni* OR exhaustion OR exhausted OR exhaust* OR loss of energy[tiab] OR energy loss[tiab] OR loss of vitality OR (vital* AND loss) OR weary[tiab] OR weariness[tiab] OR weakness OR apathy[mh] OR apath* OR lassitude[tiab] OR lethargy[mh] OR letharg* OR sleep OR sleep deprivation OR sleepiness[tiab] OR drowsy[tiab]OR drowsiness[tiab] OR chronic fatigue syndrome OR CFS OR (CF AND syndrome[tiab])
**Limits:**	English language<br>Humans<br>Published 1990-2019
**Combined**	**1 AND (2 OR 3) AND 4** **= 2150 hits**

Table S4c. Search strategy “Fatigue screening tool” from the Cochrane Childhood Cancer Group searched on March 18th 2019 (Pubmed (Medline)).

**1. Childhood cancer**	((leukemia OR leukemi* OR leukaemi* OR (childhood ALL) OR AML OR lymphoma OR lymphom* OR hodgkin OR hodgkin* OR T-cell OR B-cell OR non-hodgkin OR sarcoma OR sarcom* OR sarcoma, Ewing's OR Ewing* OR osteosarcoma OR osteosarcom* OR wilms tumor OR wilms* OR nephroblastom* OR neuroblastoma OR neuroblastom* OR rhabdomyosarcoma OR rhabdomyosarcom* OR teratoma OR teratom* OR hepatoma OR hepatom* OR hepatoblastoma OR hepatoblastom* OR PNET OR medulloblastoma OR medulloblastom* OR PNET* OR neuroectodermal tumors, primitive OR retinoblastoma OR retinoblastom* OR meningioma OR meningiom* OR glioma OR gliom*) OR (pediatric oncology OR paediatric oncology) OR (childhood cancer OR childhood tumor OR childhood tumors)) OR (brain tumor* OR brain tumour* OR brain neoplasms OR central nervous system neoplasm OR central nervous system neoplasms OR central nervous system tumor* OR central nervous system tumour* OR brain cancer* OR brain neoplasm* OR intracranial neoplasm*) OR testis neoplasm OR neoplasm, testicular OR testicular neoplasm OR testicular neoplasms OR testis cancer OR testicular cancer OR testis tumor OR testicular cancer OR cancer of testis OR testis tumour OR testis neoplasm* OR testis tumour* OR testis tumor* OR (leukemia, lymphocytic, acute[mh])
**2. Screening**	(screening[tiab] OR "diagnosis"[Subheading] OR "diagnosis"[All Fields] OR (tool OR tools) AND (diagnostic OR screening OR assessment) OR questionnaire OR test[tiab] OR measure[tiab] OR scale[tiab]
**3. Validation**	validation OR reliability OR validity OR sensitivity OR specificity OR psychometric* OR psychometrics[mh]
**4. Fatigue**	fatigue[mh] OR fatigue OR fatigu* OR tired[tiab] OR tiredness[tiab] OR tired* OR asthenia[mh] OR asthenia OR astheni* OR exhaustion OR exhausted OR exhaust* OR loss of energy[tiab] OR energy loss[tiab] OR loss of vitality OR (vital* AND loss) OR weary[tiab] OR weariness[tiab] OR weakness OR apathy[mh] OR apath* OR lassitude[tiab] OR lethargy[mh] OR letharg* OR sleep OR sleep deprivation OR sleepiness[tiab] OR drowsy[tiab] OR drowsiness[tiab] OR chronic fatigue syndrome OR CFS OR (CF AND syndrome[tiab]))
**Limits:**	English language<br>Humans<br>Published April 12th 2011 – March 18th 2019
**Combined**	**1 AND 2 AND 3 AND 4** **= 359 hits**

Table S5. Inclusion and exclusion criteria.

Inclusion criteria	Exclusion criteria
• Patients:<br>o CAYA cancer survivors: typical CAYA cancer diagnosis (Leukemia, central nervous system tumor, hodgkin- and non-hodgkin-lymphoma, soft tissue sarcoma, neuroblastoma, renal tumor, bone tumor, retinoblastoma, hepatic tumor, testicular cancer, craniopharyngioma)<br>o Study group: ≥75% <30 years at cancer diagnosis (or cancer treatment if age at diagnosis not reported)<br>o "Survivors": ≥50% of study population followed 2 years after cancer diagnosis<br>• Language: English<br>• Published in the last 25 years<br>• Study design: all studies<br>o Sample size: ≥ 20 patients<br>o If possible: Multivariate analyses<br>o Regarding reviews: During screening of abstracts include reviews (and mark them). After reading full text: in case it is a systematic review, then include (and use conclusions for generating evidence tables). In case it is a narrative review, then exclude, but screen reference lists at the end of process (when the draft evidence tables are ready) in order to check if we missed any relevant papers<br><br>**Additional inclusion criteria:**<br>CQ1 & 2 – Who needs surveillance?:<br>• Treatment: Any treatment<br>• Outcome: Risk factors for developing Fatigue (diagnosis, treatment, etc.)<br>• Risk factors: Multivariate analyses<br><br>CQ3 & 4 – At what age or time from exposure should surveillance be performed? At what frequency should surveillance be performed?:<br>• Treatment: Any treatment<br>• Outcome:<br>o latency time to develop Fatigue<br>o risk to develop over time<br><br>CQ5 – What surveillance modality should be used?:<br>• Patients:<br>o "Survivors": ≥50% of study population followed 2 years after cancer diagnosis OR<br>o Patients of CAYA cancer<br>• Outcome: diagnostic tool to diagnose Fatigue in CAYA<br><br>CQ6 to CQ9 – What should be done when abnormalities are found?:<br>• Patients:<br>o "Survivors": ≥50% of study population followed 2 years after cancer diagnosis OR<br>o Patients of CAYA cancer	• Duplicate<br>• No typical CAYA cancer diagnosis (e.g. breast cancer, lung cancer, cervical cancer, etc.; other diseases than CAYA cancer)<br>• Age at diagnosis is ≥ 30 years (in ≥25% of study population)<br>• Patients, not survivors (≥50% of study population followed <2 years after cancer diagnosis): exception: if the paper describes a diagnostic tool or an intervention in CAYA cancer patients<br>• papers' outcome is not Fatigue<br>• Sample size is n<20<br>• Language: not English<br>• Published ≤1990<br>• not an original article or review from peer-reviewed journal

Abbreviations: CQ= clinical question; CAYA= childhood, adolescent, and young adult

Table S6. Risk of bias assessment criteria for observational studies developed by Cochrane Childhood Cancer.

	**Internal validity**
**Study group**	Selection bias<br>Is the study group representative? yes/no/unclear<br>Yes if:<br>• the study group consisted of more than 75% of the original cohort of childhood cancer survivors<br>• *or* it was a random sample with respect to the cancer treatment
**Follow-up**	Attrition bias<br>Is the follow-up adequate? yes/no/unclear<br>Yes if:<br>• the outcome was assessed for more than 75% of the study group
**Outcome**	Detection bias<br>Are the outcome assessors blinded for important determinants related to the outcome? yes/no/unclear<br>Yes if:<br>• the outcome assessors were blinded for important determinants related to the outcome
**Risk estimation**	Confounding<br>Are the analyses adjusted for important confounding factors? yes/no/unclear<br>Yes if:<br>• important prognostic factors (i.e. age, gender, co-treatment, follow-up) were taken adequately into account

Table S7. Criteria for grading and formulating overall conclusions (adapted version of the *Grading of Recommendations Assessment Development and Evaluation* criteria [50]).

Conclusions of evidence	Study quality	Study findings for risk factors	Wording in conclusions
**A**<br>**High level of evidence**	Evidence from well performed and high quality studies or systematic reviews (low risk of bias, direct*, consistent, precise)	If a risk factor is significantly associated with the outcome in **≥95%** of the studies	'There is evidence that…'
**B**<br>**Moderate/ Low level of evidence**	Evidence from studies or systematic reviews with few important limitations	If a risk factor is significantly associated with the outcome in **≥50%** of the studies reporting on this risk factor, and in the remaining studies this association is not significant	'Evidence suggests that…'<br><br>'There is moderate quality evidence …'
		If a risk factor is not significantly associated with the outcome in all studies (at least ≥2 studies)	
**C**<br>**Very low level of evidence**	Evidence from studies with serious flaws (high risk of bias, indirect, inconsistent, imprecise)	If a risk factor is significantly or not significantly associated with the outcome in **1 study**	'Some evidence suggests that…'<br><br>'There is low quality evidence …'
		If a risk factor is significantly associated with the outcome in **<50%** of the studies, while in the remaining studies this association is not significant	
		If a risk factor is significantly (either positively or negatively) associated with the outcome in **>50%** of the studies, while the remaining studies show the opposite association of the risk factor and outcome	
**Conflicting evidence**	N/A	If a risk factor is significantly (both positively and negatively) associated with the outcome in the same number of studies of comparable quality	'There is conflicting evidence…'
**No evidence**	N/A	If no studies reported on a risk factor	'No studies reported on…'

* Direct evidence comes from research that directly compares the interventions in which we are interested when applied to the populations in which we are interested and measures outcomes important to patients. Studies are indirect if there are differences in study population (our population of interest is childhood cancer survivors), interventions, or outcome measures, or if there are indirect comparisons of interventions.

Table S8. Criteria for grading the levels of evidence and strength of recommendations (adapted from the *Applying classification of recommendations and level of evidence criteria* of the American Heart Association [51]).

**Grade of Recommendation**<br><br>**Conclusions of evidence based on GRADE**	**Strong recommendation to do**<br><br>Benefits >>> risk & harms	**Moderate recommendation to do**<br><br>Benefits > or = risk & harms	**Recommendation not to do**<br><br>No benefit / Potentially harm
**High quality of evidence**<br>Consistent evidence from well performed and high quality studies or systematic reviews (low risk of bias, direct, consistent, precise).	Strong recommendation based on high quality evidence	Moderate recommendation based on high quality evidence	Recommendation not to do based on high quality evidence
**Moderate quality of evidence**<br>Evidence from studies or systematic reviews with few important limitations.	Strong recommendation based on moderate quality evidence	Moderate recommendation based on moderate quality evidence	Recommendation not to do based on moderate quality evidence
**Low to very low quality of evidence**<br>Evidence from studies with serious flaws, only expert opinion, or standards of care.	Strong recommendation based on expert opinion	Moderate recommendation based on (very) low quality evidence<br>Diverging expert opinions	Recommendation not to do based on expert opinion
	**Wording in recommendations:**		
	It is recommended …	It is reasonable …	It is not recommended …

Table S9. Concordances and discordances between existing surveillance recommendations for cancer-related fatigue.

	COG [52]	DCOG [53]	UKCCLG [54]	SIGN [55]	Concordant/ discordant
**Who needs surveillance?**					
**At risk**					
All survivors	yes	yes	..	..	Discordant
**High risk**					
Female gender	yes	..	..	..	Discordant
Depression	yes	..	..	..	Discordant
Obesity	yes	..	..	..	Discordant
Central CNS tumor	yes, e.g. cranio-pharyngioma	..	..	..	Discordant
Unemployment	yes	..	..	..	Discordant
Sleep disturbance	yes	..	..	..	Discordant
**Highest risk**					
Pulmonary radiation	yes	..	..	..	Discordant
**At what age or time from exposure should surveillance be performed?**					
At the later outpatient clinic		yes	..	..	Discordant
2 years after end of treatment	yes		..	..	Discordant
**At what frequency should surveillance be performed?**					
Once every 5 years		yes	..	..	Discordant
Yearly	yes		..	..	Discordant
**What surveillance modality should be used?**					
Complete the VVV questionnaire		yes	..	..	Discordant
CIS20R questionnaire for tiredness		yes, if score > 18 on the VVV	..	..	Discordant
Psychosocial assessment	yes		..	..	Discordant
Screen for physical sources of fatigue	yes	yes, if score > 18 on the VVV	..	..	Discordant
**What should be done when abnormalities are found?**					
Individual cognitive therapy		yes	..	..	Discordant
Revalidation program (Recovery and Balance)		yes	..	..	Discordant
Individual physiotherapy		yes	..	..	Discordant

Abbreviations: COG=Children's Oncology Group, DCOG=Dutch Childhood Oncology Group, UKCCLG=United Kingdom Children's Cancer Study Group Late Effects Group, SIGN=Scottish Intercollegiate Guidelines Network, n.a.=not available/not specified, VVV=verkorte vermoeidheidsvragenlijst (shortened fatigue questionnaire), CIS20R=Checklist Individual Strength

Table S10. Five key issues and corresponding clinical questions (CQ).

1. **Who needs surveillance?**

   CQ1: What is the risk and what are risk factors for suffering from Fatigue in childhood, adolescent and young adult cancer (CAYA) survivors?

   CQ2: What is the risk for suffering from Fatigue in CAYA survivors who had received pulmonary radiation vs. no pulmonary radiation?

2. **At what age or time from exposure should surveillance be performed?**

3. **At what frequency should surveillance be performed?**

   CQ3: What is the latency time to develop Fatigue in CAYA survivors?

   CQ4: Does the risk of developing Fatigue change over time in CAYA survivors?

4. **What surveillance modality should be used?**

   CQ5: What is the most valid and reliable diagnostic tool to diagnose Fatigue in CAYA survivors?

5. **What should be done when abnormalities are found?**

   CQ6: What is the effect of individual cognitive behavioral therapy in the treatment of Fatigue in CAYA survivors?

   CQ7: What is the effect of individual physiotherapy in the treatment of Fatigue in CAYA survivors?

   CQ8: What is the effect of a revalidation program in the treatment of Fatigue in CAYA survivors?

   CQ9: What is the effect of any intervention in the treatment of Fatigue in CAYA survivors?

Table S11. Evidence tables used for extracting the data from included studies of the surveillance recommendations of cancer-related fatigue in childhood, adolescent and young adult cancer survivors.

1. What is the **risk and what are risk factors for suffering from Fatigue** in CAYA survivors?				
Ho et al. Psychometric properties of the Chinese version of the fatigue scale-adolescent. 2015				
**Study Design<br>Treatment era<br>Years of follow-up<br>Fatigue measurement**	**Participants**	**Treatment**	**Main outcomes**	**Quality assessment<br>Remarks**
**Study Design:**<br>Cross-sectional study<br><br>**Treatment era:**<br>n.a.<br><br>**Years of follow-up:**<br>62% (n=124) have ≥25 months since treatment completed; n=37 (18.5%) 13-24 months; n=39 (19.5%) 6-12 months.<br><br>**Fatigue measurement:**<br>Fatigue-scale adolescent (FS-A)<br><br>**Country:**<br>Hong Kong, China	**Sample size:**<br>N=200 adolescent cancer survivors (CCS)<br>N=50 adolescent cancer patients (ACP)<br><br>**Diagnoses:**<br>• Leukemia n=91 (45.5%)<br>• Lymphoma n=57 (28.5%)<br>• Brain tumor n=33 (16.5%)<br>• Osteosarcoma n=9 (4.5%)<br>• Kidney tumor n=4 (2.0%)<br>• Germ-cell tumor n=6 (3.0%)<br><br>**Age at diagnosis:**<br>Not available<br><br>**Age at study:**<br>N=200 CCS: 13-14 years: n=48 (24%)<br>15-16 years: n=70 (35%)<br>17-18 years: n=82 (41%)<br><br>N=50 ACP: 13-14 years: n=13 (26%)<br>15-16 years: n=18 (36%)<br>17-18 years: n=19 (38%)<br><br>**Controls:**<br>N=50 healthy controls (age at study): 13-14 years: n=15 (30%)<br>15-16 years: n=18 (36%)<br>17-18 years: n=17 (34%)	ACS:<br>• Surgery n=23 (11.5%)<br>• Chemotherapy n=90 (45%)<br>• Bone Marrow Transplant n=22 (11%)<br>• Mixed:<br>○ Chemo & radio n=12 (6%)<br>○ Surgery & chemo n=19 (9.5%)<br>○ Chemo & bone marrow transplantation n=23 (11.5%)<br>○ Radio & surgery n=11 (5.5%)<br><br>ACP:<br>• Surgery n=5 (10%)<br>• Chemotherapy n=22 (44%)<br>• Bone Marrow Transplant n=5 (10%)<br>• Mixed:<br>○ Chemo & radio n=3 (6%)<br>○ Surgery & chemo n=5 (10%)<br>○ Chemo & bone marrow transplantation n=7 (14%)<br>○ Radio & surgery n=3 (6%)	**Risk:**<br>CCS: mean level of fatigue 28.6 (SD 3.7).<br>ACP: mean level of fatigue 31.3 (SD 5.2)<br>Healthy controls: mean level of fatigue 22.1 (SD 4.8; p<0.001 compared to ACS)<br><br>**Risk factors:**<br>We do not extract risk factors, as this study did not perform a multivariable analysis.	Selection bias: 0<br>Convenience sample of 200 survivors.<br>Attrition bias: 1<br>All answered the fatigue questionnaire.<br>Detection bias: 0<br>Questionnaire survey, no blinding possible.<br>Confounding: 0<br>Multivariable analysis were not used.<br><br>**Total quality: 1/4**

Table S11 continued

1. What is the **risk and what are risk factors for suffering from Fatigue** in CAYA survivors?				
Macpherson et al. Exercise and Fatigue in Adolescent and Young Adult Survivors of Hodgkin Lymphoma: A Report from the Children's Oncology Group. 2015				
**Study Design<br>Treatment era<br>Years of follow-up<br>Fatigue measurement**	**Participants**	**Treatment**	**Main outcomes**	**Quality assessment<br>Remarks**
**Study Design:**<br>Retrospective cohort study with data from a RCT<br><br>**Treatment era:**<br>Not available<br><br>**Years of follow-up:**<br>End of therapy, 12 and 36 months post-therapy measurements.<br><br>**Fatigue measurement:**<br>No standardized measurement<br><br>**Country:**<br>USA	**Sample size:**<br>N=103<br><br>**Diagnoses:**<br>• Hodgkin Lymphoma<br><br>**Age at diagnosis:**<br>Mean age at dx: 15.46 years (13-21 years)<br><br>**Age at study:**<br>Not available<br><br>**Controls:**<br>No controls.	Protocol treatment arm:<br><br>Rapid early responders:<br><br>**Rapid early responders:**<br>• ABVE-PC x 4, <CR, IFRT n=47 (45.6%)<br>• ABVE-PC x 4, CR, IFRT n=15 (14.6%)<br>• ABVE-PC x 4, CR, NO IFRT n=26 (25.2%)<br><br>**Slow early responders:**<br>• ABVE-PC x 4 + IFRT + DECA x 2 n= 10 (9.7%)<br>• ABVE-PC x 4 + IFRT n=5 (4.9%)	**Risk:**<br>"Amount of […] fatigue improved from end of therapy to 36 months post-therapy, although not significantly.<br>Items (Scale 0 "very much so" to 4 "not at all") and means 36 months post-therapy:<br>"felt tired" n=94 mean 2.73 (SD 1.18)<br>"had trouble finishing tasks because tired quickly" n=93 mean 3.46 (SD 0.88)<br>"needed to sleep during the day" n=94 mean 3.25 (SD 0.96)<br>"frustrated by being too tired to do things he/she wanted to do" n=93 mean 3.54 (SD 0.90)<br>"needed to limit social activities because of fatigue" n=94 mean 3.68 (SD 0.79)<br><br>**Risk factors from generalized estimation equation, and adjusting for sex, age at diagnosis, stage at diagnosis, and protocol treatment arm:**<br>• "[…] amount of exercise was not predictive of fatigue at end of therapy or at 12 or 36 months post-therapy (p>0.05)."	Selection bias: 0<br>Secondary analysis of data collected as a randomized controlled trial. There's no information on how the randomization was done. One inclusion criterion is "completed a self-report survey at end of treatment, 12 and 36 months" → then it's rather not representative<br>Attrition bias: 1<br>N=93/103 responded fatigue questions at 36 months → 90.3%<br>Detection bias: 0<br>Questionnaire survey, no blinding possible.<br>Confounding: 1<br>Multivariable logistic regression was used to evaluate association with exercise.<br><br>**Total quality 2/4**

Table S11 continued

1. What is the **risk and what are risk factors for suffering from Fatigue** in CAYA survivors?				
Daniel et al. Relationship between sleep problems and psychological outcomes in adolescent and young adult cancer survivors and controls. 2016				
**Study Design**<br>**Treatment era**<br>**Years of follow-up**<br>**Fatigue measurement**	**Participants**	**Treatment**	**Main outcomes**	**Quality assessment**<br>**Remarks**
**Study Design:**<br>Cohort study, convenience sample?<br>**Treatment era:**<br>n.a.<br>**Years of follow-up:**<br>on average 12.29 years since dx (range 4-23 years)<br>**Fatigue measurement:**<br>Health Knowledge Inventory, one question about fatigue<br>**Country:**<br>USA	**Sample size:**<br>N=154 survivors<br>**Diagnoses:**<br>• Leukemia n=68 (44.8%)<br>• Lymphoma n=32 (20.8%)<br>• Solid tumor n=53 (34.4%)<br>**Age at diagnosis:**<br>≤18 years<br>**Age at study:**<br>Mean age 20.08 years (SD 3.17)<br>**Controls:**<br>N=170 healthy AYA controls recruited at preventive or acute primary care appointments.<br>Mean age at study 21.08 years (SD 3.43) p=0.007	**Treatment intensity:**<br>• Least n=5 (4%)<br>• Moderately n=72 (44%)<br>• Very n=57 (36%)<br>• Most intense n=26 (16%)	**Risk:**<br>40% of survivors reported fatigue problems, compared to 22% of controls.<br>When adjusted for age and income, survivors reported significantly more fatigue compared to controls (OR=2.47, p=0.002).<br>**Risk factors:**<br>We do not extract risk factors, as this study did not perform a multivariable analysis.	Selection bias: 0<br>Unclear how large original cohort was.<br>Attrition bias: 0<br>Unclear whether there was missing data or how many participants responded to T1 and T2.<br>Detection bias: 0<br>Questionnaire survey, no blinding possible.<br>Confounding: 1<br>Adjusted for age & income.<br>**Total quality: 1/4**

Table S11 continued

1. What is the **risk and what are risk factors for suffering from Fatigue** in CAYA survivors?				
Barrera et al. Health related quality of life in adolescent and young adult survivors of lower extremity. 2012				
**Study Design**<br>**Treatment era**<br>**Years of follow-up**<br>**Fatigue measurement**	**Participants**	**Treatment**	**Main outcomes**	**Quality assessment**<br>**Remarks**
**Study Design:**<br>Cross-sectional study (Questionnaire survey)<br>**Treatment era:**<br>N/ A<br>**Years of follow-up:**<br>**N/ A**<br>**Fatigue measurement:**<br>**EORTC-QLQ-30**<br>**Country:**<br>Canada	**Sample size:**<br>n = 28<br>**Diagnoses:**<br>• Lower extremity bone tumors:<br>○ Osteogenic sarcoma n=23 (82.1%)<br>○ Ewing's sarcoma n=5 (17.9%)<br>**Age at diagnosis:**<br>6 – 16 years. Mean age 11.6 years<br>**Age at study:**<br>18 – 32 years. Mean age at study 25.1 years.<br>**Controls:**<br>No controls<br>Reference scores for the EORTC-QLQ-C30 were obtained from Scott et al. (2008. EORTC QLQ-C30 reference values.) and represent average scores for cancer survivors under the age of 50.	Limb salvage (LS) n=19:<br>• Allograft fusion n=15 (53.6%)<br>• Endoprosthesis n=4 (14.3%)<br>Amputation (AMP) n=9:<br>• Van Nes rotationsplasty n=6 (21.4%)<br>• Amputation n=3 (10.7%)	**Risk:**<br>EORTC-QLQ-C30 Fatigue subscale: sample mean 18.65 (SD 20.30). reference score mean 33.9 (SD 26.1). → sign. less fatigue (p<0.001) in survivors than reference population.<br>LS reported poorer HRQOL than AMP participants for […] fatigue (LS mean 22.81 (SD 18.69), AMP mean 9.88 (SD 21.83); p=0.033).<br>Female survivors reported significantly more symptoms of Fatigue than male survivors (female: 26.19 (SD 22.05) vs. male: 11.11 (SD 15.71); p=0.047)<br>Older survivors (≥26 years) reported more symptoms of Fatigue than younger survivors (≥26 years: 23.93 (SD 21.20) vs. ≤25 years: 14.07 (SD 19.00). However, this difference did not reach statistical significance (p=0.206).<br>**Risk factors:**<br>We do not extract risk factors, as this study did not perform a multivariable analysis.	Selection bias: 0<br>sample was identified primarily from the registry (POGONIS) of the Pediatric Oncology Group of<br>Ontario – 70 survivors were eligible, 28 participated → 28/70=40%<br>Attrition bias: 1<br>28/28 answered the EORTC-QLQ-30 → 100%<br>Detection bias: 0<br>Questionnaire survey, no blinding possible.<br>Confounding: 0<br>No multivariate analyses.<br>**Total quality 1/ 4**

Table S11 continued

1. What is the **risk and what are risk factors for suffering from Fatigue** in CAYA survivors?				
De Ruiter et al. Psychosocial profile of pediatric brain tumor survivors with neurocognitive complaints. 2016				
**Study Design<br>Treatment era<br>Years of follow-up<br>Fatigue measurement**	**Participants**	**Treatment**	**Main outcomes**	**Quality assessment<br>Remarks**
**Study Design:**<br>part of the PRISMA study, a randomized placebo-controlled double-blind trial to investigate whether neurofeedback can improve neurocognitive functioning in PBTS<br><br>**Treatment era:**<br>Unclear.<br><br>**Years of follow-up:**<br>Mean: 6.98 (SD 3.57)<br><br>**Fatigue measurement:**<br>CIS (checklist individual strength)<br><br>**Country:**<br>The Netherlands	**Sample size:**<br>N=82 participants<br><br>**Diagnoses:**<br>Brain tumors:<br>• High grade:<br>○ Medulloblastoma n=12<br>○ Supratentorial PNET n=8<br>○ Ependymoma n=5<br>○ Astrocytoma gr III n=5<br>○ Germ cell tumor n=4<br>• Low grade:<br>○ Low grad glioma n=35<br>○ Craniopharyngioma n=7<br>○ Plexus papilloma n=6<br><br>**Age at diagnosis:**<br>Mean: 6.87 (SD 3.77)<br><br>**Age at study:**<br>Mean: 13.85 (SD 3.15)<br><br>**Controls:**<br>N=43 siblings in the age range 8-18 years as control group for the fatigue outcome measure	• Radiotherapy n= 34 (42%)<br>• Chemotherapy n=35 (43%)<br>• Surgery n=72 (88%) (N=37 had surgery only)<br>• Other n=2 (2%)<br>• Biopsy only n=1<br>• CSF pressure relief only n=1	**Risk**:<br>PBTS reported more concentration problems than the sibling control group (p<0.01, medium effect size). A trend toward decreased physical activity in PBTS compared to the sibling control group was found as well as a trend toward a higher total scale compared to the siblings (p<0.05, medium effect sizes), indicating more fatigue related problems. The PBTS did not differ from the siblings on subjective fatigue and motivation problems.<br>Survivors had a higher total score of Fatigue (63.23 (SD 21.80) vs. controls: 51.76 (SD 21.88), p=0.010) and reported more concentration problems (subscale of the CIS) (19.09 (SD 7.78) vs. controls: 14.45 (SD 7.19), p=0.003)<br><br>**Risk factors**:<br>No risk factor analyses performed.	Selection bias: 0<br>N=249 eligible, 82 participated → 33%<br>Attrition bias: 1<br>100% of survivors and 40/43=93% of siblings answered the CIS<br>Detection bias: 0<br>Questionnaire survey, no blinding possible.<br>Confounding: 0<br>Only univariate analyses.<br><br>**Total quality: 1/4**<br><br>As fatigue was only one of many parameters in a specifiy disease group results expected

Table S11 continued

1. What is the **risk and what are risk factors for suffering from Fatigue** in CAYA survivors?				
Berg et al. Participation and Self-Management Strategies of Young Adult Childhood Cancer Survivors. 2013				
**Study Design**<br>**Treatment era**<br>**Years of follow-up**<br>**Fatigue measurement**	**Participants**	**Treatment**	**Main outcomes**	**Quality assessment**<br>**Remarks**
**Study Design:**<br>descriptive study using a survey approach (cross-sectional study)<br>**Treatment era:**<br>N/A<br>**Years of follow-up:**<br>8.9 ± 4.9 years (range: 3–20)<br>**Fatigue measurement:**<br>Multiple sources for survey, no standardized Fatigue instrument<br>**Country:**<br>USA	**Sample size:**<br>N=42<br>**Diagnoses:**<br>• Leukemia n=16 (38%)<br>• CNS n=7 (17%)<br>• Lymphoma n=5 (12%)<br>• Hodgkin's lymphoma n= 4 (9.5%)<br>• Wilm's tumor n=4 (9.5%)<br>• Sarcoma n=3 (7%)<br>• Bone n=3 (7%)<br>**Age at diagnosis:**<br>9.8 ± 5.4 years (range: 1–17)<br>**Age at study:**<br>20.5 ± 1.8 years<br>**Controls:**<br>No	• Chemotherapy n=12 (28%)<br>• Radiation n=2 (5%)<br>• Chemotherapy/surgery n=6 (14%)<br>• Radiation/surgery n=3 (7%)<br>• Chemotherapy/radiation n=5 (12%)<br>• Chemotherapy/surgery/ radiation n=14 (33%)	**Risk:**<br>Eighty-eight percent (n=37) of the 42 responders struggled with at least one of the six late effects (memory, body image, fatigue, cognition, pain, depression).<br><br>22 survivors (52%) reported fatigue, and 8 (36%) reported their fatigue was severe enough to limit work activities.<br>**Risk factors:**<br>Not investigated.	Selection bias: 0<br>180 eligible, n=42 participants<br>42/180=23%<br>Attrition bias: 1<br>42/42 answered late effects question<br>Detection bias: 0<br>Blinding not possible.<br>Confounding: 0<br>No multivariate analyses<br>**Total quality 1/4**

Table S11 continued

1. What is the **risk and what are risk factors for suffering from Fatigue** in CAYA survivors?				
Hamre et al. High Prevalence of Chronic Fatigue in Adult Long-Term Survivors of Acute Lymphoblastic Leukemia and Lymphoma during Childhood and Adolescence. 2013a				
**Study Design<br>Treatment era<br>Years of follow-up<br>Fatigue measurement**	**Participants**	**Treatment**	**Main outcomes**	**Quality assessment<br>Remarks**
**Study Design:**<br>Cross-sectional study including mailed questionnaire and 2-day outpatient examination<br><br>**Treatment era:**<br>Diagnosed between 1970 and 2000<br><br>**Years of follow-up:**<br>Survival for >=5 years, median observation time of 21.1 years (range: 6.9 – 39.4 years)<br><br>**Fatigue measurement:**<br>**11-item Chalder Fatigue Questionnaire (FQ)**<br><br>**Country:**<br>Norway	**Sample size:**<br>290 survivors and 1405 controls<br><br>**Diagnoses:**<br>Hodgkin lymphoma (HL), non-Hodgkin lymphoma (NHL), acute lymphoblastic leukemia (ALL)<br><br>**Age at diagnosis:**<br>Median age at diagnosis 9.5 years (range: 0.3 – 18.4 years)<br><br>**Age at study:**<br>Median age at study 29.6 years (18.3 – 54.5 years)<br><br>**Controls:**<br>Persons representative of the entire Norwegian population, median age at study 34.0 years (range: 19.0 – 50.0 years)	ALL: predominantly based on chemotherapy alone<br><br>Lymphoma: included in most cases a combination of chemotherapy and radiotherapy, with large-field radiotherapy applied to patients with HL in the 1970s<br><br>Details of the therapy are described elsewhere:<br><br>• Moe PJ, Seip M, Finne PH. Intermediate dose methotrexate (IDM) in childhood acute lymphocytic leukemia in Norway. Preliminary results of a national treatment program. Acta Paediatr Scand. 1981;70(1):73–9.<br>• Gustafsson G, Schmiegelow K, Forestier E, et al. Improving outcome through two decades in childhood ALL in the Nordic countries: the impact of high-dose methotrexate in the reduction of CNS irradiation. Nordic Society of Pediatric Haematology and Oncology (NOPHO). Leukemia. 2000; 14(12):2267–75.<br>• Hamre H, Kiserud CE, Ruud E, et al. Gonadal function and parenthood 20 years after treatment for childhood lymphoma: a cross-sectional study. Pediatr Blood Cancer. 2012; 59(2):271–7.	**Risk:** 28% of survivors had CF, 8% of controls had CF (p<0.001)<br>OR for having CF: adjusted OR=4.5 (3.1-6.4), p<0.001 (adjusted for age at study and sex)<br>Risk highest among HL survivors (adjusted OR=5.9 (3.6-9.7), p<0.001), followed by NHL survivors (adjusted OR=4.4 (2.2-9.0), p<0.001) and ALL survivors (adjusted OR=3.6 (2.3-5.7), p<0.001)<br><br>**Risk factors for chronic fatigue from multivariable logistic regression:**<br>Whole sample of survivors (n=279). (partnership, education, BMI were n.s. in the univariable model and not included in the multivariable model)<br>• NHL (vs. ALL): OR=1.5 (95% CI: 0.6-3.4), p=0.4<br>• HL (vs ALL): OR=1.7 (0.8-3.5), p=0.2<br>• Age at survey: OR=1.05 (1.0-1.1), p=0.1<br>• Treatment 1970-1985 (vs. Treatment after 1985): OR=0.8 (0.3-2.1), p=0.7<br>• Female (vs. Male): OR=0.8 (0.46-1.5), p=0.6<br>• Present hypothyroidism (vs. Thyroid status normal): OR=1.4 (0.7-3.0), p=0.4<br>• **HADS (Hospital Anxiety and Depression Scale) total score: OR=1.15 (1.1-1.2), p<0.001**<br><br>Sub-analysis ALL survivors (n=148), multivariate (relapse, anthracyclines, radiotherapy, heart function and lung function were were n.s. in the univariable model and not included in the multivariable model):<br>• **Age at survey: OR=1.1 (1.0-1.2), p=0.01**<br>• Treatment 1970-1985 (vs. Treatment after 1985): OR=0.6 (0.2-2.1), p=0.4<br>• Female (vs. Male): OR=0.9 (0.4-2.1), p=0.8<br><br>Sub-analysis HL and NHL survivors (n=131), multivariable (relapse, disease stage, anthracyclines, radiotherapy, heart function and lung function were were n.s. in the univariable model and not included in the multivariable model):<br>• Age at survey: OR=1.0 (0.9-1.1), p=0.5<br>• Treatment after 1985 (vs. Treatment 1970-1985): OR=0.6 (0.2-2.3), p=0.5<br>• Female (vs. Male): OR=0.9 (0.4-2.0), p=0.9<br>• B-symptoms Yes (vs. No): OR=2.5 (1.0-6.2), p=0.05<br>• B-symptoms Unknown (vs. No): OR=1.0 (0.3-2.7), p=0.9	Selection bias: 0<br>Survivors: response rate 65% → no<br>Controls: persons representative of the entire Norwegian population → yes<br>Attrition bias: 1<br>Outcome data for 96.2% of survivors → yes<br>Detection bias: 0<br>Assessors were not blinded → no<br>Confounding: 1<br>Adjusted OR and multivariate analyses → yes<br><br>**Total quality 2/4**<br><br>Abbreviations:<br>CF: chronic fatigue

Table S11 continued

1. What is the **risk and what are risk factors for suffering from Fatigue** in CAYA survivors?				
Clanton et al. Fatigue, Vitality, Sleep, and Neurocognitive Functioning in Adult Survivors of Childhood Cancer. A Report from the Childhood Cancer Survivor Study. 2011				
**Study Design<br>Treatment era<br>Years of follow-up<br>Fatigue measurement**	**Participants**	**Treatment**	**Main outcomes**	**Quality assessment<br>Remarks**
**Study Design:**<br>Childhood Cancer Survivor Study (CCSS), retrospective cohort study<br><br>**Treatment era:**<br>Treated between 1970 and 1986<br><br>**Years of follow-up:**<br>Survival for >=5 years, mean time since diagnosis 24.0 years (SD=4.7 years, range: 16.2 – 34.3 years)<br><br>**Fatigue measurement:**<br>Fatigue subscale of the Functional Assessment of Chronic Illness Therapy-Fatigue (FACIT-Fatigue)<br><br>**Country:**<br>USA	**Sample size:**<br>1426 survivors and 384 sibling controls<br><br>**Diagnoses:**<br>Leukemia 14.0%<br>CNS tumor 15.0%<br>Hodgkin lymphoma (HL) 53.9%<br>Other cancer 17.1%<br>*Survivors of HL were oversampled to represent a majority of the cohort, given the increased rates of fatigue reported in this group*<br><br>**Age at diagnosis:**<br>Mean age at diagnosis 11.9 years (SD=5.6 years, range: 0 – 21 years)<br><br>**Age at study:**<br>Mean age at study 35.9 years (SD=7.5 years, range: 19.2 – 53.4 years)<br><br>**Controls:**<br>Randomly selected sibling controls	**Chemotherapy treatment**<br>Alkylators 50.6%<br>Anthracycline 28.5%<br>Antimetabolite (IV) 18.6%<br>Antimetabolite (IT) 55.8%<br>Corticosteroids 38.0%<br>Epipodophyllotoxin 3.2%<br><br>**CRT**<br>No CRT 21.7%<br>CRT <20 Gy 54.6%<br>CRT ≥20 Gy 14.1%	**Risk:**<br>Cutoff score of ≥ highest 10% of siblings was used.<br><br>197 of 1426 survivors (13.8%) fatigued<br><br>**Risk factors:**<br>N/A	Selection bias: 0<br>Survivors of HL were oversampled to represent a majority of the cohort, given the increased rates of fatigue reported in this group; response rates not reported → unclear<br>Attrition bias: 1<br>Fatigue assessed for the whole study sample → yes<br>Detection bias: 0<br>Assessors not blinded → no<br>Confounding: 0<br>Prevalence of fatigue not adjusted → no<br><br>**Total quality 1/4**<br><br>Abbreviations:<br><br>CRT: cranial radiation therapy<br><br>Gy: grays

Table S11 continued

1. What is the **risk and what are risk factors for suffering from Fatigue** in CAYA survivors?				
Berg et al. Late Effects of Childhood Cancer, Participation, and Quality of Life of Adolescents. 2009				
**Study Design<br>Treatment era<br>Years of follow-up<br>Fatigue measurement**	**Participants**	**Treatment**	**Main outcomes**	**Quality assessment<br>Remarks**
**Study Design:**<br>90-minute interview with the adolescents<br>**Treatment era:**<br>N/A<br>**Years of follow-up:**<br>Survivors >=2 years post-cancer intervention; mean time since diagnosis 7.2 years (SD=3.3 years)<br>**Fatigue measurement:**<br>Data gathered from a 90-minute interview with the adolescents; fatigue among late effects reported<br>**Country:**<br>USA	**Sample size:**<br>25 survivors<br>**Diagnoses:**<br>Acute lymphoblastic leukemia 56%<br>Wilms tumor 16%<br>Non-Hodgkin lymphoma 8%<br>Hodgkin lymphoma 4%<br>Neuroblastoma 4%<br>Ewing sarcoma 4%<br>Renal sarcoma 4%<br>Rhabdomyosarcoma 4%<br>**Age at diagnosis:**<br>Mean age at diagnosis 5.2 years (SD=3.6 years)<br>**Age at study:**<br>Mean age at study 14.0 years (SD=2.2 years)<br>**Controls:**<br>No controls	Chemotherapy 44%<br>Chemotherapy and radiation 20%<br>Chemotherapy, radiation, and surgery 36%	**Risk:**<br>6 of 25 (24%) survivors reported fatigue (fatigue among late effects reported)<br>**Risk factors:**<br>N/A	Selection bias: 0<br>Convenience sample of survivors, contacted sample included the first 26 consecutive clinic patients who met the inclusion criteria; 96.2% of contacted survivors participated → no<br>Attrition bias: 1<br>Outcome from all 25 participating survivors → yes<br>Detection bias: 0<br>Assessors not blinded → no<br>Confounding: 0<br>Prevalence of fatigue not adjusted → no<br>**Total quality 1/4**

Table S11 continued

1. What is the **risk and what are risk factors for suffering from Fatigue** in CAYA survivors?				
Korinthenberg et al. Assessing Quality of Life in Long-Term Survivors after [125]I Brachytherapy for Low-Grade Glioma in Childhood. 2011				
**Study Design**<br>**Treatment era**<br>**Years of follow-up**<br>**Fatigue measurement**	**Participants**	**Treatment**	**Main outcomes**	**Quality assessment**<br>**Remarks**
**Study Design:**<br>Cross-sectional study (questionnaire survey)<br><br>**Treatment era:**<br>1984-2003<br><br>**Years of follow-up:**<br>Median: 134 months from ^{125}I brachytherapy (range 29-293 months)<br><br>**Fatigue measurement:**<br>EORTC QLQ-30 (only for survivors >18 years)<br><br>**Country:**<br>Germany	**Sample size:**<br>N=51 (53.7% response rate)<br><br>**Diagnoses:**<br>Deep-seated low-grade gliomas:<br>• Pilocytic astrocytoma WHO I (n=34)<br>• fibrillary astrocytoma WHO II (n=7)<br>• unspecified astrocytoma (n=3)<br>• oligodendroglioma WHO II (n=3)<br>• oligo-astrocytoma WHO II (n=1)<br><br>**Age at diagnosis:**<br>Median age of 8.3 years (range 1.5 – 17.7. years) at the time of radiosurgery<br><br>**Age at study:**<br>N=29 >18 years<br>N= 18 11-17 years<br>N=4 <11 years<br><br>**Controls:**	Stereotactically-inserted temporary ^{125}I seeds<br><br>14 patients underwent repeated ^{125}I radiosurgery due to lack of response or secondary progression<br><br>14 patients had undergone treatment other than ^{125}I radiosurgery in the later course (9 surgery only, 2 external beam radiotherapy, 1 chemotherapy, 2 combination of surgery and radiotherapy)	**Risk:**<br>EORTC QLQ-30 mean Fatigue score in n=28 survivors (>18 years): ~28%<br>In the normal population: ~28.8%<br>→ Survivors score a bit lower, but not statistically significant.<br><br>**Risk factors:**<br>We do not extract risk factors, as this study did not perform a multivariable analysis.	Selection bias: 0<br>Original cohort n=156 CCS. Only 95 (60.9%) were included for the study.<br>Attrition bias: 0<br>The response rate of the whole study group was 53.7%.<br>Detection bias: 0<br>Questionnaire survey, no blinding possible.<br>Confounding: 0<br>Only descriptive statistics and correlations used.<br><br>**Total quality: 0/4**

Table S11 continued

1. What is the **risk and what are risk factors for suffering from Fatigue** in CAYA survivors?				
Geenen et al. Medical Assessment of Adverse Health Outcomes in Long-term survivors of childhood cancer. 2007				
**Study Design<br>Treatment era<br>Years of follow-up<br>Fatigue measurement**	**Participants**	**Treatment**	**Main outcomes**	**Quality assessment<br>Remarks**
**Study Design:**<br>Retrospective cohort study<br>**Treatment era:**<br>1966-1996<br>**Years of follow-up:**<br>Median follow-up time of 17.0 years (interquartile range 11.6-23.3 years)<br>**Fatigue measurement:**<br>No specific Fatigue measurement, but the "Common Terminology Criteria for Adverse Events version 3.0"<br>**Country:**<br>The Netherlands	**Sample size:**<br>N=1284 (response rate: 94.3%)<br>**Diagnoses:**<br>• Leukemia n=335 (24.6%)<br>• Lymphoma 259 (19.0)<br>• Kidney/Wilms tumor 189 (13.9)<br>• Brain/CNS tumor 107 (7.9)<br>• Bone tumor 116 (8.5)<br>• Soft tissue sarcoma 151 (11.1)<br>• Neuroblastoma 85 (6.2)<br>• Other 120 (8.8)<br>**Age at diagnosis:**<br>0-4 years: 43.8% (n=596)<br>5-9 years: 27.8% (n=378)<br>10-14 years: 22.7% (n=309)<br>15-18 years: 5.8% (n=79)<br>**Age at study:**<br>Median age at end of follow-up: 24.4 years (n=1194 (88%) younger than 35 years).<br>**Controls:**	• Chemotherapy only (with/without surgery): n=652 (47.9%)<br>• Radiotherapy only (with/without surgery): n=93 (6.8%)<br>• Chemotherapy + radiotherapy first treatment, no recurrence: n=334 (24.5%)<br>• Chemotherapy + radiotherapy first treatment including recurrence treatment: n=180 (13.2%)<br>• Surgery only: n=103 (7.6%)	**Risk:**<br>N=131 (/1284=10.2%) suffer from Fatigue.<br>Of those:<br>n=25: Grade 1<br>n=98: Grade 2 (indicates moderate fatigue or that causing some difficulty performing some activities of daily living)<br>n= 8: Grade 3/4/5 (Grade 3: severe fatigue interfering with activities of daily living; Grade 4: disabling fatigue)<br>**Risk factors for fatigue in multivariable logistic regression analysis adjusted for follow-up duration and age at diagnosis:**<br>• Female vs. male: RR 2.77 (95% CI 1.94-3.94)<br>• Radiotherapy to head and/or neck vs. none: RR 1.76 (95% CI 1.14-2.71)<br>• Radiotherapy to thorax and/or abdomen vs. none: 1.09 (95% CI 0.64-1.86)<br>• Radiotherapy to head and/or neck and thorax and/or abdomen including craniospinal vs. none: RR 2.43 (95% CI 1.54-3.82)<br>• Radiotherapy to extremities only vs. none: RR 0.99 (95% CI 0.40-2.44)<br>• TBI* vs. none: RR 1.67 (95% CI 0.62-4.47)<br>• Anthracyclines vs. none: RR 1.84 (95% CI 0.99-3.42)<br>• Alkylating agents vs. none: RR 1.40 (95% CI 0.81-2.42)<br>• Anthracyclines and alkylating agents vs. none: RR 1.33 (95% CI 0.75-2.37)<br>• Other chemotherapy only vs. none: RR 1.31 (95% CI 0.74-2.30)<br>• Surgery yes vs. no: RR 1.09 (95% CI 0.76-1.58)<br><br>• *TBI=total body irradiation	Selection bias: 0<br>Original cohort consists of n=2596 patients. Only survivors who survived for at least 5 years were included in the study cohort.<br>Attrition bias: 1<br>Response rate 94.3%<br>Detection bias: 0<br>Questionnaire survey, no blinding possible.<br>Confounding: 1<br>Multivariable logistic regression was used to evaluate treatment-related risk factors.<br>**Total quality: 2/4**

Table S11 continued

1. What is the **risk and what are risk factors for suffering from Fatigue** in CAYA survivors?				
Sterkenburg et al. Survival, hypothalamic obesity, and neuropsychological/psychosocial status after childhood-onset craniopharyngioma: newly reported long-term outcomes. 2015				
**Study Design<br>Treatment era<br>Years of follow-up<br>Fatigue measurement**	**Participants**	**Treatment**	**Main outcomes**	**Quality assessment<br>Remarks**
**Study Design:**<br>Cross-sectional study, Questionnaire survey<br><br>**Treatment era:**<br>Diagnosed in the years: 1966-2000<br><br>**Years of follow-up:**<br>Median follow-up time: **16.3 years (range 9.8-36.4)**<br><br>**Fatigue measurement: Multidimensional Fatigue Inventory (MFI-20)**<br>EORTC QLQ-C30 score<br><br>**Country:**<br>Germany	**Sample size:**<br>N=108<br><br>**Diagnoses:**<br>• Childhood-onset craniopharyngioma<br>- n=52 (48%) with hypothalamic involvement (HI)<br>- n=25 (23%) without HI<br>n= 31 (29%) not specified<br><br>**Age at diagnosis:**<br>Median 8.1 years (range 0.05-18.8)<br><br>**Age at study:**<br>Median 24.8 years (range 14.8-42.7)<br><br>**Controls:**<br>Siblings, but not for Fatigue measurements (see remarks)	Degree of resection:<br>• Total n=44 (41%)<br>- n=21 (40%) with hypothalamic involvement (HI)<br>- n=13 (52%) without HI<br>• Subtotal n=54 (50%)<br>- n=29 (56%) with HI<br>- n=10 (40%) without HI<br><br>Radiotherapy:<br>• All n=36 (33%)<br>- n=20 (38%) with HI<br>- n=7 (28%) without HI<br><br>Repeated surgery:<br>• All n=23 (21%)<br>- n=17 (33%) with HI<br>- n=3 (12%) without HI	"In the MFI-20 questionnaire, participants with HI showed a higher score in the domains of physical fatigue (mean score of 9.7 vs. 7.2) and reduced motivation (mean score of 7.8 vs. 6.3). The scores of the other MFI-20 domains (general fatigue, reduced activity and mental fatigue) were comparable in CP participants with and without HI."<br><br>**Risk EORTC QLQ-C30 score:**<br>No HI involvement: median: ca. 21% (0%=no fatigue; 100%=very fatigued)<br>HI involvement: median: ca. 37% (0%=no fatigue; 100%=very fatigued)<br><br>**Risk MFI-20:** All five domains can have a score from 4-20.<br>General Fatigue:<br>• No HI involvement: Median: ca. 9<br>• HI involvement: Median: ca. 10<br>Physical Fatigue: (p=0.024 between HI-no HI)<br>• No HI involvement: Median: 7.2<br>• HI involvement: Median: 9.7<br>Reduced activity:<br>• No HI involvement: Median: ca. 6<br>• HI involvement: Median: ca. 8<br>Reduced motivation: (p=0.042 between HI-no HI)<br>• No HI involvement: Median: ca. 5<br>• HI involvement: Median: ca. 7<br>Mental fatigue:<br>• No HI involvement: Median: ca. 6<br>• HI involvement: Median: ca. 6.5<br><br>**Risk factors:**<br>We do not extract risk factors, as this study did not perform a multivariable analysis.	Selection bias: 0<br>Patients were recruited from a multinational CP registry, but not clear whether that's population-based.<br>Attrition bias: 0<br>Originally n=280 patients. N=165 were contacted (58.9%), n=108 participated in the FU survey (38.6%)<br>Detection bias: 0<br>Questionnaire survey, no blinding possible.<br>Confounding: 0<br>Only descriptive statistics used.<br><br>**Total quality: 0/4**<br><br>They had a sibling control group, but only for the psychosocial status questionnaire, not for the Fatigue outcomes.

Table S11 continued

1. What is the **risk and what are risk factors for suffering from Fatigue** in CAYA survivors?				
Khan et al. Neurologic morbidity and quality of life in survivors of childhood acute lymphoblastic leukemia: a prospective cross-sectional study. 2014				
**Study Design**<br>**Treatment era**<br>**Years of follow-up**<br>**Fatigue measurement**	**Participants**	**Treatment**	**Main outcomes**	**Quality assessment**<br>**Remarks**
**Study Design:**<br>Prospective, single institution, cross-sectional study<br><br>**Treatment era:**<br><br>**Years of follow-up:**<br>Median time from diagnosis 10.2 years (range 5-22.7 years)<br><br>**Fatigue measurement:**<br>Criteria proposed by Cella et al. (Cella D, Davis K, Breitbart W, Curt G. Fatigue Coalition. Cancer-related fatigue: prevalence of proposed diagnostic criteria in a United States sample of cancer survivors. J Clin Oncol. 2001;19:3385-91.<br>and<br>Common Terminology Criteria for Adverse Events v4.0 (CTCAE)<br>and<br>The Brief Fatigue Inventory<br><br>**Country:**<br>USA	**Sample size:**<br>N=162<br><br>**Diagnoses:**<br>• Childhood acute lymphoblastic leukemia (ALL)<br><br>**Age at diagnosis:**<br>Median age at cancer diagnosis 3.9 years (range 0.4-18.6 years)<br><br>**Age at study:**<br>Median age at study enrollment 15.7 years (range 6.9-29.0 years)<br><br>**Controls:**	Number of intrathecal chemotherapy doses (all participants received triple intrathecal therapy with cytarabine, methotrexate and hydrocortisone):<br>9-12: n=100 (61.7%)<br>≥13: n=62 (38.3%)<br><br>CNS radiation: n=23 (14%)<br><br>Intravenous methotrexate dose ≥5mg/m^2: n=25 (15%)	**Risk:**<br>Fatigue was determined in 35 (21.6%) participants: 21 (13%) with mild (CTCAE grade-1), 11 (6.8%) with moderate (CTCAE grade-2), and 3 (1.8%) with severe fatigue (CTCAE grade-3).<br>This was confirmed by examining scores on the Brief Fatigue Inventory where three participants scored in the severe range (mean score ≥7) and 12 had moderate fatigue (mean score >4).<br><br>**Risk factors for fatigue from multivariate logistic regression analyses:**<br>• History of leukemia relapse vs. none OR=8.35, 95% CI: 1.16-59.93, p<0.03<br>Unclear what other variables were included in the model, only history of leukemia relapse is reported.	Selection bias: 0<br>“An introductory letter was mailed to all potential participants” - unclear how large the original cohort was.<br>Attrition bias: 0<br>N=432 met eligibility criteria., n=260 were approached to participate.<br>N=162 participants (response rate 37%)<br>Detection bias: 0<br>Questionnaire survey, no blinding possible.<br>Confounding: 1<br>Multivariable analysis were used.<br><br>**Total quality: 1/4**

Table S11 continued

1. What is the **risk and what are risk factors for suffering from Fatigue** in CAYA survivors?				
Calaminus et al. Quality of life in long-term survivors following treatment for Hodgkin's disease during childhood and adolescence in the German multicenter studies between 1978 and 2002. 2014				
**Study Design<br>Treatment era<br>Years of follow-up<br>Fatigue measurement**	**Participants**	**Treatment**	**Main outcomes**	**Quality assessment<br>Remarks**
**Study Design:**<br>Cross-sectional study; Questionnaire survey<br><br>**Treatment era:**<br>1978-2002<br><br>**Years of follow-up:**<br>Time (in years) since diagnosis: mean 15.26 (range 4.24-28.73)<br><br>**Fatigue measurement:**<br>EORTC QLQ-C30<br><br>**Country:**<br>Germany	**Sample size:**<br>N=725<br><br>**Diagnoses:**<br>• Hodgkin's disease (HD)<br><br>**Age at diagnosis:**<br>Mean 13.63 years (standard deviation (SD) 3.09 years)<br><br>**Age at study:**<br>Mean 28.44 years (SD 5.21 years)<br><br>**Controls:**<br>The sample of HD survivors was compared to an age-adjusted sub-sample of the German norm population (all participants included were between 21 and 41 years, n=659) randomly drawn from a major population-based, representative norm group.<br>Mean age at study: 32.69 years (SD 5.68 years)	Maximum dose radiotherapy:<br>• None 30 (4.1%)<br>• ≤20 Gy 167 (23.1%)<br>• >20≤30 Gy 299 (41.2%)<br>• >30 Gy 229 (31.6%)<br><br>Chemotherapy cycles:<br>• 0: 28 (3.9%)<br>• 2: 334 (46.1%)<br>• 3: 1 (0.1%)<br>• 4: 155 (21.4%)<br>• 6: 207 (28.7%)	**Risk:**<br>Stratified by sex:<br>Males: mean score survivors: 19.02 (SD 21.7) vs. controls 7.85 (SD14.6)<br>Females: mean score survivors: 26.57 (SD 24.8) vs. controls 14.02 (SD 20.09)<br><br>**Risk factors for fatigue from three-way factorial ANOVA test:**<br>Not reported in detail.	Selection bias: 0<br>Original cohort: 2169 patients eligible. N=725 participated in the survey → <75%.<br>Attrition bias: 0<br>Only n=725/2169 answered the fatigue question.<br>Detection bias: 0<br>Questionnaire survey, no blinding possible.<br>Confounding: 1<br>Three-way factorial ANOVA test<br><br>**Total quality: 1/4**

Table S11 continued

1. What is the **risk and what are risk factors for suffering from Fatigue** in CAYA survivors?				
Yi et al. Perceived long-term and physical health problems after cancer: Adolescent and young adult survivors of childhood cancer in Korea. 2014				
**Study Design<br>Treatment era<br>Years of follow-up<br>Fatigue measurement**	**Participants**	**Treatment**	**Main outcomes**	**Quality assessment<br>Remarks**
**Study Design:**<br>Cross-sectional study, questionnaire survey<br>**Treatment era:**<br><br>**Years of follow-up:**<br>Mean time since diagnosis 12.03 years (standard deviation (SD) 5.94 years; range 2-29 years)<br>**Fatigue measurement:**<br>Survivors could indicate whether they suffer from fatigue (yes/no) as one item of ten.<br><br>SF-8 (Medical Outcomes Study Short Form-8)<br>**Country:**<br>Korea	**Sample size:**<br>N=225<br>**Diagnoses:**<br>• Hematological cancers n=159 (71.9%)<br>• Solid or soft tissue tumors n=32 (14.5%)<br>• CNS or brain tumors n=30 (13.6%)<br>**Age at diagnosis:**<br>Mean: 9.89 years (range 0-18 years)<br>**Age at study:**<br>Mean: 21.9 years (range 15-38 years)<br>**Controls:**	Not reported.	**Risk:**<br>Chronic fatigue: Yes 58/225 = 25.78%<br>**Risk factors:**<br>We do not extract risk factors, as this study did not perform a multivariable analysis.	Selection bias: 0<br>Patients were recruited through websites and support groups.<br>Attrition bias: 1<br>All n=225 participants were included in the analysis.<br>Detection bias: 0<br>Questionnaire survey, no blinding possible.<br>Confounding: 1<br>Multivariate regression analyses were used, but not with Fatigue as the dependent variable.<br>**Total quality: 2/4**

Table S11 continued

1. What is the **risk and what are risk factors for suffering from Fatigue** in CAYA survivors?				
Zeller et al. Chronic Fatigue in Long-term Survivors of Childhood Lymphomas and Leukemia: Persistence and Associated Clinical Factors. 2014				
**Study Design<br>Treatment era<br>Years of follow-up<br>Fatigue measurement**	**Participants**	**Treatment**	**Main outcomes**	**Quality assessment<br>Remarks**
**Study Design:**<br>Case-control study<br>**Treatment era:**<br>1970-2002<br>**Years of follow-up:**<br>Median 25.3 years (range 11.3-39.9)<br>**Fatigue measurement: Fatigue Questionnaire (FQ)**<br>**Country:**<br>Norway	**Sample size:**<br>Total n=62/102<br>**Diagnoses:**<br>• Lymphoma n=33<br>• Acute lymphoblastic leukemia (ALL) n=29<br>**Age at diagnosis:**<br>Not mentioned.<br>**Age at study:**<br>Mean 34.05 years<br>**Years of follow-up:**<br>Mean 23.5 years<br><br>Controls did not differ from "cases" (with chronic fatigue (CF)) in sex, age at study, diagnosis, therapy, follow-up time	Radiation therapy:<br>• CF: 43%<br>• Controls: 57%<br>Cum. Anthracycline dose (mg):<br>• CF: mean 166.2 (SD 139.9)<br>• Controls: 170.0 (SD 127.6)	**Risk:**<br>No prevalence measure given, case-control study!<br>FQ total score:<br>CF: median 20.0 (range 13-32)<br>Controls: median 10.5 (range 4-24)<br>CF cases had significantly higher levels in FQ than controls (p<0.001)<br>**Risk factors for persistent chronic fatigue (PCF) caseness from multiple logistic regression analysis:**<br>• Insomnia: not significant<br>• PHQ9 score: not significant<br>• Pain severity score: not significant<br>• Number of steps per day: not significant<br>• Level of depressive symptoms (PHQ8 score) remained the only significant predictor of PCF (OR 1.3, 95%CI:1.1-1.7, p=0.014)	Selection bias: 0<br>Original cohort was 430 survivors, only 102 were included for this study.<br>Attrition bias: 0<br>62/102 were analyzed.<br>Detection bias: 0<br>Questionnaire survey, no blinding possible.<br>Confounding: 1<br>Multivariate statistics were used.<br>**Total quality: 1/4**

Table S11 continued

1. What is the **risk and what are risk factors for suffering from Fatigue** in CAYA survivors?				
McClellan et al. Understanding the functional late effects and informational needs of adult survivors of childhood cancer. 2013				
**Study Design**<br>**Treatment era**<br>**Years of follow-up**<br>**Fatigue measurement**	**Participants**	**Treatment**	**Main outcomes**	**Quality assessment**<br>**Remarks**
**Study Design:**<br>Descriptive, mixed methods survey<br>- Questionnaires<br>- Qualitative content analysis of additional information provided in the questionnaire.<br>**Treatment era:**<br>Not stated<br>**Years of follow-up:**<br>n.a.<br>**Fatigue measurement:**<br>No standardized fatigue measurement.<br>**Country:**<br>USA	**Sample size:**<br>N=271<br>**Diagnoses:**<br>• Grouped into:<br>• Leukemia/lymphomas (48%)<br>• Solid tumors (33%)<br>• Brain tumors (19%)<br>**Age at diagnosis:**<br>Mean age 10 yrs (5.22 SD)<br>**Age at study:**<br>mean age of 24 years (18 to 38)<br>**Controls:**<br>none	Describe treatment intensity as defined by: ITR-2 (Werba et al.,2007)<br>92% received at a minimum moderately intense treatment<br>50% received higher intesity treatmetn, including relapse protocols or transplant	**Risk:**<br>Main outcome: Number of late effects from a list compiled by the authors + late effects added in an open question option by the survivors.<br><br>The overall incidence of fatigue in survivors in this sample was 30% but brain tumor survivors reported 47%<br>**Risk factors:**<br>Data was not extracted for the risk factors, because no multivariable analyses were done.	Selection bias: 1<br>response rate of 47.5%, convenience sample.<br>Recruited from tumor registries at two US hospitals. Excluding those not receiving treatment from an oncologist.<br>Attrition bias: 1<br>N=710 invited<br>N = 139 unknown address<br>N = 271% responded (47.5%)<br>Higher response rate among non-Hispanic whites than blacks.<br>Detection bias: 0<br>Not possible<br>Confounding: 0<br>Only simple statistics and qualitative analyses done<br>**Total quality: 2/4**

Table S11 continued

1. What is the **risk and what are risk factors for suffering from Fatigue** in CAYA survivors?				
Kanellopoulos et al. Factors Associated With Poor Quality of Life in Survivors of childhood Acute Lymphoblastic Leukemia and Lymphoma. 2013				
**Study Design<br>Treatment era<br>Years of follow-up<br>Fatigue measurement**	**Participants**	**Treatment**	**Main outcomes**	**Quality assessment<br>Remarks**
**Study Design:**<br>- Questionnaire study<br>- Recruited from hospital records<br><br>**Treatment era:**<br>NHL and HL: 1970-2000<br>ALL: 1970-2002<br><br>**Years of follow-up:**<br>21 years (range: 7–39 years)<br><br>**Fatigue measurement:**<br>**The fatigue questionnaire (FQ). (Chalders fatigue questionnaire)**<br><br>**Country:**<br>Norway	**Sample size:**<br>N=285<br><br>**Diagnoses:**<br>• N= 91 Hodgkin lymphoma (HL)<br>• N=45 Non-Hodgkin (NHL)<br>• N = 149 Acute lymphoblastic leukemia (ALL)<br><br>**Age at diagnosis:**<br>10 years (range: 0–18 years)<br>ALL patients being younger at diagnosis (median: 5, range: 0–16 years) than lymphoma patients (median: 14, range: 2–18 years)<br><br>**Age at study:**<br>30 years (range: 18–54 years),<br><br>**Controls:**<br>Age matched controls from the general population (Statistics Norway)	HL: The majority of the patients had received a combination of irradiation of the involved fields and a chemotherapy regimen comprising alkylating agents, podophyllotoxins, vinka alkaloids, low-dose anthracyclines, and glucocorticoids.<br><br>NHL: From the late 1980s onward, defined protocols (Berlin–Frankfurt–Münster-regimens, CHOP [cyclophosphamide, doxorubicin, vincristine, and prednisolone]) have been used. Infrequently, the protocols included limited-field radiotherapy.<br><br>ALL: was predominantly based on chemotherapy only. The treatment protocols used [Norwegian protocol until 1980, NOPHO (Nordic Society of Paediatric Haematology and Oncology) protocols from 1981] have previously been described in detail [13–15]. Prophylactic craniospinal irradiation (18–24 Gy) has not been used routinely in Norway after 1975. Its use was restricted to a small number of patients with high risk disease, overt CNS leukemia or relapse.	**Risk:**<br>Total fatigue: mean=13.9 (SD 5.3)<br>Cases of chronic fatigue: 27%<br><br>SF-36 Vitality:<br>Survivors 51.1 (SD 21.6)<br>Controls 60.1 (SD 19.3)<br>$p_{adj}<0.001$<br>(adjusted for education, paired relationship, and work)<br><br>Fatigue as a predictor of Qol (SF-36 dichotomised into poor and good)<br>Fatigue OR 1.17 (95% CI: 1.08-1.27) predicting QoL, adjusted for a range of demographic and health variables.<br><br>Population scores for fatigue not provided.<br><br>**Risk factors:**<br>Fatigue used as a predictor variable only	Selection bias: 1<br>- Random sample selected from national cohort or from hospital records of Norway's largest hospital (more than 50% of childhood cancer patients)<br>- Response rate overall: 69%<br>Attrition bias: 1<br>HL/NHL:<br>N=220 invited<br>N = 141 responded (67%)<br><br>ALL:<br>N=210 invited<br>N = 160 agreed to participate in clinical study<br>N = 155 completed questionnaires (74%)<br><br>Excluded N = 10 were excluded due to incomplete data<br><br>Compared to respondents, non-responders were significantly more likely to be male, and lymphoma patients. There were no significant differences concerning age at diagnosis, age at survey, or followup time<br>Detection bias: 0<br>Not possible, questionnaire study<br>Confounding: 0<br>fatigue as a predictor – not as an outcome<br><br>**Total quality: 2/4**

Table S11 continued

1. What is the **risk and what are risk factors for suffering from Fatigue** in CAYA survivors?				
Hamre et al. Serum cytokines and chronic fatigue in adults surviving after childhood leukemia and lymphoma 2013b				
**Study Design<br>Treatment era<br>Years of follow-up<br>Fatigue measurement**	**Participants**	**Treatment**	**Main outcomes**	**Quality assessment<br>Remarks**
**Study Design:**<br>Questionnaire and Clinical study<br>**Treatment era:**<br>NHL and HL: 1970-2000<br>ALL: 1970-2002<br>**Years of follow-up:**<br>21 years (range: 7–39 years)<br>**Fatigue measurement:**<br>**The fatigue questionnaire (FQ). (Chalders fatigue questionnaire)**<br>→ Scored chronic fatigue (CF) or not<br>**Country:**<br>Norway	**Sample Size:**<br>n=232<br>**Diagnoses:**<br>• n=68 Hodgkin lymphoma (HL)<br>• n=47 Non-Hodgkin (NHL)<br>• n=117 Acute lymphoblastic leukemia (ALL)<br>**Age at diagnosis:**<br>Median 9.6 (Range 0.3–18.0) years,<br>**Age at study:**<br>Median 29.7 (Range 18.6–54.5) years<br>**Controls:**<br>Survivors without chronic fatigue	**Chemotherapy only:**<br>90% of ALL survivors<br>57% of NHL survivors<br>37% HL<br>**Radiation only:**<br>0% ALL<br>2% NHL<br>18% HL<br>**Chemo and radiation therapy:**<br>10% ALL<br>41% NHL<br>63% HL<br>A total of 15 survivors had received radiotherapy to the central nervous system (CNS), 12 being ALL survivors. Among the 62 survivors who had undergone treatment with mediastinal irradiation 90% were HL survivors	**Risk:**<br>In total: 28% had CF<br>Highest for HL survivors (36%); NHL, 26% and ALL 24%<br>**Risk factors:**<br>First, the impact of possible confounders were explored in univariate analyses, variables which displayed odds ratio's (OR) with p-values ≤0.1, were included in the final analysis (diagnosis, age, gender, BMI and reduced heart function).<br>Results of logistic regression analysis (unclear whether uni- or multivariable):<br>• Older age at survey; Age OR=1.04 (95% CI: 1.00–1.1) *p*=0.03<br>• Female gender OR=1.09 (95%CI: 0.6-1.9), p=0.8<br>• Diagnosis: NHL (Ref. ALL): OR=1.3 (95% CI: 0.6–2.8), *p*=0.6<br>• Diagnosis: HL (Ref. ALL) OR=1.8 (95% CI: 0.9–3.3), *p* =0.08<br>• Smoking OR=1.34 (95%CI=0.7-2.5), p=0.3<br>• BMI OR=1.1 (95%CI:1.0-1.1), p=0.1<br>• Regular use of analgesics OR=1.6 (95%CI:0.7-3.7), p=0.2<br>• Reduced heart function OR=1.8 (95%CI:1.0-3.3), p=0.06<br>• T-cell origin: Yes (Ref. No): OR=10.3 (95% CI: 2.7–39.3), *p*=0.01<br>• T-cell origin: Unknown (Ref. No): OR=1.7 (95%CI:0.7-3.9), p=0.2<br>• CNS-irradiation OR=0.9 (95%CI:0.3-2.9), p=0.9<br>• B-symptoms at diagnosis: Yes (Ref. No): OR =2.5 (95% CI: 1.0–6.2), *p*=0.05;<br>• B-symptoms at diagnosis: Unknown (Ref. No): OR=1.1 (95% CI:0.4–3.1), p=0.9<br>• A multivariable logistic regression model with CF as outcome and various cytokine level measures as predictor variables, no associations were significant.	Selection bias: 1<br>- Unselected sample.<br>- Sample recruited from national cohort or from hospital records of Norway's largest hospital (more than 50% of childhood cancer patients)<br>- Response rate overall: 69%<br>Attrition bias: 1<br>-Eligible: n = 434<br>-Non-responses: n = 134<br>-Excluded for various reasons (questionnaire data only, pregnant, secondary cancer etc)N = 68<br>-Included N = 232<br>Detection bias: 0<br>Not possible, questionnaire study<br>Confounding: 1<br>Multivariable analysis were used.<br>**Total quality: 3/4**

Table S11 continued

1. What is the **risk and what are risk factors for suffering from Fatigue** in CAYA survivors?				
Gordijn et al. Sleep, fatigue, depression, and quality of life in survivors of childhood acute lymphoblastic leukemia 2013				
**Study Design**<br>**Treatment era**<br>**Years of follow-up**<br>**Fatigue measurement**	**Participants**	**Treatment**	**Main outcomes**	**Quality assessment**<br>**Remarks**
**Study Design:**<br>Questionnaire study<br>Child and parental proxy reports<br><br>**Treatment era:**<br>1997-2008<br><br>**Years of follow-up:**<br>36 (interquartile range 22–62) months after finishing treatment<br><br>**Fatigue measurement:**<br>PedsQL^{TM} multidimensional fatigue scale (child and parent reports)<br><br>**Country:**<br>The Netherlands	**Sample Size:**<br>n = 62 children<br><br>**Diagnoses:**<br>ALL<br><br>**Age at diagnosis:**<br>Not provided<br><br>**Age at study:**<br>Mean age: 9.7 (SD 3.2), range 5 – 17 yrs<br><br>**Controls:**<br>Dutch norm references	All participants had been successfully treated according to the Dutch Childhood Oncology Group (DCOG) ALL-9 or ALL-10 protocol between May 1997 and February 2008 in the VU University Medical Center Amsterdam, the University Medical Center Utrecht or the Radboud University Nijmegen Medical Center in the Netherlands. Based on clinical and biological factors and on the response to treatment, patients treated according to the ALL-9 protocol were classified in a nonhigh risk (NHR) or a high risk (HR) group and patients treated according to the ALL-10 protocol were classified in a standard risk (SR), a medium risk (MR) or a high risk (HR) group. Both ALL treatment protocols did not include cranial irradiation.	**Risk**<br>Effect sizes varied from moderate to large, with parents rating the ALL survivors as having more general fatigue and total fatigue than the norm. Fatigue reported by survivors themselves did not differ from the Dutch norm:<br>Child report: Total fatigue mean 78.73* (SD 12.49) vs. Dutch norm mean 76.84* (SD 12.67) (p=0.399)<br>Parent report: Total fatigue mean 74.25* (SD 17.94) vs. Dutch norm 81.21* (SD 12.62) (p=0.004)<br>(*higher score = less symptoms of fatigue)<br><br>**Risk factors:**<br>Data was not extracted for the risk factors, because no multivariable analyses were done.	Selection bias: 1<br>- Response rate overall: 42%<br>- Recruited from treating hospital<br>Attrition bias: 1<br>-invited: n = 146<br>-responses from n= 62<br>No significant differences emerged among participants and non-participants with respect to age, gender, treatment protocol, risk group stratification, and time since end of treatment.<br>Detection bias: 0<br>Not possible, questionnaire study<br>Confounding: 0<br>fatigue as a predictor – not as an outcome<br><br>**Total quality: 2/4**

Table S11 continued

1. What is the **risk and what are risk factors for suffering from Fatigue** in CAYA survivors?				
Manley et al. Sleep dysfunction in long term survivors of craniopharyngioma 2012				
**Study Design<br>Treatment era<br>Years of follow-up<br>Fatigue measurement**	**Participants**	**Treatment**	**Main outcomes**	**Quality assessment<br>Remarks**
**Study Design:**<br>Questionnaire and Clinical study<br>**Treatment era:**<br>2003-2007<br>**Years of follow-up:**<br>median follow up time was 130.5 months (range, 24–312 months)<br>**Fatigue measurement:**<br>Unsure – clinic specific symptom list assessed using questionnaires and interview<br>No standardized tool<br>**Country:**<br>USA	**Sample Size:**<br>n = 28<br>**Diagnoses:**<br>• craniopharyngioma<br>**Age at diagnosis:**<br>median age at the time of diagnosis was 8 years (range 2–16 years).<br>**Age at study:**<br>29.7 (18.6–54.5) years<br>**Controls:**<br>Survivors without chronic fatigue	Surgery for all (gross or subtotal resection)<br>Some radiotherapy (N = 22?)<br>No chemotherapy	**Risk**<br>14 of 28 reported fatigue (50%)<br>**Risk factors:**<br>Data was not extracted for the risk factors, because no multivariable analyses were done.	Selection bias: 1<br>- Recruited from survivorship care clinic at hospital<br>- - participation rate: 39,5%<br>Attrition bias: 1<br>-Eligible: n = 71<br>Of which:<br>- n = 27 no clinical data available<br>- n = 15 lost to follow up<br>- n = 1 diseased<br>- n = 28 included in study<br>Detection bias: 0<br>Not possible, questionnaire study<br>Confounding: 0<br>fatigue as a predictor – not as an outcome<br>**Total quality: 2/4**

Table S11 continued

1. What is the **risk and what are risk factors for suffering from Fatigue** in CAYA survivors?				
Nagai et al. Fatigue in survivors of childhood acute lymphoblastic and myeloid leukemia in Japan. 2012				
**Study Design<br>Treatment era<br>Years of follow-up<br>Fatigue measurement**	**Participants**	**Treatment**	**Main outcomes**	**Quality assessment<br>Remarks**
**Study Design:**<br>Questionnaire<br><br>**Treatment era:**<br>Not provided<br><br>**Years of follow-up:**<br>Mean 5.8 years (SD 3.8)<br><br>**Fatigue measurement:**<br>Self-made (12 items)<br>Chalder fatigue scale<br><br>**Country:**<br>Japan	**Sample Size:**<br>n = 81<br><br>**Diagnoses:**<br>ALL 77.8% and AML 22.2%<br><br>**Age at diagnosis:**<br>Mean 6.7 years (SD 3.5)<br><br>**Age at study:**<br>Mean 14.1 years (SD 5.7)<br><br>**Controls:**<br>n = 243 healthy controls	Chemotherapy only n=45 (55.6%)<br>Chemotherapy + radiation n=8 (9.9%)<br>Chemotherapy + SCT n=10 (12.3%)<br><br>Chemotherapy + radiation + SCT n=18 (22.2%)	**Risk**<br>Fatigue prevalence not reported<br>Fatigue scores:<br>Physical fatigue: mean 3.5 vs. 4.2 (in controls), p<0.05<br>Decreased function: mean 3.7 vs. 4.2 (in controls), p=0.084<br>Altered mood: mean 2.6 vs. 2.9 (in controls), p=0.31<br>Total: mean 9.8 vs. 11.4 (in controls), p<0.05<br>Mean total fatigue scores were significantly lower in leukemia survivors (indicating less fatigue) than in controls.<br><br>NB their Fatigue measure confounded with questions consistent with symptoms of depression and anxiety<br><br>**Risk factors from multiple regression analysis:**<br>Total fatigue was associated with:<br>- Present age (years): β=0.24, p<0.05<br>- Gender: β=0.35, p>0.05<br>- Diagnosis: β=-0.02, p>0.05<br>- Cranial irradiation: β=-0.04, p>0.05<br>- Total body irradiation: β=2.72, p>0.05<br>- Duration after completion of treatment (years): β=-0.45, p<0.05	Selection bias: 1<br>- Recruited from treating hospital and attended follow-up clinic<br><br>- participation rate: 90%<br>Attrition bias: 1<br>-total available survivors: n = 90<br>- n = 81 included in study<br>Detection bias: 0<br>Not possible, questionnaire study<br>Confounding: 0<br><br>**Total quality: 2/4**

Table S11 continued

1. What is the **risk and what are risk factors for suffering from Fatigue** in CAYA survivors?				
Mört et al. Fatigue in Young Survivors of Extracranial Childhood Cancer: A Finnish Nationwide Survey. 2011				
**Study Design<br>Treatment era<br>Years of follow-up<br>Fatigue measurement**	**Participants**	**Treatment**	**Main outcomes**	**Quality assessment<br>Remarks**
**Study Design:**<br>Cross-sectional quantitative study (Questionnaires)<br><br>**Treatment era:**<br>Not stated, but calculated from that the oldest survivor were 18 at study mean they were diagnosed apr. from 1988-2001<br><br>**Years of follow-up:**<br>N/A<br><br>**Fatigue measurement:**<br>**PedsQL Multidimensional Fatigue Scale**<br><br>**Country:**<br>Finland	**Sample size:**<br>N=199<br><br>**Diagnoses:**<br>• Leukemia n=110 (55%)<br>• Non-Hodgkin Lymphoma n=13 (13%)<br>• Hodgkin Lymphoma n=5 (3%)<br>• Neuroblastoma n=15 (8%)<br>• Wilms tumor n=16 (8%)<br>• Gondal tumor n=7 (4%)<br>• Osteosarcoma n=6 (3%)<br>• Retinoblastoma n=6 (3%)<br>• Soft tissue sarcoma n=13 (7%)<br>• Other n=8 (4%)<br><br>**Age at diagnosis:**<br>Mean 3.6 years old.<br>Range 0-12<br><br>**Age at study:**<br>Mean 14.4 years old.<br>Range 11-18<br><br>**Controls:**<br>Matched controls N=252	Surgery only n=7 (4%)<br><br>Chemotherapy (alone or with surgery) n=115 (58%)<br><br>Radiation (alone or with chemotherapy or surgery) n=32 (16%)<br><br>Stem cell transplantation n=19 (10%)<br><br>Not known or stated N=19 (10%)	**Risk:**<br>PedsQL Multidimensional Fatigue Scale captures total fatigue (TF), general fatigue (GF), sleep or rest fatigue (SF), and cognitive fatigue (CF)<br>• The controls reported significantly more fatigue than the survivors (Total fatigue: Survivors Median 83.33; Controls Median 80.56, p<0.01).<br>• Survivors scored more Total fatigue when compared with their parent proxy scores, but not statistically significant (Total fatigue: Survivors Median 83.33; Parents of Survivors Median 84.03, p>0.05)<br><br>**Risk factors for Total Fatigue from multivariate regression analysis:**<br>Lower scores indicate more fatigue.<br>• Age at study: β=-1.87, p<0.001<br>• Gender: female (Ref. male) β =2.99, p>0.05<br>• Diagnosis: NHL (Ref. leukemia) β =-2.49, p>0.05<br>• Diagnosis: Sarcoma (Ref. leukemia) β =-13.28, p<0.01<br>• Diagnosis: NBL (Ref. leukemia) β =-2.3, p>0.05<br>• Diagnosis: Other (Ref. Leukemia) β =-0.85, p>0.05<br>• Treatment: Chemotherapy (Ref. surgery alone) β =-4.2, p>0.05<br>• Treatment: Radiation (Ref. surgery alone) β =-8.73, p>0.05<br>• Treatment: SCT (Ref. surgery alone) β =-3.17, p>0.05<br>• Treatment: Other treatment (Ref. surgery alone) β =-5.09, p>0.05<br>• Length of survival: More than 10 years (Ref. 10 years or less) β =-3.6, p>0.05<br>• Additional diagnosis: No (Ref. Yes) β =2.2, p>0.05<br>• Remedial education: No (Ref. Yes) β =-1.43, p>0.05<br>• Overall average grade: β =2.47, p>0.05<br>• Self-rated happiness: No (Ref. Yes) β =-1.13, p>0.05<br>• HRQoL score: β =0.87, p<0.001	Selection bias: 0<br>Population-based study<br>n= 384 received questionnaire.<br>N=199 (53%) replied<br>No information about non-responders<br>Attrition bias: 1<br>Detection bias: 0<br>Questionnaire survey, no blinding possible.<br>Confounding bias: 1<br>Multivariable analysis were used.<br><br>**Total quality: 2/4**

Table S11 continued

1. What is the **risk and what are risk factors for suffering from Fatigue** in CAYA survivors?				
Johannsdottir et al. Increased Prevalence of Chronic Fatigue Among Survivors of Childhood Cancers: A Population-Based Study. 2012				
**Study Design<br>Treatment era<br>Years of follow-up<br>Fatigue measurement**	**Participants**	**Treatment**	**Main outcomes**	**Quality assessment<br>Remarks**
**Study Design:**<br>Cross sectional study.<br>**Treatment era:**<br>1985-2001<br>**Years of follow-up:**<br>**4-20 years**<br>**Fatigue measurement:**<br>**Fatigue Questionnaire (FQ)**<br>**Country:**<br>Norway	**Sample size:**<br>N= 398<br>151 young group (YG) (13-18 years)<br>247 older group (OG) (19 and above)<br>**Diagnoses:**<br>• Acute myeloid leukemia (AML), n=90<br>• Infratentorial astrocytoma (IA) n=125<br>• Wilms tumor (WT) n=183<br>**Age at diagnosis:**<br>1-18 mean 5 years old<br>**Age at study:**<br>13-34<br>**Controls:**<br>N=763	**AML:**<br>Stem cell transplantation n=56 (60%)<br>Chemotherapy only n=34 (40%)<br>**IA:**<br>75% surgery only<br>16% radiotheraphy in addition<br>The rest 9% treatment unknown<br>**WT:**<br>57% surgery and chemotherapy<br>40% supplementary radiotherapy	**Risk:**<br>11% of the survivors had chronic fatigue (significantly more prevalent<br>in the OG (13.6%) than in the YG (6.8%), P<0.05)<br>• Risk of chronic fatigue (CF): Survivors (OG) vs. controls: OR 3.29 (95% CI 1.90-5.70; from multivariable logistic regression, adjusted for age, sex, education, marital status, employment, social benefits)<br>**Risk factors for chronic fatigue in univariate analysis:**<br>• Older aged females had sig. higher levels of fatigue (Mental fatigue (MF); Physical fatigue (PF) and Total fatigue (TF) compared with general population.<br>• Older aged survivors had higher levels of fatigue compared to younger aged survivors (TF 12.4 vs. 10.9; P<0.01, PF 8.0 vs. 7.0; P<0.01, and MF 4.4 vs. 4.0; P<0.05).<br>**Risk factors for chronic fatigue from multivariable logistic regression analysis (n=33 OG; n=44 GP):**<br>• **Age at assessment: OR 1.08 (95% CI 1.01-1.16)**<br>• Females vs. males: OR 1.54 (95% CI 0.94-2.54)<br>• Academic education yes vs. no: OR 0.63 (95% CI 0.36-1.12)<br>• Married/cohabiting yes vs. no: OR 1.09 (95% CI 0.64-1.85)<br>• Gainfully employed yes vs. no: OR 1.18 (95% CI 0.67-2.07)<br>• Receiving social benefits yes vs. no: OR 1.79 (95% CI 0.61-5.26)	Selection bias: 1<br>Population-based Survey study from the Nordic countries (Norway, Denmark, Sweden, Finland, and Iceland).<br>Attrition bias: 0<br>65% response rate among the young group and 74% among the older group<br>n= 567 received questionnaire.<br>N=398 replied<br>Detection bias: 0<br>Questionnaire survey, no blinding possible.<br>Confounding bias: 1<br>Multivariable analysis were used.<br>**Total quality: 2/4**

Table S11 continued

1. What is the **risk and what are risk factors for suffering from Fatigue** in CAYA survivors?				
Kenney et al. Health Status of the Oldest Adult Survivors of Cancer During Childhood. 2010				
**Study Design**<br>**Treatment era**<br>**Years of follow-up**<br>**Fatigue measurement**	**Participants**	**Treatment**	**Main outcomes**	**Quality assessment**<br>**Remarks**
**Study Design:**<br>Cross sectional Survey study<br><br>**Treatment era:**<br>1947-1968<br><br>**Years of follow-up:**<br>36-65<br><br>**Fatigue measurement:**<br>Functional Assessment of Chronic Illness Therapy-Fatigue.<br><br>**Country:**<br>USA	**Sample size:**<br>N=55 (63% response rate)<br><br>**Diagnoses:**<br>• Sarcoma n=18 (33%)<br>• NHL N=10 (18)<br>• Wilms tumor n=10 (18%)<br>• Hodgkin lymphoma n=6 (11%)<br>• Neuroblastoma n=5 (9%)<br>• Other N=6 (11%)<br><br>**Age at diagnosis:**<br>0-18 mean 8 years old<br><br>**Age at study:**<br>51-71 mean 56 years old<br><br>**Controls:**<br>N=32	Surgery only n=4 (7%)<br><br>Radiation only n=15 (27%)<br><br>Chemotherapy only n=14 (26%)<br><br>Radiation and chemotherapy n=22 (40%)	**Risk:**<br>Scores on the fatigue scale range from 0 to 52, with higher scores indicating better functioning and less fatigue; scores <30 can be interpreted as indicating significant fatigue<br><br>Survivors' mean fatigue score of 40.56 (standard deviation [SD] 10.40) was significantly lower that the siblings' mean of 45.19 (SD 6.88, *t*=.2.43, *p*=0.02), indicating more significant problems with fatigue.<br>A larger proportion of survivors had fatigue scores in the clinically significant range (8 of 50 [16%]) compared with siblings (1 of 32 [3.1%]) (OR=5.90), but the difference only approached statistical significance (Fisher exact test, *P*=0.067).<br><br>**Risk factors:**<br>Data was not extracted for the risk factors, because no multivariable analyses were done.	Selection bias: 0<br>single institution cohort Of 1100 survivors in the cohort, 222 were eligible by birth date, 115 for this analysis (68 deceased, rest different reasons) resulting in 107 potential cases. Of them 16 were deceased. So 88 were enrolled in the study.<br>Attrition bias: 0<br>63% response rate<br>n= 88 received questionnaire.<br>N=55 replied<br>Analysis of nonparticipants available similar to respondents on demographic variables.<br>Detection bias: 0<br>Questionnaire survey, no blinding possible.<br>Confounding bias:1<br>Multivariable analysis were used.<br><br>**Total quality: 1/4**

Table S11 continued

1. What is the **risk and what are risk factors for suffering from Fatigue** in CAYA survivors?				
Blaauwbroek et al. The effect of exercise counselling with feedback from a pedometer on fatigue in adult survivors of childhood cancer: a pilot study. 2009				
**Study Design<br>Treatment era<br>Years of follow-up<br>Fatigue measurement**	**Participants**	**Treatment**	**Main outcomes**	**Quality assessment<br>Remarks**
**Study Design:**<br>Intervention study<br><br>**Treatment era:**<br>n.a.<br><br>**Years of follow-up:**<br>Mean since diagnosis 21.8. range 14.7-28.9<br><br>**Fatigue measurement:**<br>Visual Analogue Scale for chronic fatigue (VAS fatigue)<br>Checklist individual strength (CIS)<br><br>**Country:**<br>The Netherlands	**Sample size:**<br>N=46<br><br>**Diagnoses:**<br>• Leukemia n=22 (46.8%)<br>• Malignant lymphoma n=6 (12.8)<br>• Bone tumor n=4 (8.5)<br>• Soft tissue sarcoma n=3 (6.4%)<br>• Wilms tumor n=1(2.1%)<br>• Langerhans cell histiocytosis n=2 (4.3%)<br>• CNS tumor n=6 (12.8%)<br>• Other n=3 (6.4%)<br><br>**Age at diagnosis:**<br>Mean age 8 years. Range 1.5-14.8<br><br>**Age at study:**<br>Median age 29 years. Range 18-61<br><br>**Controls:**<br>N=33 (recruited by the survivors among healthy siblings or peers)	Chemotherapy only 22 (47.8%)<br><br>Surgery only 2 (4.4%)<br><br>Radiotherapy only 0<br><br>Chemo and radiotherapy 22 (47.8)<br><br>Cranial radiation 12 (26.1)	**Risk:**<br>Fatigue was the primary outcome and it was measured with a visual analogue scale for fatigue and the CIS. The CIS is a validated 20-item questionnaire, that is designed to measure four aspects of fatigue that may have been experienced during the previous 2 weeks<br><br>67/254 (26.4%) survivors had a VAS score of ≥70mm.<br>Mean CIS score before the intervention was 81.42 (SD 20.14) for survivors and 47.39 (SD 19.06) for controls, $p<0.0005$.<br><br>**Risk factors:**<br>Data was not extracted for the risk factors, because no multivariable analyses were done.	Selection bias: 0<br><br>Attrition bias: 0<br>n= 486 eligible<br>n=453 were sent questionnaire<br>respons rate 56%.<br>46 were enrolled into the study but eight dropped out<br>Detection bias: 0<br><br>Confounding bias:0<br>Descriptive statistics and Linear regression used.<br><br>**Total quality: 0/4**

Table S11 continued

1. What is the **risk and what are risk factors for suffering from Fatigue** in CAYA survivors?				
Mulrooney et al. Fatigue and Sleep Disturbance in Adult Survivors of Childhood Cancer. A report from the Childhood Cancer Survivor Study (CCSS). 2008				
**Study Design<br>Treatment era<br>Years of follow-up<br>Fatigue measurement**	**Participants**	**Treatment**	**Main outcomes**	**Quality assessment<br>Remarks**
**Study Design:**<br>Epidemiologic study; Sleep questionnaire from the Childhood Cancer Survivor Study (CCSS), sent with the second follow-up questionnaire<br><br>**Treatment era:**<br>Diagnosed between 1970 and 1986<br><br>**Years of follow-up:**<br>Survival for >=5 years following diagnosis;<br>15-19 years 26.1%<br>20-24 years 34.1%<br>25-29 years 26.0%<br>30+ years 13.9%<br><br>**Fatigue measurement:**<br>Fatigue subscale of the Functional Assessment of Chronic Illness Therapy-Fatigue (FACIT-Fatigue)<br><br>**Country:**<br>USA	**Sample size:**<br>1897 survivors and 369 siblings as controls<br><br>**Diagnoses:**<br>Leukemia 15.7%<br>CNS malignancy 15.8%<br>Hodgkin disease 52.5%<br>Soft tissue sarcoma 7.9%<br>Bone cancer 8.2%;<br>*Oversampling of Hodgkin disease survivors due to reports of excessive fatigue in this population*<br><br>**Age at diagnosis:**<br>Diagnosed before the age of 21 years;<br>0-4 years 18.6%<br>5-9 years 20.6%<br>10-14 years 27.6%<br>15+ years 33.3%<br><br>**Age at study:**<br>18-29 years 23.8%<br>30-39 years 46.3%<br>40-49 years 28.0%<br>50+ years 1.8%<br><br>**Controls:**<br>Nearest-age siblings from the study participants (n=369)	**Chemotherapy**<br>Yes 59.1%<br>No 40.9%<br><br>**Radiation**<br>Yes 70.2%<br>No 29.8%	**Risk:**<br>Comparison of mean fatigue scores: Survivors had significantly lower mean fatigue score (40.8) than their siblings (42.0), p=0.02 (comparison adjusted for age at study and sex; lower score indicates more fatigue)<br>Prevalence of fatigue: 364/1897 (19.2%)<br><br>**Risk factors from multivariate logistic regression analysis (cancer- or treatment-related variables):**<br>*OR for being fatigued*<br>• Diagnosis: CNS malignancy (Ref. ALL): OR=1.3, 95%CI:0.8-2.1<br>• Diagnosis: Hodgkin disease (Ref. ALL): OR=1.2, 95%CI:0.7-1.8<br>• Diagnosis: Soft tissue sarcoma (Ref. ALL): OR=1.0, 95%CI:0.6-1.7<br>• Diagnosis: Bone cancer (Ref. ALL): OR=1.3, 95%CI: 0.7-2.3<br>• Age at diagnosis: 0-4 years (Ref. 15+ years): OR= 0.7, 95%CI:0.4-1.2<br>• Age at diagnosis: 5-9 years (Ref. 15+ years): OR=0.9, 95%CI:0.6-1.4<br>• Age at diagnosis: 10-14 years (Ref. 15+ years): OR=0.8, 95%CI:0.6-1.1<br>• Radiation: Yes (Ref. No): OR=1.7, 95%CI:1.3-2.3<br>• Chemotherapy: Yes (Ref. No): OR=1.0, 95%CI:0.8-1.4<br>**Risk factors from multivariate logistic regression analysis (medical conditions and sociodemographic factors) in survivors:**<br>• Female (Ref. male): OR=2.1, 95%CI:1.6-2.7<br>• Congestive heart failure: Yes (Ref. No): OR=2.9, 95%CI:1.4-6.1<br>• Lung fibrosis: Yes (Ref. No): OR=2.9, 95%CI:1.5-5.4<br>• Hypothyroidism: Yes (Ref. No): OR=0.9, 95%CI:0.7-1.3<br>• Depressed: Yes (Ref. No): OR=7.5, 95%CI:5.1-10.9<br>• BMI 30+ kg/m^2: Yes (Ref. No): OR=1.3, 95%CI:0.9-1.7<br>• Marital status: Not married (Ref. Married): OR=2.7, 95%CI:2.0-3.6<br>• Employment status: Not working full time (Ref. working full time): OR=1.2, 95%CI:0.3-1.6<br>• Infant at home <6mo old: Yes (Ref. No): OR=1.9, 95%CI:0.7-5.0	Selection bias: 0<br>Survivors: response rate 72%; Oversampling of Hodgkin disease survivors due to reports of excessive fatigue in this population → no<br>Controls: response rate 73.8% → no<br>Attrition bias: 1<br>Outcome for all included survivors → yes<br>Detection bias: 0<br>Assessors were not blinded → no<br>Confounding: 1<br>Adjusted comparison of mean fatigue scores and multivariate analyses → yes<br><br>**Total quality 2/4**<br><br>**Remarks:**<br>To dichotomize the scales, we classified the lowest 10th percentile of the sibling scores on the FACIT-Fatigue as fatigued.

Table S11 continued

1. What is the **risk and what are risk factors for suffering from Fatigue** in CAYA survivors?				
Aksnes et al. Young survivors of malignant bone tumours in the extremities: a comparative study of quality of life, fatigue and mental distress. 2007				
**Study Design<br>Treatment era<br>Years of follow-up<br>Fatigue measurement**	**Participants**	**Treatment**	**Main outcomes**	**Quality assessment<br>Remarks**
**Study Design:**<br>Cross-sectional quantitative study<br><br>**Treatment era:**<br>Not stated<br><br>**Years of follow-up:**<br>At least 5 years after end of primary treatment, years since diagnosis is only reported stratified by type of diagnosis and sex (mean years since diagnosis around 9-14 years for all subgroups<br>Male EBT survivors 14 years (SD 4.5)<br>Female EBT survivors 11 years (SD 4.8)<br><br>**Fatigue measurement:**<br>**The Fatigue Questionnaire**<br><br>**Country:**<br>Norway	**Sample size:**<br>N= 57 with matched controls (TC and HD)<br><br>**Diagnoses:**<br>• Extremity bone tumor (EBT) n=57<br>Controls:<br>• Testicular cancer n= 62<br>• Hodgkin's n=89<br><br>**Age at diagnosis:**<br>Males EBT survivors mean 20 years (SD 8.2)<br>Females EBT survivors mean 16 years (SD 4.5)<br><br>**Age at study:**<br>Male EBT survivors 34 years (SD 9.4)<br>Female EBT survivors 27 years (SD 4.8)<br><br>**Controls:**<br>Hodgkin n=89<br>Testicular cancer n=89<br>Norm population: five randomly chosen gender- and age-adjusted cases for each EBT survivor (n=285)	Not clear, they had treatment according to one of the osteosarcoma or Ewing tumor protocols of the Scandinavian Sarcoma Group (SSG)	**Risk:**<br>No significant differences in the fatigue scores were observed between the survivor groups. The hypothesis that the EBT survivors, because of more extensive treatment, would display more fatigue than HD survivors and TC survivors, and gender- and age-matched individuals from the general population was not confirmed because EBT survivors hardly differed from HD survivors, TC survivors or NORMs except in the physical dimensions of QoL.<br>EBT survivors had a significantly higher Total fatigue score (p=0.003) compared to their NORMs<br>Total fatigue, mean: EBT: 13.2 (SD 3.8), NORMs: 11.8 (SD 3.9), p=0.003; HD survivors 13.4 (SD 4.8), TC survivors 13.4 (SD 4.7), both p=0.95 compared to EBT<br>Chronic fatigue: n=8 (14%) of EBT, n=27 (10%) of NORMs, p=0.30; n=19 (21%) of HD survivors; n=10 (16%) of TC survivors, both p=0.49 compared to EBT<br><br>**Risk factors:**<br>No risk factors for fatigue were analyzed.	Selection bias: 0<br>Unclear if this is a Population-based study<br>Attrition bias: 1<br>n= 75 received questionnaire 58 responded (77%)<br>No difference between responders and non responders on age, sex, type of treatment or time since diagnoses<br>Detection bias: 0<br>Questionnaire survey, no blinding possible.<br>Confounding bias: 1<br>Multivariable analysis were used.<br><br>**Total quality: 2-3/4**

Table S11 continued

1. What is the **risk and what are risk factors for suffering from Fatigue** in CAYA survivors?				
Meeske et al. Prevalence and Correlates of Fatigue in Long-Term Survivors of Childhood Leukemia. 2005				
**Study Design<br>Treatment era<br>Years of follow-up<br>Fatigue measurement**	**Participants**	**Treatment**	**Main outcomes**	**Quality assessment<br>Remarks**
**Study Design:**<br>Cross sectional, single centre study<br>**Treatment era:**<br>1975-1995<br>**Years of follow-up:**<br>Average time from end of therapy was 13.9 years (range 4-23 years)<br>**Fatigue measurement:**<br>-The Revised–Piper Fatigue Scale (R-PFS)<br>-Profile of Mood State fatigue inertia subscale (POMS)<br>- Rand SF-36 (SF-36) vitality subscale<br>-Symptom Distress Scale (SDS).<br>**Country:**<br>USA	**Sample size:**<br>N=161<br>**Diagnoses:**<br>• Acute lymphoblastic leukemia (ALL)<br>**Age at diagnosis:**<br>0-18<br>Average age at diagnosis was 7.4 years<br>**Age at study:**<br>18-41<br>**Controls:**	Cranial irradiation n=103 (65%)<br>Anthracycline n=104 (66%)<br>BMT n=12 (7%)	**Risk:**<br>Prevalence of fatigue (30%) fell within the general population normal limits (n=48 (30%) were classified as fatigued).<br>Fatigue was the most frequently reported symptom (61%) on the SDS. Distress levels were higher for fatigue than for any other symptom.<br>Survivors' average POMS fatigue-inertia score was 7.2 (standard deviation [SD], 6.3), which is within the normal range reported for college students.<br>Survivors' SF-36 vitality mean score was 63.4 (SD 23.2), which is slightly higher (more energy) than the norms for the general population (61.3; SD 20.2).<br>**Risk factors from multivariate logistic regression (a best-fitting multivariable logistic regression model was obtained through stepwise elimination): final model**<br>• Married vs. not married: OR=0.11, 95%CI:0.02-0.50<br>• Children vs. no children: OR=5.80, 95%CI:1.30-25.82<br>• Sleep problems: OR=6.15; 95%CI:2.33-16.22<br>• Pain: OR=5.56; 95%CI:2.13-14.48<br>• Obesity: OR=3.80; 95%CI:1.41-10.26<br>• Neuro-cognitive impairment: OR=2.56; 95%CI:1.02-6.38<br>• Exercise-induced symptoms: OR=2.98, 95%CI:1.11-8.02<br>**Risk factors from multivariate logistic regression: Significantly associated with fatigue (data not shown)**<br>• Not working or attending school<br>• Being married (included in final model)<br>• Having children (included in final model)<br>• Relapse<br>• Neurocognitive impairments (included in final model)<br>• Obesity (included in final model)<br>• Sleep problems (included in final model)<br>• Pain (included in final model)<br>It's unclear which other variables were included in the multivariable models but were not statistically significant.	Selection bias: 0<br>Low response rate<br>Attrition bias: 1<br>Detection bias: 0<br>Questionnaire survey, no blinding possible.<br>Confounding bias: 1<br>Multivariable analysis were used.<br>**Total quality: 2/4**

Table S11 continued

1. What is the **risk and what are risk factors for suffering from Fatigue** in CAYA survivors?				
Langeveld et al. No excess fatigue in young adult survivors of childhood cancer. 2003				
**Study Design<br>Treatment era<br>Years of follow-up<br>Fatigue measurement**	**Participants**	**Treatment**	**Main outcomes**	**Quality assessment<br>Remarks**
**Study Design:**<br>Cross-sectional study<br>**Treatment era:**<br>Not mentioned<br>**Years of follow-up:**<br>mean time since completion of therapy: 15.5 years (SD 5.9)<br>**Fatigue measurement:**<br>Multidimensional Fatigue Inventory (MFI-20). The questionnaire consist of 20 items on a five point scale. Items are combined to form five scales: general fatigue, physical fatigue, mental fatigue, reduced activity and reduced motivation. Higher scores indicate higher levels of fatigue.<br>**Country:**<br>The Netherlands	**Sample size:**<br>N=416<br>**Diagnoses:**<br>Leukaemia/non-hodgkin lymphoma without CRT: n=116 (28%)<br>Leukaemia/non-hodgkin lymphoma with CRT: n=87 (21%)<br>Solid tumor: n=183 (44%)<br>Brain/CNS tmour: n=30 (7%)<br>**Age at diagnosis:**<br>Mean age at diagnosis: 8 years (SD 4.7)<br>**Age at study:**<br>Mean age at follow-up: 24 years (SD 5.2)<br>**Controls:**<br>n=1026, recruited via survivors GPs. They were asked to help in selecting sex and age matched controls.	Chemotherapy (with or without surgery): n=197 (47%)<br>Radiotherapy (with or without surgery): n=29 (7%)<br>Combination therapy (chemotherapy and radiotherapy with or without surgery): n= 190 (46%)	**Risk:**<br>"Survivors scored significantly lower (i.e. reflecting less fatigue) for general fatigue (P <0.05, effect size -0.14) and reduced motivation (P <0.05, effect size -0.19), but statistically higher (i.e. reflecting worse fatigue) for mental fatigue (P <0.05, effect size 0.15) than controls."<br>Mean scores on the MFI-20 for General fatigue: survivors 7.5 (SD 4.3), controls 8.8 (SD 3.8), p<0.001<br>**Risk factors for fatigue from multivariable regression analysis (Full model):**<br>General fatigue:<br>**Female versus male: Beta coefficient 0.19, p<0.001**<br>Age at follow-up: Beta coefficient 0.01, NS<br>Married vs not married: Beta coefficient 0.04, NS<br>Higher education level vs lower: Beta coefficient 0.03, NS<br>Student/homemaker vs unemployed: Beta coefficient -0.12, NS<br>**Employed vs unemployed: Beta coefficient -0.20, p<0.05**<br>Age at diagnosis: Beta coefficient 0.06, NS<br>**Leukaemia/non-hodgkin lymphoma with CRT vs without CRT: Beta coefficient -0.16, p<0.05**<br>Solid tumor vs Leukaemia/NHL without CRT: Beta coefficient 0.02, NS<br>Brain/CNS tumor vs Leukaemia/NHL without CRT: Beta coefficient -0.08, NS<br>Duration of treatment: Beta coefficient 0.02, NS<br>Years since completion of therapy: Beta coefficient 0.02, NS<br>**Late effects/health problems: Beta coefficient 0.14, p<0.05**<br>Radiation therapy* vs chemotherapy*: Beta coefficient 0.01, NS<br>Combination therapy* vs chemotherapy*: Beta coefficient 0.04, NS<br>**Depression: Beta coefficient 0.54, p<0.001**	Selection bias: 0<br>Hospital based study with patients from one hospital, but not clear whether that's population based.<br>Attrition bias: 1<br>study group n=459. Included and outcome assessed n=416 (90.6%)<br>Detection bias: 0<br>Questionnaire survey, no blinding possible.<br>Confounding: 1<br>Prognostic factors are taken into account.<br>Descriptive for risk (stratified for gender and age at assessment). And included in the full model.<br>**Total quality: 2/4**<br>**Remarks:**<br>*With or without surgery

Table S11 continued

1. What is the **risk and what are risk factors for suffering from Fatigue** in CAYA survivors?				
Zebrack et al. Quality of life in childhood cancer survivors. 2002				
**Study Design**<br>**Treatment era**<br>**Years of follow-up**<br>**Fatigue measurement**	**Participants**	**Treatment**	**Main outcomes**	**Quality assessment**<br>**Remarks**
**Study Design:**<br>Cross-sectional<br>(aim to validate a QoL questionnaire in young people diagnosed with cancer in childhood)<br><br>**Treatment era:**<br>Not mentioned<br><br>**Years of follow-up:**<br>Mean years since diagnosis 13.3 (SD 5.7) range 3-27 years<br><br>**Fatigue measurement:**<br>Quality of Life-Cancer survivors. 41 item scale composed of four subscales. Each item is scored on a 0 (lowest or worst QoL) to 10 (highest or best QoL) scale.<br>Fatigue is one of the items of the 8 item physical subscale.<br><br>**Country:**<br>USA	**Sample size:**<br>n=176<br><br>**Diagnoses:**<br>Leukemia: n=53<br>Brain/CNS: n=19<br>Lymphoma: n=37<br>Wilm's Tumor: n=18<br>Sarcomas: n=28<br>Other (including neuroblastoma and retinoblastoma): n=20<br><br>**Age at diagnosis:**<br>Mean 8.5 (SD 5.1) range 0-22 years<br><br>**Age at study:**<br>Mean 21.8 (SD 3.3) range 16-28 years<br><br>**Controls:**<br>No	Not mentioned	**Risk:**<br>Mean score on the fatigue item was 7.32. It was the symptom with the lowest score in the physical subscale of the Quality of Life-Cancer Survivors. Thus indicating most problematic relative to other symptoms.<br><br>**Risk factors:**<br>No regression analyses with fatigue as outcome.	Selection bias: 0<br>original cohort n=493<br>eligible participants n= 335<br>participated/sample size n=176<br>Attrition bias: 0<br>study group n=335<br>sample size n= 176 (53%)<br>Detection bias: 0<br>Questionnaire survey, no blinding possible<br>Confounding: 0<br>For the fatigue part, only descriptive statistics were used.<br><br>**Total quality: 0/4**

Table S11 continued

1. What is the **risk and what are risk factors for suffering from Fatigue** in CAYA survivors?				
Zeltzer et al. Comparison of Psychologic outcome in adult survivors of childhood acute lymphoblastic leukemia versus sibling controls: a cooperative children's cancer group and national institutes of health study. 1997				
**Study Design**<br>**Treatment era**<br>**Years of follow-up**<br>**Fatigue measurement**	**Participants**	**Treatment**	**Main outcomes**	**Quality assessment**<br>**Remarks**
**Study Design:**<br>Cross-sectional study<br>**Treatment era:**<br>Diagnosed in 1970 or after 1970<br>**Years of follow-up:**<br>Not mentioned. In the method section it is stated that 95% of the survivors had survived for at least 5 years after diagnosis.<br>**Fatigue measurement:**<br>Profile of Mood State (POMS). 65 item self-report questionnaire to meausure six mood states, including fatigue. Individual items are scored on a scale from 0 to 4.<br>Higher scores on the fatigue subscale suggest persons with low energy.<br>**Country:**<br>USA	**Sample size:**<br>n=580<br>**Diagnoses:**<br>Acute lymphoblastic leukemia<br>**Age at diagnosis:**<br>Not mentioned.<br>**Age at study:**<br>Mean 22.6 years (SD3.2)<br>range 18.02-33.25<br>**Controls:**<br>Sibling controls: n=396	Not mentioned	**Risk:**<br>High score on the POMS indicates low energy/high fatigue<br>Fatigue mean score in survivors: 7.87 (SD 5.58); n=552<br>Fatigue mean score in controls: 8.36 (SD 5.83); n=394<br>Results of t-test (p=0.19) and regression analyses (p=0.20) showed no significant difference between survivors and controls in level of fatigue.<br>**Risk factors:**<br>No regression analyses to identify possible risk factors for fatigue subscale.	Selection bias: 1<br>Original cohort n=731<br>Participated: n=593<br>Completed both POMS and interview n=580 (79%)<br>Attrition bias: 1<br>Particpated = 593 included in analysis: n=580<br>Fatigue assessed = 552 (resp. 93% and 95%)<br>Detection bias: 0<br>Questionnaire survey, blinding not possible.<br>Confounding: 1<br>analyses for difference between survivors and controls, was controlled for age, sex, and survivor-sex interaction.<br>**Total quality: 3/4**

Table S11 continued

1. What is the **risk and what are risk factors for suffering from Fatigue** in CAYA survivors?				
Vannatta et al. A controlled study of peer relationships of children surviving brain tumors: teacher, peer, and self ratings.1998				
**Study Design**<br>**Treatment era**<br>**Years of follow-up**<br>**Fatigue measurement**	**Participants**	**Treatment**	**Main outcomes**	**Quality assessment**<br>**Remarks**
**Study Design:**<br>Case-control<br><br>**Treatment era:**<br>Not mentioned<br><br>**Years of follow-up:**<br>Average time since diagnosis: 36 months (SD 13; range 18-62 months)<br><br>**Fatigue measurement:**<br>Revised Class Play (RCP). Descriptive matching instrument on which children or teachers are asked to cast classmates into different roles. Role about fatigue is described as "someone who is tired a lot". Scores are standardized with a mean of 0 and SD of 1.<br><br>**Country:**<br>USA	**Sample size:**<br>n=28<br><br>**Diagnoses:**<br>Brain tumors:<br>Astrocytomas: n=9<br>Primitive neuroectodermal tumors: n=6<br>Oligodendrogliomas: n=5<br>Craniopharyngiomas: n=4<br>Ependymomas: n=2<br>Hypothalamic glioma: n=1<br>Brain stem glioma: n=1<br><br>**Age at diagnosis:**<br>not mentioned<br><br>**Age at study:**<br>Mean age 11.2 years (SD 2.8)<br><br>**Controls:**<br>Classroom Comparison Peers (COMP): n=28 (for each survivor a classmate is selected for comparison based on race, gender and closest in date of birth)	Surgery alone: n=14<br><br>surgery and radiotherapy: n=7<br><br>surgery, radiotherapy and chemotherapy: n=7	**Risk:**<br>RCP score for "Tired a lot" of Brain tumor survivors 0.90 (SD 1.24)<br>RCP score for "Tired a lot" of COMP -0.24 (SD 0.81)<br>This difference was statistically significant p<0.001 (two-tailed)<br><br>"For the RCP supplementary roles related to illness, fatigue, and missing school, peers nominated the children surviving brain tumors significantly<br>more often than COMP."<br><br>**Risk factors:**<br>No multivariable risk factor analysis performed.	Selection bias: 0<br>Unclear what the original cohort is.<br>Attrition bias: 1<br>Eligible: n=28<br>Sample size: n=28 (100%)<br>Detection bias: 0<br>Blinding not possible<br>Confounding: 1<br>Comparison classmate is selected based on age, race and gender<br><br>**Total quality: 2/4**<br><br>Note: Fatigue standardized score is based on peer ratings.

Table S11 continued

1. What is the **risk and what are risk factors for suffering from Fatigue** in CAYA survivors?				
Sato et al. Impact of late effects on health-related quality of life in survivors of pediatric brain tumors. 2014				
**Study Design<br>Treatment era<br>Years of follow-up<br>Fatigue measurement**	**Participants**	**Treatment**	**Main outcomes**	**Quality assessment<br>Remarks**
**Study Design:**<br>Cross-sectional design<br><br>**Treatment era:**<br>Not mentioned<br><br>**Years of follow-up:**<br>Mean time since completion of antitumor therapy 11.1 years (SD 8.3)<br><br>**Fatigue measurement:**<br>EORTC-QLQ-C30. Symptom fatigue scale (three items). Scored on 4 point likert scale. Score is linearly transformed on a 0-100 scale. Lower scores indicating beter QoL (less fatigue)<br><br>**Country:**<br>Japan	**Sample size:**<br>n=104;<br>>18 years: n=51 (see remarks)<br><br>**Diagnoses:**<br>Brain tumor:<br>Germinoma: n=23<br>Other germ cell tumor: n=5<br>Medulloblastoma/PNET: n=5<br>Low-grade glioma: n=9<br>High-grade glioma: n=4<br>Others: n=5<br><br>**Age at diagnosis:**<br>Mean 13.3 years (SD 3.5)<br><br>**Age at study:**<br>Mean 26.8 (SD7.6)<br><br>**Controls:**<br><br>No	Neurosurgery: n=47<br><br>Radiation treatment: n=44<br><br>Chemotherapy: n=34	**Risk:**<br>Mean fatigue score: 26.6 (SD 20.1)<br><br>**Risk factors for fatigue (unclear from what analysis, impact represents the extent to which each late effect influences the scores of fatigue):**<br><br>Motility disturbance of limbs: impact -5.5, p = 0.308<br><br>Seizure: impact -7.9, p = 0.158<br><br>Ocular/vision impairment: impact 5.9, p = 0.315<br><br>Endocrine abnormality: impact 12.9, p = 0.20<br><br>Higher brain dysfunction: impact 15.2, p=0.004<br><br>Analysis were adjusted for possible confounders: age, gender, age at diagnosis, hydrocephalus at diagnosis, tumor pathology, tumor location, neurosurgery, radiation treatment, chemotherapy, tumor recurrence and time since completion of antitumor therapy.<br><br>A positive impact indicates that the late effect deteriorates the aspects of HRQOL; a negative impact indicates improvement	Selection bias: 0<br>Unclear how large the original cohort was. Only eligible patients are mentioned.<br>Attrition bias: 1<br>Study group >18 years: n=66Sample size/ included >18 years: n=51 (77%)<br>Detection bias: 0<br>Questionnaire survey, blinding not possible.<br>Confounding: 1<br>Important factors were taken into account in the risk factor analysis.<br><br>**Total quality: 2/4**<br><br>Remarks: only results of the respondents aged >18 years are collected. Fatigue was not assessed in respondents aged 12-17.

Table S11 continued

1. What is the **risk and what are risk factors for suffering from Fatigue** in CAYA survivors?				
Brand et al. Screening for fatigue in adolescent and young adult pediatric brain tumor survivors: accuracy of a single-item screening measure. 2016				
**Study Design<br>Treatment era<br>Years of follow-up<br>Fatigue measurement**	**Participants**	**Treatment**	**Main outcomes**	**Quality assessment<br>Remarks**
**Study Design:**<br>Cross-sectional<br><br>**Treatment era:**<br>Not mentioned<br><br>**Years of follow-up:**<br>Mean time since diagnosis 10.55 years (SD 5.57; range 2-27 years)<br><br>**Fatigue measurement:**<br>Fatigue Thermometer (FT): Visual scale labeled from 0 (no fatigue) to 10 (worst fatigue imaginable).<br><br>Pediatric Quality of life inventory multidimensional fatigue scale (MFS) : 18 items rated on 5 point Likert scale. Higher scores indicate fewer symptoms of fatigue.<br><br>**Country:**<br>USA	**Sample size:**<br>n=142<br><br>**Diagnoses:**<br>Brain tumor:<br>Low-grade glioma: n=80<br>Embryonal tumor: n=29<br>Ependymoma: n=14<br>Craniopharyngioma: n=8<br>Germ cell: n=8<br>Choroid plexus: n=2<br>High-grade glioma: n=1<br><br>**Age at diagnosis:**<br>Mean 9.72 (SD 4.87; range 4 months-22 years)<br><br>**Age at study:**<br>Mean 20.24 (SD 4.81; range 12-32 years)<br><br>**Controls:**<br>No	Not specified	**Risk:**<br>MFS:<br>Mean total MFS score: 70.67 (SD 18.72; range 22.22-100)<br><br>Clinically significant fatigue (defined as MFS score >1 SD below the mean for normative samples): n=42 (/142=29.57%)<br><br>FT:<br>No fatigue (score 0): n=35<br>Mild fatigue (score 1-3): n=51<br>Moderate fatigue (score 4-6): n=27<br>Severe fatigue (score 7-10): n=18<br><br>**Risk factors:**<br>No multivariable risk factor analysis performed.	Selection bias: 0<br>Original cohort brain tumor survivor project REACH: n= 245<br>Eligible for this study: n=191 (77%)<br>Included: n=142 (58%)<br>Attrition bias: 1<br>Detection bias: 0<br>Questionnaire survey, blinding not possible.<br>Confounding: 1<br><br>**Total quality: 2/4**

Table S11 continued

1. What is the **risk and what are risk factors for suffering from Fatigue** in CAYA survivors?				
Adams et al. Cardiovascular Status in Long-Term Survivors of Hodgkin's Disease Treated With Chest Radiotherapy. 2004				
**Study Design<br>Treatment era<br>Years of follow-up<br>Fatigue measurement**	**Participants**	**Treatment**	**Main outcomes**	**Quality assessment<br>Remarks**
**Study Design:**<br>Cross sectional study<br>**Treatment era:**<br>After 1969<br>**Years of follow-up:**<br>Median 14.3 years since diagnosis (range 5.9-27.5)<br>**Fatigue measurement:**<br>"General health status form" designed for this study<br>**Country:**<br>USA	**Sample size:**<br>N=48<br>**Diagnoses:**<br>• Hodgkin's disease<br>**Age at diagnosis:**<br>Median 16.5 years (range 6.3-25.0)<br>**Age at study:**<br>Median 31.9 years (range 18.7-49.5)<br>**Controls:**	• Chemotherapy n=21 (43.8%)<br>• Anthracycline n=4 (8.3%)<br>• Mediastinal irradiation n=48 (100%)<br>Total mediastinal dose, including emergency dose, Gy: median 40 (range 27.0-51.7)	**Risk:**<br>• 67% [n=32 of 48] reported feeling tired/fatigued<br>• 35% [n=17 of 48] stated that it was a moderate to severe problem (≥2 on a 0 to 4 scale)<br>**Risk factors:**<br>Data on risk factors was not extracted, because no multivariable analyses were done.	Selection bias: 1<br>Yes, all patients fulfilling the inclusion criteria were contacted.<br>Attrition bias: 1<br>All participants were included in the analysis.<br>Detection bias: 0<br>Questionnaire survey, no blinding possible.<br>Confounding: 0<br>No multivariate analyses were used.<br>**Total quality: 2/4**

Table S11 continued

1. What is the **risk and what are risk factors for suffering from Fatigue** in CAYA survivors?				
Enskär et al. Prevalence of aspects of distress, coping, support and care among adolescents and young adults undergoing and being off cancer treatment. 2007				
**Study Design<br>Treatment era<br>Years of follow-up<br>Fatigue measurement**	**Participants**	**Treatment**	**Main outcomes**	**Quality assessment<br>Remarks**
**Study Design:**<br>Cross sectional study<br><br>**Treatment era:**<br>n.a.<br><br>**Years of follow-up:**<br>n.a.<br><br>**Fatigue measurement:**<br>Life Situation Scale for Adolescents (LSS-A)<br><br>**Country:**<br>Sweden	**Sample size:**<br>N=54 (n=15 on treatment; n=39 off treatment)<br><br>**Diagnoses:**<br>• Leukaemia n=18<br>• Lymphoma n=8<br>• Brain tumor n=7<br>• Sarcoma n=7<br>• Other tumors n=14<br><br>**Age at diagnosis:**<br>n.a.<br><br>**Age at study:**<br>Mean 16.0 years (SD 2.1; range 13-22)<br><br>**Controls:**<br>n.a.	n.a.	**Risk:**<br>"Fatigue was experienced by 67% of the adolescents and young adults off treatment."<br><br>**Risk factors:**<br>No risk factor analysis.	Selection bias: 0<br>Only survivors coming into hospital for FU consultations were recruited.<br>Attrition bias: 1<br>Response rate 84%<br>Detection bias: 0<br>Questionnaire survey, no blinding possible.<br>Confounding: 0<br>No multivariate analyses were used.<br><br>**Total quality: 2/4**

Table S11 continued

1. What is the **risk and what are risk factors for suffering from Fatigue** in CAYA survivors?				
Frederick et al. Fatigue in adolescent and adult survivors of non-CNS childhood cancer: a report from project REACH. 2016				
**Study Design**<br>**Treatment era**<br>**Years of follow-up**<br>**Fatigue measurement**	**Participants**	**Treatment**	**Main outcomes**	**Quality assessment**<br>**Remarks**
**Study Design:**<br>Cross sectional study<br>**Treatment era:**<br><br>**Years of follow-up:**<br>Mean time since diagnosis was 13.1 years<br>2-9 years: n=80<br>10-14 years: n=74<br>15-19 years: n=52<br>20-24 years: n=24<br>25-29 years: n=17<br>30+ years: n=21<br>**Fatigue measurement: PedsQL Multidimensional Fatigue scale (MFS)**<br>**Country:**<br>USA	**Sample size:**<br>N=268<br>**Diagnoses:**<br>• Leukemia: n=94 (35.1%)<br>• Hodgkin Lymphoma: n=41 (15.3%)<br>• Non-Hodgkin Lymphoma: n=24 (9.0%)<br>• Bone Tumors: n=25 (9.3%)<br>• Soft tissue sarcoma: n=20 (7.5%)<br>• Neuroblastoma: n=27 (10.1%)<br>• Wilms Tumor: n=20 (7.5%)<br>• Other: n=17 (6.3%)<br>**Age at diagnosis:**<br>Median age at diagnosis: 6.4 years<br>0-4 years: n=112<br>5-9 years: n=53<br>10-14 years: n=55<br>15+ years: n=46<br>**Age at study:**<br>Range 12-49 years, median age of 21.4 years<br>12-15 years: n=74<br>16-19 years: n=45<br>20-29 years: n=83<br>30-39 years: n=48<br>40-49 years: n=18	Chemotherapy:<br>Yes: n=239<br><br>Doxorubicin:<br>Yes: n=74<br><br>Any Radiation therapy:<br>Yes: n=171<br><br>CNS directed radiation therapy:<br>Yes: n=84<br><br>Surgery:<br>Yes: n=117<br><br>Bone Marrow Transplant:<br>Yes: n=33	**Risk:**<br>"Based on comparison with published data for the MSF in community samples, 37 survivors (13.8 %) were considered fatigued (MDF score ≥1 standard deviation below means for non-cancer patients of similar age) which is not statistically different from the 16 % (43 cases) that would have been expected based on community sample data [15, 16, 14] for the MFS (z= −0.727, p=0.467)."<br>**Risk factors for fatigue caseness** (20% of participants with lowest scores on the MFS) **from multivariate logistic regression analysis:**<br>Ethnicity, diagnosis, age at diagnosis, recurrence, chemotherapy, doxorubicin, any radiation therapy, CNS directed radiation therapy, surgery, bone marrow transplant were not statistically significantly associated with CRF in univariate analysis, and therefore not included in the multivariable model<br>• Gender: Female (Ref. Male) OR=1.39 (95%CI:0.69-2.81), p=0.348<br>• Age at survey: 16-19 years (Ref. 12-15 years) OR=0.27 (95%CI:0.05-1.39)<br>• Age at survey: 20-29 years (Ref. 12-15 years) OR=1.36 (95%CI:0.54-3.47)<br>• Age at survey: 30-39 years (Ref. 12-15 years) OR=2.06 (95%CI:0.58-7.27)<br>• Age at survey: 40-49 years (Ref. 12-15 years) OR=3.68 (95%CI:0.49-27.49)<br>• Household income: Less than $49,999 (Ref. $100,000 and greater) OR=1.29 (95%CI:0.52-3.19)<br>• Household income: $50-99,999 (Ref. $100,000 and greater) OR=2.16 (95%CI:0.98-4.76)<br>• Survival time: 10-14 years (Ref. 2-9 years) OR=0.83 (95%CI:0.32-2.18)<br>• Survival time: 15-19 years (Ref. 2-9 years) OR=1.33 (95%CI:0.45-3.91)<br>• Survival time: 20-24 years (Ref. 2-9 years) OR=0.55 (95%CI:0.14-2.15)<br>• Survival time: 25-29 years (Ref. 2-9 years) OR=0.34 (95%CI:0.05-2.17)<br>• Survival time: 30+ years (Ref. 2-9 years) OR=0.83 (95%CI:0.14-5.16)<br>• Chronic conditions: 1-2 (Ref. 0) OR=1.23 (95%CI:0.55-2.74)<br>• **Chronic conditions: 3 or more (Ref. 0) OR=4.27 (95%CI:1.52-11.99)**	Selection bias: 1<br>301 were eligible, 268 participated. However, participants were drawn from a larger cohort followed up in a survivorship clinic, thus probably not representative for all CCS.<br>Attrition bias: 1<br>268 evaluated<br>Detection bias: 0<br>Not applicable<br>Confounding: 1<br>Multivariable analyses performed<br>**Total quality: 3/4**

Table S11 continued

1. What is the **risk and what are risk factors for suffering from Fatigue** in CAYA survivors?				
Cheung et al. Impact of Sleep, Fatigue, and Systemic Inflammation on Neurocognitive and Behavioral Outcomes in Long-Term Survivors of Childhood Acute Lymphoblastic Leukemia. 2017				
**Study Design<br>Treatment era<br>Years of follow-up<br>Fatigue measurement**	**Participants**	**Treatment**	**Main outcomes**	**Quality assessment<br>Remarks**
**Study Design:**<br>Cross-sectional study<br>**Treatment era:**<br>2000-2010<br>**Years of follow-up:**<br>Mean years from diagnosis 7.4 years (SD 1.9)<br>**Country:**<br>USA (treated at St. Jude Children's Research Hospital, SJCRH)<br>**Fatigue measurement: PedsQL Multidimensional Fatigue Scale, domains assessed: general fatigue, sleep-rest fatigue and cognitive fatigue**	**Sample size:**<br>N = 70 (male, n = 35)<br>**Diagnoses:**<br>Childhood Acute Lymphoblastic Leukemia<br>**Age at diagnosis:**<br>Male: mean 7.0 years (SD 4.8), range 1.2-16.5 years<br>Female: mean 6.8 years (SD 4.5), range 1.9-17.7 years<br>**Age at study:**<br>Male: mean 14.8 years (SD 5.1), range 8.2-25.5 years<br>Female: mean 13.9 years (SD 4.3), range 8.1-25.4 years<br>**Controls:**<br>No age-matched healthy comparison control group	All treated with chemotherapy only	**Risk:**<br>Survivors self-reported more behavioral problems and greater fatigue compared with the general population (Supporting Information 3).<br>Cognitive fatigue:<br>All survivors mean -0.75 (SD 1.2) vs. expected population value (mean=0, SD=1), p=0.0003<br>Male mean -0.64 (SD 1.1) vs. female mean -0.85 (SD 1.3), p=0.61<br>General fatigue:<br>All survivors mean -0.61 (SD 1.2) vs. expected population value (mean=0, SD=1), p=0.0003<br>Male mean -0.30 (SD .9) vs. female mean -0.88 (SD 1.4), p=0.19<br>Sleep-rest fatigue:<br>All survivors mean -0.27 (SD 1.2) vs. expected population value (mean=0, SD=1), p=0.07<br>Male mean 0.16 (SD 1.0) vs. female mean -0.64 (SD 1.2), p=0.04<br><br>**Risk factors:**<br>No risk factors for CRF were analyzed.	Selection bias: 1<br>- Recruited from SJCRH (treatment)<br>- Response rate overall: 83%<br>Attrition bias: 1<br>- Complete: 70<br>Detection bias: 0<br>Not possible, questionnaire study<br>Confounding: 0<br>- Fatigue as a predictor, not as an outcome<br><br>**Total quality: 2/4**<br><br>Remarks:<br>Neurocognitive testing, behavioral ratings, self-reported symptoms of fatigue, parent-reported (8-12 years) or self-reported (13-21 years) sleep measures, and serum collection (5 mL of blood)

Table S11 continued

1. What is the **risk and what are risk factors for suffering from Fatigue** in CAYA survivors?				
Nies et al. Long-Term Quality of Life in Adult Survivors of Pediatric Differentiated Thyroid Carcinoma. 2017				
**Study Design**<br>**Treatment era**<br>**Years of follow-up**<br>**Fatigue measurement**	**Participants**	**Treatment**	**Main outcomes**	**Quality assessment**<br>**Remarks**
**Study Design:**<br>Cross-sectional study<br><br>**Treatment era:**<br>1970-2013<br><br>**Years of follow-up:**<br>Median 17.8 years, range 5-44.7 years)<br><br>**Country:**<br>The Netherlands<br><br>**Fatigue measurement:**<br>Multidimensional Fatigue Inventory-20 (MFI-20)	**Sample size:**<br>N = 67 (males n = 9)<br><br>**Diagnoses:**<br>Pediatric Differentiated Thyroid Carcinoma (DTC)<br><br>**Age at diagnosis:**<br>Median 15.8 years, range 7.9-18.8 years<br><br>**Age at study:**<br>Median 34.2 years, range 18.8-61.7 years<br><br>**Controls:**<br>Peers without a medical history of malignancy approached by participants (+/- 5 years)<br>N = 56 (males n = 7)<br>Median age at evaluation 34.0 years, range 19.4-60.2 years	All survivors underwent a total thyroidectomy and 131-I was administered to 97.0%.	**Risk:**<br>Mental fatigue scores were significantly higher in survivors (p=0.012; higher scores represent more fatigue). Scores from the other MFI-20 subscales did not differ significantly between survivors and controls.<br><br>Survivors vs. controls (median (25th percentile, 75th percentile)<br>General fatigue: survivors 10 (8, 15) vs. controls 9 (5, 12), p=0.075<br>Physical fatigue: survivors 8 (5, 12) vs. controls 6 (4, 10), p=0.083<br>Reduced activity: survivors 8 (5, 11) vs. controls 8 (5, 11), p=0.613<br>Reduced motivation: survivors 6 (4, 9) vs. controls 6 (4, 9), p=0.879<br>Mental fatigue: survivors 9 (5, 15) vs. controls 7 (4, 10), p=0.012<br>Total: survivors 41 (31, 57) vs. controls 36 (27, 54), p=0.129<br><br>**Risk factors:**<br>No multivariable risk factor analyses for fatigue	Selection bias:1<br>- Recruited from nationwide follow-up study<br>- Response rate overall: 89.3%<br>Attrition bias: 1<br>- Included: n=67<br>Detection bias: 0<br>Not possible, questionnaire study<br>Confounding: 0<br>- No multivariable analyses<br><br>**Total quality: 2/4**<br><br>Remarks:<br>- No multivariable analysis<br>- Survivors of the nationwide study and participating survivors differed in age at evaluation (median 19.1 vs. 34.2 years, p<0.001) and follow-up duration (median 2.8 vs. 17.8 years, p<0.001)

Table S11 continued

1. What is the **risk and what are risk factors for suffering from Fatigue** in CAYA survivors?				
Rach et al. Predictors of fatigue and poor sleep in adult survivors of childhood Hodgkin's lymphoma: a report from the Childhood Cancer Survivor Study. 2017				
**Study Design**<br>**Treatment era**<br>**Years of follow-up**<br>**Fatigue measurement**	**Participants**	**Treatment**	**Main outcomes**	**Quality assessment**<br>**Remarks**
**Study Design:**<br>Baseline (2000-2002) and follow-up (2003-2007) questionnaire<br>**Treatment era:**<br>n.a.<br>**Years of follow-up:**<br>>5 years<br>**Country:**<br>USA<br>**Fatigue measurement:**<br>The Functional Assessment of Chronic Illness Therapy- Fatigue (FACIT-F)<br>Survivors with a total score of ≤30 were classified as having clinically significant fatigue.	**Sample size:**<br>N = 751 (male n = 372)<br>**Diagnoses:**<br>Pediatric Hodgkin's lymphoma (HL)<br>**Age at diagnosis:**<br>0-10: n=150 (20%)<br>11-15: n=319 (42.5%)<br>16-20: n=282 (37.5%)<br>**Age at study:**<br>Follow-up survey age<br>18-29: n=53 (7.1%)<br>30-34: n=154 (20.5)<br>≥35: n=544 (72.4%)<br>**Controls:**<br>No healthy comparison control group.<br>Comparisons have only been made between HL survivors with clinical elevations of fatigue and sleep problems and HL survivors without elevated fatigue.	Radiation therapy<br>- Chest RT<30Gy (n=230, 30.6%)<br>- Chest RT≥30Gy (n=445, 59.3%)<br>Chemotherapy (patients may receive multi chemotherapy so the percentage exceeds 100)<br>- Anthracycline (n=158, 21%)<br>- Alkylating agents (n=419, 55.8%)<br>- Bleomycin (n=147, 19.6%)<br>- Vinca alkaloids and heavy metals (n=418, 55.7%)<br>- None (n=326, 43.4%)	**Risk:**<br>The proportion of survivors endorsing elevated fatigue was 17%.<br>**Risk factors from multivariable logistic regression analysis:**<br>• Sex: Female (Ref. Male) OR=4.75 (95%CI:2.47-9.15, p<0.001)<br>• Emotional distress: Impaired (Ref. not impaired) OR=8.38 (95%CI:4.28-16.42, p<0.001)<br>• Work status: Unemployed (Ref. employed) OR=2.90 (95%CI:1.27-6.62, p<0.01)<br>• Body pain: Impaired (Ref. not impaired) OR=3.73 (95%CI:2.09-6.67, p<0.001)<br>• Physical function: Impaired (Ref. not impaired) OR=3.28 (95%CI:1.75-6.15, p<0.001)<br>• BMI: Overweight (Ref. Normal) OR=0.95 (95%CI:0.50-1.79, n.s.)<br>• BMI: Obese (Ref. Normal) OR=1.06 (95%CI:0.52-2.15, n.s.)	Selection bias: 1<br>- Survivors of HL randomly selected from Childhood Cancer Survivor Study (CCSS)<br>- Response rate overall: 79%<br>Attrition bias: 1<br>- Complete: 751<br>Detection bias: 0<br>Not possible, questionnaire study<br>Confounding: 1<br>- Multivariable logistic regression analyses investigated the demographic, psychological, and physical variables<br><br>**Total quality: 3/4**

Table S11 continued

1. What is the **risk and what are risk factors for suffering from Fatigue** in CAYA survivors?				
Arpaci & Kilicarslan Toruner. Assessment of problems and symptoms in survivors of childhood acute lymphoblastic leukaemia. 2016				
**Study Design**<br>**Treatment era**<br>**Years of follow-up**<br>**Fatigue measurement**	**Participants**	**Treatment**	**Main outcomes**	**Quality assessment**<br>**Remarks**
**Study Design:**<br>Cross-sectional questionnaire<br><br>**Treatment era:**<br>n.a.<br><br>**Years of follow-up:**<br>Mean = 2.55 years (SD 1.19), range 1-5 years<br><br>**Country:**<br>Turkey<br><br>**Fatigue measurement:**<br>Collection form developed by the researchers	**Sample size:**<br>N = 91<br><br>**Diagnoses:**<br>Acute lymphoblastic leukaemia<br><br>**Age at diagnosis:**<br>Mean = 6.38 years (SD 3.84), range 1-14 years<br><br>**Age at study:**<br>Mean = 11.66 years (SD 4.17), range 5-23 years<br><br>**Controls:**<br>No control group	Chemotherapy (CT): n=50 (54.9%)<br><br>Chemotherapy and radiotherapy (CT+RT): n=37 (40.7%)<br><br>Haematopoietic stem cell transplantation (HSCT): n=4 (4.4%)	**Risk:**<br>In total 29.7% (n=27) had fatigue<br><br>**Risk factors:**<br>No risk factor analyses for fatigue.	Selection bias: 1<br>- Recruited from three hospitals located in Ankara<br>- Response rate overall: 95%<br><br>Attrition bias: 1<br>- Included: n=91<br><br>Detection bias: 0<br>Not possible, questionnaire study<br><br>Confounding: 0<br>Multivariable analyses have not been performed<br><br>**Total quality: 2/4**<br><br>Remarks:<br>The variables were investigated using the Mann–Whitney U and chi-square test.

Table S11 continued

1. What is the **risk and what are risk factors for suffering from Fatigue** in CAYA survivors?				
Graef et al. Sleepiness, Fatigue, Behavioral Functioning, and Quality of Life in Survivors of Childhood Hematopoietic Stem Cell Transplant. 2016				
**Study Design<br>Treatment era<br>Years of follow-up<br>Fatigue measurement**	**Participants**	**Treatment**	**Main outcomes**	**Quality assessment<br>Remarks**
**Study Design:**<br>Cross-sectional study<br><br>**Treatment era:**<br>n.a.<br><br>**Years of follow-up:**<br>Mean 7.76 years (SD 1.87), range 5-14 years post-HSCT<br><br>**Country:**<br>USA<br><br>**Fatigue measurement:**<br>Pediatric Quality of Life Inventory Multidimentional Fatigue Scale (PedsQL MFS)	**Sample size:**<br>N = 76 (males n=45)<br><br>**Diagnoses:**<br>Acute myeloid leukemia (n=31, 40.8%)<br>Acute lymphoblastic leukemia (n=22, 29.0%)<br>Severe aplastic anemia or other conditions requiring HSCT (n=16, 21.0%)<br>Chronic myeloid leukemia (n=7, 9.2%)<br><br>**Age at diagnosis:**<br><22 years of age at the time of transplant<br><br>**Age at study:**<br>Mean = 17.84 years (SD 6.04), range 8-29 years<br>• Child (<13 years): n=18 (23.68%)<br>• Adolescent (13-18 years): n=24 (31.58%)<br>• Young adult (>18 years): n=34 (44.74)<br><br>**Controls:**<br>No control group	Pediatric hematopoietic stem cell transplant (HSCT)	**Risk:**<br>Mean levels of fatigue were 69.21 (SD 20.14) for self-report (n=65) and 72.15 (SD 20.79) by parent report (n=38), indicating moderately elevated fatigue symptoms (scores range from 0 to 100, with higher scores indicating less fatigue).<br>Compared to ratings described in another study*, ratings of total fatigue in survivors of this study indicated more fatigue than in healthy peers (p<0.001), but no difference compared to children on and off treatment for cancer (p>0.05).<br><br>**Risk factors:**<br>No multivariable risk factor analyses for fatigue.<br><br>* Varni, J. W., Burwinkle, T. M., Katz, E. R., Meeske, K., & Dickinson, P. (2002). The PedsQL in pediatric cancer: Reliability and validity of the Pediatric Quality of Life Inventory Generic Core Scales, Multidimensional Fatigue Scale, and Cancer Module. Cancer, 94, 2090–2106.	Selection bias: 1<br>- Recruited from St. Jude Children's Research Hospital (sample representative of those who receive allogeneic transplant in that hospital)<br>- Response rate overall: 78.4%<br>Attrition bias: 1<br>- Included (self-report): n=76<br>Detection bias: 0<br>Not possible, questionnaire study<br>Confounding: 0<br>Fatigue as a predictor, not as an outcome<br><br>**Total quality: 2/4**<br><br>Remarks:<br>Self-report measures were completed for patients >18 years of age, and both self-and parent-proxy measures were completed for patients 8-18 years. Clinical information was obtained from electronic medical records.

Table S11 continued

1. What is the **risk and what are risk factors for suffering from Fatigue** in CAYA survivors?				
Lowe et al. Distinct health behavior and psychosocial profiles of young adult survivors of childhood cancers: a mixed methods study. 2016				
**Study Design**<br>**Treatment era**<br>**Years of follow-up**<br>**Fatigue measurement**	**Participants**	**Treatment**	**Main outcomes**	**Quality assessment**<br>**Remarks**
**Study Design:**<br>Mixed methods: Mail-based survey and semi-structured interviews<br><br>**Treatment era:**<br>n.a.<br><br>**Years of follow-up:**<br>Average time since diagnosis: 8.42 years (SD 5.73)<br><br>**Country:**<br>USA<br><br>**Fatigue measurement:**<br>Profile of Mood States (POMS)	**Sample size:**<br>N = 104 (male: n=53)<br><br>**Diagnoses:**<br>• Hodgkin's lymphoma: n=24, 23.1%<br>• Non-Hodgkin's lymphoma: n=9, 8.7%<br>• Burkitt's lymphoma: n=4, 3.8%<br>• Acute lymphoblastic leukemia: n=17, 16.3%<br>• Acute myelogenous leukemia: n=3, 2.9%<br>• Blastoma: n=6, 5.8%<br>• Sarcoma: n=11, 10.6%<br>• Thyroid cancer: n=10, 9.6%<br>• Other: n=20, 19.2%<br><br>**Age at diagnosis:**<br><18 years<br><br>**Age at study:**<br>Mean 22.13 years (SD 3.18)<br><br>**Controls:**<br>No control group, only comparisons among risk clusters were made	• Chemotherapy: n=86, 82.7%<br>• Surgery: n=81, 77.9%<br>• Radiation: n=58, 55.8%	**Risk:**<br>POMS, fatigue-inertia: mean 8.13 (SD 5.99)<br><br>**Risk factors:**<br>No systematic risk factor analyses.	Selection bias: 0<br>- Recruitment limited to survivors whose current address and telephone number were available<br>- Response rate overall: 55.5%<br>Attrition bias: 1<br>- Included: n=104 (98%)<br>Detection bias: 0<br>Not possible, questionnaire study<br>Confounding: 0<br>Multivariable analyses were not performed<br>**Total quality: 1/4**<br><br>Remarks:<br>Only data concerning the quantitative study are reported here

Table S11 continued

1. What is the **risk and what are risk factors for suffering from Fatigue** in CAYA survivors?				
Fortmann et al. Sleep Quality, Fatigue, and Quality of Life Among Teenage and Young Adult Cancer Survivors. 2018				
**Study Design<br>Treatment era<br>Years of follow-up<br>Fatigue measurement**	**Participants**	**Treatment**	**Main outcomes**	**Quality assessment<br>Remarks**
**Study Design:**<br>Cross-sectional survey study<br><br>**Treatment era:**<br>n.a.<br><br>**Years of follow-up:**<br>Time since active treatment in the off-treatment group:<br>≤3 months: 14%<br>4-11 months: 20%<br>1-5 years: 45.2%<br>>5 years: 3.7%<br>On active surveillance: 12.6%<br><br>**Country:**<br>United Kingdom<br><br>**Fatigue measurement:**<br>13-item fatigue subscale of the Functional Assessment of Chronic Illness Therapy Fatigue (FACIT-F)<br>Scores above 22 were considered as<br>clinically significant fatigue*.<br><br>* Reeves WC, Lloyd A, Vernon SD, et al. Identification of ambiguities in the 1994 chronic fatigue syndrome research case definition and recommendations for resolution. BMC Health Serv Res. 2003;3(1):25.	**Sample size:**<br>N = 202 (male: n=71; on treatment: n=67, off treatment: n=135, n=8: treatment status not known)<br><br>**Diagnoses:**<br>Leukemia n=55<br>Lymphoma n=66<br>Bone tumor n=16<br>Soft tissue tumor n=15<br>Carcinoma n=6<br>Germ cell tumor n=5<br>CNS tumor n=3<br>Melanoma n=2<br>Other n=31<br><br>**Age at diagnosis:**<br>On treatment: mean 17.8 years (SD 3.3)<br>Off treatment: mean 16.3 years (SD 4.3)<br><br>**Age at study:**<br>13-24 years at study (inclusion criterion)<br>On treatment: mean 19.6 years (SD 3.1)<br>Off treatment: mean 20.2 years (SD 2.9)<br><br>**Controls:**<br>n.a.	Chemotherapy or radiotherapy<br>• On treatment: n=64 (95.52%)<br>• Off treatment: n=128 (94.81%)<br><br>No chemotherapy or radiotherapy<br>• On treatment: n=3 (4.48%)<br>• Off treatment: n=7 (5.19%)	**Risk:**<br>Mean fatigue score in off-treatment TYA survivors was 15.56 (SD=10.98)<br>26.67% of TYAs off treatment reported clinically significant levels of fatigue.<br><br>**Risk factors:**<br>No systematic risk factor analyses for fatigue.	Selection bias: 0<br>- TYA were recruited regardless of their date of diagnosis and treatment status<br>- Response rate overall: n.a. (number of eligible participants not specified)<br>Attrition bias: 1<br>- Incomplete responses were excluded<br>Detection bias: 0<br>Not possible, questionnaire study<br>Confounding: 1<br>Even though fatigue was an independent variable in the analysis, they controlled for age at survey, age at diagnosis, gender and ethnicity<br><br>**Total quality: 2/4**

Table S11 continued

1. What is the **risk and what are risk factors for suffering from Fatigue** in CAYA survivors?				
Spathis et al. Cancer-Related Fatigue in Adolescents and Young Adults After Cancer Treatment: Persistent and Poorly Managed. 2017				
**Study Design**<br>**Treatment era**<br>**Years of follow-up**<br>**Fatigue measurement**	**Participants**	**Treatment**	**Main outcomes**	**Quality assessment**<br>**Remarks**
**Study Design:**<br>Cross-sectional study<br><br>**Treatment era:**<br>n.a.<br><br>**Years of follow-up:**<br>Months since diagnosis: mean 31 months (inter-quartile range (IQR) 18-49 mths)<br>Months since last treatment: mean 18 months (IQR 10-32 mths)<br><br>**Country:**<br>United Kingdom<br><br>**Fatigue measurement:**<br>PedsQL Multidimensional Fatigue Scale (MFS)	**Sample size:**<br>N = 80 (male: n=26)<br><br>**Diagnoses:**<br>Leukemia: n=20 (25%)<br>Lymphoma: n=35 (44%)<br>Osteosarcoma/Ewing's: n=6 (8%)<br>Brain neoplasm: n=1 (1%)<br>Other: n=18 (23%)<br><br>**Age at diagnosis:**<br>Mean 18.9 years (SD 3.1) range 12-24 years<br><br>**Age at study:**<br>Mean 22.1 years (SD 2.7) range 17-27 years<br><br>**Controls:**<br>n.a.	n.a.	**Risk:**<br>68 respondents (85%) experienced fatigue during the preceding month. The mean fatigue severity of the *fatigued participants* was 44.3 (SD=20.5).<br><br>Fatigue severity was worse more than 1 year after cancer treatment (M=39, SD=19.7) compared to <1 year (M=53.8, SD=19.7; independent samples t-test, t(56)=2.8, p=0.007).<br><br>Fatigue was worse in females (M=39.6, SD=19.3) than males (M=55.6, SD=19.6; t(66)=3.1, p=0.003), but was not associated with other demographic variables, including cancer type or treatment duration.<br><br>**Risk factors:**<br>No systematic multivariable risk factor analyses for fatigue.	Selection bias: 0<br>- Recruited from three teenage and young adult principal treatment centers (TYA PTCs) in the UK<br>- Response rate overall: 41%<br>Attrition bias: 1<br>- Included: n=80<br>Detection bias: 0<br>Not possible, questionnaire study<br>Confounding: 0<br><br>**Total quality: 1/4**<br><br>Remarks:<br>Demographic data were collected for both respondents and non-respondents. Only data related to respondents was reported.

Table S11 continued

1. What is the **risk and what are risk factors for suffering from Fatigue** in CAYA survivors?				
Johannsdottir et al. Adverse Health Outcomes and Associations with Self-Reported General Health in Childhood Lymphoma Survivors. 2017				
**Study Design<br>Treatment era<br>Years of follow-up<br>Fatigue measurement**	**Participants**	**Treatment**	**Main outcomes**	**Quality assessment<br>Remarks**
**Study Design:**<br>Cross-sectional study with clinical examinations<br><br>**Treatment era:**<br>1970-2000<br><br>**Years of follow-up:**<br>Median 20 years (range: 7-37 years)<br><br>**Country:**<br>**Norway**<br><br>**Fatigue measurement:**<br>**Fatigue Questionnaire (FQ)**	**Sample size:**<br>N = 124 (male: n=58)<br><br>**Diagnoses:**<br>Non-Hodgkin lymphoma (n=43)<br>Hodgkin lymphoma (n=81)<br><br>**Age at diagnosis:**<br>Median 15 years (range: 2-18 years)<br><br>**Age at study:**<br>Median 33 years (range: 19-54 years)<br><br>**Controls:**<br>General health was compared with 478 individuals from the Norwegian general population, aged 30-39 years	Chemotherapy only (n=38)<br><br>Radiotherapy only (n=14)<br><br>Chemotherapy and radiotherapy (n=72)<br><br>10 participants received also stem cell transplantation in combination with total body radiation (n=5) or chemotherapy as conditioning regimen (n=5)	**Risk:**<br><br>Grade 0: Asymptomatic (no) fatigue; n=86 (/124=69.4%)<br><br>Grade 2: Chronic fatigue (i.e. substantial fatigue (≥4; with duration of at least 6 months); n=38 (/124=30.6%)<br><br>**Risk factors:**<br><br>No systematic risk factor analyses for fatigue.	Selection bias: 1<br>- Identified through the Norwegian cancer registry<br>- Response rate overall: 56%<br>Attrition bias: 1<br>- Complete questionnaires: 124<br>Detection bias: 0<br>Not possible, questionnaire study<br>Confounding: 0<br>No multivariable analysis<br><br>**Total quality: 2/4**<br><br>Remarks:<br>Psychosocial adverse health outcomes (AHOs) were assessed by the survivor's competition of validated instruments as the Hospital Anxiety and Depression Scale (HADS) and the fatigue questionnaire

Table S11 continued

1. What is the **risk and what are risk factors for suffering from Fatigue** in CAYA survivors?				
Karimi et al. Fatigue, Physical and Functional Mobility, and Obesity in Pediatric Cancer Survivors. 2019				
**Study Design<br>Treatment era<br>Years of follow-up<br>Fatigue measurement**	**Participants**	**Treatment**	**Main outcomes**	**Quality assessment<br>Remarks**
**Study Design:**<br>Cross-sectional study<br><br>**Treatment era:**<br>n.a.<br><br>**Years of follow-up:**<br>Time since diagnosis mean 5.9 years (SD 4.5)<br><br>**Country:**<br>USA<br><br>**Fatigue measurement:**<br>PROMIS V1.0 Pediatric Profile 25: among others 4 items on fatigue, 5-point likert scale (0-4), higher scores represent higher levels of fatigue. Scored by summing items, possible range of 0-16.	**Sample size:**<br>N=144<br><br>**Diagnoses:**<br>ALL/AML n=64 (44.5%)<br>Brain tumor n=23 (16.0%)<br>Lymphoma n=13 (9.0%)<br>Solid tumor n=38 (26.4%)<br>Neurocutaneous syndrome n=2 (1.4%)<br>Other n=4 (2.8%)<br><br>**Age at diagnosis:**<br>n.a.<br><br>**Age at study:**<br>Mean 12.9 years (SD 3.0)<br><br>**Controls:**<br>No control group.	Bone marrow transplant n=7 (4.9%)<br><br>Stem cell transplant:<br>Allotransplantation n=6 (4.2%)<br>Autotransplantation n=5 (3.4%)<br><br>Chemotherapy n=135 (93.8%)<br><br>Radiation therapy n=50 (34.7%)<br><br>Surgery n=90 (62.5%)	**Risk:**<br>Children reported normal levels of fatigue (mean 4.1 (SD 4.0); range 0-16).<br>22 children (/144=15.3%) reported elevated levels of fatigue*<br><br>**Risk factors for fatigue from hierarchical linear regression**<br>(adjusted for age, sex, race, time since diagnosis, diagnosis, chemotherapy, radiation, depression, parent reported depression/anxiety, BMI, physical and function mobility):<br><br>Shorter time since diagnosis, more depression symptoms, and more difficulty with mobility predicted higher levels of fatigue.<br>• Age at survey: β=-0.005, p=0.935<br>• Gender**: β=0.008, p=0.895<br>• **Race**: β=-0.123, p=0.047**<br>• **Time since diagnosis: β=-0.154, p=0.019**<br>• Diagnosis**: β=-0.045, p=0.464<br>• Chemotherapy: β=0.097, p=0.121<br>• Radiation: β=-0.030, p=0.625<br>• **Depression: β=0.396, p<0.001**<br>• Parent-reported depression/anxiety: β=0.117, p=0.095<br>• BMI: β=-0.036, p=0.560<br>• **Physical and function mobility: β=-0.427, p<0.001**	Selection bias: 1<br>Review of medical charts, ≥80% gave consent<br><br>Attrition bias: 1<br>All analyzed<br><br>Detection bias: 0<br>No blinding possible<br><br>Confounding: 1<br>Analyses adjusted for important confounders<br><br>**Total quality: 3/4**<br><br>Remarks:<br>*definition of fatigue caseness is not clear from the manuscript<br>** reference categories not specified, variable therefore not included in the overall conclusions

Table S11 continued

4. Does the risk of developing Fatigue change over time in CAYA survivors?				
Macpherson et al. Exercise and Fatigue in Adolescent and Young Adult Survivors of Hodgkin Lymphoma: A Report from the Children's Oncology Group. 2015				
**Study Design<br>Treatment era<br>Years of follow-up<br>Fatigue measurement**	**Participants**	**Treatment**	**Main outcomes**	**Quality assessment<br>Remarks**
**Study Design:**<br>Retrospective cohort study with data from a RCT<br>**Treatment era:**<br>Not available<br>**Years of follow-up:**<br>End of therapy, 12 and 36 months post-therapy measurements.<br>**Fatigue measurement:**<br>No standardized measurement<br>**Country:**<br>USA	**Sample size:**<br>N=103<br>**Diagnoses:**<br>• Hodgkin Lymphoma<br>**Age at diagnosis:**<br>Mean age at dx: 15.46 years (13-21 years)<br>**Age at study:**<br>Not available<br>**Controls:**<br>No controls.	Protocol treatment arm:<br>Rapid early responders:<br>**Rapid early responders:**<br>• ABVE-PC x 4, <CR, IFRT n=47 (45.6%)<br>• ABVE-PC x 4, CR, IFRT n=15 (14.6%)<br>• ABVE-PC x 4, CR, NO IFRT n=26 (25.2%)<br>**Slow early responders:**<br>• ABVE-PC x 4 + IFRT + DECA x 2 n= 10 (9.7%)<br>• ABVE-PC x 4 + IFRT n=5 (4.9%)	Five items (Scale 0 "very much so" to 4 "not at all"), measured at end of therapy, 12 months post-therapy and 36 months post-therapy:<br>"Felt tired": No significant changes at 12-month or 36-month assessment compared to baseline<br>"Had trouble finishing tasks because tired quickly": No significant changes at 12-month or 36-month assessment compared to baseline<br>"Needed to sleep during the day": No significant changes at 12-month or 36-month assessment compared to baseline<br>"Frustrated by being too tired to do things he/she wanted to do": No significant changes at 12-month or 36-month assessment compared to baseline<br>"Needed to limit social activities because of fatigue": Slight improvement at 12-month assessment ($p<0.045$), but no significant change at 36-month assessment compared to baseline.	Selection bias: 0<br>Secondary analysis of data collected as a randomized controlled trial. There's no information on how the randomization was done. One inclusion criterion is "completed a self-report survey at end of treatment, 12 and 36 months" → then it's rather not representative<br>Attrition bias: 1<br>N=93/103 responded fatigue questions at 36 months → 90.3%<br>Detection bias: 0<br>Questionnaire survey, no blinding possible.<br>Confounding: 1<br>Multivariable logistic regression was used to evaluate association with exercise.<br>**Total quality 2/4**

Table S11 continued

4. Does the risk of developing Fatigue change over time in CAYA survivors?				
Zeller et al. Chronic Fatigue in Long-term Survivors of Childhood Lymphomas and Leukemia: Persistence and Associated Clinical Factors. 2014				
**Study Design<br>Treatment era<br>Years of follow-up<br>Fatigue measurement**	**Participants**	**Treatment**	**Main outcomes**	**Quality assessment<br>Remarks**
**Study Design:**<br>Case-control study<br>**Treatment era:**<br>1970-2002<br>**Years of follow-up:**<br>Median 25.3 years (range 11.3-39.9)<br>**Fatigue measurement:**<br>**Fatigue Questionnaire (FQ)**<br>**Country:**<br>Norway	**Sample size:**<br>Total n=62/102<br>**Diagnoses:**<br>• Lymphoma n=33<br>• Acute lymphoblastic leukemia (ALL) n=29<br>**Age at diagnosis:**<br>Not mentioned.<br>**Age at study:**<br>Mean 34.05 years<br>**Years of follow-up:**<br>Mean 23.5 years<br><br>Controls did not differ from “cases” (with chronic fatigue (CF)) in sex, age at study, diagnosis, therapy, follow-up time	Radiation therapy:<br>• CF: 43%<br>• Controls: 57%<br>Cum. Anthracycline dose (mg):<br>• CF: mean 166.2 (SD 139.9)<br>• Controls: 170.0 (SD 127.6)	1. Fatigue assessment: 79/290 (27.2%) survivors were fatigued [≥5 years since diagnosis]<br>2. Fatigue assessment at a median of 2.7 years later (1-4.3 years) [mean follow-up time of 23-24 years]: case-control study (no prevalence measure possible).<br>**Persistent fatigue:**<br>32 of 53 former CF cases (60.4%) were still fatigued.<br>**Persistently non-fatigued:**<br>40 of 49 former non-CF cases (81.6%) were still not fatigued<br>**Converters:**<br>21 of 53 former CF cases (39.6%) were no longer fatigued<br>9 of 49 former non-CF cases (18.4%) were now fatigued	Selection bias: 0<br>Original cohort was 430 survivors, only 102 were included for this study.<br>Attrition bias: 0<br>62/102 were analyzed.<br>Detection bias: 0<br>Questionnaire survey, no blinding possible.<br>Confounding: 1<br>Multivariate statistics were used.<br>**Total quality: 1/4**

Table S11 continued

**5. What is the most reliable and valid diagnostic tool to diagnose Fatigue in CAYA survivors?**				
Ho et al. Psychometric properties of the Chinese version of the fatigue scale-adolescent. 2015				
**Study Design<br>Treatment era<br>Years of follow-up<br>Diagnostic tool**	**Participants**	**Diagnostic tool**	**Main outcomes**	**Quality assessment<br>Remarks**
**Study Design:**<br>Cross-sectional study<br><br>**Treatment era:**<br>n.a.<br><br>**Years of follow-up:**<br>62% (n=124) have ≥25 months since treatment completed; n=37 (18.5%) 13-24 months; n=39 (19.5%) 6-12 months.<br><br>**Fatigue measurement: Fatigue-scale adolescent (FS-A)**<br><br>**Country:**<br>Hong Kong, China	**Sample size:**<br>N=200 adolescent cancer survivors (ACS)<br>N=50 adolescent cancer patients (ACP)<br><br>**Diagnoses:**<br>• Leukemia n=91 (45.5%)<br>• Lymphoma n=57 (28.5%)<br>• Brain tumor n=33 (16.5%)<br>• Osteosarcoma n=9 (4.5%)<br>• Kidney tumor n=4 (2.0%)<br>• Germ-cell tumor n=6 (3.0%)<br><br>**Age at diagnosis:**<br>Not available<br><br>**Age at study:**<br>N=200 CCS: 13-14 years: n=48 (24%)<br>15-16 years: n=70 (35%)<br>17-18 years: n=82 (41%)<br><br>N=50 ACP: 13-14 years: n=13 (26%)<br>15-16 years: n=18 (36%)<br>17-18 years: n=19 (38%)<br><br>**Controls:**<br>N=50 healthy controls (HC; age at study): 13-14 years: n=15 (30%)<br>15-16 years: n=18 (36%)<br>17-18 years: n=17 (34%)	Chinese version of the Fatigue Scale for Adolescents (FS-A)<br><br>**Cases of Fatigue:**<br>Levels of fatigue<br>ACS:28.6 (SD 3.7)<br>ACP: 31.3 (SD 5.2)<br>HC: 22.1 (SD 4.8)	**Reliability:**<br>"The test-retest reliability coefficient of the Chinese version of the FS-A at a 2-week interval was 0.85 (ICCvalue), indicating a reliability of 0.80 or higher, which is acceptable for an instrument to be used in research."<br>After deletion of items 6 and 10, Cronbach's alpha was 0.89.<br><br>**Validity:**<br>- Semantic equivalence was high (94%).<br>- Content validity index was 82%, after omission of items 6 and 10 even higher (92%), indicating good content validity.<br>- The known-groups validity (ACS, ACP, HC) was supported, mean FS-A score of the ACS was significantly lower than that of the ACP, but significantly higher than that of the HC.<br>- The discriminant validity of the FS-A was supported: There was a strong positive correlation between scores on the FS-A and CES-DC (r=0.53, n=200, P<0.01), indicating that adolescents with higher levels of fatigue were associated with more depressive symptoms. In addition, there was a strong negative correlation between scores on the FS-A and PedsQL (r=−0.58, n=200, P<0.01), indicating that higher levels of fatigue were to be associated with lower quality of life.	Selection bias: 0<br>Convenience sample of 200 survivors.<br>Attrition bias: 1<br>All answered the fatigue questionnaire.<br>Detection bias: 0<br>Questionnaire survey, no blinding possible.<br>Confounding: 0<br>Multivariable analysis were not used.<br>**Total quality: 1/4**

Table S11 continued

5. What is the most reliable and valid diagnostic tool to diagnose Fatigue in CAYA survivors?				
Nascimento et al. High validity and reliability of the PedsQL Multidimensional Fatigue Scale for Brazilian children with cancer. 2015				
**Study Design<br>Treatment era<br>Years of follow-up<br>Diagnostic tool**	**Participants**	**Diagnostic tool**	**Main outcomes**	**Quality assessment<br>Remarks**
**Study Design:**<br>Cross-sectional study<br><br>**Treatment era:**<br>n.a.<br><br>**Years of follow-up:**<br>any stage (outpatient, hospitalized, palliative care); >50% still in active treatment<br><br>**Country:**<br>Brazil	**Sample size:**<br>N=42 children (8-12 years)<br>N=68 teenagers (13-17 years)<br>N=106 caregivers<br><br>**Diagnoses:**<br>Leukemias and lymphomas 45.9%<br>CNS tumor 21.6%<br>Sarcomas 14.4%<br>Other 18%<br><br>**Age at diagnosis:**<br>n.a.<br><br>**Age at study:**<br>See above<br><br>**Treatment:**<br>Chemo 39.6%<br>Chemo and surgery 27.9%<br>Chemo and radio 11.7%<br>Chemo, radio and surgery 9.9%<br>Surgery 5.4%<br>Other 5.4%	PedsQL MFS-Brazilian version<br><br>**Cases of Fatigue:**<br>n.a.	**Reliability:**<br>"[…] overall scale reliability was acceptable, as Cronbach's alpha statistic values varied between 0.70 and 0.90 for all dimensions, self and proxy versions. The only exception was the self-reported dimension sleep/rest<br>fatigue, for which a Cronbach's alpha of 0.55 was observed."<br>"Overall, the results showed acceptable levels of reliability, except for the self-reported sleep/rest fatigue dimension."<br><br>**Validity:**<br>"[…] in all cases linear correlation coefficients were greater than 0.40 for the dimension to which the item belonged (convergent validity). Adjustment values of 100% for all dimensions for both the proxy and self-reported versions (divergent validity) were also observed."<br>"Root mean square error of approximation values were also within acceptable limits (0.08-0.10), with 0.098 and 0.095 for the self-report and proxy versions, respectively. These values indicate that the factorial structure of the construct is maintained in the model adapted for Brazil."<br>"The comparative fit index for children and teenagers was lower than the expected threshold of 0.90 (0.699 and 0.847, for the self and proxy versions, respectively)."<br><br>"The results of this study demonstrate acceptable reliability and validity of the Brazilian version of the scale for use in children with cancer."	Selection bias: 0<br>Convenience sample<br><br>Attrition bias: 1<br>Outcome was assessed for >75% of remaining<br><br>Detection bias: 0<br>No blinding<br><br>Confounding: 1<br><br>Total 2/4

Table S11 continued

5. What is the most reliable and valid diagnostic tool to diagnose Fatigue in CAYA survivors?				
Baptista et al. Psychometric properties of the multidimensional fatigue inventory in Brazilian Hodgkin's lymphoma survivors. 2012				
**Study Design<br>Treatment era<br>Years of follow-up<br>Diagnostic tool**	**Participants**	**Diagnostic tool**	**Main outcomes**	**Quality assessment<br>Remarks**
**Study Design:**<br>Cross sectional study<br><br>**Treatment era:**<br>1996-2004<br><br>**Years of follow-up:**<br>Median follow-up was seven years (range 3.6-12.7 years)<br><br>**Country:**<br>Brazil	**Sample size:**<br>N=200<br><br>**Diagnoses:**<br>Hodgkin's lymphoma<br><br>**Age at diagnosis:**<br>n.a.<br><br>**Age at study:**<br>Median age 29 years (range 16-77 years)<br><br>**Treatment:**<br>n.a.	Brazilian version of the Multidimensional Fatigue Inventory<br><br>**Cases of Fatigue:**<br>n.a.	**Reliability:**<br>Overall Cronbach's alpha: "The overall Cronbach's alpha coefficient for the 20 items was 0.84, and the Cronbach's alpha of each of the five scales ranged from 0.59 to 0.81."<br>"Cronbach's alpha coefficient was higher than 0.7 in all dimensions, indicating a fairly good reliability, except for "reduced motivation"."<br><br>**Validity:**<br>Construct validity: "The factor analysis yielded a five-factor solution that explained 65% of the variance, which is consistent with the multidimensional concept of fatigue."<br><br>"The present findings support the reliability and validity of the Brazilian Portuguese version, which can be used to assess fatigue in clinical and epidemiological studies; […]"	Selection bias: 0<br>Only 229/335 were contacted = 68.4%<br><br>Attrition bias: 1<br>Outcome was assessed for >75% of remaining<br><br>Detection bias: 0<br>No blinding<br><br>Confounding: 1<br><br>Total 2/4

Table S11 continued

**5. What is the most reliable and valid diagnostic tool to diagnose Fatigue in CAYA survivors?**				
Robert et al. Feasibility, reliability, and validity of the Pediatric Quality of Life Inventory generic core scales, cancer module, and multidimensional fatigue scale in long-term adult survivors of pediatric cancer. 2012				
**Study Design<br>Treatment era<br>Years of follow-up<br>Diagnostic tool**	**Participants**	**Diagnostic tool**	**Main outcomes**	**Quality assessment<br>Remarks**
**Study Design:**<br>Cross-sectional study<br>**Treatment era:**<br>n.a.<br>**Years of follow-up:**<br>25.2 years (range 5-43)<br>**Country:**<br>USA	**Sample size:**<br>N=64<br>**Diagnoses:**<br>Solid tumor 51.6%<br>Leukemia 17.2%<br>Lymphoma 17.2%<br>CNS tumor 14.1%<br>**Age at diagnosis:**<br>Mean 9.6 years (range 1-21)<br>**Age at study:**<br>Mean 34.5 years (range 25-53)<br>**Treatment:**<br>n.a.	PedsQL Multidimensional Fatigue Scale (adaptation to 18-25 year olds)	**Reliability:**<br>PedsQL Multidimensional Fatigue Scale: Total Fatigue Score had a Cronbach's alpha of 0.95; all subscales >0.88<br>**Validity:**<br>n.a.	Selection bias: 0<br>Convenience sample<br><br>Attrition bias: 0<br>Outcome was assessed for <75% of remaining<br><br>Detection bias: 0<br>No blinding<br><br>Confounding: 1<br><br>Total 1/4

Table S11 continued

5. What is the most reliable and valid diagnostic tool to diagnose Fatigue in CAYA survivors?				
Nagai et al. Fatigue in survivors of childhood acute lymphoblastic and myeloid leukemia in Japan. 2012				
**Study Design<br>Treatment era<br>Years of follow-up<br>Diagnostic tool**	**Participants**	**Diagnostic tool**	**Main outcomes**	**Quality assessment<br>Remarks**
**Study Design:**<br>Cross-sectional study<br>**Treatment era:**<br>n.a.<br>**Years of follow-up:**<br>Mean time after completion of treatment: 5.8 years (SD 3.8)<br>**Country:**<br>Japan	**Sample Size:**<br>n = 81<br>**Diagnoses:**<br>ALL 77.8% and AML 22.2%<br>**Age at diagnosis:**<br>Mean 6.7 years (SD 3.5)<br>**Age at study:**<br>Mean 14.1 years (SD 5.7)<br>**Treatment:**<br>Chemo only 55.6%<br>Chemo + radiation 9.9%<br>Chemo + SCT 12.3%<br>Chemo + radiation + SCT 22.2%	Devised their own 12-item fatigue questionnaire	**Validity:**<br>“Cronbach’s alpha for the total and each of the three fatigue dimension scores was between 0.75 and 0.88 in both the patient and control groups. These values (i.e. >0.7) are considered to indicate good internal consistency.”<br>**Reliability:**<br>“We evaluated the reliability of the questionnaire by comparing total fatigue scores in the control subjects with the subscales in the Chalder scale. The correlation coefficient between the questionnaire and the Chalder scale was 0.89, supporting the construct validity of the questionnaire.”<br><br>“We developed our own questionnaire consisting of 12 items, and it demonstrated good validity and reliability.”	Selection bias: 0<br>Participants were recruited at follow-up appointment, unclear whether convenience sample or population based<br><br>Attrition bias: 1<br>81/90 included & analyzed<br><br>Detection bias: 0<br>No blinding possible<br><br>Confounding: 1<br><br>Total 2/4

Table S11 continued

**5. What is the most reliable and valid diagnostic tool to diagnose Fatigue in CAYA survivors?**				
Bektas et al. Developing scales for the assessment of fatigue in Turkish pediatric oncology patients aged 13-18 and their parents. 2014				
**Study Design<br>Treatment era<br>Years of follow-up<br>Diagnostic tool**	**Participants**	**Diagnostic tool**	**Main outcomes**	**Quality assessment<br>Remarks**
**Study Design:**<br>Cross-sectional study<br><br>**Treatment era:**<br>n.a.<br><br>**Years of follow-up:**<br>n.a.<br><br>**Country:**<br>Turkey	**Sample size:**<br>N=184<br><br>**Diagnoses:**<br>57.6% leukemia<br><br>**Age at diagnosis:**<br>n.a.<br><br>**Age at study:**<br>Average age 14.6+/-1.4<br><br>**Treatment:**<br>59.8% only chemotherapy	Scale for the Assessment of Fatigue in Pediatric Oncology Patients Aged 13-18<br><br>Scale for the Assessment of Fatigue in Pediatric Oncology Patients Aged 13-18 for Parents	**Sensitivity:**<br>Child form: 1.00 (cutoff 75.5 points)<br>Parent form: 1.00 (cutoff 73 points)<br><br>**Specificity:**<br>Child form: 0.06 (cutoff 75.5 points)<br>Parent form: 0.06 (cutoff 73 points)<br><br>**Reliability:**<br>Cronbach's alpha=0.99 in total for the scale<br><br>**Validity:**<br>Parent Form:<br>"The Kaiser-Meyer-Olkin coefficient (KMO) was determined as 0.799"<br>"The total variance being explained is 90.5%."<br>Known group comparison: "a statistically significant difference was determined between the score averages"<br><br>Child Form:<br>"the KMO was determined as 0.777"<br>"The total variance being explained is 89.4%."<br>Known group comparison: "a statistically significant difference was determined between the score averages"<br><br>"This study suggests that the Scale for the Assessment of Fatigue in Pediatric Oncology Patients Aged 13-18 and the Scale for the Assessment of Fatigue in Pediatric Oncology Patients Aged 13-18 for Parents are valid and reliable instruments in assessing the fatigue symptoms of children in Turkey."	Selection bias: 0<br>Convenience sample<br><br>Attrition bias: 1<br>Outcome was assessed for >75% of remaining<br><br>Detection bias: 0<br>No blinding<br><br>Confounding: 1<br><br>Total 2/4

Table S11 continued

5. What is the most reliable and valid diagnostic tool to diagnose Fatigue in CAYA survivors?				
Brand et al. Screening for fatigue in adolescent and young adult pediatric brain tumor survivors: accuracy of a single-item screening measure. 2016				
**Study Design<br>Treatment era<br>Years of follow-up<br>Diagnostic tool**	**Participants**	**Diagnostic tool**	**Main outcomes**	**Quality assessment<br>Remarks**
**Study Design:**<br>Cross-sectional<br>**Treatment era:**<br>Not mentioned<br>**Years of follow-up:**<br>Mean time since diagnosis 10.55 years (SD 5.57; range 2-27 years)<br>**Country:**<br>USA	**Sample size:**<br>n=142<br>**Diagnoses:**<br>Brain tumor:<br>Low-grade glioma: n=80<br>Embryonal tumor: n=29<br>Ependymoma: n=14<br>Craniopharyngioma: n=8<br>Germ cell: n=8<br>Choroid plexus: n=2<br>High-grade glioma: n=1<br>**Age at diagnosis:**<br>Mean 9.72 (SD 4.87; range 4 months-22 years)<br>**Age at study:**<br>Mean 20.24 (SD 4.81; range 12-32 years)<br>**Controls:**<br>No	Fatigue Thermometer (FT): Visual scale labeled from 0 (no fatigue) to 10 (worst fatigue imaginable).<br><br>Pediatric Quality of life inventory multidimensional fatigue scale (MFS) : 18 items rated on 5 point Likert scale. Higher scores indicate fewer symptoms of fatigue.	"The AUC for the FT was 0.822, indicating the FT had good diagnostic utility relative to the gold standard of the total MFS."<br>"No possible cutoff scores for the FT could be chosen that resulted in a sensitivity and specificity meeting the a priori criteria (sensitivity of >0.90 and specificity of >0.75)."<br>"Results from this study suggest that a single-item screening measure for fatigue is not able to reliably identify clinically significant fatigue in AYA brain tumor survivors."	Selection bias: 0<br>Original cohort brain tumor survivor project REACH: n= 245<br>Eligible for this study: n=191 (77%)<br>Included: n=142 (58%)<br>Attrition bias: 1<br>81/90 included & analyzed<br>Detection bias: 0<br>No blinding possible<br>Confounding: 1<br><br>Total 2/4

Table S11 continued

5. What is the most reliable and valid diagnostic tool to diagnose Fatigue in CAYA survivors?				
Kudubes et al. Developing a scale for the assessment of fatigue in pediatric oncology patients aged 7-12 for children and parents. 2014				
**Study Design<br>Treatment era<br>Years of follow-up<br>Diagnostic tool**	**Participants**	**Diagnostic tool**	**Main outcomes**	**Quality assessment<br>Remarks**
**Study Design:**<br>Cross sectional study<br><br>**Treatment era:**<br>n.a.<br><br>**Years of follow-up:**<br>n.a.<br><br>**Country:**<br>Turkey	**Sample size:**<br>N=204<br><br>**Diagnoses:**<br>Leukemia 49%<br><br>**Age at diagnosis:**<br><br>**Age at study:**<br>Aged 7-12 years<br><br>**Treatment:**<br>Only chemo 51%<br>Chemo and radio and surgery 25%<br>Radiotherapy head-neck 18.1%	Scale for the Assessment of Fatigue in Pediatric Oncology Patients Aged 7-12<br><br>Scale for the Assessment of Fatigue in Pediatric Oncology Patients Aged 7-12 for Parents	**Sensitivity:**<br>Child form: 0.73<br><br>**Specificity:**<br>Child form: 0.93<br><br>**Reliability:**<br>Cronbach's alpha=0.98 in total for the scale<br><br>**Validity:**<br>Parent Form:<br>"the Kaiser-Meyer-Olkin coefficient (KMO) was determined as 0.791 […] The total variance being explained is 85.7%."<br>Known group comparison: "a statistically significant difference was determined between the score averages"<br><br>Child Form:<br>"the KMO was determined as 0.863 […] The total variance being explained is 84.7%"<br>Known group comparison: "a statistically significant difference was determined between the score averages"<br><br>"This study suggests that our scales for the assessment of fatigue in pediatric oncology patients aged 7-12 and their parents are valid and reliable instruments."	Selection bias: 0<br>Convenience sample<br><br>Attrition bias: 1<br>Outcome was assessed for >75% of remaining<br><br>Detection bias: 0<br>No blinding<br><br>Confounding: 1<br><br>Total 2/4<br><br>**Remarks:**

Table S11 continued

5. What is the most reliable and valid diagnostic tool to diagnose Fatigue in CAYA survivors?				
Gerceker et al. Reliability and validity of Turkish versions of the child, parent and staff cancer fatigue scales. 2012				
**Study Design**<br>**Treatment era**<br>**Years of follow-up**<br>**Diagnostic tool**	**Participants**	**Diagnostic tool**	**Main outcomes**	**Quality assessment**<br>**Remarks**
**Study Design:**<br>Cross-sectional study<br><br>**Treatment era:**<br><br>**Years of follow-up:**<br>still in active treatment<br><br>**Country:**<br>Turkey	**Sample size:**<br>N=52 children<br>N=86 parents<br>N=43 nurses<br><br>**Diagnoses:**<br>Leukemia 59.6%<br>Lymphoma 11.5%<br>Other 28.9%<br><br>**Age at diagnosis:**<br>7-12 years<br><br>**Age at study:**<br>7-12 years;<br>Mean age 9.67 years (SD1.89)<br><br>**Treatment:**<br>Corticosteroid treatment 44.2%<br>Radiotherapy treatment 21.2%<br>Surgery treatment 25.0%	Child Fatigue Scale-24 Hours<br>Parent Fatigue Scale-24 Hours<br>Staff Fatigue Scale-24 Hours<br><br>**Cases of Fatigue:**	**Reliability:**<br>"The Cronbachs Alpha coefficient for internal consistency was ascertained for the CFS-24 hours as 0.83; for the PFS-24 hours as 0.77 and for the SFS-24 hours as 0.72."<br><br>**Validity:**<br>Content Validity was tested by assessing the appropriateness of all items by ten academics working in the field of pediatrics and oncology. The items that needed improvements were reviewed once again and changes were made.<br><br>"[…] the Turkish versions of CFS-24 hours, SFS-24 hours and PFS-24 hours were reaffirmed as valid and reliable in evaluating cancer related fatigue."	Selection bias: 0<br>Convenience sample<br><br>Attrition bias: 1<br>Outcome was assessed for >75% of remaining<br><br>Detection bias: 0<br>No blinding<br><br>Confounding: 1<br><br>Total 2/4

Table S11 continued

5. What is the most reliable and valid diagnostic tool to diagnose Fatigue in CAYA survivors?				
Tomlinson et al. Psychometric properties of instruments used to measure fatigue in children and adolescents with cancer: a systematic review. 2013				
**Study Design**<br>**Treatment era**<br>**Years of follow-up**<br>**Diagnostic tool**	**Participants**	**Diagnostic tool**	**Main outcomes**	**Quality assessment**<br>**Remarks**
**Study Design:**<br>Systematic review<br><br>**Treatment era:**<br>n.a.<br><br>**Years of follow-up:**<br>n.a.	**Sample size:**<br>N=25 articles		The most commonly used instruments were:<br>1. Fatigue Scale-Child (FS-C) and Fatigue Scale-Adolescent (FS-A) and the proxy versions for parents (Fatigue Scale-Parents) and staff (Fatigue Scale-Staff) and<br>2. PedsQL Multidimensional Fatigue Scale (MFS) self-report and parent proxy versions.<br>Four other CRF instruments also had psychometric properties reported (Pediatric Functional Assessment of Chronic Illness Therapy-Fatigue; Memorial Symptom Assessment Scale (MSAS); Daily Fatigue Report Scale; and McCorkle Symptom Distress Scale (SDS).<br><br>The FS-C is recommended for children aged 7-12 years, the FS-A for adolescents aged 13-18 years. The FS generally has good internal consistency, inter-rater reliability and responsiveness. Known group validity is more variable.<br><br>The PedsQL MFS child report has versions for three age ranges (5-7, 8-12 and 13-18 years), the parent report includes a forth age group (2-4 years).<br>In general, this instrument has good internal consistency and responsiveness. Similar to the Fatigue Scale, known group validity is inconsistent.<br><br>"In conclusion, our findings demonstrate that either the Fatigue Scale or the PedsQL MFS can be incorporated into clinical trials as endpoints when the intention of the study is to evaluate fatigue or the effects of an intervention on fatigue in a population of children or adolescents with cancer."	Remark:<br>"[…] fatigue is primarily a subjective experience; child self-report should be the primary source of information for fatigue intensity where possible, based on age, cognitive and communicative abilities, and situational factors."

Table S11 continued

**5. What is the most reliable and valid diagnostic tool to diagnose Fatigue in CAYA survivors?**				
Hinds et al. PROMIS pediatric measures in pediatric oncology: valid and clinically feasible indicators of patient-reported outcomes. 2013				
**Study Design<br>Treatment era<br>Years of follow-up<br>Diagnostic tool**	**Participants**	**Diagnostic tool**	**Main outcomes**	**Quality assessment<br>Remarks**
**Study Design:**<br>Cross-sectional study<br><br>**Treatment era:**<br>n.a.<br><br>**Years of follow-up:**<br>Active treatment (received disease-directed therapy within the past 45 days) or survivorship group (completed cancer treatment, disease-free, in follow-up care)<br><br>**Country:**<br>USA	**Sample size:**<br>N=200<br>(n=107 survivors; n=93 in active treatment)<br><br>**Diagnoses:**<br>Leukemia or lymphoma n=120 (60.0%)<br>Brain tumor N=22 (11.0%)<br>Solid tumor n=58 (29.0%)<br><br>**Age at diagnosis:**<br><17 years<br><br>**Age at study:**<br>8-12y 45.5%<br>13-17y 54.5%<br>Mean age 12.9 years (SD 2.9)<br><br>**Treatment:**<br>n.a.	Eight PROMIS pediatric measures:<br>1. Physical Functioning-Mobility<br>2. Physical Functioning-Upper Extremity<br>3. Pain Interference<br>4. Fatigue<br>5. Depression<br>6. Anxiety<br>7. Peer Relationships<br>8. Anger	**Sensitivity:**<br>n.s<br><br>**Specificity:**<br>n.s.<br><br>**Reliability:**<br>n.s.<br><br>**Validity:**<br>Known-group validity: children in the active treatment group had significantly higher (worse) scores on the PROMIS fatigue outcome measure (short form):<br>Active treatment: 52.9 (SD 13.5)<br>vs. Survivorship care: 43.8 (SD 11.8), p<0.001<br>This remained so even after controlling for demographic variables, tumor type, and presence/absence of other health problems.<br><br>Acceptability and feasibility of the PROMIS measures was high. Known-groups validity was supported by the findings.	Selection bias: 1<br>200/203 participated<br><br>Attrition bias: 1<br>195/200 analyzed<br><br>Detection bias: 0<br>Blinding not possible<br><br>Confounding: 1<br>Controlled for main confounders<br><br>Total 3/4

Table S11 continued

5. What is the most reliable and valid diagnostic tool to diagnose Fatigue in CAYA survivors?				
Mandrell et al. Psychometric and Clinical Assessment of the 13-Item Reduced Version of the Fatigue Scale-Adolescent Instrument. 2011				
**Study Design<br>Treatment era<br>Years of follow-up<br>Diagnostic tool**	**Participants**	**Diagnostic tool**	**Main outcomes**	**Quality assessment<br>Remarks**
**Study Design:**<br>Cross-sectional study<br><br>**Treatment era:**<br>2011<br><br>**Years of follow-up:**<br>Assessed during active treatment<br><br>**Country:**<br>USA	**Sample size:**<br>N=138<br><br>**Diagnoses:**<br>ALL 37.7%<br>AML 2.9%<br>HL/Lymphoma 37.7%<br>Solid tumor 18.1%<br>Germ cell tumor 3.6%<br><br>**Age at diagnosis:**<br>See below.<br><br>**Age at study:**<br>Mean age of 15.51 years<br><br>**Treatment:**	Fatigue Scale-Adolescent (13-18 year old)	**Reliability:**<br>"13-item FS-A achieved an internal consistency coefficient (Cronbach alpha) of 0.87."<br>"Confirmatory factor analysis suggested a reasonable fit of the 4-factor structure: The goodness-of-fit index was 0.8551, and the root mean square residual was 0.080. The Spearman correlation coefficient between the FS-A and FS-P was 0.347 (p=0.0033) in the 75 patient/parent dyads."<br><br>"According to the Youden index, the cut score of the 13-item FS-A was 31, sensitivity was 66.6%, and specificity was 82.6%. The AUC was 0.797."<br><br>"The 13-item FS-A has acceptable psychometric properties and is able to identify adolescent oncology patients with high fatigue."	Selection bias: 0<br>Unclear how the participants were recruited in the 9 studies.<br><br>Attrition bias: 0<br>Unclear<br><br>Detection bias: 0<br>No blinding possible<br><br>Confounding: 1<br><br>Total 1/4

Table S11 continued

**5. What is the most reliable and valid diagnostic tool to diagnose Fatigue in CAYA survivors?**				
Ho et al. The Psychometric Properties of the Chinese Version of the Fatigue Scale for Children. 2016				
**Study Design**<br>**Treatment era**<br>**Years of follow-up**<br>**Diagnostic tool**	**Participants**	**Diagnostic tool**	**Main outcomes**	**Quality assessment**<br>**Remarks**
**Study Design:**<br>Cross-sectional design<br><br>**Treatment era:**<br>n.a.<br><br>**Years of follow-up:**<br>Mean time of recovery was 4.2 years<br><br>**Country:**<br>China, Hong Kong	**Sample size:**<br>N=200 cancer survivors (CS)<br>N=50 cancer children (CC)<br>N=50 healthy children (HC)<br><br>**Diagnoses:**<br>Leukemia 33.5%<br>Lymphoma 23.0%<br>Brain tumor 17.5%<br>Osteosarcoma 13.0%<br>Kidney tumor 7.5%<br>Germ cell tumor 5.5%<br><br>**Age at diagnosis:**<br><12 years (not specified)<br><br>**Age at study:**<br>Median slightly below 9 years (not specified)<br><br>**Treatment:**<br>Chemotherapy 44.5%<br>Surgery 8%<br>BMT 8%<br>Chemo and BMT 15%<br>Surgery and chemo 10.5%<br>Chemo and radio 7.5%<br>Radio and surgery 6.5%	Chinese Version of the Fatigue Scale for Children (FS-C),<br><br>**Cases of Fatigue:**<br>Levels of Fatigue:<br>CS 27.0 (SD 8.3; p=0.02 compared to HC)<br>CC 30.4 (SD 7.2; p<0.001 compared to HC)<br>HC 22.6 (SD 5.0)	**Reliability:**<br>"A Cronbach's alpha of 0.88 confirmed the internal consistency of the Chinese version of the FS-C (14 items), thus supporting its use for research purposes."<br>"All of the items were highly correlated with the scale except item 8, which had a correlation of 0.20. After a thorough discussion among the panel members, it was removed from the scale. Cronbach's alpha for the remaining 13 items was 0.91."<br><br>**Validity:**<br>"The semantic equivalence for items ranged from 83% to 100%, indicating that the meanings of the translated items were equivalent to those of the original items."<br>"After removing item 8 from the analysis, the content validity index was 0.83 for scale and ranged from 0.83 to 1.00 for items."<br>"The convergent validity and discriminant validity of the scales are […] supported."<br>"The mean level of fatigue reported by the survivors was significantly lower than that of the children currently receiving cancer treatment, but statistically significantly higher than that of the healthy comparison group, with P < .05 for both. This supports the Chinese version of the FS-C having good known-group validity."	Selection bias: 0<br>unclear<br><br>Attrition bias: 1<br>Outcome was assessed for >75% of remaining<br><br>Detection bias: 0<br>No blinding<br><br>Confounding: 1<br><br>Total 2/4

Table S11 continued

5. What is the most reliable and valid diagnostic tool to diagnose Fatigue in CAYA survivors?				
Macpherson et al. Comparison of Legacy Fatigue Measures With the PROMIS Pediatric Fatigue Short Form. 2018				
**Study Design<br>Treatment era<br>Years of follow-up<br>Diagnostic tool**	**Participants**	**Diagnostic tool**	**Main outcomes**	**Quality assessment<br>Remarks**
**Study Design:**<br>Longitudinal study<br><br>**Treatment era:**<br>n.a.<br><br>**Years of follow-up:**<br>During treatment<br><br>**Country:**<br>USA	**Sample size:**<br>N=96<br><br>**Diagnoses:**<br>Lymphoma or ALL n=56<br>Solid tumor n=35<br>Brain tumor n=5<br><br>**Age at diagnosis:**<br>n.a.<br><br>**Age at study:**<br>8-12 years: n=40<br>13-18 years: n=56<br><br>**Treatment:**<br>n.a.	PROMIS Pediatric Fatigue Short Form<br><br>Fatigue Scale-Adolescent<br><br>Fatigue Scale-Child<br><br>Symptom Distress Scale fatigue item	**Sensitivity:**<br>n.a.<br><br>**Specificity:**<br>n.a.<br><br>**Reliability:**<br>Over the three time points, Cronbach's Alpha was 0.93-0.94 for FS-A, 0.96 for PROMIS-completed by adolescents, 0.83-0.94 for FS-C, and 0.93-0.94 for PROMIS-completed by children<br><br>**Validity:**<br>Correlations between PROMIS and FS-A were consistently strong (r=0.85-0.9), and moderate to strong for FS-C (r=0.65-0.88).<br>The area under the curve (AUC) was 0.84-0.93 for the FS-A, 0.82-0.87 for the PROMIS-completed by adolescents, 0.84-0.87 for the FS-C, and 0.72-0.86 for the PROMIS-completed by children. Differences between the measures were not statistically significant.	Selection bias: 0<br>Response rate not clear<br><br>Attrition bias: 1<br>At least 84/96 (87.5%) responded at all timepoints<br><br>Detection bias: 0<br>Blinding not possible<br><br>Confounding: 0<br>No multivariable analyses<br><br>Total 1/4<br><br>**Remarks:**<br>T1: beginning of a course of chemotherapy<br>T2: count nadir, on average 11.1 days after T1 (SD=3.2)<br>T3: just before the beginning of the next course of chemotherapy, on average 18.4 days after T2 (SD=10.7) and 28.6 days after T1 (SD=10.8)<br><br>Same sample as Hinds et al. 2019

Table S11 continued

**5. What is the most reliable and valid diagnostic tool to diagnose Fatigue in CAYA survivors?**				
Lai et al. Computerized Adaptive Testing in Pediatric Brain Tumor Clinics. 2017				
**Study Design<br>Treatment era<br>Years of follow-up<br>Diagnostic tool**	**Participants**	**Diagnostic tool**	**Main outcomes**	**Quality assessment<br>Remarks**
**Study Design:**<br>Cross-sectional study<br><br>**Treatment era:**<br>n.a.<br><br>**Years of follow-up:**<br>Years since diagnosis: mean 5.2 yrs (SD=4.6)<br>Years since last treatment: mean 3.7 yrs (SD=3.4)<br><br>**Country:**<br>USA	**Sample size:**<br>N=161<br><br>**Diagnoses:**<br>Brain tumors<br>Embryonal tumors medulloblastoma 23.0%<br>Ganglioma 18.3%<br>Pilocytic astrocytoma 13.5%<br>Astrocytoma (diffuse, infiltrative, fibrillary) 11.1%<br><br>**Age at diagnosis:**<br>n.a.<br><br>**Age at study:**<br>Mean 13.9 yrs (SD=3.7)<br><br>**Treatment:**<br>Chemotherapy 77.5%<br>Radiotherapy 54.2%<br>Surgery 69.9%<br>Surgery, chemotherapy, and radiation 20.3%<br>No surgery, chemotherapy and radiation 5.3%	PROMIS pediatric fatigue measure: computerized adaptive testing (CAT) and short form (SF)	**Sensitivity:**<br>n.a.<br><br>**Specificity:**<br>n.a.<br><br>This study compared PROMIS CAT to PROMIS SF measures.<br><br>Correlations between CAT and SF fatigue scores were strong: Pearson r=0.976<br>Correlations were acceptable when comparing CAT and SF fatigue scores by T-score groups (<45 r=0.88, p<0.001; 45-55 r=0.79, p=0.128; >55 r=0.90, p=0.080)<br>Differences between CAT and SF fatigue scores were significantly different (CAT T-score mean 43.7 (SD=12.9) vs. SF T-score mean 44.8 (SD=11.6)), but effect size was 0.08 indicating this difference was negligible.<br><br>The authors recommend use of the dynamic CATs, as they enable a more individualized assessment (floor effects were observed for the PROMIS fatigue SF). However, they need more infrastructure (access to a computer). If no computer is available, fixed-length SFs can be used. PROMIS CATs and SFs produce comparable scores for children with a brain tumor.	Selection bias: 0<br>Unclear how many were recruited.<br><br>Attrition bias: 1<br>At least n=147 analyzed (>75%)<br><br>Detection bias: 0<br>Blinding not possible<br><br>Confounding: 0<br>No multivariable analyses.<br><br>Total 1/4<br><br>**Remarks:**<br>Participants were classified into three groups based on their T-scores: <45 (1/2 standard deviation [SD] below norm), 45-55 (1/2 SD within the norm), and >55 (1/2 SD above the norm). Higher scores represent worse Fatigue.

Table S11 continued

5. What is the most reliable and valid diagnostic tool to diagnose Fatigue in CAYA survivors?				
Al-Gamal et al. The psychometric properties of an Arabic version of the PedsQL Multidimensional Fatigue Scale tested for children with cancer. 2017				
**Study Design<br>Treatment era<br>Years of follow-up<br>Diagnostic tool**	**Participants**	**Diagnostic tool**	**Main outcomes**	**Quality assessment<br>Remarks**
**Study Design:**<br>Cross-sectional study<br><br>**Treatment era:**<br>n.s.<br><br>**Years of follow-up:**<br>During active treatment<br><br>**Country:**<br>Jordan	**Sample size:**<br>N=70<br><br>**Diagnoses:**<br>Leukemia n=34 (46%)<br>Lymphoma n=4 (5.7%)<br>Other types of cancer n=32 (48.3%)<br><br>**Age at diagnosis:**<br><br>**Age at study:**<br>Range 5-18 years<br>Mean 10.17 years (SD=3.4 yrs)<br><br>**Treatment:**<br>n.s.	Paediatric Quality of Life (PedsQL) Multidimensional Fatigue Scale (child report)- Arabic Version<br><br>Compared to PedsQL TM 4.0 Generic Core scale (existing Arabic version)	**Sensitivity:**<br>Not reported<br><br>**Specificity:**<br>Not reported<br><br>**Reliability:**<br>Internal consistency was good or excellent for total scale (α=0.90), general fatigue subscale (α=0.94), and cognitive fatigue subscale (α=0.87). Internal consistency was questionable for sleep/rest subscale (α=0.67).<br>The effect of individual items on the reliability of their subscale was tested using the Alpha if item deleted approach. Removing any individual items on all three subscales resulted in minimal changes in Cronbach's alpha, indicating that all items should be retained.<br><br>**Validity:**<br>To measure construct validity, correlation of PedsQL MFS and PedsQL TM 4.0 Generic Core scale was tested. PedsQL MFS total components and PedsQL TM 4.0 Generic Core subscales were significantly positively correlated: Higher scores on the PedsQL MFS (fewer problems) were associated with higher scores on the PedsQL TM 4.0 Generic Core subscales (better overall HRQoL).<br><br>The authors conclude that the PedsQL Multidimensional Fatigue Scale-Arabic Version is useful to measure fatigue in Arabic children with cancer. They state that reliability was good for the total PedsQL MFS (α=0.90), and that the PedsQL Multidimensional Fatigue Scale-Arabic Version is a valid instrument.	Selection bias: 0<br>Unclear<br><br>Attrition bias: 1<br>All n=70 assessed.<br><br>Detection bias: 0<br>No blinding possible.<br><br>Confounding: 0<br>Analyses not controlled for confounders.<br><br>Total 1/4

Table S11 continued

5. What is the most reliable and valid diagnostic tool to diagnose Fatigue in CAYA survivors?				
Hinds et al. PROMIS pediatric measures validated in a longitudinal study design in pediatric oncology. 2019				
**Study Design<br>Treatment era<br>Years of follow-up<br>Diagnostic tool**	**Participants**	**Diagnostic tool**	**Main outcomes**	**Quality assessment<br>Remarks**
**Study Design:**<br>Longitudinal study (three time points)<br>**Treatment era:**<br>n.a.<br>**Years of follow-up:**<br>During chemotherapy. Mean time since diagnosis was 0.7 years<br>**Country:**<br>USA	**Sample size:**<br>N=96<br>**Diagnoses:**<br>ALL/Lymphoma n=56 (58.3%)<br>Brain tumor n=5 (5.2%)<br>Solid tumor n=35 (36.5%)<br>**Age at diagnosis:**<br>n.a.<br>**Age at study:**<br>8-12 years: n=40 (41.7%)<br>13-18 years: n=56 (58.3%)<br>**Treatment:**<br>n.a.	PROMIS pediatric short-form fatigue<br><br>Symptom Distress Scale (SDS)	**Sensitivity:**<br>n.a.<br>**Specificity:**<br>n.a.<br>**Reliability:**<br>n.a.<br>**Validity:**<br>Construct validity: PROMIS fatigue scores increased (got worse) from T1 to T2, but decreased (improved) at T3. PROMIS fatigue scores correlated with PROMIS performance measures (mobility, peer relationship, and upper extremity function; r=(-0.3)-(-0.68), all p<0.01). Results suggest reasonable construct validity of the PROMIS fatigue measure.<br>Concurrent validity: Correlations of the PROMIS fatigue measure with the corresponding items of the SDS were highly significant (p<0.0001). Correlation coefficients are not presented separately for the different PROMIS symptom measures. Results suggest concurrent validity of the PROMIS fatigue measure.<br>**Responsiveness to change:**<br>The standardized response mean (SRM) was small for fatigue (0.29). In terms of within-child analyses (short-term responsiveness), fatigue worsened slightly but not significantly from T1 to T2, then improved significantly from T2 to T3. For long-term responsiveness (T1 to T3) and using generalized estimating equation (GEE; controlling for age, sex, hemoglobin, and time since diagnosis) fatigue scores improved significantly as predicted.<br>Importantly, the cancer-specific SDS was not as responsive across time as the PROMIS pediatric measures.	Selection bias: 0<br>Unclear how many participants were contacted<br><br>Attrition bias: 1<br>All analyzed<br><br>Detection bias: 0<br>No blinding possible<br><br>Confounding: 1<br>Controlled for important confounders<br><br>Total 2/4<br><br>**Remarks:**<br>T1: time of stability, between 7 days before the start of a new course of chemotherapy<br>T2: during course of chemotherapy treatment when adverse effects were predictably present; average 11.1 days after T1<br>T3: after the course of chemotherapy, when stability was predictably achieved; average 18.4 days after T2

Table S11 continued

5. What is the most reliable and valid diagnostic tool to diagnose Fatigue in CAYA survivors?				
Hinds et al. Validity and Reliability of a New Instrument to Measure Cancer-Related Fatigue in Adolescents. 2007				
**Study Design<br>Treatment era<br>Years of follow-up<br>Diagnostic tool**	**Participants**	**Diagnostic tool**	**Main outcomes**	**Quality assessment<br>Remarks**
**Study Design:**<br>4 studies:<br>1. Measuring Fatigue in Childhood Cancer (MFCC): longitudinal (2 timepoints)<br>2. Sleep, Fatigue, and Dexamethasone in Childhood ALL (SLEEP): longitudinal (4 timepoints)<br>3. Sleep, Fatigue, and Enhanced Physical Activity in Hospitalized Pediatric Oncology Patients (SLEEP2; 2-4 timepoints)<br>4. Symptom Clusters in Pediatric Oncology (CLUSTERS; 3 timepoints)<br>**Treatment era:**<br>n.a.<br>**Years of follow-up:**<br>In treatment<br>**Country:**<br>USA	**Sample size:**<br>A total of n=64 adolescents in the 4 studies (and n=61 parents, and n=18 staff)<br>**Diagnoses:**<br>ALL n=39 (60.9%)<br>AML n=3 (4.7%)<br>Hodgkin's disease/lymphoma n=6 (9.4%)<br>Solid tumor n=16 (25.0%)<br>**Age at diagnosis:**<br>n.a.<br>**Age at study:**<br>Mean age 15.3 years (SD 1.52); range 12.75-18.26 years<br>**Treatment:**<br>n.a.	Fatigue Scale-Adolescent (FS-A), with parent and staff versions<br><br>Reynolds Depression Scale (RDS)	**Sensitivity:**<br>n.a.<br>**Specificity:**<br>n.a.<br>**Internal consistency:**<br>For 11 of the 13 data-collection points, the FS-A had strong coefficient alpha estimates, Cronbach-if-deleted coefficients ranged from 0.597 to 0.956. Two items (9 and 10) diminished reliability. Item to scale correlation ranged from 0.24 to 0.92, except for item 10 (r=-0.088 at one time point). Standardized Cronbach's Alpha coefficient was between 0.70 and 0.95 for 10/13 timepoints (FS-A), 10/13 timepoints (FS-P), 2/5 timepoints (FS-S). For the MFCC study, Cronbach's Alpha was 0.81 (FS-A), 0.75 (FS-P), and 0.85 (FS-S) at T1.<br>**Validity:**<br>Exploratory factor analysis revealed four factors of the FS-A, all items loaded onto these four factors and correlation coefficients were 0.353-0.758.<br>Correlation between total scale scores of RDS and FS-A were strong (r=0.71, p<0.001)<br>Known-group comparison: anemic patients scored higher than non-anemic patients by parent-report (p=0.04)<br>**Responsiveness:**<br>Across the four studies, the FS-A scores increased significantly between the two designated time points (p=0.01), but FS-P scores did not.	Selection bias: 0<br>Unclear how many participants were contacted<br><br>Attrition bias: 1<br>All analyzed<br><br>Detection bias: 0<br>No blinding possible<br><br>Confounding: 1<br>Controlled for important confounders<br><br>Total 2/4<br><br>**Remarks:**<br>T1: time of stability, between 7 days before the start of a new course of chemotherapy<br>T2: during course of chemotherapy treatment when adverse effects were predictably present; average 11.1 days after T1<br>T3: after the course of chemotherapy, when stability was predictably achieved; average 18.4 days after T2

Table S11 continued

6. What is the **effect of individual cognitive behavioral therapy** in the treatment of CRF in CAYA cancer survivors?				
Boonstra et al. Cognitive Behavior Therapy for Persistent Severe Fatigue in Childhood Cancer Survivors: A Pilot Study. 2018				
**Study Design<br>Treatment era<br>Years of follow-up<br>Diagnostic tool**	**Participants**	**Intervention**	**Main outcomes**	**Quality assessment<br>Remarks**
**Study Design:**<br>Intervention study<br><br>**Treatment era:**<br>n.a.<br><br>**Years of follow-up:**<br>Time since diagnosis mean: 13.0 years (SD 7.3), range: 5-34 years<br><br>**Country:**<br>The Netherlands<br><br>**Fatigue measurement:**<br>Fatigue Severity Subscale of the Checklist Individual Strength (CIS); severe fatigue was defined as a score of ≥35 on the Fatigue Severity Subscale of the CIS and a duration of fatigue of at least 6 months.	**Sample size:**<br>N=33 (males: n=8)<br><br>**Diagnoses:**<br>Leukemia: n=13<br>Lymphoma: n=7<br>Bone cancer: n=5<br>Solid cancer: n=4<br>Brain cancer: n=3<br>Other cancer: n=1<br><br>**Age at diagnosis:**<br>Mean: 9.7 years (SD 4.4), range: 0-17 years<br><br>**Age at study:**<br>Mean: 23.1 years (SD 7.0), range 11-42 years<br><br>**Treatment:**<br>Chemotherapy: n=29<br>Surgery: n=13<br>Stem cell transplantation radiotherapy: n=4<br>Noncranial (spinal) radiotherapy: n=8<br>Cranial (spinal) radiotherapy: n=1	Patients suffering fatigue completed a questionnaire assessing fatigue severity and maintaining factors, demographic and health characteristics. Medical records provided data on the medical history. A face-to-face interview was conducted with the aim to screen for the presence of psychiatric disorders and psychological problems that might explain fatigue and eligible survivors were offered cognitive behavioral therapy (CBT). CBT consisted of 12 to 14 sessions over 6 to 8 months, covering 6 modules addressing different maintaining factors (coping with cancer, fear of recurrence, cognitions with regard to fatigue, social interactions, sleep-wake pattern, activity pattern regulation including a graded activity program).	Using intention-to-treat analyses, fatigue serverity decreased significantly from pretreatment to posttreatment (pretreatment mean 46.2 (SD 4.5) vs. posttreatment mean 28.9 (SD 13.7); mean difference -17.4 (95%CI:-22.1 to -12.7, p<0.001)) and the effect size was large (1.7 (95%CI: 1.1-2.3).<br><br>In total, 23 of the 33 CCS (70%) showed a clinically significant improvement of fatigue. Of the CCSs who completed CBT (N =25), 22 (88%) survivors reported a clinically significant improvement.<br><br>Of the 25 survivors who completed CBT, 5 CCS indicated that they were completely recovered (20%), 17 CCS reported a significant improvement (68%), and 3 CCS reported that fatigue levels had not changed (12%).	Selection bias: 1<br>- Response rate overall: 76%<br>Attrition bias: 1<br>- Eligible: n=33<br>- Complete: n=25 (76%)<br>Detection bias: 0<br>- No blinded outcome assessors<br>Confounding: 0<br>- No multivariable analyses, no control group<br><br>**Total quality: 2/4**<br><br>Remarks:<br>No control group

Table S11 continued

**9.** What is the effect of **any intervention** in the treatment of Fatigue in CAYA survivors?				
Chang et al. Systematic Review and Meta-Analysis of Nonpharmacological Interventions for Fatigue in Children and Adolescents With Cancer. 2013				
**Study Design**<br>**Treatment era**<br>**Years of follow-up**<br>**Diagnostic tool**	**Participants**	**Intervention**	**Main outcomes**	**Quality assessment Remarks**
**Study Design:**<br>Systematic Review and Meta-Analysis<br>**Treatment era:**<br>n.a.<br>**Years of follow-up:**<br>All except one study on CAYA patients<br>**Diagnostic tool:**<br>• Fatigue-CIS-20<br>• Lansky play performance scale (PPS)<br>• Child fatigue scale (CFS)<br>• **Fatigue Scale (FS-C, FS-A, FS-P, FS-S)**<br>• Pediatric Quality of Life Multidimensional Fatigue Scale (Peds QL-MFS)<br>**Country:**<br>5x USA, 1x Taiwan	**Sample size:**<br>6 studies included<br>**Diagnoses:**<br>Acute lymphoblastic leukemia<br>Acute myeloid leukemia<br>Lymphoma<br>Solid tumor<br>**Age at diagnosis:**<br>Dependent on the study<br>**Age at study:**<br>1-18 years<br>**Treatment:**<br>• Dependent on the study	a. 12-week exercise training in survivors of ALL (n=9)<br>b. 4-week massage therapy in patients (n=17)<br>c. Enhanced physical activity in patients (n=29)<br>d. 16-week physical activity in patients (n=10)<br>e. Effective nursing interventions in patients (n=60)<br>f. 6-week home-based aerobic exercise in patients (n=24)	The meta-analysis included 2 studies (d. & f.) and revealed a statistically significant effect of exercise interventions in reducing general fatigue (effect size = –0.76; 95% CI [–1.35–0.17]) in children and with cancer.<br><br>3 of the 6 studies with no change, the other 3 with significant differences:<br>a. 12-week exercise training → n.s. differences<br>b. 4-week massage therapy → n.s. differences between groups<br>c. Enhanced physical activity → n.s. differences between groups<br>d. 16-week physical activity → sign. differences short- & long-term<br>e. Effective nursing interventions → sign. differences between groups<br>The results indicate that fatigue in children with cancer can be reduced by implementing appropriate nursing interventions (education about fatigue and suggestions for activities that can reduce fatigue).<br>f. 6-week home-based aerobic exercise → sign. differences between groups<br><br>This study found that exercise interventions had no effect on reduction of total fatigue, sleep or rest fatigue, cognitive fatigue.	

Table S11 continued

**9.** What is the effect of **any intervention** in the treatment of Fatigue in CAYA survivors?				
Baumann et al. Clinical exercise interventions in pediatric oncology: a systematic review. 2013				
**Study Design<br>Treatment era<br>Years of follow-up<br>Diagnostic tool**	**Participants**	**Intervention**	**Main outcomes**	**Quality assessment<br>Remarks**
**Study Design:**<br>Systematic review<br>**Treatment era:**<br>Dependent on the study<br>**Years of follow-up:**<br>Dependent on the study, some studies on treatment, some not with cancer patients	**Sample size:**<br>17 studies included, 257 children with cancer<br>**Diagnoses:**<br>Mixed cancer types, but mainly ALL<br>**Age at diagnosis:**<br>0-21 years<br>**Age at study:**<br>Dependent on the study<br>**Treatment:**<br>• Dependent on the study	Different exercise interventions: In-hospital endurance/strength training; home-based endurance exercise program; supervised group exercise and educational intervention; supervised and home-based exercise program	The findings confirm that clinical exercise interventions are feasible and safe, especially with acute lymphoblastic leukemia (ALL) patients and during medical treatment. No adverse effects have been reported.<br><br>Regarding fatigue: two studies found no effect, whereas three studies found a positive effect of clinical exercise during medical treatment or survivorship.<br><br>The authors conclude: “Relatively good evidence is given in terms of positive effects of supervised exercise programs during medical treatment on fatigue, muscle strength, and quality of life.	

Table S11 continued

9. What is the effect of **any intervention** in the treatment of Fatigue in CAYA survivors?				
**Blaauwbroek et al.** The effect of exercise counselling with feedback from a pedometer on fatigue in adult survivors of childhood cancer: a pilot study				
**Study Design<br>Treatment era<br>Years of follow-up<br>Diagnostic tool**	**Participants**	**Intervention**	**Main outcomes**	**Quality assessment<br>Remarks**
**Study Design:**<br>Intervention study<br>**Treatment era:**<br><br>**Years of follow-up:**<br>Mean since diagnosis 21.8. range 14.7-28.9<br>**Fatigue measurement: Visual Analogue Scale for chronic fatigue (VAS fatigue)**<br>**Country:**<br>The Netherlands	**Sample size:**<br>N=46<br>**Diagnoses:**<br>• Leukemia n=22 (46.8%)<br>• Malignant lymphoma n=6 (12.8)<br>• Bone tumor n=4 (8.5)<br>• Soft tissue sarcoma n=3 (6.4%)<br>• Wilms tumor n=1(2.1%)<br>• Langerhans cell histiocytosis n=2 (4.3%)<br>• CNS tumor n=6 (12.8%)<br>• Other n=3 (6.4%)<br>**Age at diagnosis:**<br>Mean age 8 years. Range 1.5-14.8<br>**Age at study:**<br>Median age 29 years. Range 18-61<br>**Controls:**<br>N=33 (recruited by the survivors among healthy siblings or peers)	10 week Home-based daily physical activity counselling programme—with feedback from a pedometer—on fatigue in adult survivors of childhood cancer was evaluated.<br>A counsellor trained according to the COACH protocol visited the survivor and explained the use of pedometer and step diaries at **week 1**. They also filled in Checklist Individual Strength (CIS). Survivor wore the pedometer during two weeks to assess steps at baseline and in week 4 and 10 during the study. In the end of each day they record daily steps counts and duration in minuets in an online or posted diary.<br>At 3, 6 and 9 weeks the counsellor phoned the survivor. At week 3 the use of pedometer were discussed and they were asked how many steps they could improve-Together with the counselor a goal were set. After this conversation, the survivor received a written summary of the conversation and wore the pedometer again.<br>At week 6 the couselor and surv evaluated if the goal were reached if not, they explored barriers. Survivors were asked to plan a peak day were they walked as many steps as possible. Again, a written summary were received and asked to wear the pedometer on the peak day in week 7.<br>At week 9 the counsellor and survivor evaluated the peak day and asked if they could adjust their goal to a higher steps per day. Written summary received of the conversation. And asked to wear pedometer for week 10 and fill out a questionnaire. They did the same in week 36.	The stimulation of daily physical activity using exercise counselling and a pedometer over 10 weeks leads to a significant decrease in fatigue in adult survivors of childhood cancer, and this improvement lasts for at least 36 weeks.<br><br>Mean CIS scores ± SD of participants (81.42±20.14 at T1; 62.62±20.86 at T10 (p<0.0005); 63.67±23.12 at T 36 (p<0.0005 compared to T1)) and controls (47.39±19.06 at T1; 46.18±17.70 at T10; 42.57± 17.40 at T36)<br>There was no statistically significant difference in the mean CIS scores of the controls during the study period.	Selection bias: 0<br><br>Attrition bias: 0<br>n= 486 eligible<br>n=453 were sent questionnaire<br>respons rate 56%. 46 were enrolled into the study but eight dropped out<br>Detection bias: 0<br><br>Confounding bias: 0<br>Descriptive statistics and Linear regression used.<br>**Total quality: 0/4**

Table S11 continued

9. What is the effect of **any intervention** in the treatment of Fatigue in CAYA survivors?			
Robinson et al. Guideline for the Management of Fatigue in Children and Adolescents with Cancer and Pediatric Hematopoietic Stem Cell Transplantation Recipients. 2018			
**Study Design<br>Treatment era<br>Years of follow-up<br>Diagnostic tool**	**Participants**	**Main outcomes**	**Quality assessment<br>Remarks**
**Study Design:**<br>Clinical practice guideline, based on systematic review & meta-analysis<br><br>**Treatment era:**<br>n.a.<br><br>**Years of follow-up:**<br>n.a.<br><br>**Country:**<br>n.a.	**Sample size:**<br>They included 6 pediatric and 456 adult randomized trials<br><br>**Diagnoses:**<br>Mixed<br><br>**Age at diagnosis:**<br>n.a.<br><br>**Age at study:**<br>n.a.<br><br>**Treatment:**<br>n.a.	Recommendation 1: **Use physical activity**. They made a strong recommendation to use physical activity, preferably aerobic, neuromotor (incl. yoga and tai chi), or combination exercises. Quality of evidence was downgraded to moderate, as evidence was mostly from adults.<br><br>Recommendation 2: **Do not routinely use pharmacological approaches**. They made a strong recommendation against erythropoietin use, and methylphenidate use. They made a strong recommendation that pharmacological agents should not be routinely used in the management of fatigue in children and adolescents.<br><br>Recommendation 3: **Use relaxation or mindfulness, or both**. They made a strong recommendation for the use of relaxation, mindfulness or both (acupressure, mindfulness, relaxation techniques, massage therapy, energy therapies, energizing yogic breathing, and others). A challenge might be the implantation of these interventions in younger children and cranial irradiation survivors due to immaturity or cognitive ability.<br><br>Recommendation 4: **Cognitive or cognitive behavioral therapies may be offered**. They made a weak recommendation to use cognitive behavioral therapy, due to higher costs of intervention and lack of randomized data in children. However, if trained professionals are available at an institution, or if physical activity, mindfulness and relaxation were not feasible or successful, cognitive behavioral therapy should be considered.<br><br>Other interventions: Other interventions (e.g. symptom screening, nutrition-focused, music therapy, and cognitive rehabilitation training) were too heterogeneous to analyze and the authors did not formulate a recommendation.<br><br>The authors conclude "Using the Grades of Recommendation Assessment, Development and Evaluation approach, strong recommendations were made for the use of physical activity, relaxation and mindfulness to reduce fatigue. Where these approaches are not feasible or were not successful, cognitive or cognitive behavioral therapies may be offered. Maturity and cognitive ability will influence intervention feasibility. Systemic pharmacological approaches should not be routinely used for the management of fatigue in children."	

Table S11 continued

9. What is the effect of **any intervention** in the treatment of CRF in CAYA cancer survivors?				
Li et al. Adventure-based training to promote physical activity and reduce fatigue among childhood cancer survivors: A randomized controlled trial. 2018				
**Study Design<br>Treatment era<br>Years of follow-up<br>Diagnostic tool**	**Participants**	**Intervention**	**Main outcomes**	**Quality assessment<br>Remarks**
**Study Design:**<br>Randomized Control Trial (RCT)<br>**Treatment era:**<br>n.a.<br>**Years of follow-up:**<br>All completers (n=192):<br><25 months: n=112 (58.3%)<br>25-48 months: n=54 (28.1%)<br>37-60 months: n=40 (20.8%)<br>>60 months: n=4 (2.1%)<br>**Country:**<br>Hong Kong, China<br>**Fatigue measurement: Fatigue Scale-Child (FS-C)**	**Sample size:**<br>N=222 (males: n=118)<br>Experimental gr.: n=117<br>Completed: n=103<br>Dropped out: n=14<br>Control group: n=105<br>Completed: n=89<br>Dropped out: n=16<br>**Diagnoses (all completers n=192):**<br>Leukaemia: n=81 (42.2%)<br>Lymphoma: n=51 (26.6%)<br>Brain tumor: n=25 (13.0%)<br>Bone tumor: n=21 (10.9%)<br>Neuroblastoma: n=14 (7.3%)<br>**Age at diagnosis:**<br>n.a.<br>**Age at study:**<br>Range 9-16 years<br>Experimental (completers): mean 12.8 years (SD 2.6)<br>Control (completers): mean 12.4 years (SD 2.6)<br>**Treatment (all completers n=192):**<br>Surgery: n=14 (7.3%)<br>Chemotherapy: n=137 (71.4%)<br>Radiotherapy: n=5 (2.6%)<br>Mixed method: n=36 (18.8%)	The treatment group participated to 4 training days (2 weeks, 2, 4, 6 months after randomization respectively) of maximum 12 participants. Each session started with a 40-min briefing session. Then, participants take part in adventure activities (ice-breaking, team-building games, shuttle runs, rock climbing, rope courses, descending) with increasing levels of difficulty. After activities, physical fitness was assessed and a 75-min debriefing session was organized. Data were collected at baseline (T1), after 6 (T2) and 12 (T3) months.<br>The **control group** received a placebo treatment including health talks, leisure activities, and museum visits, etc.	Participants in the experimental group reported significantly lower levels of fatigue than those in the control group at the 12-month follow-up.<br><br>T1, mean (SD):<br>Experimental group: 29.4 (4.2)<br>Control group: 29.2 (4.1)<br>p-value: 0.83<br><br>T2 mean (SD):<br>Experimental group: 26.6 (4.9)<br>Control group: 28.5 (4.2)<br>p-value: 0.09<br><br>T3 mean (SD):<br>Experimental group: 22.3 (4.2)<br>Control group: 28.9 (4.9)<br>p-value: <0.001<br><br>Mixed between-within-subjects ANOVA revealed a significant effect for time on cancer-related fatigue (CRF), reflecting a significant change in participants' CRF (Eta Squared=0.61, p<0.001).<br>The effect for interaction on CRF was also significant (Eta Squared=0.55, p<0.001), indicating that the change in CRF over time in the experimental group differed from that in the control group.<br>The effect for intervention on CRF was smaller, but also significant (Eta Squared=0.04, p=0.02); participants in the experimental group reported lower levels of CRF than those in the control group during the 12-month follow-up.<br><br>Participants of the experimental group also reported higher levels of physical activity, self-efficacy, and better QoL than controls at the 12-month follow-up.	Selection bias: 1<br>- Random sample with respect to treatment<br>Attrition bias: 1<br>- Complete experimental: n=103<br>- Complete control: n=89<br>Detection bias: 0<br>It is not mentioned that assessors were blinded<br>Confounding: 1<br>- randomization of participants<br><br>**Total quality: 3/4**<br><br>**Remarks:**<br>Data on diagnosis, age, treatment are provided only for participants who completed either treatment or control (n=192)

Table S11 continued

9. What is the effect of **any intervention** in the treatment of CRF in CAYA cancer survivors?				
Nunes et al. Interventions minimizing fatigue in children/adolescents with cancer: An integrative review. 2018				
**Study Design**<br>**Treatment era**<br>**Years of follow-up**<br>**Diagnostic tool**	**Participants**	**Intervention**	**Main outcomes**	**Quality assessment**<br>**Remarks**
**Study Design:**<br>Integrative review<br>Studies published between January 2000 and December 2016<br><br>**Years of follow-up:**<br>Patients and survivors<br><br>**Country:**<br>6 studies from USA<br>2 from the Netherlands<br>1 each from Taiwan, Germany, Turkey, Canada, Iran & Canada<br><br>**Instrument:**<br>**5 PedsQL-MFS**<br>**6 Fatigue Scales (FS-C, FS-A, FS-P)**<br>1 CIS-20<br>1 Visual Analog Scale<br>1 My Fatigue meter	**Sample size:**<br>Median sample size was n=22; range 9-120<br><br>**Diagnoses:**<br>Mixed diagnoses<br><br>**Age at diagnosis:**<br>Childhood & Adolescence	The studies tested six different types of interventions:<br>1. Exercise<br>2. Exercise plus leisure activities<br>3. exercise plus psychological training<br>4. Massage<br>5. Healing touch<br>6. Acupressure	Exercise (seven studies):<br>Four (n=22/23/11/16) found a decrease of CRF in participants. Interventions used were home-based aerobic exercise, in-patient bicycle ergometer use, in-patient yoga sessions, weekly step goal with FitBitR tracker.<br>Three studies (n=29/9/13) found no effect of exercise on CRF in participants. Interventions used were stationary bicycle-style exerciser, muscular strength/aerobic fitness/resistance range, yoga.<br><br>Exercise plus leisure activities (one study):<br>One study (n=60) found a decrease of CRF in participants after an intervention including exercise (15min) and leisure activities, such as drawing, reading, listening to music (45min).<br><br>Exercise plus psychosocial intervention (one study):<br>One study (n=30) found no effect of a physical exercise training, and additional psychosocial training (psychoeducation and cognitive-behavioral techniques).<br><br>Healing touch (one study):<br>One study (n=9) found a decrease of CRF in participants after an intervention of healing touch.<br><br>Massage (two studies):<br>Two studies (n=17/34) found no effect of massage to reduce CRF.<br><br>Acupressure (one study):<br>One study (n=60) found a decrease of CRF in participants after an intervention of acupressure (point ST36) compared to controls (point LI12). Positive effects were observed only immediately after intervention.	

Table S11 continued

<table>
<tr><td colspan="5">9. What is the effect of any intervention in the treatment of CRF in CAYA cancer survivors?</td></tr>
<tr><td colspan="5">Kudubes et al. The Effect of Fatigue-Related Education on Pediatric Oncology Patients' Fatigue and Quality of Life. 2018</td></tr>
<tr><th>Study Design
Treatment era
Years of follow-up
Diagnostic tool</th><th>Participants</th><th>Intervention</th><th>Main outcomes</th><th>Quality assessment
Remarks</th></tr>
<tr>
<td>Study Design:
Controlled trial, non-randomized

Treatment era:
2015-2017

Years of follow-up:
In active treatment

Country:
Turkey

Fatigue measurement:
Scale for the Assessment of Fatigue in Pediatric Oncology Patients (Versions 7-12 years, and 7-12 years for Parents)[56] (scale ranges from 27-135, higher values indicate less fatigue)

Data was collected at three timepoints: before the intervention (pretest), 3 months later (posttest 1), and 6 months later (posttest 2)</td>
<td>Sample size:
N=80 (each n=40 in the experimental and control group)

Diagnoses (exp. vs. cont.):
Oncological disease:
n=23 (57.5%) vs.
n=21 (52.5%)
Hematologic disease:
n=17 (42.5%) vs.
n=19 (47.5%)

Age at diagnosis:
Newly diagnosed

Age at study:
9.4 years (SD=2.2) vs.
9.1 years (SD=1.7)

Treatment:
Chemotherapy:
n=22 (55.0%) vs.
n=25 (62.5%)
Combination therapy:
n=18 (45.0%) vs.
n=15 (37.5%)
Corticosteroid therapy:
n=33 (82.5%)
n=32 (80.0%)</td>
<td>Based on the literature, an educational pamphlet for children and parents was developed. Different experts were involved.

An intervention including five modules, each consisting of one or two 45min sessions, was developed:
1. Opening and Basic information on Fatigue
2. Fatigue Coping Methods – Symptom Management
3. Fatigue Coping Methods – Energy Conservation and Activity (Exercise) Regulation
4. Fatigue Coping Methods – Ensuring adequate sleep and sleep quality
5. Fatigue Coping Methods – Stress Management/Stress Coping

It's unclear in within what time frame the five modules were delivered, and whether all modules were delivered to patients before posttest 1.</td>
<td>Pretest:
Patients in the experimental group had more fatigue than those in the control group (lower scores indicate more fatigue).
Posttest 1:
Mean level of fatigue for patients in the experimental group improved, while mean level of fatigue in the control group worsened.
Posttest 2:
Mean level of fatigue for patients in the experimental group improved again, while mean level of fatigue in the control group worsened again.
<table>
<tr><th></th><th>Pretest (mean)</th><th>Posttest 1 (mean)</th><th>Posttest 2 (mean)</th></tr>
<tr><td>Experimental</td><td>46.3</td><td>67.9</td><td>77.9</td></tr>
<tr><td>Control</td><td>65.4</td><td>50.5</td><td>29.5</td></tr>
</table>
Multidimensional variance analysis was used:
There was a statistically significant effect of group (F=40.6; p<0.001), indicating that patients in the experimental and control group differ in the total mean scores (fatigue).
There was a statistically significant effect of time (F=4.2; p=0.017), indicating that patients' total mean scores (fatigue) differ over time.
There was a statistically significant interaction of group*time (F=154.7; p<0.001), indicating that the effect of time depends on the group.
No post-hoc tests were used to test which factor levels differ.

Linear regression analysis to analyze associations with total mean score fatigue:
Experimental group (Ref. control group) β=0.844 (p<0.001)</td>
<td>Selection bias: 0
Unclear how many were approached

Attrition bias: 1
80/80 participants completed the intervention

Detection bias: 0
No blinding

Confounding: 0
No additional confounders controlled for; description of analysis partly unclear

Total quality: 1/4

Remarks:
1. Kudubes AA, Bektas M, Ugur O. Developing a scale for the assessment of fatigue in pediatric oncology patients aged 7-12 for children and parents. Asian Pac J Cancer Prev. 2014;15(23):10199-10207.</td>
</tr>
</table>

Table S12. Evidence summaries and overall conclusions for all clinical questions.

1. What is the risk for suffering from cancer-related fatigue (CRF) in CAYA survivors?	
**Conclusion single studies**	
**Chalder Fatigue Questionnaire (FQ)**<br>Widely used questionnaire for assessment of fatigue severity and for case detection in clinical and epidemiological studies; 4-point Likert scoring for all 11 items, total fatigue defined by simple addition with higher scores implying higher levels of fatigue; two additional items ask for the duration and extent of fatigue; for the definition of *chronic fatigue* scores are dichotomized (0,0,1,1) and *chronic fatigue* is defined by a sum score of ≥4 for all 11 dichotomized items and a duration of ≥6 months.	
**30.6%** of childhood lymphoma survivors* reported chronic fatigue.<br>*n=124; median 33 years at study; median 20 years of observation time	*Johannsdottir et al. 2017*
Survivors* with CF had a mean FQ total score of 20.0, survivors without CF a mean FQ total score of 10.5 (p<0.001).<br>*n=62; Lymphoma, ALL; mean age at study 34.05 years; median 25.3 years of follow-up; follow-up study with all 62 survivors also participating in the ***Hamre et al. 2013a***	*Zeller et al. 2014*
Survivors* were significantly more fatigued than controls**: OR=4.5 (p<0.001) for having chronic fatigue. **28% of survivors had chronic fatigue (CF)**, 8% of controls had chronic fatigue.<br>*Hodgkin lymphoma (HL), non-Hodgkin lymphoma (NHL), acute lymphoblastic leukemia (ALL); n=290; median age at diagnosis 9.5 years; median age at study 29.6 years; **Norwegian population sample; n=1405, median age at study 34.0 years	*Hamre et al. 2013a*
**28% of survivors* had CF**.<br>*n=232; n=117 ALL, n=68 HL, n=47 NHL, median age at diagnosis: 9.6 years, median age at study: 29.7 years; same sample as ***Hamre et al. 2013a***	*Hamre et al. 2013b*
Total fatigue in survivors*: mean=13.9 (SD 5.3). **Cases of chronic fatigue: 27%** (n=76)<br>SF-36 domain «Vitality»: Survivors mean=51.1 (SD 21.6), controls mean=60.1 (SD 19.3) (p<0.001)<br>*n=285; diagnoses: n=91 Hodgkin lymphoma (HL), n=45 Non-Hodgkin (NHL), n=149 Acute lymphoblastic leukemia (ALL); median age at diagnosis: 10 years; Median age at study: 30 years; same sample as ***Hamre et al. 2013a;*** Age matched controls from the general population of Norway.	*Kanellopoulos et al. 2013*
**11% of the survivors* had CF**. CF was significantly more prevalent in the older group (OG; 13.6%) than in the younger group (YG; 6.8%, p<0.05). The OG also had a higher occurrence of CF relative to the general population (GP; 5.9%, p<0.001).<br>* n=398; acute myeloid leukemia (AML)>astrocytoma>Wilms tumor (WT); age at diagnosis range 1-18 years; younger group (YG) 13-18 years at study; older group (OG) ≥19 years at study; Comparison group for OG from general population (GP; n=763)	*Johannsdottir et al. 2012*
Survivors of malignant extremity bone tumors (EBT; total N=57, mean age at diagnosis male/female: 20/16 years; mean years since diagnosis male/female: 14/11) were compared with Hodgkin's disease (HD; n=89) survivors, testicular cancer (TC; n=62) survivors and the general population (NORM; n=285).<br>**14% of EBT, 21% of HD and 16% of TC survivors suffered from chronic fatigue**, compared to 10% of NORMS (p=0.30). No significant differences in the fatigue scores were observed between EBT and the other survivor groups, but EBT survivors had a **significantly higher total fatigue score compared to NORMs** (13.2 (SD 3.8) vs. 11.8 (SD 3.9), p=0.003).	*Aksnes et al. 2007*
**EORTC-QLQ-30**<br>30 items: global quality of life (2 items), five functional scales (social function (2 items), cognitive function (2 items), emotional function (4 items), role function (2 items), physical function (5 items)), three symptom scales (fatigue (3 items), nausea and vomiting (2 items), pain (2 items)) and six single items (financial problems, diarrhea, constipation, lack of appetite, insomnia, dyspnea).<br>Scores of 0-100 for every scale or single item. Global quality of life, functional scales: high values = high QOL; symptom scales & single items: high values = low QOL → **Fatigue: higher values mean higher symptoms of fatigue**	
Survivors of childhood-onset craniopharyngioma* with no hypothalamic involvement (HI) have a median score of **21**, survivors with HI a median score of **37**.<br>*n=108; median age at diagnosis: 8.1 years; median follow-up time: 16.3 years	*Sterkenburg et al. 2015*
Survivors of Hodgkin's disease* compared to controls**:<br>male survivors had mean scores of **19.02** (SD 21.7) vs. controls 7.85 (SD 14.6)<br>female survivors had mean scores of **26.57** (SD 24.8) vs. controls 14.02 (SD 20.09) (survivors had significantly more fatigue than controls, p<0.001)<br>*n=725; mean age at diagnosis: 13.63 years; mean time since diagnosis: 15.26 years; **age-adjusted sample of the German norm population	*Calaminus et al. 2014*
The mean fatigue score of the study population* was **26.6** (SD 20.1), no control group was present. Mean fatigue score was the second highest score of the four symptom scales used in this study (eg. drowsiness, communication deficit and insomnia).<br>*n=104, mean age at diagnosis 13.3 years, mean age at study 26.8 years, brain tumor survivors	*Sato et al. 2014*
Lower extremity bone tumor survivors* were significantly less fatigued (**sample mean 18.65** (SD 20.30)) than the control population (cancer survivors under the age of 50; sample mean of 33.9 (SD 26.1); p<0.001).<br>*n=28; mean age at diagnosis 11.6 years	*Barrera et al. 2012*
Survivors of deep-seated low-grade gliomas* have a mean score of **28**, the normal population 28.8 (difference not statistically significant).<br>*n=28; age at radiosurgery: median 8.3 years; years of follow-up: 134 months=11.17 years	*Korinthenberg et al. 2011*

Table S12 continued

**Fatigue subscale of the Functional Assessment of Chronic Illness Therapy – Fatigue (FACIT-Fatigue)**<br>13-item scale; validated in cancer patients; measure of physical and functional consequences of fatigue; reverse 4-point Likert scale, ranging from 0 to 52, lower scores indicate more fatigue; for dichotomization: lowest 10th percentile of the sibling scores classified as *fatigued*.	
26.7% of teenage and young adult cancer survivors* reported clinically significant levels of fatigue (scores>22**). Mean fatigue score in off-treatment survivors (n=135) was 15.56** (SD=10.98).<br>*mixed diagnoses; n=202; age at study 13-24 years; **this study did not reverse code the FACIT-Fatigue scale; the scale ranges from 0-52, but lower scores indicate less fatigue	*Fortmann et al. 2018*
**17% of Hodgkin's lymphoma survivors*** reported elevated fatigue (total score ≤30).<br>*Survivors of the childhood cancer survivor study (CCSS; n=751; 42.5% aged 11-15 years at diagnosis; at least 5 years since diagnosis)	*Rach et al. 2017*
**13.8% of survivors*** showed fatigue (cutoff score of ≤ lowest 10% of siblings was used).<br>*CCSS; mixed diagnoses; n=1426; mean age at diagnosis 11.9 years; mean age at study 35.9 years	*Clanton et al. 2011*
Survivors* had a mean fatigue score of 40.56 (SD 10.40) was significantly lower than the siblings' mean of 45.19 (SD 6.88; p=0.02), indicating more significant problems with fatigue among survivors. **16% of survivors** had fatigue scores in the clinically significant range (scores<30), compared to 3.1% in siblings, but the difference only approached statistical significance (p=0.067).<br>*n=55, mixed diagnoses; median age at diagnosis: 8 years; median current age: 56 years	***Kenney et al. 2010***
Survivors* were significantly more likely to be fatigued than their siblings**. The **prevalence of fatigue was 19.2%** in survivors (cutoff score of ≤ lowest 10% of siblings was used).<br>*CCSS; n=1897; mixed diagnoses; diagnosed before the age of 21 years; at least 5 years from diagnosis; **nearest-age siblings n=369; mean 40.8 vs. 42.0	*Mulrooney et al. 2008*
**PedsQL (Pediatric Quality of Life Inventory) Multidimensional Fatigue Scale**<br>This validated scale comprises six items about general fatigue, six items about sleep/rest fatigue, another six items about cognitive fatigue, and finalizing into a sum score of all 18 items. Age-categorized versions for the parent proxy report (age: 4, 5-7, 8-12 and 13-18 years) of the PedsQL were administered in this study. Higher scores indicate less fatigue, i.e. better fatigue-related QoL.	
Survivors of childhood acute lymphoblastic leukemia* reported greater fatigue compared with the general population. Cognitive fatigue survivors mean**: -0.75 (SD 1.2) vs. 0 (SD 1.0) expected in the general population, p=0.0003. General fatigue survivors mean*: -0.61 (SD 1.2), p=0.0003. Sleep-rest fatigue survivors mean*: -0.27 (SD 1.2), p=0.07).<br>*n=70; 1.2-17.7 years at diagnosis; mean 7.4 years since diagnosis; **fatigue scores were transformed into age-adjusted Z-scores (mean=0, SD=1.0)	*Cheung et al. 2017*
**85%** of survivors of adolescent and young adult cancer experienced fatigue during the preceding month. The fatigued survivors had a mean MFS level of 44.3 (SD=20.5).<br>*n=80; mixed diagnoses; mean 18.9 years at diagnosis; mean 22.1 years at survey	*Spathis et al. 2017*
Survivors of hematopoietic stem cell transplant (HSCT) in childhood*: Mean levels of fatigue was 69.21 (SD 20.14) for self-report and 72.15 (SD 20.79) for parent-report, indicating moderately elevated fatigue symptoms. Compared to ratings described in another study**, ratings of total fatigue in survivors of this study indicated more fatigue than in healthy peers (p<0.001), but no difference compared to children on and off treatment for cancer (p>0.05).<br>*n=76; <22 years at transplant; mean 17.8 years at study; mean 7.8 years since HSCT; ** Varni, J. W., Burwinkle, T. M., Katz, E. R., Meeske, K., & Dickinson, P. (2002). The PedsQL in pediatric cancer: Reliability and validity of the Pediatric Quality of Life Inventory Generic Core Scales, Multidimensional Fatigue Scale, and Cancer Module. Cancer, 94, 2090–2106.	*Graef et al. 2016*
**13.8%** of childhood and adolescent cancer survivors* were considered fatigued**. This did not statistically differ from the 16% (43 cases) that would have been expected based on community sample data for the MFS (p=0.467)<br>*n=268; median age at diagnosis: 6.4 years; mean time since diagnosis 13.1 years; median age at study 21.4 years; Leukemia>HL>NL>Bone tumors>soft tissue sarcoma>neuroblastoma>wilms tumor>other; **MFS score ≥1 SD below means for non-cancer patients of similar age	*Frederick et al. 2016*
Survivors of brain tumors*: Mean total MFS score 70.67 (SD 18.72). 42 of the 142 study participants had clinically significant fatigue** (**29.5%**). No control group was present.<br>*n=142, age at diagnosis mean 9.72 years (SD 4.87), mean age at study 20.24 years; **defined as MFS score >1 SD below the mean for normative samples	*Brand et al. 2016*
Survivors*: Child/Parent report «Total fatigue»: 78.73/74.25.<br>Controls**: Child/Parent report «Total fatigue»: 76.84/81.21.<br>Parents rated the ALL survivors as having more general fatigue and total fatigue than the norm. Fatigue reported by survivors themselves did not differ from the Dutch norm.<br>*Survivors of ALL (n=62; age at diagnosis 5-17 years; mean age at study: 9.7 yrs). **Controls: Dutch norm references.	*Gordijn et al. 2013*
Survivors*: Child/Parent report «Total fatigue»: **83.33/84.03**.<br>Controls**: Child/Parent report «Total fatigue»: **80.56/83.33**.<br>The controls reported significantly more total fatigue than the survivors (p<0.01).<br>Survivors scored higher on fatigue when compared with their parent proxy scores, but not statistically significantly (p>0.05).<br>*Survivors of extracranial childhood cancer (n=199; mean age at diagnosis: 3.6 years; mean age at study: 14.4 years). **Matched controls from the Finnish Population Registry.	*Mört et al. 2011*

Table S12 continued

**Checklist individual strength (CIS)**<br>20 items scored on a 7-point Likert scale; four subscales *subjective fatigue*, *concentration*, *motivation* and *physical activity*. Total score by summing up all items. Higher scores indicate more fatigue-related problems.	
Brain tumor survivors* had a higher total score of Fatigue (**63.23** (SD 21.80)) than controls** (51.76 (SD 21.88); p=0.01).<br>*n=82; mean age at diagnosis: 6.87 years; mean time of follow-up: 6.98 years; **siblings	*De Ruiter et al. 2016*
Survivors* had a a higher mean score of **81.42** (SD 20.14) than controls** 47.39 (SD 19.06, p<0.001). **26.4% of survivors** had a VAS score (Visual Analogue Scale for chronic fatigue) of ≥70mm.<br>*mixed diagnoses; n=46; median age at diagnosis: 8.1; median age at study: 29.8 years; **n=33 siblings or healthy peers as controls	*Blaauwbroek et al. 2009*
**Multidimensional Fatigue Inventory (MFI-20)**<br>**The MFI-20 questionnaire measures fatigue in 5 dimensions: *general fatigue*, *physical fatigue*, *reduced activity*, *reduced motivation* and *mental fatigue*.** The domains of MFI-20 are measured by 20 questions that are scored on a scale from 1-5. The 5 domains can have a total score of 4-20, expressed as a percentage: the higher the score, the more fatigue the participant experiences.	
Survivors of pediatric differentiated thyroid carcinoma* reported **more mental fatigue** compared to controls** (9 vs. 7, p=0.012). There were no statistically significant differences for the two groups regarding general fatigue (survivors 10 vs. controls 9, p=0.075), physical fatigue (8 vs. 6, p=0.083), reduced activity (8 vs. 8, p=0.613), reduced motivation (6 vs. 6, p=0.879), and total fatigue (41 vs. 36, p=0.129).<br>*n=67; median age at diagnosis was 15.8 years; median 17.8 years of follow-up; **n=56 controls: healthy peers	*Nies et al. 2017*
In comparison to the control group**, **survivors* scored significantly lower for general fatigue** and **reduced motivation** (p<0.05, effect size GF: -0.14, effect size RM: -0.19), **but significantly higher for mental fatigue** (p<0.05, effect size 0.15).<br>*n=416; mean age at diagnosis 8 years; mean age at study 24 years; Leukemia/Lymphoma>Solid tumor>brain/CNS tumor, **n=1026; sex and age matched, recruited via survivors GPs	*Langeveld et al. 2003*
**PROMIS V1.0 Pediatric Profile 25**<br>Pediatric Profile 25 is a collection instrument of self-reporting short forms containing items from the PROMIS domains.26. Domains used in this study included fatigue, physical and functional mobility, and depressive symptoms; each included 4 items. Fatigue and depression are scored on a 5-point Likert scale where 0 = never to 4 = almost always; the higher scores represent higher levels of fatigue and depression. Subscales are scored by summing items, with a possible range of 0 to 16.	
Pediatric cancer survivors* reported normal levels of fatigue: mean 4.1 (SD 4.0), range 0-16 (no comparison group). 22 children (**15.3%**) reported elevated levels of fatigue.<br>*n=144; mixed diagnoses; mean age at study 12.9 years, mean 5.9 years since diagnosis; no control group	*Karimi et al. 2019*
**Fatigue-scale adolescent (FS-A)**<br>The FS-A is a 14-item questionnaire that asks adolescents (age 13 to 18 years), to evaluate their fatigue experience during the previous week. Responses are rated using a 5-point Likert scale ranging from 1 to 5 (1=not at all; 2= a little; 3=some; 4= quite a bit; 5= a lot). Total possible scores range from 14 to 70. Higher scores indicate higher levels of fatigue.	
Participants were adolescent survivors of childhood cancer (CCS) and adolescent cancer patients (ACP).* CCS had a mean level of fatigue **28.6** (SD 3.7), ACP 31.3 (SD 5.2), whereas healthy controls had a mean level of 22.1 (SD 4.8; p<0.001 compared to CCS).<br>*CCS n=200/ ACP n=50; Leukemia>Lymphoma>Brain tumor; 62% >2 years since treatment completion)	*Ho et al. 2015*
**Health Knowledge Inventory**<br>One question about fatigue	
**40%** of survivors of childhood cancer*reported fatigue problems, compared to 22% of controls**. When adjusted for age and income, survivors reported significantly more fatigue compared to controls (p=0.002).<br>*n=154; Leukemia>Lymphoma>Solid tumors; ≤18 years at diagnosis; on average 12.29 years since diagnosis; mean age of 20.1 years at study; **n=170; healthy AYA controls; mean age 21.1 years at study	*Daniel et al. 2016*
**POMS (Profile of Mood State)**<br>The POMS is a 65-item self-report questionnaire designed to measure six identifiable mood states (tension/anxiety, depression, anger, confusion, vigor and fatigue) with demonstrated reliability and validity. High scores on the fatigue subscale suggest persons with low energy. Subjects are asked to describe the extent to which the adjectives describe the way they had been feeling during the past week, on a scale that ranged from 0 ("not at all") to 4 ("extremely").	
POMS fatigue-inertia mean score was 8.13 (SD=5.99) for survivors of childhood cancer*.<br>*n=104; diagnosed <18 years; average 8.4 years since diagnosis	*Lowe et al. 2016*
No significant difference in mean fatigue score between ALL survivors* and sibling controls was found (mean score 7.87 (SD 5.58) vs mean score 8.36 (SD 5.83), t-test p=0.19).<br>*n=580; diagnosed <20 years; at least 2 years from diagnosis	*Zeltzer et al. 1997*

Table S12 continued

**Quality of Life-Cancer survivors questionnaire**<br>Fatigue was measured as part of the physical subscale of the Qualtiy of Life-Cancer survivors questionnaire (scale 0(severe problem)-10(no problem))	
Participants were childhood cancer survivors*. Fatigue was the symptom with the lowest score in this subscale (mean score 7.32), which indicates that fatigue was experienced as the most problematic symptom relative to other symptoms included in the physical subscale (e.g. nausea, aches and pain, constipation, appetite changes, sleep changes and menstrual/fertility changes). No control group was present.<br>*n=176; mean 8.5 years at dx; Leukemia>Lymphoma>Sarcomas; mean time since dx 13.3 years	*Zebrack et al. 2002*
**Revised-Class Play (RCP)**	
This study compared children who survived a brain tumor* with a peer control group. Peers nominated the children surviving a brain tumor significantly more often as fatigued than the control group (mean score for survivors 0.90 vs mean score of control group -0.24, p<0.001).<br>*n=28; average time since diagnosis 36 months	*Vannatta et al. 1998*
**Revised-Piper Fatigue Scale (R-PFS)**<br>The Piper Fatigue Scale is composed of 22 numerically scaled, 0-10 items that measure four dimensions of subjective fatigue: behavioral/severity (6 items), affective meaning (5 items), sensory (5 items), and cognitive/mood (6 items). These 22 items are used to calculate the four subscale/dimensional scores and the total fatigue scores. Subscales are scored by summing up items and dividing by number of items (0-10 subscale score). Total fatigue score is calculated by adding the 22 item scores together and divide by 22 (0-10 total score). Higher scores indicate higher levels of fatigue.	
Survivors of childhood leukemia (n=161; average age at diagnosis: 7.4 years; average time since end of treatment 13.9 years).<br>Symptom distress scale (SDS): **Fatigue was the most frequently reported symptom (61%)**.<br>POMS: Survivors' average **POMS fatigue-inertia score was 7.2** (SD 6.3), which is within the normal range reported for college students.<br>SF-36: Survivors' **SF-36 vitality mean score was 63.4**, which is slightly higher (more energy) than the norms for the general population (61.3).<br>R-PFS: **Prevalence of fatigue was 30%**.	*Meeske et al. 2005*

Table S12 continued

Non-standardized measurement tool	
**29.7%** of survivors of acute lymphoblastic leukemia* reported fatigue.<br>*ALL; n=61; mean age at study 6.4 years; mean 2.6 years since treatment	*Arpaci & Kilicarslan Toruner 2016*
Survivors of Hodgkin Lymphoma* reported on four items**: “felt tired” mean 2.73, “had trouble finishing tasks because tired quickly” mean 3.46; “needed to sleep during the day” mean 3.25, “frustrated by being too tired to do things he/she wanted to do” mean 3.54, “needed to limit social activities because of fatigue” mean 3.68.<br>*n=103; mean age at diagnosis 15.5 years; 36 months post therapy; **(0=”very much so”-4”not at all”)	*Macpherson et al. 2015*
Fatigue was determined in **21.6%** of childhood acute lymphoblastic leukemia survivors*. Of those, 60% Grade 1/mild, 31% Grade 2/moderate, 9% Grade 3/severe Fatigue.<br>*n=162; median age at diagnosis: 3.9 years; median time from diagnosis: 10.2 years	*Khan et al. 2014*
**25.78%** of childhood cancer survivors* suffer from Fatigue.<br>*n=225; hematologic cancers>solid or soft tissue tumors>CNS or brain tumors; mean age at diagnosis: 9.89 years; mean time since diagnosis 12.03 years	*Yi et al. 2014*
**52%** of childhood cancer survivors reported fatigue. Of those, 36% reported their fatigue was severe enough to limit work activities.<br>*n=42; Leukemia>CNS>Lymphoma>Hodgkin’s lymphoma; mean age at diagnosis 9.8 years; mean time of follow-up: 8.9 years	*Berg et al. 2013*
Overall incidence of fatigue in survivors* was **30%**, but brain tumor survivors reported 47%.<br>*mixed diagnoses; n=271; Mean age at diagnosis: 10 years, mean age at survey: 24 years	*McClellan et al. 2013*
**50% of craniopharyngioma survivors* reported fatigue**<br>*n=28; median age at diagnosis: 8 years; age at study: 29.7 years	*Manley et al. 2012*
12 items, 0-3 Likert scale (0= not at all; 3= every day; Total score 0-36)<br>**Survivors* scored significantly lower than controls**** in total fatigue (9.8 vs. 11.4).<br>Childhood leukemia survivors had equal or less fatigue compared with that of their age- and gender matched controls in multidimensional aspects of fatigue.<br>*n=81, diagnoses: ALL and AML, age at diagnosis: mean 6.7 years; age at study: mean 14.1 years; **n=243 healthy controls	*Nagai et al. 2012*
**24% of survivors*** reported fatigue.<br>*n=25; about half acute lymphoblastic leukemia; mean age at diagnosis 5.2 years; mean age at study 14.0 years	*Berg et al. 2009*
**10.2%** of childhood cancer survivors* suffered from Fatigue. Of those, 19% Grade 1, 75% Grade 2, 6% Grade 3/4/5.<br>*n=1284; Leukemia>Lymphoma>Kidney/Wilms tumor>Soft tissue sarcoma; median follow-up time: 17 years	*Geenen et al. 2007*
**67%** of adolescents and young adults off treatment* experienced fatigue.<br>*Leukemia>Lymphoma>Brain tumor; mean age at study 16 years	*Enskär et al. 2007*
**67%** of Hodgkin’s disease survivors* reported feeling fatigued. 35% stated that it was a moderate to severe problem.<br>*n=48; median age at diagnosis: 16.5 years; median 14.3 years	*Adams et al. 2004*
**Overall conclusion**	
**Prevalence of CRF**<br>There is evidence that survivors of childhood, adolescent and young adult cancers are at risk for CRF. In 28 studies the prevalence of CRF in CAYACS ranged from 10 to 85%.	28 studies<br>(24 samples)<br>**Level A**
**Prevalence of CRF in CAYACS versus controls**<br>Some evidence suggests that there is an increased risk for CRF in survivors of childhood, adolescent and young adult cancers as compared to controls. In 5 studies, there was a higher prevalence of CRF in survivors compared to controls with a difference ranging from 5 to 20%. One study reported lower prevalence of CRF in survivors compared to community norms.	6 studies<br>**Level C**
**Levels of CRF in CAYACS versus controls**<br>Evidence suggests that survivors of childhood, adolescent and young adult cancers have higher levels of CRF compared to controls. In 12 studies, survivors had significantly higher levels of CRF compared to controls. Two studies reported lower levels of CRF in survivors compared to controls.	18 studies<br>**Level B**

Table S12 continued

1.1 What is the risk of CRF in CAYA cancer survivors **by sex**?	
**Conclusion single studies**	
Multivariable linear regression analysis* showed that females are at significantly higher risk for CRF:<br>• **Female vs. male: β=0.19, p<0.001**<br>Childhood cancer survivors (n=416; mean age at diagnosis 8 years; mean age at study 24 years; Leukemia/Lymphoma>Solid tumor>brain/CNS tumor); *adjusted for age at study, marital status, educational achievement, employment, age at diagnosis, diagnosis, treatment duration, follow-up time, late effects, treatment, and depression	*Langeveld et al. 2003*
Multivariable logistic regression* showed that females are at significantly higher risk for CRF:<br>• **Female vs. male: RR=2.77 (95%CI:1.94-3.94)**<br>Childhood cancer survivors (n=1284; Leukemia>Lymphoma>Kidney/WT>Soft tissue sarcoma; median follow-up time: 17 years; median age of 24.4 years); *adjusted for radiation, TBI, chemotherapy, surgery, follow-up duration, and age at diagnosis	*Geenen et al. 2007*
Multivariable logistic regression analysis* showed that females are at significantly higher risk for CRF:<br>• **Female vs. male: OR=2.1 (95%CI:1.6-2.7)**<br>Childhood cancer survivors (CCSS; n=1897; mixed diagnoses; diagnosed before the age of 21 years; at least 5 years from diagnosis); *adjusted for heart failure, lung fibrosis, hypothyroidism, depression, BMI, marital status, employment status, and infant at home	*Mulrooney et al. 2008*
Multivariable logistic regression analysis* showed no significant association between sex and total fatigue:<br>• Female vs. male: β=2.99, p>0.05<br>Survivors of extracranial childhood cancer (n=199; mean age at diagnosis: 3.6 years; mean age at study: 14.4 years). **Lower scores of the effect measure indicate more fatigue.** *adjusted for age at study, diagnosis, treatment, follow-up time, additional diagnosis, remedial education, overall average grade, happiness, and HRQoL	*Mört et al. 2011*
Multivariable logistic regression analysis* showed no significant association between sex and CRF:<br>• Female vs. male: OR=1.54 (95%CI:0.94-2.54)<br>Childhood cancer survivors (only n=33 from older group (≥19 years) included for risk factor analysis); AML >astrocytoma>WT; age at diagnosis range 1-18 years;); *adjusted for age at study, educational achievement, marital status, employment, and receiving social benefits	*Johannsdottir et al. 2012*
Multivariable linear regression analysis* showed no significant association between sex and total fatigue:<br>• Female (Ref. Male): β=0.35, p>0.05<br>Survivors of childhood leukemia (n=81, diagnoses: ALL and AML, age at diagnosis: mean 6.7 years; age at study: mean 14.1 years); *adjusted for age at study, diagnosis, cranial irradiation, TBI, and follow-up time	*Nagai et al. 2012*
Multivariable logistic regression analysis* showed no significant association between sex and CRF:<br>• Female vs. male: OR=0.8 (95%CI:0.46-1.5), p=0.6<br>Childhood cancer survivors (n=290; HL, NHL, ALL; median age at diagnosis 9.5 years; median age at study 29.6 years); *adjusted for diagnosis, age at survey, treatment era, thyroid status, HADS (Hospital Anxiety and Depression scale) total score	*Hamre et al. 2013a*
Multivariable logistic regression analysis* showed no significant association between sex and CRF:<br>• Female gender OR=1.09 (95%CI: 0.6-1.9), p=0.8<br>Childhood cancer survivors (n=232; HL, NHL, ALL; median age at diagnosis 9.6 years; median age at study 29.7 years; same sample as ***Hamre et al. 2013a***); *adjusted for age at survey, diagnosis, smoking, BMI, analgesics use, heart function, T-cell origin, CNS-irradiation, and B-symptoms at diagnosis	*Hamre et al. 2013b*
Multivariable logistic regression analysis* showed no significant association between sex and CRF:<br>• Female vs. male: OR=1.39 (95%CI:0.69-2.81), p=0.348<br>Childhood and adolescent cancer survivors (n=268; median age at diagnosis: 6.4 years; mean time since diagnosis 13.1 years; median age at study 21.4 years; Leukemia>HL>NL>Bone tumors>soft tissue sarcoma>neuroblastoma>WT>other); *adjusted for age at study, income, survival time, and chronic conditions	*Frederick et al. 2016*
Multivariable logistic regression* showed that females were at higher risk for fatigue:<br>• **Female (Ref. Male) OR=4.75 (95%CI:2.47-9.15), p<0.001**<br>Hodgkin's lymphoma survivors of the childhood cancer survivor study (CCSS; n=751; 42.5% aged 11-15 years at diagnosis; at least 5 years since diagnosis); *adjusted for sex, emotional distress, employment, pain, physical function, and BMI	*Rach et al. 2017*
Hierarchical linear regression* showed no significant association between gender and CRF:<br>• Gender: β=0.008, p=0.895<br>**Pediatric cancer survivors (n=144; mixed diagnoses; mean age at study 12.9 years, mean 5.9 years since diagnosis); *adjusted for age, sex, race, time since dia**gnosis, diagnosis, chemotherapy, radiation, depression, parent reported depression/anxiety, BMI, physical and function mobility	*Karimi et al. 2019*
**Overall conclusion**	
Some evidence suggests that **female sex** is associated with an **increased risk for CRF** in survivors of childhood, adolescent and young adult cancers.	10 studies (9 samples)<br>**Level C**

Table S12 continued

1.2 What is the **risk of CRF** in CAYA cancer survivors **by age at follow-up**?	
**Conclusion single studies**	
Multivariable regression analysis* showed no significant association between age at follow-up and CRF:<br>• Age at follow-up: β=0.01, p>0.05<br>Childhood cancer survivors (n=416; mean age at diagnosis 8 years; mean age at study 24 years; Leukemia/Lymphoma>Solid tumor>brain/CNS tumor); *adjusted for sex, marital status, educational achievement, employment, age at diagnosis, diagnosis, treatment duration, follow-up time, late effects, treatment, and depression	*Langeveld et al. 2003*
Multivariable regression analysis* showed that older age at follow-up was significantly associated with an increased risk of total fatigue:<br>• **Age at study: β= -1.87, p<0.001**<br>Survivors of extracranial childhood cancer (n=199; mean age at diagnosis: 3.6 years; mean age at study: 14.4 years). **Lower scores of the effect measure indicate more fatigue**. *adjusted for sex, diagnosis, treatment, follow-up time, additional diagnosis, remedial education, overall average grade, happiness, and HRQoL	*Mört et al. 2011*
Multivariable logistic regression analysis* showed that older age at follow-up was significantly associated with an increased risk of CRF:<br>• **Age at assessment: OR=1.08 (95%CI:1.01-1.16)**<br>Childhood cancer survivors (only n=33 from older group (≥19 years) included for risk factor analysis); AML >astrocytoma>WT; age at diagnosis range 1-18 years;); *adjusted for sex, educational achievement, marital status, employment, and receiving social benefits	*Johannsdottir et al. 2012*
Multivariable logistic regression analysis* showed that older age at follow-up was significantly associated with an increased risk of total fatigue:<br>• **Present age (years): β=0.24, p<0.05**<br>Childhood cancer survivors (n=81, diagnoses: ALL and AML, age at diagnosis: mean 6.7 years; age at study: mean 14.1 years); *adjusted for sex, diagnosis, cranial irradiation, TBI, and follow-up time	*Nagai et al. 2012*
Multivariable logistic regression analysis* showed no significant association between age at follow-up and CRF:<br>• Age at survey: OR=1.05 (95%CI:1.0-1.1), p=0.1<br>Childhood cancer survivors (n=290; HL, NHL, ALL; median age at diagnosis 9.5 years; median age at study 29.6 years); *adjusted for diagnosis, treatment era, sex, thyroid status, HADS (Hospital Anxiety and Depression scale) total score	*Hamre et al. 2013a*
Multivariable logistic regression analysis* showed that older age at survey was associated with an increased risk for CRF:<br>• **Age: OR=1.04 (95% CI: 1.00–1.1) p=0.03**<br>Childhood cancer survivors (n=232; HL, NHL, ALL; median age at diagnosis 9.6 years; median age at study 29.7 years; same sample as ***Hamre et al. 2013a***); *adjusted for sex, diagnosis, smoking, BMI, analgesics use, heart function, T-cell origin, CNS-irradiation, and B-symptoms at diagnosis	*Hamre et al. 2013b*
Multivariable logistic regression analysis* showed no significant association between age at follow-up and CRF:<br>• Age at survey: 16-19 years (Ref. 12-15 years) OR=0.27 (95%CI:0.05-1.39)<br>• Age at survey: 20-29 years (Ref. 12-15 years) OR=1.36 (95%CI:0.54-3.47)<br>• Age at survey: 30-39 years (Ref. 12-15 years) OR=2.06 (95%CI:0.58-7.27)<br>• Age at survey: 40-49 years (Ref. 12-15 years) OR=3.68 (95%CI:0.49-27.49)<br>Childhood and adolescent cancer survivors (n=268; median age at diagnosis: 6.4 years; mean time since diagnosis 13.1 years; median age at study 21.4 years; Leukemia>HL>NL>Bone tumors>soft tissue sarcoma>neuroblastoma>WT>other); *adjusted for sex, income, survival time, and chronic conditions	*Frederick et al. 2016*
Hierarchical linear regression* showed no significant association between age at survey and CRF:<br>• Age at survey: β=-0.005, p=0.935<br>Pediatric cancer survivors (n=144; mixed diagnoses; mean age at study 12.9 years, mean 5.9 years since diagnosis); *adjusted for age, sex, race, time since diagnosis, diagnosis, chemotherapy, radiation, depression, parent reported depression/anxiety, BMI, physical and function mobility	*Karimi et al. 2019*
**Overall conclusion**	
Evidence suggests that **older age at follow-up** is associated with an **increased risk for CRF** in survivors of childhood, adolescent and young adult cancers.	8 studies (7 samples)<br>**Level B**

Table S12 continued

1.3 What is the **risk of CRF** in CAYA cancer survivors **by age at diagnosis**?	
**Conclusion single studies**	
Multivariable regression analysis* showed no significant association between age at diagnosis and CRF:<br>• Age at diagnosis: β=0.06, not significant<br>Childhood cancer survivors (n=416; mean age at diagnosis 8 years; mean age at study 24 years; Leukemia/Lymphoma>Solid tumor>brain/CNS tumor); *adjusted for sex, age at study, marital status, educational achievement, employment, diagnosis, treatment duration, follow-up time, late effects, treatment, and depression	*Langeveld et al. 2003*
Multivariate logistic regression analysis* showed no significant association between age at diagnosis and CRF:<br>• Age at diagnosis: 0-4 years (Ref. 15+ years): OR= 0.7 (95%CI:0.4-1.2)<br>• Age at diagnosis: 5-9 years (Ref. 15+ years): OR=0.9 (95%CI:0.6-1.4)<br>• Age at diagnosis: 10-14 years (Ref. 15+ years): OR=0.8 (95%CI:0.6-1.1)<br>Survivors (CCSS; n=1897; mixed diagnoses; diagnosed before the age of 21 years; at least 5 years from diagnosis); *adjusted for age at diagnosis, radiation, and chemotherapy	*Mulrooney et al. 2008*
Univariable logistic regression showed no significant association between age at diagnosis and CRF (variable was therefore not included in the multivariable model):<br>• Age at diagnosis: not significant<br>Childhood and adolescent cancer survivors (n=268; median age at diagnosis: 6.4 years; mean time since diagnosis 13.1 years; median age at study 21.4 years; Leukemia>HL>NL>Bone tumors>soft tissue sarcoma>neuroblastoma>WT>other).	*Frederick et al. 2016*
**Overall conclusion**	
Evidence suggests that **age at diagnosis** is **not significantly associated** with the risk for **CRF** in survivors of childhood, adolescent and young adult cancers.	3 studies<br>**Level B**

1.4 What is the **risk of CRF** in CAYA cancer survivors **by time since diagnosis**?	
**Conclusion single studies**	
Multivariable regression analysis* showed no significant association between years since completion of therapy and CRF:<br>• Years since completion of therapy: β=0.02, p>0.05<br>Childhood cancer survivors (n=416; mean age at diagnosis 8 years; mean age at study 24 years; Leukemia/Lymphoma>Solid tumor>brain/CNS tumor); *adjusted for sex, age at study, marital status, educational achievement, employment, age at diagnosis, diagnosis, treatment duration, late effects, treatment, and depression	*Langeveld et al. 2003*
Multivariable regression analysis* showed no significant association between follow-up time and total fatigue:<br>• Length of survival: More than 10 years (Ref. 10 years or less) β= -3.6, p>0.05<br>Survivors of extracranial childhood cancer (n=199; mean age at diagnosis: 3.6 years; mean age at study: 14.4 years). **Lower scores of the effect measure indicate more fatigue**. *adjusted for age at study, sex, diagnosis, treatment, additional diagnosis, remedial education, overall average grade, happiness, and HRQoL	*Mört et al. 2011*
Multiple regression analysis* showed that longer duration after completion of treatment was significantly associated with a decreased risk of CRF:<br>• **Duration after completion of treatment (years): β= -0.45, p<0.05**<br>Survivors (n=81, diagnoses: ALL and AML, age at diagnosis: mean 6.7 years; age at study: mean 14.1 years); *adjusted for age at study, sex, diagnosis, cranial irradiation, and TBI	*Nagai et al. 2012*
Multivariable logistic regression* showed no significant association of survival time with risk for CRF:<br>• Survival time: 10-14 years (Ref. 2-9 years) OR=0.83 (95%CI:0.32-2.18)<br>• Survival time: 15-19 years (Ref. 2-9 years) OR=1.33 (95%CI:0.45-3.91)<br>• Survival time: 20-24 years (Ref. 2-9 years) OR=0.55 (95%CI:0.14-2.15)<br>• Survival time: 25-29 years (Ref. 2-9 years) OR=0.34 (95%CI:0.05-2.17)<br>• Survival time: 30+ years (Ref. 2-9 years) OR=0.83 (95%CI:0.14-5.16)<br>Childhood and adolescent cancer survivors (n=268; median age at diagnosis: 6.4 years; mean time since diagnosis 13.1 years; median age at study 21.4 years; Leukemia>HL>NL>Bone tumors>soft tissue sarcoma>neuroblastoma>WT>other); *adjusted for sex, age at study, income, and chronic conditions	*Frederick et al. 2016*
Hierarchical linear regression* showed that shorter time since diagnosis was associated with higher levels of CRF:<br>• **Time since diagnosis: β=-0.154, p=0.019**<br>Pediatric cancer survivors (n=144; mixed diagnoses; mean age at study 12.9 years, mean 5.9 years since diagnosis); *adjusted for age, sex, race, time since diagnosis, diagnosis, chemotherapy, radiation, depression, parent reported depression/anxiety, BMI, physical and function mobility	*Karimi et al. 2019*
**Overall conclusion**	
Some evidence suggests that **longer time since diagnosis** is associated with a **decreased risk for CRF** in survivors of childhood, adolescent and young adult cancers.	5 studies<br>**Level C**

Table S12 continued

1.5 What is the **risk of CRF** in CAYA cancer survivors by **ethnicity**?	
**Conclusion single studies**	
Univariable logistic regression showed no significant association of ethnicity and risk for CRF (variable was therefore not included in the multivariable model):<br>• Ethnicity: not significant<br>Childhood and adolescent cancer survivors (n=268; median age at diagnosis: 6.4 years; mean time since diagnosis 13.1 years; median age at study 21.4 years; Leukemia>HL>NL>Bone tumors>soft tissue sarcoma>neuroblastoma>WT>other).	*Frederick et al. 2016*
**Overall conclusion**	
Some evidence suggests that **ethnicity** is **not significantly associated** with the risk for **CRF** in survivors of childhood, adolescent and young adult cancers.	2 studies<br>**Level C**

1.6 What is the **risk of CRF** in CAYA cancer survivors **by partnership status**?	
**Conclusion single studies**	
Multivariable regression analysis* showed no significant association of marital status and CRF:<br>• Married vs. not married: β=0.04, p>0.05<br>Childhood cancer survivors (n=416; mean age at diagnosis 8 years; mean age at study 24 years; Leukemia/Lymphoma>Solid tumor>brain/CNS tumor); *adjusted for sex, age at study, educational achievement, employment, age at diagnosis, diagnosis, treatment duration, follow-up time, late effects, treatment, and depression	*Langeveld et al. 2003*
Multivariable logistic regression* showed that being married is associated with a lower risk for CRF:<br>• **Married vs. not married: OR=0.11, 95%CI:0.02-0.50**<br>Survivors of childhood leukemia (n=161; average age at diagnosis: 7.4 years; average time since end of treatment 13.9 years); *adjusted for having children, sleep problems, pain, obesity, neuro-cognitive impairment, exercise-induced symptoms, unemployment, and relapse	*Meeske et al. 2005*
Multivariable logistic regression analysis* showed that not being married was associated with an increased risk of CRF:<br>• **Marital status: Not married (Ref. Married): OR=2.7, 95%CI:2.0-3.6**<br>Childhood cancer survivors (CCSS; n=1897; mixed diagnoses; diagnosed before the age of 21 years; at least 5 years from diagnosis); *adjusted for sex, heart failure, lung fibrosis, hypothyroidism, depression, BMI, employment status, and infant at home	*Mulrooney et al. 2008*
Multivariable logistic regression analysis* showed no significant association of marital status/cohabiting and CRF:<br>• **Married/cohabiting: Yes (vs. No): OR=1.09 (95%CI:0.64-1.85)**<br>Childhood cancer survivors (only n=33 from older group (≥19 years) included for risk factor analysis); AML >astrocytoma>WT; age at diagnosis range 1-18 years;); *adjusted for age at study, sex, educational achievement, employment, and receiving social benefits	*Johannsdottir et al. 2012*
Univariable logistic regression analysis showed no significant association between partnership and CRF (variable was therefore not included in the multivariable model):<br>• Partnership: p>0.05<br>Childhood cancer survivors (n=290; HL, NHL, ALL; median age at diagnosis 9.5 years; median age at study 29.6 years).	*Hamre et al. 2013a*
**Overall conclusion**	
Some evidence suggests that **not being married** is associated with an **increased risk for CRF** in survivors of childhood, adolescent and young adult cancers.	5 studies<br>**Level C**

1.7 What is the **risk of CRF** in CAYA cancer survivors **who have children?**	
**Conclusion single studies**	
Multivariable logistic regression analysis* showed that having children was associated with an increased risk for CRF:<br>• **Children (vs. no children): OR=5.80 (95%CI:1.30-25.82)**<br>Survivors of childhood leukemia (n=161; average age at diagnosis: 7.4 years; average time since end of treatment 13.9 years). *adjusted for marital status, sleep problems, pain, obesity, neuro-cognitive impairment, exercise-induced symptoms, unemployment, and relapse	*Meeske et al. 2005*
Multivariable logistic regression analysis* showed no significant association of having an infant at home and CRF:<br>• Infant at home <6 months old: Yes (Ref. No): OR=1.9 (95%CI:0.7-5.0)<br>Childhood cancer survivors (CCSS; n=1897; mixed diagnoses; diagnosed before the age of 21 years; at least 5 years from diagnosis); *adjusted for sex, heart failure, lung fibrosis, hypothyroidism, depression, BMI, marital status, and employment status	*Mulrooney et al. 2008*
**Overall conclusion**	
Some evidence suggests that **having children** is associated with an **increased risk for CRF** in survivors of childhood, adolescent and young adult cancers.	2 studies<br>**Level C**

Table S12 continued

<table>
<tr><th colspan="2">1.8 What is the risk of CRF in CAYA cancer survivors by education?</th></tr>
<tr><td colspan="2">Conclusion single studies</td></tr>
<tr><td>Multivariable regression analysis* showed no significant association of education level and CRF:
• Higher education level (vs. lower): β=0.03, p>0.05
Childhood cancer survivors (n=416; mean age at diagnosis 8 years; mean age at study 24 years; Leukemia/Lymphoma>Solid tumor>brain/CNS tumor); *adjusted for sex, age at study, marital status, employment, age at diagnosis, diagnosis, treatment duration, follow-up time, late effects, treatment, and depression</td><td>Langeveld et al. 2003</td></tr>
<tr><td>Multivariate regression* showed no significant association between educational outcomes and total fatigue:
• Remedial education: No (Ref. Yes) β= -1.43, p>0.05
• Overall average grade: β=2.47, p>0.05
Survivors of extracranial childhood cancer (n=199; mean age at diagnosis: 3.6 years; mean age at study: 14.4 years). Lower scores of the effect measure indicate more fatigue. *adjusted for age at study, sex, diagnosis, treatment, follow-up time, additional diagnosis, happiness, and HRQoL</td><td>Mört et al. 2011</td></tr>
<tr><td>Multivariable logistic regression analysis* showed no significant association between academic education and CRF:
• Academic education: Yes (vs. No): OR 0.63 (95% CI 0.36-1.12)
Childhood cancer survivors (only n=33 from older group (≥19 years) included for risk factor analysis); AML >astrocytoma>WT; age at diagnosis range 1-18 years;); *adjusted for age at study, sex, marital status, employment, and receiving social benefits</td><td>Johannsdottir et al. 2012</td></tr>
<tr><td>Univariable logistic regression analysis showed no significant association of level of education and CRF (variable was therefore not included in the multivariable model):
• Education: p>0.05
Childhood cancer survivors (n=290; HL, NHL, ALL; median age at diagnosis 9.5 years; median age at study 29.6 years).</td><td>Hamre et al. 2013a</td></tr>
<tr><td colspan="2">Overall conclusion</td></tr>
<tr><td>Evidence suggests that level of education, overall average grade and remedial education are not significantly associated with the risk for CRF in survivors of childhood, adolescent and young adult cancers.</td><td>4 studies
Level B</td></tr>
</table>

<table>
<tr><th colspan="2">1.9 What is the risk of CRF in CAYA cancer survivors by household income?</th></tr>
<tr><td colspan="2">Conclusion single studies</td></tr>
<tr><td>Multivariable logistic regression* showed no significant association between household income and CRF:
• Household income: Less than $49,999 (Ref. $100,000 and greater) OR=1.29 (95%CI:0.52-3.19)
• Household income: $50-99,999 (Ref. $100,000 and greater) OR=2.16 (95%CI:0.98-4.76)
Childhood and adolescent cancer survivors (n=268; median age at diagnosis: 6.4 years; mean time since diagnosis 13.1 years; median age at study 21.4 years; Leukemia>HL>NL>Bone tumors>soft tissue sarcoma>neuroblastoma>wilms tumor>other); *adjusted for sex, age at study, survival time, and chronic conditions</td><td>Frederick et al. 2016</td></tr>
<tr><td colspan="2">Overall conclusion</td></tr>
<tr><td>Some evidence suggests that household income is not significantly associated with the risk for CRF in survivors of childhood, adolescent and young adult cancers.</td><td>1 study
Level C</td></tr>
</table>

Table S12 continued

1.10 What is the **risk of CRF** in CAYA cancer survivors **by employment status**?	
**Conclusion single studies**	
Multivariable regression analysis* showed that being employed was significantly associated with a decreased risk of CRF and found no significant association between being a student or homemaker and CRF:<br>• Student/homemaker vs. unemployed: β= -0.12, p>0.05<br>• **Employed vs. unemployed: β= -0.20, p<0.05**<br>Childhood cancer survivors (n=416; mean age at diagnosis 8 years; mean age at study 24 years; Leukemia/Lymphoma>Solid tumor>brain/CNS tumor); *adjusted for sex, age at study, marital status, educational achievement, age at diagnosis, diagnosis, treatment duration, follow-up time, late effects, treatment, and depression	*Langeveld et al. 2003*
Survivors of childhood leukemia (n=161; average age at diagnosis: 7.4 years; average time since end of treatment 13.9 years).<br>Multivariate logistic regression (adjusted for marital status, having children, sleep problems, pain, obesity, neuro-cognitive impairment, exercise-induced symptoms, and relapse) showed that not working or attending school was significantly associated with an increased risk of CRF:<br>• **Not working or attending school: p<0.05 (effect measure not reported)**	*Meeske et al. 2005*
Childhood cancer survivors (CCSS; n=1897; mixed diagnoses; diagnosed before the age of 21 years; at least 5 years from diagnosis).<br>Multivariate logistic regression analysis (adjusted for sex, heart failure, lung fibrosis, hypothyroidism, depression, BMI, marital status, and infant at home) showed no significant association between employment status and CRF:<br>• Not working full time (Ref. working full time): OR=1.2 (95%CI:0.3-1.6)	*Mulrooney et al. 2008*
Multivariable logistic regression analysis (adjusted for age at study, sex, educational achievement, marital status, and receiving social benefits) showed no significant association between being gainfully employed and CRF:<br>• Gainfully employed: Yes (vs. No): OR=1.18 (95%CI:0.67-2.07)<br>Childhood cancer survivors (only n=33 from older group (≥19 years) included for risk factor analysis); AML >astrocytoma>WT; age at diagnosis range 1-18 years;);	*Johannsdottir et al. 2012*
Hodgkin's lymphoma survivors of the childhood cancer survivor study (CCSS; n=751; 42.5% aged 11-15 years at diagnosis; at least 5 years since diagnosis). Multivariable logistic regression (adjusted for sex, emotional distress, employment, pain, physical function, and BMI) showed that **unemployed was associated with an increased risk for CRF:**<br>• **Unemployed (Ref. employed) OR=2.90 (95%CI:1.27-6.62, p<0.01)**	*Rach et al. 2017*
**Overall conclusion**	
Evidence suggests that **being employed** or attending school is associated with a **decreased risk of CRF** in survivors of childhood, adolescent and young adult cancers.	5 studies<br>**Level B**

1.11 What is the **risk of CRF** in CAYA cancer survivors **by social benefits**?	
**Conclusion single studies**	
Multivariable logistic regression analysis (adjusted for age at study, sex, educational achievement, marital status, and employment) showed no significant association between receiving social benefits and CRF:<br>• Receiving social benefits: Yes (vs. No): OR=1.79 (95%CI:0.61-5.26)<br>Childhood cancer survivors (only n=33 from older group (≥19 years) included for risk factor analysis); AML >astrocytoma>WT; age at diagnosis range 1-18 years;);	*Johannsdottir et al. 2012*
**Overall conclusion**	
Some evidence suggests that **receiving social benefits** is **not significantly associated** with the risk of **CRF** in survivors of childhood, adolescent and young adult cancers.	1 study<br>**Level C**

Table S12 continued

1.12 What is the **risk of CRF** in CAYA cancer survivors **by amount of exercise**?	
**Conclusion single studies**	
Multiple logistic regression analysis* showed no significant association between number of steps per day and CRF:<br>• Number of steps per day: p>0.05 (effect measure not reported)<br>Childhood cancer survivors (n=62; Lymphoma, ALL; mean age at study 34.05 years; median 25.3 years of follow-up; follow-up study with all 62 survivors also participating in the ***Hamre et al. 2013a***); *adjusted for insomnia, PHQ9 score, pain, and depressive symptoms	*Zeller et al. 2014*
Generalized estimation equation* showed no significant association between amount of exercise and CRF:<br>• "[…] amount of exercise was not predictive of fatigue at end of therapy or at 12 or 36 months post-therapy (p>0.05)."<br>Survivors of Hodgkin Lymphoma (n=103; mean age at diagnosis 15.5 years; 36 months post therapy); *adjusted for sex, age at diagnosis, stage at diagnosis and protocol treatment arm	*Macpherson et al. 2015*
**Overall conclusion**	
Evidence suggests that **amount of exercise** is **not significantly associated** with the risk of **CRF** in survivors of childhood, adolescent and young adult cancers.	2 studies<br>**Level B**

1.13 What is the **risk of CRF** in **overweight/obese** CAYA cancer survivors?	
**Conclusion single studies**	
Multivariable logistic regression* showed that obesity was significantly associated with an increased risk for CRF:<br>• **Obesity: OR=3.80 (95%CI:1.41-10.26)**<br>Survivors of childhood leukemia (n=161; average age at diagnosis: 7.4 years; average time since end of treatment 13.9 years); *adjusted for marital status, having children, sleep problems, pain, neuro-cognitive impairment, exercise-induced symptoms, unemployment, and relapse	*Meeske et al. 2005*
Multivariate logistic regression analysis* showed no significant association between obesity and CRF:<br>• BMI 30+ kg/m^2: Yes (Ref. No): OR=1.3 (95%CI:0.9-1.7)<br>Childhood cancer survivors (CCSS; n=1897; mixed diagnoses; diagnosed before the age of 21 years; at least 5 years from diagnosis); *adjusted for sex, heart failure, lung fibrosis, hypothyroidism, depression, marital status, employment status, and infant at home	*Mulrooney et al. 2008*
Univariable logistic regression showed no significant association between BMI and CRF (variable was therefore not included in the multivariable model):<br>• BMI: not significant<br>Childhood cancer survivors (n=290; HL, NHL, ALL; median age at diagnosis 9.5 years; median age at study 29.6 years).	*Hamre et al. 2013a*
Multivariable logistic regression analysis* showed no significant association between BMI and CRF:<br>• BMI OR=1.1 (95%CI:1.0-1.1), p=0.1<br>Childhood cancer survivors (n=232; HL, NHL, ALL; median age at diagnosis 9.6 years; median age at study 29.7 years; same sample as ***Hamre et al. 2013a***); (adjusted for age at study, sex, diagnosis, smoking, analgesics use, heart function, T-cell origin, CNS-irradiation, and B-symptoms at diagnosis)	*Hamre et al. 2013b*
Multivariable logistic regression* showed no significant association between overweight/obesity and CRF:<br>• BMI: Overweight (Ref. Normal) OR=0.95 (95%CI:0.50-1.79, n.s.)<br>• BMI: Obese (Ref. Normal) OR=1.06 (95%CI:0.52-2.15, n.s.)<br>Hodgkin's lymphoma survivors of the CCSS (n=751; 42.5% aged 11-15 years at diagnosis; at least 5 years since diagnosis); *adjusted for sex, emotional distress, employment, pain, physical function, and BMI	*Rach et al. 2017*
Hierarchical linear regression* showed no significant association between BMI and CRF:<br>• BMI: β=-0.036, p=0.560<br>Pediatric cancer survivors (n=144; mixed diagnoses; mean age at study 12.9 years, mean 5.9 years since diagnosis); *adjusted for age, sex, race, time since diagnosis, diagnosis, chemotherapy, radiation, depression, parent reported depression/anxiety, BMI, physical and function mobility	*Karimi et al. 2019*
**Overall conclusion**	
Some evidence suggests that higher **BMI or obesity** is associated with an **increased risk for CRF** in survivors of childhood, adolescent and young adult cancers.	6 studies (4 samples)<br>**Level C**

Table S12 continued

1.14 What is the **risk of CRF** in CAYA cancer survivors who **smoke**?	
**Conclusion single studies**	
Multivariable logistic regression analysis* showed no significant association between smoking and CRF:<br>• Smoking OR=1.34 (95%CI=0.7-2.5), p=0.3<br>Childhood cancer survivors (n=232; HL, NHL, ALL; median age at diagnosis 9.6 years; median age at study 29.7 years; same sample as ***Hamre et al. 2013a***); *adjusted for age at study, sex, diagnosis, BMI, analgesics use, heart function, T-cell origin, CNS-irradiation, and B-symptoms at diagnosis	*Hamre et al. 2013b*
**Overall conclusion**	
Some evidence suggests that **smoking** is **not significantly associated** with the risk of CRF in survivors of childhood, adolescent, and young adult cancers.	1 study<br>**Level C**

1.15 What is the **risk of CRF** in CAYA cancer survivors **with sleep problems**?	
**Conclusion single studies**	
Multivariate logistic regression analysis* showed that having sleep problems was significantly associated with an increased risk of CRF:<br>• **Sleep problems: OR=6.15 (95%CI:2.33-16.22)**<br>Survivors of childhood leukemia (n=161; average age at diagnosis: 7.4 years; average time since end of treatment 13.9 years); *adjusted for marital status, having children, pain, obesity, neuro-cognitive impairment, exercise-induced symptoms, unemployment, and relapse	*Meeske et al. 2005*
Multiple logistic regression analysis* showed no significant association between insomnia and CRF:<br>• Insomnia present vs. insomnia absent: not significant (effect measure not reported)<br>Childhood cancer survivors (n=62; Lymphoma, ALL; mean age at study 34.05 years; median 25.3 years of follow-up; follow-up study with all 62 survivors also participating in the ***Hamre et al. 2013*a**); *adjusted for PHQ9 score, pain, number of steps, and depressive symptoms	*Zeller et al. 2014*
**Overall conclusion**	
Some evidence suggests that **sleep problems** are associated with an **increased risk for CRF** in survivors of childhood, adolescent and young adult cancers.	2 studies<br>**Level C**

1.16 What is the **risk of CRF** in CAYA cancer survivors **by quality of life (QoL)?**	
**Conclusion single studies**	
Multivariable regression analysis* showed that better health-related quality of life was significantly associated with a decreased risk of total fatigue:<br>• **HRQoL score: β= 0.87, p<0.001**<br>Survivors of extracranial childhood cancer (n=199; mean age at diagnosis: 3.6 years; mean age at study: 14.4 years). **Lower scores of the effect measure indicate more fatigue**. *adjusted for age at study, sex, diagnosis, treatment, follow-up time, additional diagnosis, remedial education, overall average grade, and happiness	*Mört et al. 2011*
**Overall conclusion**	
Some evidence suggests that better **health-related quality of life** is associated with a **decreased risk for CRF** in survivors of childhood, adolescent and young adult cancers.	1 study<br>**Level C**

1.17 What is the **risk of CRF** in CAYA cancer survivors **by happiness**?	
**Conclusion single studies**	
Multivariate regression analysis* showed no significant association of self-rated happiness and total fatigue.<br>• Self-rated happiness: No (Ref. Yes) β= -1.13, p>0.05<br>Survivors of extracranial childhood cancer (n=199; mean age at diagnosis: 3.6 years; mean age at study: 14.4 years). **Lower scores of the effect measure indicate more fatigue**. *adjusted for age at study, sex, diagnosis, treatment, follow-up time, additional diagnosis, remedial education, overall average grade, and HRQoL	*Mört et al. 2011*
**Overall conclusion**	
Some evidence suggests that self-rated **happiness** is **not significantly associated** with the risk of **CRF** in survivors of childhood, adolescent and young adult cancers.	1 study<br>**Level C**

Table S12 continued

1.18 What is the **risk of CRF** in CAYA cancer survivors **with late effects or health problems**?	
**Conclusion single studies**	
Multivariable regression analysis* showed that suffering from late effects/health problems was significantly associated with an increased risk of CRF:<br>• **Late effects/health problems: β= 0.14, $p<0.05$**<br>Childhood cancer survivors (n=416; mean age at diagnosis 8 years; mean age at study 24 years; Leukemia/Lymphoma>Solid tumor>brain/CNS tumor); *adjusted for sex, age at study, marital status, educational achievement, employment, age at diagnosis, diagnosis, treatment duration, follow-up time, treatment, and depression	*Langeveld et al. 2003*
Multivariable regression analysis* showed no significant association of an additional non-cancer diagnosis and total fatigue:<br>• Additional diagnosis: No (Ref. Yes) β=2.2, $p>0.05$<br>Survivors of extracranial childhood cancer (n=199; mean age at diagnosis: 3.6 years; mean age at study: 14.4 years). **Lower scores of the effect measure indicate more fatigue**. *adjusted for age at study, sex, diagnosis, treatment, follow-up time, remedial education, overall average grade, happiness, and HRQoL	*Mört et al. 2011*
Multivariable logistic regression* showed that 3 or more chronic conditions was significantly associated with an increased risk of CRF:<br>• Chronic conditions: 1-2 (Ref. 0) OR=1.23 (95%CI:0.55-2.74)<br>• **Chronic conditions: 3 or more (Ref. 0) OR=4.27 (95%CI:1.52-11.99)**<br>Childhood and adolescent cancer survivors (n=268; median age at diagnosis: 6.4 years; mean time since diagnosis 13.1 years; median age at study 21.4 years; Leukemia>HL>NL>Bone tumors>soft tissue sarcoma>neuroblastoma>wilms tumor>other); *adjusted for sex, age at study, income, and survival time	*Frederick et al. 2016*
Multivariable logistic regression* showed that impaired physical function was associated with an increased risk for CRF:<br>• **Physical functioning limitations (Ref. no limitations) OR=3.28 (95%CI:1.75-6.15, $p<0.001$)**<br>Hodgkin's lymphoma survivors of the childhood cancer survivor study (CCSS; n=751; 42.5% aged 11-15 years at diagnosis; at least 5 years since diagnosis); *adjusted for sex, emotional distress, employment, pain, physical function, and BMI	*Rach et al. 2017*
Hierarchical linear regression* showed that problems with physical and function mobility was associated with increased levels of CRF:<br>• **Physical and function mobility: β=-0.427, $p<0.001$**<br>Pediatric cancer survivors (n=144; mixed diagnoses; mean age at study 12.9 years, mean 5.9 years since diagnosis); *adjusted for age, sex, race, time since diagnosis, diagnosis, chemotherapy, radiation, depression, parent reported depression/anxiety, BMI, physical and function mobility	*Karimi et al. 2019*
**Overall conclusion**	
Evidence suggests that **late effects or health problems** are associated with an **increased risk for CRF** in survivors of childhood, adolescent and young adult cancers.	5 studies<br>**Level B**

1.19 What is the **risk of CRF** in CAYA cancer survivors **with neuro-cognitive impairment**?	
**Conclusion single studies**	
Multivariate logistic regression* showed that neuro-cognitive impairment was significantly associated with an increased risk of CRF:<br>• **Neuro-cognitive impairment: OR=2.56 (95%CI:1.02-6.38)**<br>Survivors of childhood leukemia (n=161; average age at diagnosis: 7.4 years; average time since end of treatment 13.9 years).; *adjusted for marital status, having children, sleep problems, pain, obesity, exercise-induced symptoms, unemployment, and relapse	*Meeske et al. 2005*
**Overall conclusion**	
Some evidence suggests that **neuro-cognitive impairment** is associated with an **increased risk for CRF** in survivors of childhood, adolescent and young adult cancers.	1 study<br>**Level C**

Table S12 continued

1.20 What is the **risk of CRF** in CAYA cancer survivors **with higher brain dysfunction**?	
**Conclusion single studies**	
Multivariable regression analysis* showed that higher brain dysfunction was associated with an increased risk of CRF.<br>• **Higher brain dysfunction: Impact= 15.2, p=0.004**<br>Childhood brain tumor survivors (n=104, mean age at diagnosis 13.3 years, mean age at study 26.8 years). A positive impact **indicates more fatigue; a negative impact less fatigue.** *adjusted for age, sex, age at diagnosis, hydrocephalus at diagnosis, tumor pathology, tumor location, neurosurgery, radiation treatment, chemotherapy, tumor recurrence and time since completion of antitumor therapy	*Sato et al. 2014*
**Overall conclusion**	
Some evidence suggests that **higher brain dysfunction** is associated with an **increased risk for CRF** in survivors of childhood, adolescent and young adult cancers.	1 study<br>**Level C**

1.21 What is the **risk of CRF** in CAYA cancer survivors **with seizures**?	
**Conclusion single studies**	
Multivariable regression analysis* showed no significant association between seizures and CRF:<br>• Seizure: Impact= -7.9, p=0.158<br>Childhood brain tumor survivors (n=104, mean age at diagnosis 13.3 years, mean age at study 26.8 years, brain tumors) . A positive impact **indicates more fatigue; a negative impact less fatigue.** *adjusted for age, sex, age at diagnosis, hydrocephalus at diagnosis, tumor pathology, tumor location, neurosurgery, radiation treatment, chemotherapy, tumor recurrence and time since completion of antitumor therapy	*Sato et al. 2014*
**Overall conclusion**	
Some evidence suggests that **seizures** are **not significantly associated** with the risk for **CRF** in survivors of childhood, adolescent and young adult cancers.	1 study<br>**Level C**

1.22 What is the **risk of CRF** in CAYA cancer survivors **with heart problems**?	
**Conclusion single studies**	
Multivariate logistic regression analysis* showed that congestive heart failure was significantly associated with an increased risk of CRF:<br>• **Congestive heart failure: Yes (Ref. No): OR=2.9 (95%CI:1.4-6.1)**<br>Childhood cancer survivors (CCSS; n=1897; mixed diagnoses; diagnosed before the age of 21 years; at least 5 years from diagnosis); *adjusted for sex, lung fibrosis, hypothyroidism, depression, BMI, marital status, employment status, and infant at home	*Mulrooney et al. 2008*
Multivariable logistic regression analysis* showed no significant association between reduced heart function and CRF:<br>• Reduced heart function OR=1.8 (95%CI:1.0-3.3), p=0.06<br>Childhood cancer survivors (n=232; HL, NHL, ALL; median age at diagnosis 9.6 years; median age at study 29.7 years; same sample as ***Hamre et al. 2013a***); *adjusted for age at study, sex, diagnosis, smoking, BMI, analgesics use, T-cell origin, CNS-irradiation, and B-symptoms at diagnosis	*Hamre et al. 2013b*
**Overall conclusion**	
Some evidence suggests that a **heart problem** is associated with an **increased risk for CRF** in survivors of childhood, adolescent and young adult cancers.	2 studies<br>**Level C**

1.23 What is the **risk of CRF** in CAYA cancer survivors **with exercise-induced symptoms**?	
**Conclusion single studies**	
Multivariate logistic regression* showed that exercise-induced symptoms are associated with an increased risk of CRF:<br>• **Exercise-induced symptoms: OR=2.98 (95%CI:1.11-8.02)**<br>Survivors of childhood leukemia (n=161; average age at diagnosis: 7.4 years; average time since end of treatment 13.9 years); *adjusted for marital status, having children, sleep problems, pain, obesity, neuro-cognitive impairment, unemployment, and relapse	*Meeske et al. 2005*
**Overall conclusion**	
Some evidence suggests that **exercise-induced symptoms** are associated with an **increased risk for CRF** in survivors of childhood, adolescent and young adult cancers.	1 study<br>**Level C**

Table S12 continued

1.24 What is the **risk of CRF** in CAYA cancer survivors **with motility disturbance of limbs**?	
**Conclusion single studies**	
Multivariable regression analysis* showed no significant association between motility disturbance of limbs and CRF:<br>• Motility disturbance of limbs: Impact= -5.5, p=0.308<br>Childhood brain tumor survivors (n=104, mean age at diagnosis 13.3 years, mean age at study 26.8 years) . A positive impact **indicates more fatigue; a negative impact less fatigue.** *adjusted for age, sex, age at diagnosis, hydrocephalus at diagnosis, tumor pathology, tumor location, neurosurgery, radiation treatment, chemotherapy, tumor recurrence and time since completion of antitumor therapy	*Sato et al. 2014*
**Overall conclusion**	
Some evidence suggests that **motility disturbance of limbs** is **not significantly associated** with the risk for **CRF** in survivors of childhood, adolescent and young adult cancers.	1 study<br>**Level C**

1.25 What is the **risk of CRF** in CAYA cancer survivors **with ocular/vision impairment**?	
**Conclusion single studies**	
Multivariable regression analysis* showed no significant association between ocular/vision impairment and CRF:<br>• Ocular/vision impairment: impact 5.9, p=0.315<br>Childhood brain tumor survivors (n=104, mean age at diagnosis 13.3 years, mean age at study 26.8 years, brain tumors). A positive impact **indicates that more fatigue; a negative impact less fatigue.** *adjusted for age, sex, age at diagnosis, hydrocephalus at diagnosis, tumor pathology, tumor location, neurosurgery, radiation treatment, chemotherapy, tumor recurrence and time since completion of antitumor therapy	*Sato et al. 2014*
**Overall conclusion**	
Some evidence suggests that **ocular/vision impairment** is **not significantly associated** with the risk for **CRF** in survivors of childhood, adolescent and young adult cancers.	1 study<br>**Level C**

1.26 What is the **risk of CRF** in CAYA cancer survivors **by thyroid status**?	
**Conclusion single studies**	
Multivariate logistic regression analysis* showed no significant association between hypothyroidism and CRF:<br>• Hypothyroidism: Yes (Ref. No): OR=0.9 (95%CI:0.7-1.3)<br>Childhood cancer survivors (CCSS; n=1897; mixed diagnoses; diagnosed before the age of 21 years; at least 5 years from diagnosis); *adjusted for sex, heart failure, lung fibrosis, depression, BMI, marital status, employment status, and infant at home	*Mulrooney et al. 2008*
Multivariable logistic regression analysis* showed no significant association between hypothyroidism and CRF:<br>• Present hypothyroidism (vs. Thyroid status normal): OR=1.4 (95%CI:0.7-3.0), p=0.4<br>Childhood cancer survivors (n=290; HL, NHL, ALL; median age at diagnosis 9.5 years; median age at study 29.6 years); *adjusted for diagnosis, age at survey, treatment era, sex, HADS (Hospital Anxiety and Depression scale) total score	*Hamre et al. 2013a*
**Overall conclusion**	
Evidence suggests that **thyroid status** is **not significantly associated** with the risk for **CRF** in survivors of childhood, adolescent and young adult cancers.	2 studies<br>**Level B**

1.27 What is the **risk of CRF** in CAYA cancer survivors **with endocrine abnormalities?**	
**Conclusion single studies**	
Multivariable regression analysis* showed no significant association between endocrine abnormalities and CRF:<br>• Endocrine abnormality: impact 12.9, p=0.20<br>Childhood brain tumor survivors (n=104, mean age at diagnosis 13.3 years, mean age at study 26.8 years). A positive impact **indicates that more fatigue; a negative impact less fatigue.** *adjusted for age, sex, age at diagnosis, hydrocephalus at diagnosis, tumor pathology, tumor location, neurosurgery, radiation treatment, chemotherapy, tumor recurrence and time since completion of antitumor therapy	*Sato et al. 2014*
**Overall conclusion**	
Some evidence suggests that **endocrine abnormality** is **not significantly associated** with the risk for **CRF** in survivors of childhood, adolescent and young adult cancers.	1 study<br>**Level C**

Table S12 continued

1.28 What is the **risk of CRF** in CAYA cancer survivors **with lung fibrosis**?	
**Conclusion single studies**	
Multivariate logistic regression analysis* showed that lung fibrosis was significantly associated with an increased risk of CRF:<br>• **Lung fibrosis: Yes (Ref. No): OR=2.9 (95%CI:1.5-5.4)**<br>Childhood cancer survivors (CCSS; n=1897; mixed diagnoses; diagnosed before the age of 21 years; at least 5 years from diagnosis); *adjusted for sex, heart failure, hypothyroidism, depression, BMI, marital status, employment status, and infant at home	*Mulrooney et al. 2008*
**Overall conclusion**	
Some evidence suggests that **lung fibrosis** is associated with an **increased risk for CRF** in survivors of childhood, adolescent and young adult cancers.	1 study<br>**Level C**

1.29 What is the **risk of CRF** in CAYA cancer survivors **with pain**?	
**Conclusion single studies**	
Multivariate logistic regression analysis* showed that pain was significantly associated with an increased risk of CRF:<br>• **Pain: OR=5.56 (95%CI:2.13-14.48)**<br>Survivors of childhood leukemia (n=161; average age at diagnosis: 7.4 years; average time since end of treatment 13.9 years); *adjusted for marital status, having children, sleep problems, obesity, neuro-cognitive impairment, exercise-induced symptoms, unemployment, and relapse	*Meeske et al. 2005*
Multiple logistic regression analysis* showed no significant association between the pain severity score and CRF:<br>• Pain severity score: not significant (effect measure not reported)<br>Childhood cancer survivors (n=62; Lymphoma, ALL; mean age at study 34.05 years; median 25.3 years of follow-up; follow-up study with all 62 survivors also participating in the ***Hamre et al. 2013*a**); *adjusted for insomnia, PHQ9 score, number of steps, and depressive symptoms	*Zeller et al. 2014*
Multivariable logistic regression analysis* showed no significant association between regular use of analgesics and CRF:<br>• Regular use of analgesics OR=1.6 (95%CI:0.7-3.7), p=0.2<br>Childhood cancer survivors (n=232; HL, NHL, ALL; median age at diagnosis 9.6 years; median age at study 29.7 years; same sample as ***Hamre et al. 2013a***); *adjusted for age at study, sex, diagnosis, smoking, BMI, heart function, T-cell origin, CNS-irradiation, and B-symptoms at diagnosis	*Hamre et al. 2013b*
Multivariable logistic regression* showed that body pain was associated with an increased risk for CRF:<br>• **Elevated body pain (Ref. subclinical pain) OR=3.73 (95%CI:2.09-6.67, p<0.001)**<br>Hodgkin’s lymphoma survivors of the childhood cancer survivor study (CCSS; n=751; 42.5% aged 11-15 years at diagnosis; at least 5 years since diagnosis); *adjusted for sex, emotional distress, employment, pain, physical function, and BMI	*Rach et al. 2017*
**Overall conclusion**	
Some evidence suggests that **pain** is associated with an **increased risk for CRF** in survivors of childhood, adolescent and young adult cancers.	4 studies (3 samples)<br>**Level B**

Table S12 continued

1.30 What is the **risk of CRF** in CAYA cancer survivors **by cytokine levels**?	
**Conclusion single studies**	
Multivariable logistic regression analysis* showed no significant association between cytokine levels and CRF (OR, 95%CI, p-value):<br>IL-1ra OR=0.9 (95%CI:0.6-1.3, p=0.5)<br>IL-6 OR=1.0 (0.5-2.4, p=0.9)<br>IL-7 OR=2.1 (0.02-224, p=0.7)<br>IL-8/CXCL8 OR=32.2 (0.2-5346, p=0.2)<br>IL-9 OR=1.0 (0.8-1.2, p=0.9)<br>IL-10 OR=0.5 (0.06-3.3, p=0.4)<br>IL-12 OR=0.7 (0.2-2.0, p=0.5)<br>FGF OR=5.2 (0.6-43.6, p=0.1)<br>Eotaxin/CCL11 OR=1.0 (0.9-1.1, p=0.5)<br>IP-10/CXCL10 OR=1.0 (0.9-1.1, p=0.3)<br>MCP-1/CCL2 OR=1.7 (0.3-8.5, p=0.5)<br>MIP-1 β/CCL4 OR=1.8 (0.8-4.1, p=0.2)<br>RANTES/CCL5 OR=1.0 (1.0-1.0, p=0.3)<br>PDGF OR=1.0 (1.0-1.0, p=0.3)<br>VEGF OR=0.8 (0.5-1.3, p=0.4)<br>IFN-γ OR=0.7 (0.4-1.3, p=0.3)<br>Childhood cancer survivors (n=232; HL, NHL, ALL; median age at diagnosis 9.6 years; median age at study 29.7 years; same sample as ***Hamre et al. 2013a***); *adjusted for diagnosis, age, sex, BMI, and reduced heart function	*Hamre et al. 2013b*
**Overall conclusion**	
Some evidence suggests that **cytokine levels** are **not significantly associated** with the risk for **CRF** in survivors of childhood, adolescent and young adult cancers.	1 study<br>**Level C**

1.31 What is the **risk of CRF** in CAYA cancer survivors **with psychological distress**?	
**Conclusion single studies**	
Multivariable regression analysis* showed that depression was significantly associated with an increased risk of CRF:<br>• **Depression: β=0.54, p<0.001**<br>Childhood cancer survivors (n=416; mean age at diagnosis 8 years; mean age at study 24 years; Leukemia/Lymphoma>Solid tumor>brain/CNS tumor); *adjusted for sex, age at study, marital status, educational achievement, employment, age at diagnosis, diagnosis, treatment duration, follow-up time, late effects, and treatment	*Langeveld et al. 2003*
Multivariate logistic regression analysis* showed that depression was significantly associated with an increased risk of CRF:<br>• **Depressed: Yes (Ref. No): OR=7.5 (95%CI:5.1-10.9)**<br>Childhood cancer survivors (CCSS; n=1897; mixed diagnoses; diagnosed before the age of 21 years; at least 5 years from diagnosis); *adjusted for sex, heart failure, lung fibrosis, hypothyroidism, BMI, marital status, employment status, and infant at home	*Mulrooney et al. 2008*
Multivariable logistic regression analysis* showed that depression was significantly associated with an increased risk of CRF:<br>• **HADS (Hospital Anxiety and Depression Scale) total score: OR=1.15 (95%CI:1.1-1.2), p<0.001**<br>Childhood cancer survivors (n=290; HL, NHL, ALL; median age at diagnosis 9.5 years; median age at study 29.6 years); *adjusted for diagnosis, age at survey, treatment era, sex, and thyroid status	*Hamre et al. 2013a*
Multiple logistic regression analysis* showed that depression (measured by PHQ8) was significantly associated with an increased risk of CRF:<br>• **Level of depressive symptoms (PHQ8 score): OR 1.3 (95%CI:1.1-1.7), p=0.014**<br>• PHQ9 score (patient health questionnaire-9, assesses degree of depression): not significant (effect measure not reported)<br>Childhood cancer survivors (n=62; Lymphoma, ALL; mean age at study 34.05 years; median 25.3 years of follow-up; follow-up study with all 62 survivors also participating in the ***Hamre et al. 2013a***); *adjusted for insomnia, pain, and number of steps	*Zeller et al. 2014*
Multivariable logistic regression* showed that emotional distress was associated with an increased risk for CRF:<br>• **Emotional distress (Ref. no emotional distress) OR=8.38 (95%CI:4.28-16.42, p<0.001)**<br>Hodgkin's lymphoma survivors of the childhood cancer survivor study (CCSS; n=751; 42.5% aged 11-15 years at diagnosis; at least 5 years since diagnosis); *adjusted for sex, emotional distress, employment, pain, physical function, and BMI	*Rach et al. 2017*
Hierarchical linear regression* showed that self-reported depression symptoms were associated with increased levels of CRF:<br>• **Depression: β=0.396, p<0.001**<br>• Parent-reported depression/anxiety: β=0.117, p=0.095<br>Pediatric cancer survivors (n=144; mixed diagnoses; mean age at study 12.9 years, mean 5.9 years since diagnosis); *adjusted for age, sex, race, time since diagnosis, diagnosis, chemotherapy, radiation, depression, parent reported depression/anxiety, BMI, physical and function mobility	*Karimi et al. 2019*
**Overall conclusion**	
There is evidence that **psychological distress** is associated with an **increased risk for CRF** in survivors of childhood, adolescent and young adult cancers.	6 studies (5 samples)<br>**Level A**

Table S12 continued

1.32 What is the **risk of CRF** in CAYA cancer survivors **by primary cancer diagnosis**?	
**Conclusion single studies**	
Multivariable regression analysis* showed no significant association between primary cancer diagnosis (solid tumor vs. leukemia/NHL, brain tumor vs. leukemia/NHL) and CRF:<br>• Solid tumor vs Leukaemia/NHL without CRT: β=0.02, p>0.05<br>• Brain/CNS tumor vs Leukaemia/NHL without CRT: β= -0.08, p>0.05<br>Childhood cancer survivors (n=416; mean age at diagnosis 8 years; mean age at study 24 years; Leukemia/Lymphoma>Solid tumor>brain/CNS tumor); *adjusted for sex, age at study, marital status, educational achievement, employment, age at diagnosis, treatment duration, follow-up time, late effects, treatment, and depression	*Langeveld et al. 2003*
Multivariate logistic regression analysis* showed no significant association between primary cancer diagnosis (CNS malignancy, Hodgkin disease, soft tissue sarcoma or bone cancer (all vs. ALL)) and CRF:<br>• Diagnosis: CNS malignancy (Ref. ALL): OR=1.3 (95%CI:0.8-2.1)<br>• Diagnosis: Hodgkin disease (Ref. ALL): OR=1.2 (95%CI:0.7-1.8)<br>• Diagnosis: Soft tissue sarcoma (Ref. ALL): OR=1.0 (95%CI:0.6-1.7)<br>• Diagnosis: Bone cancer (Ref. ALL): OR=1.3 (95%CI: 0.7-2.3)<br>Childhood cancer survivors (CCSS; n=1897; mixed diagnoses; diagnosed before the age of 21 years; at least 5 years from diagnosis); *adjusted for age at diagnosis, radiation, and chemotherapy	*Mulrooney et al. 2008*
Multivariate regression analysis* showed no significant association between sarcoma survivors (vs. leukemia) and CRF:<br>• **Diagnosis: NHL (Ref. leukemia) β= -2.49, p>0.05**<br>• **Diagnosis: Sarcoma (Ref. leukemia) β= -13.28, p<0.01**<br>• **Diagnosis: NBL (Ref. leukemia) β= -2.3, p>0.05**<br>• **Diagnosis: Other (Ref. Leukemia) β= -0.85, p>0.05**<br>Survivors of extracranial childhood cancer (n=199; mean age at diagnosis: 3.6 years; mean age at study: 14.4 years). **Lower scores of the effect measure indicate more fatigue**. *adjusted for age at study, sex, treatment, follow-up time, additional diagnosis, remedial education, overall average grade, happiness, and HRQoL	*Mört et al. 2011*
Multiple regression analysis* showed no significant association between primary cancer diagnosis and CRF.<br>• AML (Ref. ALL): β= -0.02, p>0.05<br>Childhood cancer survivors (n=81, diagnoses: ALL and AML, age at diagnosis: mean 6.7 years; age at study: mean 14.1 years). *adjusted for age at study, sex, cranial irradiation, TBI, and follow-up time	*Nagai et al. 2012*
Multivariable logistic regression analysis* showed no significant association between primary cancer diagnosis (lymphoma vs. leukemia) and CRF:<br>• NHL (vs. ALL): OR=1.5 (95%CI:0.6-3.4), p=0.4<br>• HL (vs ALL): OR=1.7 (95%CI:0.8-3.5), p=0.2<br>Childhood cancer survivors (n=290; HL, NHL, ALL; median age at diagnosis 9.5 years; median age at study 29.6 years); *adjusted for age at survey, treatment era, sex, thyroid status, and HADS (Hospital Anxiety and Depression scale) total score	*Hamre et al. 2013a*
Multivariable logistic regression analysis* showed no significant association between diagnosis and CRF, but T-cell origin was significantly associated with an increased risk for CRF:<br>• Diagnosis: NHL (Ref. ALL): OR=1.3 (95% CI: 0.6–2.8), p=0.6<br>• Diagnosis: HL (Ref. ALL) OR=1.8 (95% CI: 0.9–3.3), p=0.08<br>• **T-cell origin: Yes (Ref. No): OR=10.3 (95% CI: 2.7–39.3), p=0.01**<br>• T-cell origin: Unknown (Ref. No): OR=1.7 (95%CI:0.7-3.9), p=0.2<br>• B-symptoms at diagnosis: Yes (Ref. No): OR=2.5 (95% CI: 1.0–6.2), p=0.05<br>• B-symptoms at diagnosis: Unknown (Ref. No): OR=1.1 (95% CI:0.4–3.1), p=0.9<br>Childhood cancer survivors (n=232; HL, NHL, ALL; median age at diagnosis 9.6 years; median age at study 29.7 years; same sample as ***Hamre et al. 2013a***); *adjusted for age at survey, sex, smoking, BMI, analgesics use, heart function, and CNS-irradiation	*Hamre et al. 2013b*
Univariable logistic regression analysis showed no significant association between primary cancer diagnosis and CRF (variable was therefore not included in the multivariable model):<br>• Diagnosis: not significant<br>Childhood and adolescent cancer survivors (n=268; median age at diagnosis: 6.4 years; mean time since diagnosis 13.1 years; median age at study 21.4 years; Leukemia>HL>NL>Bone tumors>soft tissue sarcoma>neuroblastoma>WT>other).	*Frederick et al. 2016*
Hierarchical linear regression* showed no significant association between diagnosis and CRF:<br>• Diagnosis: β=-0.045, p=0.464<br>Pediatric cancer survivors (n=144; mixed diagnoses; mean age at study 12.9 years, mean 5.9 years since diagnosis); *adjusted for age, sex, race, time since diagnosis, diagnosis, chemotherapy, radiation, depression, parent reported depression/anxiety, BMI, physical and function mobility	*Karimi et al. 2019*
**Overall conclusion**	
Evidence suggests that primary cancer **diagnosis** is **not significantly associated** with the risk for CRF in survivors of childhood, adolescent and young adult cancer survivors.	8 studies (7 samples)<br>**Level B**

Table S12 continued

1.33 What is the **risk of CRF** in CAYA cancer survivors **with a relapse**?	
**Conclusion single studies**	
Multivariable logistic regression analysis* showed that relapse was significantly associated with an increased risk of CRF:<br>• **Relapse p<0.05 (effect measure not reported)**<br>Survivors of childhood leukemia (n=161; average age at diagnosis: 7.4 years; average time since end of treatment 13.9 years); *adjusted for marital status, having children, sleep problems, pain, obesity, neuro-cognitive impairment, exercise-induced symptoms, and unemployment	*Meeske et al. 2005*
Multivariable logistic regression analysis* showed that history of leukemia relapse was significantly associated with an increased risk of CRF:<br>• **History of leukemia relapse (vs. none): OR=8.35 (95%CI:1.16-59.93), p<0.03**<br>Childhood acute lymphoblastic leukemia survivors (n=162; median age at diagnosis: 3.9 years; median time from diagnosis: 10.2 years); *unclear what other variables were included in the model	*Khan et al. 2014*
Univariable logistic regression analysis showed no significant association between recurrence and CRF (variable was therefore not included in the multivariable model):<br>• Recurrence: not significant<br>Childhood and adolescent cancer survivors (n=268; median age at diagnosis: 6.4 years; mean time since diagnosis 13.1 years; median age at study 21.4 years; Leukemia>HL>NL>Bone tumors>soft tissue sarcoma>neuroblastoma>Wilms tumor>other).	*Frederick et al. 2016*
**Overall conclusion**	
Evidence suggests that a **relapse** is associated with an **increased risk for CRF** in survivors of childhood, adolescent and young adult cancers.	3 studies<br>**Level B**

1.34 What is the **risk of CRF** in CAYA cancer survivors who were treated with **CNS/brain irradiation**?	
**Conclusion single studies**	
Multivariable regression analysis* showed that treatment with CRT (leukemia/NHL) was significantly associated with a decreased risk of CRF (vs. without CRT):<br>• **Leukemia/Non-hodgkin lymphoma with CRT vs without CRT: β= -0.16, p<0.05**<br>Childhood cancer survivors (n=416; mean age at diagnosis 8 years; mean age at study 24 years; Leukemia/Lymphoma>Solid tumor>brain/CNS tumor); *adjusted for sex, age at study, marital status, educational achievement, employment, age at diagnosis, diagnosis, treatment duration, follow-up time, late effects, and depression	*Langeveld et al. 2003*
Multivariable logistic regression analysis* showed that radiotherapy including craniospinal radiation (vs. none) was significantly associated with an increased risk of CRF:<br>• **Radiotherapy to head and/or neck vs. none: RR=1.76 (95%CI:1.14-2.71)**<br>• **Radiotherapy to head and/or neck and thorax and/or abdomen including craniospinal vs. none: RR=2.43 (95% CI 1.54-3.82)**<br>Childhood cancer survivors (n=1284; Leukemia>Lymphoma>Kidney/Wilms tumor>Soft tissue sarcoma; median follow-up time: 17 years; median age of 24.4 years); *adjusted for sex, TBI, chemotherapy, surgery, follow-up duration, and age at diagnosis	*Geenen et al. 2007*
Multiple regression analysis* showed no significant association between cranial irradiation and total fatigue:<br>• Cranial irradiation: β= -0.04, p>0.05<br>Childhood cancer survivors (n=81, diagnoses: ALL and AML, age at diagnosis: mean 6.7 years; age at study: mean 14.1 years); *adjusted for age at study, sex, diagnosis, TBI, and follow-up time	*Nagai et al. 2012*
Univariable logistic regression analysis showed no significant association between CNS directed radiation therapy and CRF (variable was therefore not included in the multivariable model):<br>• CNS directed radiation therapy: not significant<br>Childhood and adolescent cancer survivors (n=268; median age at diagnosis: 6.4 years; mean time since diagnosis 13.1 years; median age at study 21.4 years; Leukemia>HL>NL>Bone tumors>soft tissue sarcoma>neuroblastoma>wilms tumor>other).	*Frederick et al. 2016*
Multivariable logistic regression analysis* showed no significant association between CNS-irradiation and CRF:<br>• **CNS-irradiation OR=0.9 (95%CI:0.3-2.9), p=0.9**<br>Childhood cancer survivors (n=232; HL, NHL, ALL; median age at diagnosis 9.6 years; median age at study 29.7 years; same sample as ***Hamre et al. 2013a***); *adjusted for age at survey, sex, diagnosis, smoking, BMI, analgesics use, heart function, T-cell origin, and B-symptoms at diagnosis	*Hamre et al. 2013b*
**Overall conclusion**	
There is conflicting evidence on the association of **CNS/brain irradiation** and the risk for CRF in survivors of childhood, adolescent and young adult cancers.	5 studies<br>**Conflicting evidence**

Table S12 continued

1.35 What is the **risk of CRF** in CAYA cancer survivors who were treated with **total body irradiation (TBI)**?	
**Conclusion single studies**	
Multivariable logistic regression analysis* showed no significant association between TBI and CRF:<br>• **TBI vs. none: RR 1.67 (95% CI 0.62-4.47)**<br>Childhood cancer survivors (n=1284; Leukemia>Lymphoma>Kidney/Wilms tumor>Soft tissue sarcoma; median follow-up time: 17 years; median age of 24.4 years); adjusted for sex, radiation, chemotherapy, surgery, follow-up duration, and age at diagnosis	*Geenen et al. 2007*
Multiple regression analysis* showed no significant association between TBI and:<br>• Total body irradiation: β=2.72, p>0.05<br>Survivors (n=81; ALL and AML; age at diagnosis: mean 6.7 years; age at study: mean 14.1 years); *adjusted for age at study, sex, diagnosis, cranial irradiation, and follow-up time	*Nagai et al. 2012*
**Overall conclusion**	
Evidence suggests that **total body irradiation** is **not significantly associated** with the risk for **CRF** in survivors of childhood, adolescent and young adult cancer survivors.	2 studies<br>**Level B**

1.36 What is the **risk of CRF** in CAYA cancer survivors who were treated with **radiation not further specified?**	
**Conclusion single studies**	
Multivariable regression analysis* showed no significant association between radiotherapy (compared to chemotherapy) and CRF:<br>• Radiation therapy** vs chemotherapy**: β=0.01, not significant<br>• Combination therapy** vs chemotherapy**: β=0.04, not significant<br>Childhood cancer survivors (n=416; mean age at diagnosis 8 years; mean age at study 24 years; Leukemia/Lymphoma>Solid tumor>brain/CNS tumor); *adjusted for sex, age at study, marital status, educational achievement, employment, age at diagnosis, diagnosis, treatment duration, follow-up time, late effects, and depression; ** with or without surgery	*Langeveld et al. 2003*
Multivariate logistic regression analysis* showed that radiotherapy was significantly associated with an increased risk of CRF:<br>• **Radiation: Yes (Ref. No): OR=1.7 (95%CI:1.3-2.3)**<br>Childhood cancer survivors (CCSS; n=1897; mixed diagnoses; diagnosed before the age of 21 years; at least 5 years from diagnosis); *adjusted for diagnosis, age at diagnosis, and chemotherapy	*Mulrooney et al. 2008*
Multivariable regression analysis* showed no significant association between radiotherapy (compared to surgery alone) and CRF:<br>• Radiation (Ref. surgery alone): β= -8.73, p>0.05<br>Survivors of extracranial childhood cancer (n=199; mean age at diagnosis: 3.6 years; mean age at study: 14.4 years). **Lower scores of the effect measure indicate more fatigue**. *adjusted for age at study, sex, diagnosis, follow-up time, additional diagnosis, remedial education, overall average grade, happiness, and HRQoL	*Mört et al. 2011*
Univariable logistic regression analysis showed no significant association between any radiation therapy and CRF (variable was therefore not included in the multivariable model):<br>• Any radiation therapy: not significant<br>Childhood and adolescent cancer survivors (n=268; median age at diagnosis: 6.4 years; mean time since diagnosis 13.1 years; median age at study 21.4 years; Leukemia>HL>NL>Bone tumors>soft tissue sarcoma>neuroblastoma>wilms tumor>other).	*Frederick et al. 2016*
Hierarchical linear regression* showed no significant association between radiation and CRF:<br>• Radiation: β=-0.030, p=0.625<br>Pediatric cancer survivors (n=144; mixed diagnoses; mean age at study 12.9 years, mean 5.9 years since diagnosis); *adjusted for age, sex, race, time since diagnosis, diagnosis, chemotherapy, radiation, depression, parent reported depression/anxiety, BMI, physical and function mobility	*Karimi et al. 2019*
**Overall conclusion**	
Some evidence suggests that **treatment with radiation** is associated with an **increased risk for CRF** in survivors of childhood, adolescent and young adult cancers.	5 studies<br>**Level C**

Table S12 continued

1.37 What is the **risk of CRF** in CAYA cancer survivors who were treated with **chemotherapy**?	
**Conclusion single studies**	
Multivariable logistic regression analysis* showed no significant association between chemotherapy and CRF.<br>• **Anthracyclines (vs. None): RR=1.84 (95%CI:0.99-3.42)**<br>• Alkylating agents (vs. none): RR=1.40 (95%CI:0.81-2.42)<br>• Anthracyclines and alkylating agents (vs. none): RR=1.33 (95%CI:0.75-2.37)<br>• Other chemotherapy only (vs. none): RR=1.31 (95%CI:0.74-2.30)<br>Childhood cancer survivors (n=1284; Leukemia>Lymphoma>Kidney/Wilms tumor>Soft tissue sarcoma; median follow-up time: 17 years; median age of 24.4 years); *adjusted for sex, radiation, TBI, surgery, follow-up duration, and age at diagnosis	*Geenen et al. 2007*
Multivariate logistic regression analysis* showed no significant association between chemotherapy and CRF:<br>• Chemotherapy: Yes (Ref. No): OR=1.0 (95%CI:0.8-1.4)<br>Survivors (CCSS; n=1897; mixed diagnoses; diagnosed before the age of 21 years; at least 5 years from diagnosis); *adjusted for diagnosis, age at diagnosis, and radiation	*Mulrooney et al. 2008*
Multivariate regression analysis* showed no significant association between chemotherapy (vs. surgery only) and CRF:<br>• Treatment: Chemotherapy (Ref. surgery alone) β= -4.2, p>0.05<br>Survivors of extracranial childhood cancer (n=199; mean age at diagnosis: 3.6 years; mean age at study: 14.4 years). **Lower scores of the effect measure indicate more fatigue**. *adjusted for age at study, sex, diagnosis, follow-up time, additional diagnosis, remedial education, overall average grade, happiness, and HRQoL	*Mört et al. 2011*
Univariable logistic regression analysis showed no significant association between chemotherapy and CRF (variable was therefore not included in the multivariable model):<br>• Chemotherapy: not significant<br>• Doxorubicin: not significant<br>Childhood and adolescent cancer survivors (n=268; median age at diagnosis: 6.4 years; mean time since diagnosis 13.1 years; median age at study 21.4 years; Leukemia>HL>NL>Bone tumors>soft tissue sarcoma>neuroblastoma>wilms tumor>other).	*Frederick et al. 2016*
Hierarchical linear regression* showed no significant association between chemotherapy and CRF:<br>• Chemotherapy: β=0.097, p=0.121<br>Pediatric cancer survivors (n=144; mixed diagnoses; mean age at study 12.9 years, mean 5.9 years since diagnosis); *adjusted for age, sex, race, time since diagnosis, diagnosis, chemotherapy, radiation, depression, parent reported depression/anxiety, BMI, physical and function mobility	*Karimi et al. 2019*
**Overall conclusion**	
Evidence suggests that **chemotherapy** is **not significantly associated** with the risk for **CRF** in survivors of childhood, adolescent and young adult cancer survivors.	5 studies<br>**Level B**

1.38 What is the **risk of CRF** in CAYA cancer survivors who were treated with **surgery**?	
**Conclusion single studies**	
Multivariable logistic regression analysis* showed no significant association between surgery and CRF:<br>• Surgery yes vs. no: RR=1.09 (95%CI:0.76-1.58)<br>Childhood cancer survivors (n=1284; Leukemia>Lymphoma>Kidney/Wilms tumor>Soft tissue sarcoma; median follow-up time: 17 years; median age of 24.4 years); *adjusted for sex, radiation, TBI, chemotherapy, follow-up duration, and age at diagnosis	*Geenen et al. 2007*
Univariable logistic regression analysis showed no significant association between surgery and CRF (variable was therefore not included in the multivariable model):<br>• Surgery: not significant<br>Childhood and adolescent cancer survivors (n=268; median age at diagnosis: 6.4 years; mean time since diagnosis 13.1 years; median age at study 21.4 years; Leukemia>HL>NL>Bone tumors>soft tissue sarcoma>neuroblastoma>wilms tumor>other).	*Frederick et al. 2016*
**Overall conclusion**	
Evidence suggests that **surgery** is **not significantly associated** with the risk for **CRF** in survivors of childhood, adolescent and young adult cancer survivors.	2 studies<br>**Level B**

Table S12 continued

1.39 What is the **risk of CRF** in CAYA cancer survivors who were treated with **bone marrow / stem cell transplantation**?	
**Conclusion single studies**	
Multivariable regression analysis* showed no significant association between stem cell transplant (vs. surgery only) and CRF:<br>• SCT (Ref. surgery alone): β= -3.17, p>0.05<br>Survivors of extracranial childhood cancer (n=199; mean age at diagnosis: 3.6 years; mean age at study: 14.4 years). **Lower scores of the effect measure indicate more fatigue**. *adjusted for age at study, sex, diagnosis, follow-up time, additional diagnosis, remedial education, overall average grade, happiness, and HRQoL	*Mört et al. 2011*
Univariable logistic regression analysis showed no significant association between bone marrow transplant and CRF (variable was therefore not included in the multivariable model):<br>• Bone marrow transplant: not significant<br>Childhood and adolescent cancer survivors (n=268; median age at diagnosis: 6.4 years; mean time since diagnosis 13.1 years; median age at study 21.4 years; Leukemia>HL>NL>Bone tumors>soft tissue sarcoma>neuroblastoma>WT>other).	*Frederick et al. 2016*
**Overall conclusion**	
Evidence suggests that **stem cell transplantation** is **not significantly associated** with the risk for **CRF** in survivors of childhood, adolescent and young adult cancer survivors.	2 studies<br>**Level B**

1.40 What is the **risk of CRF** in CAYA cancer survivors **by treatment duration**?	
**Conclusion single studies**	
Multivariable regression analysis* showed no significant association between the duration of treatment and CRF:<br>• Duration of treatment: β=0.02, NS<br>Survivors of childhood cancer (n=416; mean age at diagnosis 8 years; mean age at study 24 years; Leukemia/Lymphoma>Solid tumor>brain/CNS tumor); *adjusted for sex, age at study, marital status, educational achievement, employment, age at diagnosis, diagnosis, follow-up time, late effects, treatment, and depression	*Langeveld et al. 2003*
**Overall conclusion**	
Some evidence suggests that **duration of treatment** is **not significantly associated** with the risk for **CRF** in survivors of childhood, adolescent and young adult cancer survivors.	1 study<br>**Level C**

1.41 What is the **risk of CRF** in CAYA cancer survivors **by treatment era**?	
**Conclusion single studies**	
Multivariable logistic regression analysis* showed no significant association between treatment era and CRF:<br>• Treatment 1970-1985 (vs. Treatment after 1985): OR=0.8 (95%CI:0.3-2.1), p=0.7<br>Childhood cancer survivors (n=290; HL, NHL, ALL; median age at diagnosis 9.5 years; median age at study 29.6 years); *adjusted for diagnosis, age at survey, sex, thyroid status, HADS (Hospital Anxiety and Depression scale) total score	*Hamre et al. 2013a*
**Overall conclusion**	
Some evidence suggests that **treatment era** is **not significantly associated** with the risk for **CRF** in survivors of childhood, adolescent and young adult cancers.	1 study<br>**Level C**

2. What is the **risk** for suffering from Fatigue in CAYA cancer survivors who had received **pulmonary radiation vs. no pulmonary radiation**?	
**Conclusion single studies**	
No studies identified in survivors of childhood, adolescent and young adult cancers.	
**Overall conclusion**	
**Risk after pulmonary radiation**<br>No studies reported on risk of CRF after pulmonary radiation in survivors of childhood, adolescent and young adult cancers.	0 studies<br>**No studies**

3. What is the **latency time** to develop Fatigue in CAYA cancer survivors?	
**Conclusion single studies**	
No studies identified in survivors of childhood, adolescent and young adult cancers.	
**Overall conclusion**	
**Latency time to develop CRF**<br>No studies reported on latency time to develop CRF in survivors of childhood, adolescent and young adult cancers.	0 studies<br>**No studies**

Table S12 continued

4. Does the **risk** of developing Fatigue **change over time** in CAYA cancer survivors?	
**Conclusion single studies**	
In a cohort of Hodgkin Lymphoma survivors (CCSS; n=103), they found no significant changes in mean levels of fatigue from end of treatment until 36 months post-therapy.	*Macpherson et al. 2015*
In longitudinally followed survivors of childhood lymphoma and leukemia (n=102), 60.4% of former fatigue cases were persistently fatigued, 81.6% of former non-fatigue cases were persistently non-fatigued, 39.6% of former fatigue cases were no longer fatigued, 18.4% of former non-fatigue cases were fatigued a median of 2.7 years later (range 1-4.3 years).	*Zeller et al. 2014*
**Overall conclusion**	
**Change of risk over time**<br>Evidence from longitudinal studies suggests that the risk of CRF does not change over time in the majority of CAYA cancer survivors. However, there is also a suggestion that the risk of CRF may increase or decrease over time. None of the studies reported the predictors for change, only risk factors for persistent CRF or persistent non-CRF were analyzed.	2 studies<br>**Level B**

5. Which fatigue scales are **reliable and valid diagnostic tools to diagnose CRF** in CAYA cancer survivors?	
**Conclusion single studies**	
**Systematic review**<br>Includes 25 articles that were published until April 2011	
In a systematic review of children and adolescents with cancer, the **Fatigue Scale-Child** (FS-C; 7-12 years) and **Fatigue Scale-Adolescent** (FS-A; 13-18 years) and its proxy versions (Fatigue Scale-Parents, Fatigue Scale-Staff), as well as the **PedsQL Multidimensional Fatigue Scale** (MFS; versions 5-7 years, 8-12 years, 13-18 years) self-report and parent proxy versions (additional version 2-4 years) have **good internal consistency and inter-rater reliability**, but known group validity is more variable. The authors recommend use of any of the two instruments for clinical trials in a CAYA cancer population.	*Tomlinson et al. 2013*
**Fatigue Scale-Child, Fatigue Scale-Adolescent and proxy versions (FS-C, FS-A)**	
In childhood cancer patients (CP; n=50) and survivors (CS; n=200), the **Chinese version of the Fatigue Scale for Children (FS-C)** was **reliable** (Cronbach's alpha = 0.91) and **valid:** semantic equivalence 83-100%. Content validity index 0.83 for scale. Known-group validity was good: CS scored significantly lower than CP, but statistically higher than HC. Discriminant validity was supported: strong correlation with CES-DC (r=0.53, p<0.01) and strong negative correlation with PedsQL (r=-0.54, p<0.01).	*Ho et al. 2016*
In adolescent cancer patients (ACP; n=50) and adolescent survivors (ACS; n=200), the **Chinese version of the Fatigue Scale for adolescents (FS-A)** was **reliable** (Cronbach's alpha = 0.89) and **valid**: Semantic equivalence was high: 94%. Content validity index was good: 0.92. Known groups validity was supported (ACS scored significantly lower than ACP, but higher than healthy controls). Discriminant validity was also supported: strong positive correlation with CES-DC (r=0.53, p<0.01) and strong negative correlation with PedsQL (r=-0.58, p<0.01).	*Ho et al. 2015*
In childhood cancer patients (n=52, n=86 parents and n=43 nurses), the **Turkish versions of the Child, Parent and Staff Fatigue Scale-24 Hours** was **reliable** (Cronbach's alpha: 0.83 (FS-C), 0.77 (FS-P), 0.72 (FS-S)) and **valid:** Language validity was confirmed by blind back-translation. Content validity was tested by ten academics working in the field of pediatrics and oncology and the versions adapted accordingly.	*Gerceker et al. 2012*
In adolescent cancer patients (n=138), the **Fatigue Scale-Adolescent** (13-18 years old) had acceptable psychometric properties and was able to reliably identify adolescent oncology patients with high fatigue (Cronbach's alpha was 0.87). Construct validity was acceptable: It was assessed with a confirmatory factor analysis and suggested a reasonable fit of the 4-factor structure (goodness-of-fit index was 0.855). Concurrent validity was acceptable: It was assessed with the Spearman correlation coefficient between FS-A and FS-P (0.347, p=0.0033). Cut score of 31 was used to identify fatigue: **sensitivity was 66.6%** and **specificity 82.6%**.	*Mandrell et al. 2011*
In adolescent cancer patients (n=64), the **Fatigue Scale-Adolescent** and its proxy versions (parents FS-P, and staff FS-S) had moderate to high internal consistency (Cronbach's alpha 0.81 (FS-A), 0.75 (FS-P), 0.85 (FS-S), was able to distinguish between known groups, and was able to measure change over time.	*Hinds et al. 2007*

Table S12 continued

**PedsQL Multidimensional Fatigue Scale (PedsQL MFS)**<br>Studies published after April 2011	
In childhood cancer patients (n=70), the **Arabic version of the PedsQL MFS** demonstrated good to excellent reliability (Cronbach's alpha between 0.87 and 0.94) for all scales except sleep rest subscale (α=0.67)). Validity was assessed by testing correlations of PedsQL MFS subscales to PedsQL TM 4.0 Generic Core scales (Arabic version), scales were consistently positively correlated (fewer problems with fatigue correlated with better overall HRQoL).	*Al-Gamal et al. 2017*
The psychometric properties of the **Brazilian version of the PedsQL MFS** was assessed in childhood cancer patients (n=42 children (8-12 years), n=68 teenagers (13-17 years).<br>**Reliability was acceptable** (Cronbach's alpha between 0.70 and 0.90) for all dimensions except sleep/rest fatigue (Cronbach's alpha=0.55)) and **valid:** Convergent validity: all linear correlation coefficients were greater than 0.40 for the dimension to which the item belonged. Root mean square error of approximation values were within acceptable limits: 0.08-0.10, with 0.098 for self-report and 0.095 for proxy versions. This indicates that the factorial structure of the construct is maintained in the adapted Brazilian model. Comparative fit index was lower than the expected 0.90: 0.699 for self-report and 0.847 for proxy version.	*Nascimento et al. 2015*
In childhood cancer survivors (n=64) the **PedsQL MFS (adaptation to 18-25 year olds)** demonstrated high **reliability** (Cronbach's alpha for Total Fatigue Score=0.95, all subscales≥0.88). Validity was not assessed.	*Robert et al. 2012*
**PROMIS Pediatric Fatigue measures**<br>Studies published after April 2011	
In childhood and adolescent cancer patients (n=96), the **PROMIS Pediatric Fatigue Short Form** was valid: PROMIS fatigue scores correlated significantly with PROMIS performance measures (construct validity; r=-0.68 to -0.3, p<0.01) and with corresponding items of the Symptom Distress Scale (SDS; concurrent validity; p<0.0001). Responsiveness: Fatigue worsened slightly, but not significantly from T1 to T2, then improved significantly to T3. The PROMIS pediatric measures were more responsive across time than the SDS.	*Hinds et al. 2019*
In childhood and adolescent cancer patients (n=96; same sample as ***Hinds et al. 2019***), the **PROMIS Pediatric Fatigue Short Form** was similarly **reliable** (Cronbach's Alpha 0.93-0.96 over all time points and participants) as the Fatigue Scale-Child and Fatigue Scale-Adolescent (0.83-0.94 and 0.93-0.94).<br>**Validity**: PROMIS was correlated with FS-A (r=0.85-0.9) and FS-C (r=0.65-0.88). The area under the curve was 0.72-0.87 for PROMIS (0.84-0.93 for FS-A, 0.84-0.87 for FS-C; differences were not statistically significant). Because of its reliable and valid results, as well as broader applicability in age groups, the authors suggest to use the PROMIS measure for measuring fatigue in patients aged 7-18 years with cancer.	*Macpherson et al. 2018*
In childhood and adolescent brain tumor survivors (n=161; mean 13.9 years at study; mean 5.2 years since diagnosis), the **PROMIS Pediatric Fatigue Computerized Adaptive Testing** (CAT) was compared to the PROMIS Pediatric Fatigue Short Form (SF). Scores were strongly correlated (r=0.976). The authors recommend use of CATs because they enable a more individualized assessment and are less prone to floor or ceiling effects. However, if computers are not available, fixed-length SFs can be used. PROMIS CATs and SFs produced comparable scores for children with a brain tumor.	*Lai et al. 2017*
In childhood cancer patients (n=93) and survivors (n=107), the **PROMIS Pediatric Fatigue Short Form** was **valid**: Known-group validity: Children in the active treatment group had significantly worse scores than children in the survivor group (patients: mean 52.9, survivors: mean 43.8; p<0.001). This remained so even after controlling for demographic variables, tumor type and presence of other health problems.<br>**Reliability** of the tool was not analyzed.	*Hinds et al. 2013*

Table S12 continued

Other measures of CRF in CAYA cancer patients or survivors<br>Studies published after April 2011	
In adolescent and young adult brain tumor survivors (n=142), the **area under the curve (AUC)** of the **Fatigue Thermometer (FT)** as compared to the multidimensional fatigue scale (MFS, gold standard) to detect fatigue was **0.822**.<br>No possible cutoff scores for the FT could be chosen that resulted in a sensitivity and specificity meeting the a priori criteria (sensitivity of >0.90 and specificity of >0.75).	*Brand et al. 2016*
In childhood cancer patients (n=204), the **Turkish Scale for the Assessment of Fatigue in Pediatric Oncology Patients Aged 7-12** was **reliable** (Cronbach's alpha= 0.98 in total for the scale) and **valid** (14 experts assessed content validity, coherence was 0.803; factor analysis explained 84.7% of the variance; statistically significant differences were found in known group comparison). Cut-off point 75 was chosen, **sensitivity was 0.73**, **specificity was 0.93**.	*Kudubes et al. 2014*
In childhood cancer patients (n=184), the **Turkish Scale for the Assessment of Fatigue in Pediatric Oncology Patients Aged 13-18** was **reliable** (Cronbach's alpha= 0.99 in total for the scale) and **valid** (14 experts assessed content validity, coherence was 0.803; factor analysis explained 89.4% of total variance; statistically significant differences were found between groups in known group comparison). Cut-off point 75.5 was chosen (75.4 or below are fatigue cases), **sensitivity was 1.00** and **specificity 0.06**.	*Bektas et al. 2014*
In survivors of Hodgkin's Lymphoma (n=200), the **Multidimensional Fatigue Inventory (MFI)-Brazilian Portuguese version** demonstrated **acceptable reliability** (Cronbach's alpha higher than 0.7 in all dimensions except reduced motivation). Construct validity was analyzed with a factor analysis and explained 65% of the variance.	*Baptista et al. 2012*
In childhood cancer survivors (n=81), a **12-item fatigue questionnaire** was **reliable** (Internal consistency: Cronbach's alpha for the total and each of the three fatigue dimension scores between 0.75 and 0.88) and **valid**: Correlation coefficient between the questionnaire and the Chalder fatigue scale was 0.89, supporting the construct validity of the questionnaire.	*Nagai et al. 2012*
**Overall conclusion**	
In patients of CAYA cancers, evidence suggests that the **Fatigue Scale-Child (FS-C)** and **Fatigue Scale-Adolescent** (FS-A) with its proxy versions (Fatigue Scale-Parents, Fatigue Scale-Staff) is a valid and reliable instrument to measure CRF.	1 systematic review, 5 studies<br>**Level B**
In patients and survivors of CAYA cancers, evidence suggests that the **PedsQL Multidimensional Fatigue Scale** (5-7 years, 8-12 years, 13-18 years, 18-25 years) with its proxy versions (parent versions 2-4 years, 5-7 years) is a valid and reliable instrument to measure CRF.	1 systematic review, 3 studies<br>**Level B**
In patients and survivors of CAYA cancers, evidence suggests that the **PROMIS Pediatric Fatigue measures** (short form, and computerized adaptive testing) is a valid and reliable instrument to measure CRF.	4 studies<br>**Level B**
In patients and survivors of CAYA cancers, some evidence suggests that other measuring instruments, such as the Multidimensional Fatigue Inventory, and the Turkish Scale for the Assessment of Fatigue in Pediatric Oncology Patients (versions 7-12 years, 13-18 years) are valid and reliable instruments to measure CRF.	4 studies<br>**Level C**
In AYA brain tumor survivors, some evidence suggests that a single-item screening measure for CRF (Fatigue Thermometer) is not able to reliably identify clinically significant CRF.	1 study<br>**Level C**

Table S12 continued

6. What is the **effect of individual cognitive behavioral therapy** in the treatment of CRF in CAYA cancer survivors?	
This pilot study in survivors of childhood cancers (n=33; mixed diagnoses; mean 23.1 years at study; mean 13.0 years since diagnosis) found that cognitive behavior therapy was able to significantly **reduce fatigue severity** (Checklist Individual Strength; pretreatment mean 46.2 (SD 4.5) vs. posttreatment mean 28.9 (SD 13.7), p<0.001; large effect size 1.7 (95%CI:1.1-2.3)). 23 of the 33 CCS (70%) included in the study showed a clinically significant improvement, the improvement was even higher in completers of the CBT intervention (n=22/25; 88%). Of the 25 completers, 22 reported that their fatigue level improved significantly or that they were completely recovered.	*Boonstra et al. 2018*
**Overall conclusion**	
**Effect of cognitive behavioral therapy**<br>Some evidence suggests that cognitive behavioral therapy can help to reduce CRF in survivors of childhood, adolescent and young adult cancers.	1 study<br>**Level C**

**7.** What is the effect of **individual physiotherapy** in the treatment of CRF in CAYA cancer survivors?	
**Conclusion single studies**	
No studies identified in survivors of childhood, adolescent and young adult cancers.	
**Overall conclusion**	
**Effect of individual physiotherapy**<br>No studies reported on the effect of individual physiotherapy in the treatment of CRF in patients or survivors of childhood, adolescent and young adult cancers.	0 studies<br>**No studies**

**8.** What is the effect of **a revalidation program** in the treatment of CRF in CAYA cancer survivors?	
**Conclusion single studies**	
No studies identified in survivors of childhood, adolescent and young adult cancers.	
**Overall conclusion**	
**Effect of a revalidation program**<br>No studies reported on the effect of a revalidation program in the treatment of CRF in patients or survivors of childhood, adolescent and young adult cancers.	0 studies<br>**No studies**

Table S12 continued

9. What is the effect of **any intervention** in the treatment of CRF in CAYA cancer survivors?	
**Conclusion single studies CAYA cancer survivors**	
An **adventure-based training** for childhood cancer survivors (n=222; 9-16 years at intervention; 4 training days; 2 weeks, 2, 4, 6, months after randomization respectively; max. 12 participants; team-building games, shuttle runs, rock climbing, etc.) was able to significantly reduce CRF at the 12-month follow-up compared to those in the control group (Fatigue Scale-Child: Intervention Group mean 22.3 (SD 4.2) vs. Control Group mean 28.9 (SD 4.9), p<0.001).	*Li et al. 2018*
In a pilot study, an **exercise intervention** (10 week home-based daily physical activity counselling programme (n=46)) was **significantly** associated with **reduced fatigue** in adult survivors of childhood cancer that at least lasted for 36 weeks (Mean CIS scores ± SD of participants: 81.42±20.14 at T1; 62.62±20.86 at T10 (p<0.0005); 63.67±23.12 at T 36 (p<0.0005 compared to T1)); siblings/peers: 47.39±19.06 at T1; 46.18±17.70 at T10; 42.57± 17.40 at T36).	*Blaauwbroek et al. 2009*
**Conclusion single studies CAYA cancer patients and survivors**	
This intervention study investigated the effect of a fatigue education intervention in childhood cancer patients (n=80; each n=40 in the intervention and control group). The intervention consisted of five educational modules. The intervention and control group were not randomized, and differed regarding mean level of fatigue at baseline (controls having less fatigue). After 3 months, and 6 months the intervention group's mean fatigue scores had increased (indicating less fatigue), whereas the control group's mean fatigue scores had decreased (indicating more fatigue).	*Kudubes et al. 2018*
This was an integrative review including 13 studies in CAYA cancer patients and survivors (of which 4 studies were also included in the Baumann et al. 2013, and 4 in the Chang et al. 2013 review). 5/8 studies found that **exercise** (total n=72; in-patient aerobic exercise/bicycle ergometer, in-patient yoga, weekly step goal with FitBit tracker, exercise combined with quiet leisure activities (reading, listening to music)) reduced CRF in participants. 3/8 studies (total n=51; stationary bicycle exerciser, muscular strength/aerobic fitness, yoga) found no effect. Other interventions that resulted in a decrease in CRF were **healing touch** (1 study, n=9), and **acupressure** (1 study, n=60). Other interventions that found no effect on CRF were exercise plus psychosocial intervention (1 study, n=68; physical exercise plus psychoeducation and cognitive-behavioral techniques), and massage (2 studies; total n=51).	*Nunes et al. 2018*
In a systematic review including 17 studies (3 studies were also included in the Chang et al. 2013 review), **exercise interventions** (in-hospital endurance/strength training, group exercises, educational intervention, home-based exercise program) were associated with **reduced fatigue** in children with cancer, although two (of five) studies found no effect (no effect measure reported).	*Baumann et al. 2013*
In a systematic review including 6 studies (3 studies were also included in the Baumann et al. 2013 review), two **exercise interventions** (16-week physical activity (n=10) and 6-week home-based aerobic exercise (n=24)) were **significantly** associated with **reduced general fatigue** in children with cancer (effect size meta-analysis including 2 studies: -0.76 (95% CI -1.35-0.17)). These exercise interventions did not significantly reduce **total fatigue, sleep or rest fatigue, and cognitive fatigue** in children with cancer.<br>In one study, a **nursing intervention** (education about fatigue and suggestions for activities that can reduce fatigue (n=60)) was associated with **reduced fatigue** in children with cancer (no effect measure reported).	*Chang et al. 2013*
**Overall conclusion**	
**Effect of physical activity interventions**<br>Evidence suggests that physical activity can be useful in the treatment of CRF in survivors of childhood, adolescent, and young adult cancers.	4 studies<br>**Level B**
**Effect of education interventions**<br>Evidence suggests that education about fatigue can help to reduce CRF in childhood, adolescent and young adult cancer patients.	2 studies<br>**Level B**
**Effect of adventure-based training**<br>Some evidence suggests that an adventure-based training can help to reduce CRF in childhood, adolescent and young adult cancer patients.	1 study<br>**Level C**
**Effect of relaxation interventions**<br>Some evidence suggests that relaxation and mindfulness interventions (acupressure, healing touch, massage) can help to reduce CRF in childhood, adolescent and young adult cancer patients.	1 study<br>**Level C**
**Effect of combined physical activity and psychosocial interventions**<br>Some evidence suggests that exercise plus a psychosocial intervention does not decrease CRF in childhood, adolescent, and young adult cancer patients.	1 study<br>**Level C**

**Existing clinical practice guidelines CAYA cancer patients & survivors**	
In this clinical practice guideline for CAYA cancer patients and survivors including 462 randomized trials (only n=6 in CAYA cancer patients or survivors), the use of **physical activity** (preferably aerobic, neuromotor (e.g. yoga, tai chi), or combination), **relaxation and mindfulness** (e.g. acupressure, mindfulness, relaxation techniques, massage therapy) are strongly recommended to reduce CRF. Where these approaches are not feasible or were not successful, **cognitive or cognitive behavioral therapies** may be offered. It was recommended that pharmacological interventions should not be routinely used.	*Robinson et al. 2018*
**Overall conclusion**	
Physical activity, relaxation and mindfulness can be used as interventions for CRF.<br>Cognitive behavioral therapy may be used as an intervention for CRF.<br>The evidence is insufficient about the usefulness and safety of pharmacological interventions.	**Existing guideline**

Table S13. List of measures for cancer-related fatigue that have been validated in survivors of childhood, adolescent, and young adult cancers.

Full name	Versions	No. of items	No. of (sub)scales	Cut-off	Availability	Available languages	Description
Patient-Reported Outcomes Measurement Information System (PROMIS) Pediatric Fatigue measure**[57]**,[a]	Parent Proxy Bank v2.0 Fatigue (5-17 yrs)	23	Unidimensional	..	Yes: http://www.healthmeasures.net/index.php?Itemid=992	**Parent Proxy Bank v2.0 Fatigue**: English<br>**Parent Proxy Short Form v.2.0 Fatigue 10a**: English, German, Hebrew, Italian, Japanese, Korean, Spanish<br>**Pediatric Bank v2.0 Fatigue**: Dutch, English, Spanish<br>**Pediatric Short Form v2.0 Fatigue 10a**: Dutch, English, German, Hebrew, Italian, Japanese, Korean, Simplified Chinese (Mandarin), Spanish	PROMIS® (Patient-Reported Outcomes Measurement Information System) is a set of person-centered measures that evaluates and monitors physical, mental, and social health in adults and children. PROMIS Pediatric Fatigue measure assesses fatigue over the past seven days. Pediatric self-report should be considered the standard, proxy-report should be used if self-report is not possible (child too young, too ill, cognitively impaired). Higher scores indicate more fatigue.
	Parent Proxy Short form v2.0 Fatigue 10a (5-17 yrs)	10					
	Pediatric Bank v2.0 Fatigue (8-17 yrs)	25					
	Pediatric Short Form v2.0 Fatigue 10a (8-17 yrs)	10					
	Several versions of PROMIS Fatigue measure for adult cancer survivors	4-54					
PedsQL Multi-dimensional Fatigue Scale[28] (PedsQL MFS)[a,b]	Toddlers (2-4 years)[a]	18	Three:<br>1. General fatigue<br>2. Sleep/rest fatigue<br>3. Cognitive fatigue	No cut-off; One study[25] used the cut-off ≤1 SD below mean of healthy controls	Yes (for non-funded academic users, registration is required): https://eprovide.mapi-trust.org/instruments/pediatric-quality-of-life-inventory-multidimensional-fatigue-scale	**Self-report (except Toddlers), standard version**: Arabic[a,b,c,d,e], Bosnian[c,d,e], Bulgarian[c,d,e], Croatian[c,d,e], Czech[b,c,d,e], Danish[a,b,c,d,e,f], Dutch[a,b,c,d,e,f], English[a,b,c,d,e,f], Estonian[c,d,e], Finnish[a,b,c,d], French[a,b,c,d,e,f], German[a,b,c,d,e,f], Greek[c,d], Hebrew[b,c,d,e], Hungarian[b,c,d,e], Italian[a,b,c,d,e,f], Japanese[a,b,c,d], Korean[c,d], Lithuanian[a,b,c,d], Malay[c,d], Mandarin Chinese[a,b,c,d,e], Norwegian[b,c,d,e], Polish[a,b,c,d,e], Portuguese[a,b,c,d,e,f], Romanian[a,b,c,d,e], Russian[a,b,c,d,e], Serbian (Cyrillic)[c,d,e], Serbian (Latin)[c,d,e], Slovenian[c,d,e], Spanish[a,b,c,d,e,f], Swedish[b,c,d], Tamil[c,d], Thai[c,d], Turkish[a,b,c,d,e,f], Ukrainian[c,d,e]	The PedsQL MFS is a specific module of the PedsQL™. The PedsQL MFS was designed as a generic symptom-specific instrument to measure fatigue in patients with acute and chronic health conditions as well as healthy school and community populations. For each age-segment there are both an acute (past 7 days) and a standard version (past month), and both parent-proxy reported and self-reported scales (except from toddlers, where only a parent-proxy reported scale is available). There are 6 items for each subscale, and higher total scale scores indicate less fatigue.
	Young child (5-7 years)[b]						
	Child (8-12 years)[c]						
	Adolescent (13-18 years)[d]						
	Young Adult (18-25 years)[e]						
	Adult (>26 years)[f]						
Fatigue Scale[58-60] (FS)[a,b]	Child (FS-C; 7-12 years)[58]	14	Unidimensional	Yes: FS-C reduced version: ≥12[59]<br><br>No cut-offs for the other versions	Yes, by contacting authors: P. Hinds PSHinds@childrensnational.org<br><br>A copy of the FS-A is included in the authors' original article[60]	English (all versions)<br>Chinese (FS-C, FS-A)<br>Spanish (FS-A)	The FS-C and the FS-A measure self-reported fatigue during the previous week among children (7-12 years old) and adolescents (13-18 years old) with cancer. The FS-P assesses parents' perception of their child's fatigue in the last week. The FS-S assesses health professionals' perceptions of the child's fatigue during the last week. All the measures use 5-point Likert scales, and higher total scale scores indicate greater amount of perceived fatigue.
	Child reduced version (10-item FS-C; 7-12 years)[59]	10					
	Adolescent (FS-A; 13-18 years)[60]	14					
	Parent (PFS[58]; FS-P[59, 60])[58-60]	18[58]<br>14[59]					
	Staff (SFS[58]; FS-S[60])[58, 60]	9[58]					

Table S13 continued

Full name	Versions	No. of items	No. of (sub)scales	Cut-off	Availability	Available languages	Description
Multidimensional Fatigue Inventory[61, 62] (MFI-20)[a]	..	20	Five:<br>1. General fatigue<br>2. Physical fatigue<br>3. Mental fatigue<br>4. Reduced motivation<br>5. Reduced activity	75% percentile (moderate fatigue)<br>90% percentile (severe fatigue) of an age- and sex-matched representative sample of the general population[63]	Yes, by contacting authors: E.M.A. Smets e.m.smets@amc.uva.nl<br>A copy of the English MFI-20 can be found online:[64] https://www.med.upenn.edu/cbti/assets/user-content/documents/Multidimensional%20Fatigue%20Inventory%20(MFI).pdf	English<br>French<br>Chinese<br>Hindi	The MFI-20 is a multidimensional short instrument that measures fatigue through five dimensions without containing any somatic item. The five domains of MFI-20 are measured by 20 questions that are scored on a scale from 1 to 7. Higher scores correspond to higher levels of fatigue.
Fatigue Thermometer (FT)[23],[a]	..	1	Unidimensional	Yes: ≥4[65, 66]	..	..	The Fatigue Thermometer combines a visual analogue scale with a numeric rating scale: an image of a vertical thermometer with the ends labeled as "no fatigue" (0) and "worst fatigue imaginable" (10), and the thermometer labeled from 0-10. Scores were categorized as no fatigue, mild fatigue (1-3), moderate fatigue (4-6), and severe fatigue (7-10).[66]
Turkish Scale for the Assessment of Fatigue in Pediatric Oncology Patients[56, 67],[a]	Child: 7-12 years[56]	27	Three:<br>1. General problems<br>2. Sleep problems<br>3. Problems regarding treatment	Yes, 75 for child version and 75.5 for adolescent version	..	Turkish	These multidimensional scales measure fatigue by items that are scored on a scale from 1 to 5. Total score range is 27-135 for the child version, and 32-160 for the adolescent version. Higher scores correspond to lower levels of fatigue.
	Adolescent: 13-18 years[67]	32	Four:<br>4. General problems<br>5. Sleep problems<br>6. Cognitive problems<br>7. Problems regarding treatment				
12-item fatigue questionnaire[48],[a]	..	12	..	..	..	Japanese	This measure assesses fatigue over the last month using a scale from 0 ("not at all") to 3 ("almost every day") for each item. Total fatigue score with a range of 0-36 is computed. Lower scores correspond to lower levels of fatigue.

Table S13 continued

Full name	Versions	No. of items	No. of (sub)scales	Cut-off	Availability	Available languages	Description
Pediatric Functional Assessment of Chronic Illness Therapy-Fatigue[b] (Peds-FACIT-F)[68]	Tested in pediatric patients aged 8-18 years	13	Unidimensional	..	Yes: A copy of the English questionnaire is available for free; others on request: https://www.facit.org/FACITOrg/Questionnaires	Arabic, Bulgarian, Chinese (simplified), Chinese (traditional), Croation, Czech, Danish, Dutch, English, Finnish, French, German, Greek, Hebrew, Hungarian, Italian, Japanese, Korean, Latvian, Norwegian, Polish, Portuguese, Romanian, Russian, Serbian, Slovak, Slovene, Spanish, Swedish, Turkish	This measure assesses fatigue over the last seven days using a scale from 0 "none of the time" to 4 "all of the time". Total score with a range of 0-52 is computed by reverse coding 11/13 items. Lower scores correspond to higher levels of fatigue. The Peds-FACIT-F is a part of the Functional Assessment of Chronic Illness Therapy (FACIT) measurement system. It has been linked to the PROMIS Pediatric Fatigue measure (Cella et al. PROsetta Stone Analysis Report. PROMIS Pediatric Fatigue and Pediatric FACIT Fatigue. Online: http://www.prosettastone.org/LinkingTables1/Linking%20Tables%20Vol3/PROMIS%20Pediatric%20Fatigue%20and%20Pediatric%20FACIT%20Fatigue%20Full%20Report.pdf [accessed September 19th 2019]).
Memorial Symptom Assessment Scale (MSAS)[69],[b]	MSAS 7-12 years[70]<br>MSAS 10-18 years[71]<br>MSAS[69]	8<br>30<br>32	For the adult version (MSAS):<br>1. Global Distress Index (MSAS-GDI)<br>2. Physical Symptom Subscale (MSAS-PHYS)<br>3. Psychological Symptom Subscale (MSAS-PSYCH)<br>4. Total MSAS score (TMSAS)	..	Yes: A copy of the English questionnaire can be found online: http://www.npcrc.org/files/news/memorial_symptom_assessment_scale.pdf<br>MSAS 7-12[70] and MSAS 10-18[71]: The English version of these questionnaires are included in the original articles.	18 translations https://eprovide.mapi-trust.org/instruments/memorial-symptom-assessment-scale	This measure assesses a diverse group of symptoms during the past week. If a symptom is present, then frequency, severity, and distress related to the symptom is assessed.
Daily Fatigue Report Scale[72][b]	..	3/5	Unidimensional	..	Yes: The English version of this questionnaire is included in the original article.	..	This measure assesses fatigue daily (in the evening) with three numerical rating scales (0-10; severity, bother, and interference), and five open questions. Higher scores of the numerical rating scales indicate more severe fatigue, or higher bother/interference.
McCorkle Symptom Distress Scale (SDS)[73][b]	..	13	Unidimensional	..	Yes: A copy of the English questionnaire is available for free (for non-funded academic users, registration is required): https://eprovide.mapi-trust.org/instruments/symptom-distress-scale	French for Canada, Mandarin for Taiwan, Spanish for the USA, Swedish	This measure assesses 11 symptoms on a 5-point Likert scale. One item is about frequency of fatigue (1="seldom", 5="most of the time"). Higher scores indicate higher symptom burden.

[a]Measure validated in studies identified by and included in this clinical practice guideline
[b]Measure validated in studies identified by and included in the Systematic Review by Tomlinson et al. (2013)[74]

References

1. de Ruiter MA, Schouten-van Meeteren AYN, van Vuurden DG, Maurice-Stam H, Gidding C, Beek LR et al. Psychosocial profile of pediatric brain tumor survivors with neurocognitive complaints. Quality of life research : an international journal of quality of life aspects of treatment, care and rehabilitation. 2016;25(2):435-46. doi:10.1007/s11136-015-1091-7.
2. Blaauwbroek R, Bouma MJ, Tuinier W, Groenier KH, de Greef MH, Meyboom-de Jong B et al. The effect of exercise counselling with feedback from a pedometer on fatigue in adult survivors of childhood cancer: a pilot study. Support Care Cancer. 2009;17(8):1041-8. doi:10.1007/s00520-008-0533-y.
3. Barrera M, Teall T, Barr R, Silva M, Greenberg M. Health related quality of life in adolescent and young adult survivors of lower extremity bone tumors. Pediatr Blood Cancer. 2012;58(2):265-73. doi:10.1002/pbc.23017.
4. Calaminus G, Dorffel W, Baust K, Teske C, Riepenhausen M, Bramswig J et al. Quality of life in long-term survivors following treatment for Hodgkin's disease during childhood and adolescence in the German multicentre studies between 1978 and 2002. Support Care Cancer. 2014;22(6):1519-29. doi:10.1007/s00520-013-2114-y.
5. Korinthenberg R, Neuburger D, Nikkhah G, Teske C, Schnabel K, Calaminus G. Assessing quality of life in long-term survivors after 125I brachytherapy for low-grade glioma in childhood. Neuropediatrics. 2011;42(3):110-5. doi:10.1055/s-0031-1283111.
6. Sato I, Higuchi A, Yanagisawa T, Murayama S, Kumabe T, Sugiyama K et al. Impact of late effects on health-related quality of life in survivors of pediatric brain tumors: motility disturbance of limb(s), seizure, ocular/visual impairment, endocrine abnormality, and higher brain dysfunction. Cancer Nurs. 2014;37(6):E1-e14. doi:10.1097/ncc.0000000000000110.
7. Sterkenburg AS, Hoffmann A, Gebhardt U, Warmuth-Metz M, Daubenbuchel AM, Muller HL. Survival, hypothalamic obesity, and neuropsychological/psychosocial status after childhood-onset craniopharyngioma: newly reported long-term outcomes. Neuro-oncology. 2015;17(7):1029-38. doi:10.1093/neuonc/nov044.
8. Clanton NR, Klosky JL, Li C, Jain N, Srivastava DK, Mulrooney D et al. Fatigue, vitality, sleep, and neurocognitive functioning in adult survivors of childhood cancer: a report from the Childhood Cancer Survivor Study. Cancer. 2011;117(11):2559-68. doi:10.1002/cncr.25797.
9. Fortmann J, Fisher A, Hough R, Gregory A, Pugh G. Sleep Quality, Fatigue, and Quality of Life Among Teenage and Young Adult Cancer Survivors. J Adolesc Young Adult Oncol. 2018;7(4):465-71. doi:10.1089/jayao.2018.0004.
10. Kenney LB, Nancarrow CM, Najita J, Vrooman LM, Rothwell M, Recklitis C et al. Health status of the oldest adult survivors of cancer during childhood. Cancer. 2010;116(2):497-505. doi:10.1002/cncr.24718.
11. Mulrooney DA, Ness KK, Neglia JP, Whitton JA, Green DM, Zeltzer LK et al. Fatigue and sleep disturbance in adult survivors of childhood cancer: a report from the childhood cancer survivor study (CCSS). Sleep. 2008;31(2):271-81.
12. Rach AM, Crabtree VM, Brinkman TM, Zeltzer L, Marchak JG, Srivastava D et al. Predictors of fatigue and poor sleep in adult survivors of childhood Hodgkin's lymphoma: a report from the Childhood Cancer Survivor Study. Journal of cancer survivorship : research and practice. 2017;11(2):256-63. doi:10.1007/s11764-016-0583-y.
13. Aksnes LH, Hall KS, Jebsen N, Fossa SD, Dahl AA. Young survivors of malignant bone tumours in the extremities: a comparative study of quality of life, fatigue and mental distress. Support Care Cancer. 2007;15(9):1087-96. doi:10.1007/s00520-007-0227-x.
14. Hamre H, Zeller B, Kanellopoulos A, Kiserud CE, Aakhus S, Lund MB et al. High Prevalence of Chronic Fatigue in Adult Long-Term Survivors of Acute Lymphoblastic Leukemia and Lymphoma During Childhood and Adolescence. J Adolesc Young Adul. 2013;2(1):2-9. doi:10.1089/jayao.2012.0015.
15. Hamre H, Zeller B, Kanellopoulos A, Ruud E, Fossa SD, Loge JH et al. Serum cytokines and chronic fatigue in adults surviving after childhood leukemia and lymphoma. Brain Behav Immun. 2013;30:80-7. doi:10.1016/j.bbi.2013.01.006.
16. Johannsdottir IM, Hjermstad MJ, Moum T, Wesenberg F, Hjorth L, Schroder H et al. Increased prevalence of chronic fatigue among survivors of childhood cancers: a population-based study. Pediatr Blood Cancer. 2012;58(3):415-20. doi:10.1002/pbc.23111.
17. Johannsdottir IMR, Hamre H, Fossa SD, Loge JH, Drolsum L, Lund MB et al. Adverse Health Outcomes and Associations with Self-Reported General Health in Childhood Lymphoma Survivors. J Adolesc Young Adult Oncol. 2017;6(3):470-6. doi:10.1089/jayao.2017.0018.
18. Zeller B, Loge JH, Kanellopoulos A, Hamre H, Wyller VB, Ruud E. Chronic fatigue in long-term survivors of childhood lymphomas and leukemia: persistence and associated clinical factors. J Pediatr Hematol Oncol. 2014;36(6):438-44. doi:10.1097/mph.0000000000000051.
19. Ho KY, Li WH, Lam KW, Chui SY, Chan CF. Psychometric properties of the Chinese version of the fatigue scale-adolescent. BMC Cancer. 2015;15:938. doi:10.1186/s12885-015-1945-y.

20. Daniel L, Kazak AE, Li Y, Hobbie W, Ginsberg J, Butler E et al. Relationship between sleep problems and psychological outcomes in adolescent and young adult cancer survivors and controls. Support Care Cancer. 2016;24(2):539-46. doi:10.1007/s00520-015-2798-2.
21. Langeveld NE, Grootenhuis MA, Voute PA, de Haan RJ, van den Bos C. No excess fatigue in young adult survivors of childhood cancer. Eur J Cancer. 2003;39(2):204-14.
22. Nies M, Klein Hesselink MS, Huizinga GA, Sulkers E, Brouwers AH, Burgerhof JGM et al. Long-Term Quality of Life in Adult Survivors of Pediatric Differentiated Thyroid Carcinoma. J Clin Endocrinol Metab. 2017;102(4):1218-26. doi:10.1210/jc.2016-2246.
23. Brand SR, Chordas C, Liptak C, Manley P, Recklitis C. Screening for fatigue in adolescent and young adult pediatric brain tumor survivors: accuracy of a single-item screening measure. Support Care Cancer. 2016;24(8):3581-7. doi:10.1007/s00520-016-3150-1.
24. Cheung YT, Brinkman TM, Mulrooney DA, Mzayek Y, Liu W, Banerjee P et al. Impact of sleep, fatigue, and systemic inflammation on neurocognitive and behavioral outcomes in long-term survivors of childhood acute lymphoblastic leukemia. Cancer. 2017;123(17):3410-9. doi:10.1002/cncr.30742.
25. Frederick NN, Kenney L, Vrooman L, Recklitis CJ. Fatigue in adolescent and adult survivors of non-CNS childhood cancer: a report from project REACH. Support Care Cancer. 2016;24(9):3951-9. doi:10.1007/s00520-016-3230-2.
26. Gordijn MS, van Litsenburg RR, Gemke RJ, Huisman J, Bierings MB, Hoogerbrugge PM et al. Sleep, fatigue, depression, and quality of life in survivors of childhood acute lymphoblastic leukemia. Pediatr Blood Cancer. 2013;60(3):479-85. doi:10.1002/pbc.24261.
27. Graef DM, Phipps S, Parris KR, Martin-Elbahesh K, Huang L, Zhang H et al. Sleepiness, Fatigue, Behavioral Functioning, and Quality of Life in Survivors of Childhood Hematopoietic Stem Cell Transplant. Journal of pediatric psychology. 2016;41(6):600-9. doi:10.1093/jpepsy/jsw011.
28. Varni JW, Burwinkle TM, Katz ER, Meeske K, Dickinson P. The PedsQL in pediatric cancer: reliability and validity of the Pediatric Quality of Life Inventory Generic Core Scales, Multidimensional Fatigue Scale, and Cancer Module. Cancer. 2002;94(7):2090-106. doi:10.1002/cncr.10428.
29. Mort S, Lahteenmaki PM, Matomaki J, Salmi TT, Salantera S. Fatigue in young survivors of extracranial childhood cancer: a Finnish nationwide survey. Oncol Nurs Forum. 2011;38(6):E445-54. doi:10.1188/11.onf.e445-e454.
30. Spathis A, Hatcher H, Booth S, Gibson F, Stone P, Abbas L et al. Cancer-Related Fatigue in Adolescents and Young Adults After Cancer Treatment: Persistent and Poorly Managed. J Adolesc Young Adult Oncol. 2017;6(3):489-93. doi:10.1089/jayao.2017.0037.
31. Lowe K, Escoffery C, Mertens AC, Berg CJ. Distinct health behavior and psychosocial profiles of young adult survivors of childhood cancers: a mixed methods study. Journal of cancer survivorship : research and practice. 2016. doi:10.1007/s11764-015-0508-1.
32. Meeske KA, Siegel SE, Globe DR, Mack WJ, Bernstein L. Prevalence and correlates of fatigue in long-term survivors of childhood leukemia. J Clin Oncol. 2005;23(24):5501-10. doi:10.1200/jco.2005.03.210.
33. Zeltzer LK, Chen E, Weiss R, Guo MD, Robison LL, Meadows AT et al. Comparison of psychologic outcome in adult survivors of childhood acute lymphoblastic leukemia versus sibling controls: a cooperative Children's Cancer Group and National Institutes of Health study. J Clin Oncol. 1997;15(2):547-56.
34. Karimi M, Cox AD, White SV, Karlson CW. Fatigue, Physical and Functional Mobility, and Obesity in Pediatric Cancer Survivors. Cancer Nurs. 2019. doi:10.1097/NCC.0000000000000712.
35. Zebrack BJ, Chesler MA. Quality of life in childhood cancer survivors. Psychooncology. 2002;11(2):132-41.
36. Vannatta K, Gartstein MA, Short A, Noll RB. A controlled study of peer relationships of children surviving brain tumors: teacher, peer, and self ratings. Journal of pediatric psychology. 1998;23(5):279-87.
37. Kanellopoulos A, Hamre HM, Dahl AA, Fossa SD, Ruud E. Factors associated with poor quality of life in survivors of childhood acute lymphoblastic leukemia and lymphoma. Pediatr Blood Cancer. 2013;60(5):849-55. doi:10.1002/pbc.24375.
38. Adams MJ, Lipsitz SR, Colan SD, Tarbell NJ, Treves ST, Diller L et al. Cardiovascular status in long-term survivors of Hodgkin's disease treated with chest radiotherapy. J Clin Oncol. 2004;22(15):3139-48. doi:10.1200/jco.2004.09.109.
39. Arpaci T, Kilicarslan Toruner E. Assessment of problems and symptoms in survivors of childhood acute lymphoblastic leukaemia. Eur J Cancer Care (Engl). 2016;25(6):1034-43. doi:10.1111/ecc.12561.
40. Berg C, Neufeld P, Harvey J, Downes A, Hayashi RJ. Late Effects of Childhood Cancer, Participation, and Quality of Life of Adolescents. Otjr-Occupation Participation and Health. 2009;29(3):116-24. doi:10.3928/15394492-20090611-04.
41. Berg C, Hayashi RJ. Participation and Self-Management Strategies of Young Adult Childhood Cancer Survivors. Otjr-Occupation Participation and Health. 2013;33(1):21-30. doi:10.3928/15394492-20120607-01.

42. Enskar K, von Essen L. Prevalence of aspects of distress, coping, support and care among adolescents and young adults undergoing and being off cancer treatment. Eur J Oncol Nurs. 2007;11(5):400-8. doi:10.1016/j.ejon.2007.01.003.
43. Geenen MM, Cardous-Ubbink MC, Kremer LCM, Van Den Bos C, Van Der Pal HJH, Heinen RC et al. Medical assessment of adverse health outcomes in long-term survivors of childhood cancer. Journal of the American Medical Association. 2007;297(24):2705-15. doi:10.1001/jama.297.24.2705.
44. Khan RB, Hudson MM, Ledet DS, Morris EB, Pui CH, Howard SC et al. Neurologic morbidity and quality of life in survivors of childhood acute lymphoblastic leukemia: a prospective cross-sectional study. Journal of cancer survivorship : research and practice. 2014;8(4):688-96. doi:10.1007/s11764-014-0375-1.
45. Macpherson CF, Hooke MC, Friedman DL, Campbell K, Withycombe J, Schwartz CL et al. Exercise and Fatigue in Adolescent and Young Adult Survivors of Hodgkin Lymphoma: A Report from the Children's Oncology Group. J Adolesc Young Adult Oncol. 2015;4(3):137-40. doi:10.1089/jayao.2015.0013.
46. Manley PE, McKendrick K, McGillicudy M, Chi SN, Kieran MW, Cohen LE et al. Sleep dysfunction in long term survivors of craniopharyngioma. Journal of neuro-oncology. 2012;108(3):543-9. doi:10.1007/s11060-012-0859-7.
47. McClellan W, Klemp JR, Krebill H, Ryan R, Nelson EL, Panicker J et al. Understanding the functional late effects and informational needs of adult survivors of childhood cancer. Oncol Nurs Forum. 2013;40(3):254-62. doi:10.1188/13.onf.254-262.
48. Nagai A, Zou N, Kubota M, Kojima C, Adachi S, Usami I et al. Fatigue in survivors of childhood acute lymphoblastic and myeloid leukemia in Japan. Pediatrics international : official journal of the Japan Pediatric Society. 2012;54(2):272-6. doi:10.1111/j.1442-200X.2011.03530.x.
49. Yi J, Kim MA, Tian T. Perceived long-term and physical health problems after cancer: adolescent and young adult survivors of childhood cancer in Korea. Eur J Oncol Nurs. 2014;18(2):145-50. doi:10.1016/j.ejon.2013.11.006.
50. Atkins D, Best D, Briss PA, Eccles M, Falck-Ytter Y, Flottorp S et al. Grading quality of evidence and strength of recommendations. BMJ. 2004;328(7454):1490. doi:10.1136/bmj.328.7454.1490.
51. Gibbons RJ, Smith S, Antman E, American College of C, American Heart A. American College of Cardiology/American Heart Association clinical practice guidelines: Part I: where do they come from? Circulation. 2003;107(23):2979-86. doi:10.1161/01.CIR.0000063682.20730.A5.
52. Children's Oncology Group. Long-Term Follow-Up Guidelines for Survivors of Childhood, Adolescent, and Young Adult Cancers V4.0. 2013. http://www.survivorshipguidelines.org/. Accessed 28 July 2016.
53. Dutch Childhood Oncology Group. Guidelines for follow-up in survivors of childhood cancer 5 years after diagnosis. SKION, Den Haag/Amsterdam; the Netherlands: 2010. Available online: https://www.skion.nl/workspace/uploads/vertaling-richtlijn-LATER-versie-final-okt-2014_2.pdf2010.
54. United Kingdom Children's Cancer Study Group Late Effects Group. Therapy Based Long Term Follow Up - Practice Statement: 2005. Available online: http://www.uhb.nhs.uk/Downloads/pdf/CancerPbTherapyBasedLongTermFollowUp.pdf2005.
55. Scottish Intercollegiate Guidelines Network (SIGN). Long term follow up of survivors of childhood cancer. A national clinical guideline. 2013. http://www.sign.ac.uk/pdf/sign76.pdf. Accessed 28 July 2016.
56. Kudubes AA, Bektas M, Ugur O. Developing a scale for the assessment of fatigue in pediatric oncology patients aged 7-12 for children and parents. Asian Pac J Cancer Prev. 2014;15(23):10199-207.
57. Hinds PS, Nuss SL, Ruccione KS, Withycombe JS, Jacobs S, DeLuca H et al. PROMIS pediatric measures in pediatric oncology: valid and clinically feasible indicators of patient-reported outcomes. Pediatr Blood Cancer. 2013;60(3):402-8. doi:10.1002/pbc.24233.
58. Hockenberry MJ, Hinds PS, Barrera P, Bryant R, Adams-McNeill J, Hooke C et al. Three instruments to assess fatigue in children with cancer: the child, parent and staff perspectives. J Pain Symptom Manage. 2003;25(4):319-28.
59. Hinds PS, Yang J, Gattuso JS, Hockenberry M, Jones H, Zupanec S et al. Psychometric and clinical assessment of the 10-item reduced version of the Fatigue Scale-Child instrument. J Pain Symptom Manage. 2010;39(3):572-8. doi:10.1016/j.jpainsymman.2009.07.015.
60. Hinds PS, Hockenberry M, Tong X, Rai SN, Gattuso JS, McCarthy K et al. Validity and reliability of a new instrument to measure cancer-related fatigue in adolescents. J Pain Symptom Manage. 2007;34(6):607-18. doi:10.1016/j.jpainsymman.2007.01.009.
61. Smets EM, Garssen B, Bonke B, De Haes JC. The Multidimensional Fatigue Inventory (MFI) psychometric qualities of an instrument to assess fatigue. J Psychosom Res. 1995;39(3):315-25.
62. Baptista RL, Biasoli I, Scheliga A, Soares A, Brabo E, Morais JC et al. Psychometric properties of the multidimensional fatigue inventory in Brazilian Hodgkin's lymphoma survivors. J Pain Symptom Manage. 2012;44(6):908-15. doi:10.1016/j.jpainsymman.2011.12.275.

63. Kuhnt S, Ernst J, Singer S, Ruffer JU, Kortmann RD, Stolzenburg JU et al. Fatigue in cancer survivors--prevalence and correlates. Onkologie. 2009;32(6):312-7. doi:10.1159/000215943.
64. Shahid A, Wilkinson K, Marcu S, Shapiro CM. Multidimensional Fatigue Inventory (MFI). In: Shahid A, Wilkinson K, Marcu S, Shapiro CM, editors. STOP, THAT and One Hundred Other Sleep Scales. New York, NY: Springer; 2011.
65. Mock V, Atkinson A, Barsevick A, Cella D, Cimprich B, Cleeland C et al. NCCN Practice Guidelines for Cancer-Related Fatigue. Oncology (Williston Park). 2000;14(11A):151-61.
66. Temel JS, Pirl WF, Recklitis CJ, Cashavelly B, Lynch TJ. Feasibility and validity of a one-item fatigue screen in a thoracic oncology clinic. J Thorac Oncol. 2006;1(5):454-9.
67. Bektas M, Kudubes AA. Developing scales for the assessment of fatigue in Turkish pediatric oncology patients aged 13-18 and their parents. Asian Pac J Cancer Prev. 2014;15(22):9891-8.
68. Lai JS, Cella D, Kupst MJ, Holm S, Kelly ME, Bode RK et al. Measuring fatigue for children with cancer: development and validation of the pediatric Functional Assessment of Chronic Illness Therapy-Fatigue (pedsFACIT-F). J Pediatr Hematol Oncol. 2007;29(7):471-9. doi:10.1097/MPH.0b013e318095057a.
69. Portenoy RK, Thaler HT, Kornblith AB, Lepore JM, Friedlander-Klar H, Kiyasu E et al. The Memorial Symptom Assessment Scale: an instrument for the evaluation of symptom prevalence, characteristics and distress. Eur J Cancer. 1994;30A(9):1326-36. doi:10.1016/0959-8049(94)90182-1.
70. Collins JJ, Devine TD, Dick GS, Johnson EA, Kilham HA, Pinkerton CR et al. The measurement of symptoms in young children with cancer: the validation of the Memorial Symptom Assessment Scale in children aged 7-12. J Pain Symptom Manage. 2002;23(1):10-6. doi:10.1016/s0885-3924(01)00375-x.
71. Collins JJ, Byrnes ME, Dunkel IJ, Lapin J, Nadel T, Thaler HT et al. The measurement of symptoms in children with cancer. J Pain Symptom Manage. 2000;19(5):363-77. doi:10.1016/s0885-3924(00)00127-5.
72. Erickson JM, Beck SL, Christian B, Dudley WN, Hollen PJ, Albritton K et al. Patterns of fatigue in adolescents receiving chemotherapy. Oncol Nurs Forum. 2010;37(4):444-55. doi:10.1188/10.ONF.444-455.
73. McCorkle R. The measurement of symptom distress. Semin Oncol Nurs. 1987;3(4):248-56.
74. Tomlinson D, Hinds PS, Ethier MC, Ness KK, Zupanec S, Sung L. Psychometric properties of instruments used to measure fatigue in children and adolescents with cancer: a systematic review. J Pain Symptom Manage. 2013;45(1):83-91. doi:10.1016/j.jpainsymman.2012.02.010.
